# POU5F1 promotes the proliferation, migration, and invasion of gastric cancer cells by reducing the ubiquitination level of TRAF6

**DOI:** 10.1038/s41419-023-06332-8

**Published:** 2023-12-07

**Authors:** Wenshuo Yang, Xiaohan Cui, Danping Sun, Guorui Sun, Zhibo Yan, Meng Wei, Zuoyang Wang, Wenbin Yu

**Affiliations:** Department of General Surgery, Qilu Hospital of Shandong University, 250012 Jinan, Shandong China

**Keywords:** Gastrointestinal cancer, Epigenetics

## Abstract

POU5F1 plays an important role in maintaining the cancer stem cell (CSC) -like properties of gastric cancer (GC) cells. The impact of POU5F1 on the proliferation and metastasis of GC was examined, along with the potential of ATRA as a specific therapeutic agent for GC. The dysregulation of POU5F1 expression in GC tissues was analyzed using public databases and bioinformatics techniques, and the disparity in POU5F1 expression between normal gastric tissues and GC tissues was further assessed through western blot, RT-qPCR, and immunohistochemistry. The present study aimed to investigate the impact of POU5F1 on the proliferation, migration, and invasion of GC cells through both in vivo and in vitro experiments. Additionally, the effects of ATRA on the proliferation, migration, and invasion of GC cells were examined using in vivo and in vitro approaches. Our findings revealed a significant upregulation of POU5F1 in GC tissues, which was found to be associated with a poorer prognosis in patients with GC. Moreover, POU5F1 was observed to enhance the proliferation, migration, and invasion of GC cells in vitro, as well as promote subcutaneous tumor growth and lung metastasis of GC cells in vivo. The overexpression of POU5F1 mechanistically triggers the process of Epithelial-mesenchymal transition (EMT) by down-regulating E-Cadherin and up-regulating N-Cadherin and VIM. POU5F1 hinders the ubiquitination of TRAF6 through negative regulation of TRIM59, thereby facilitating the activation of the NF-κB pathway. Furthermore, the administration of ATRA effectively impedes the proliferation, migration, and invasion of GC cells by suppressing the expression of POU5F1. The upregulation of POU5F1 elicits EMT, fosters the initiation of the NF-κB signaling pathway in GC cells, and stimulates the proliferation, invasion, and metastasis of GC cells. All-trans retinoic acid (ATRA) can impede these POU5F1-induced effects, thereby potentially serving as an adjunctive therapeutic approach for GC.

## Background

Gastric cancer (GC) ranks as the fifth most prevalent malignancy on a global scale, with an annual incidence exceeding 1 million cases [1]. Due to its tendency for late-stage diagnosis, GC exhibits a high mortality rate, resulting in approximately 784,000 deaths worldwide in 2018 [2]. Despite extensive research conducted on the biological aspects of GC, surgical or endoscopic resection remains the primary therapeutic approach for this condition [3]. Despite a decline in the mortality rate of GC in recent years, patients with advanced GC continue to experience a 5-year overall survival (OS) rate of only 20–30% [4]. Consequently, there is a pressing need to investigate novel targets for the diagnosis and treatment of GC, with the aim of enhancing patients’ OS duration and therapeutic outcomes.

The transcription factor POU5F1, belonging to the POU homeobox gene family and commonly referred to as Oct4, is crucial for the preservation of self-renewal capabilities in embryonic stem (ES) cells. Additionally, it facilitates the maintenance of stemness in cancer stem-like cells (CSLCs) and contributes to the initiation and spread of tumors [5, 6]. Some results have shown that POU5F1 can enhance tumor drug resistance [7]. In the context of lung cancer, cells exhibiting high levels of POU5F1 expression have been observed to display resistance towards conventional treatment methods, including cisplatin, etoposide, paclitaxel, and targeted therapy with gefitinib [8, 9]. Furthermore, tumors characterized by elevated POU5F1 expression have exhibited a higher propensity for metastasis and a lower overall survival rate compared to tumors with lower levels of POU5F1 expression [10]. In addition, numerous studies have conducted analyses on the expression of POU5F1 mRNA and protein within various tumor types, including bladder cancer, rectal cancer, hepatocellular carcinoma, esophageal squamous cell carcinoma, among others. The findings consistently indicate that elevated levels of POU5F1 mRNA or protein are significantly associated with unfavorable prognoses [11–15]. Additionally, a separate literature source has reported a negative correlation between POU5F1 expression and cancer differentiation in GC, whereby higher levels of POU5F1 expression are linked to shorter patient survival rates [16]. Suggesting that POU5F1 may be a new tumor biological and prognostic marker and can be used as a potential therapeutic target [17].

In this study, it was observed that POU5F1 exhibited heightened expression in both GC tumor tissues and cell lines. The overexpression of POU5F1 in GC cells was found to stimulate tumor proliferation, migration, and invasion, both in vitro and in vivo. Furthermore, POU5F1 down-regulates the expression of TRIM59, resulting in a decrease in the ubiquitination level of TRAF6. This subsequently facilitated the activation of the NF-κB signaling pathway, ultimately leading to an augmentation of EMT in GC cells.

## Materials and methods

### Cell lines and cell culture

The human GC cell lines BGC823, MKN45, SGC7901, Ncl-N87, HGC27, and human gastric mucosal cell GES-1 were obtained from BeNa Technology (Hangzhou, Zhejiang, China). These cells were cultured in a CO_2_ incubator at 37 °C using 1640 medium (C3010-0500, Vivacell, Shanghai, China) supplemented with 10% fetal bovine serum (26010074, Gibco, Paisley, UK) and 1% penicillin/streptomycin (P1400, Solarbio, Beijing, China). The compounds all-trans retinoic acid (ATRA), Cycloheximide (CHX), and PR-619 were purchased from MedChemExpress (HY-14649, HY-12320, HY-13814, New Jersey, USA) and were dissolved in 10 mM DMSO. None of the cells in this study were passaged more than 20 times. All cell lines were STR validated and were verified as mycoplasma negative.

### Clinical materials

The GC tissue samples and paired adjacent normal tissues were procured from 150 GC patients admitted to Qilu Hospital of Shandong University between January 2022 and December 2022. These patients were not subjected to any preoperative interventions, such as radiotherapy or chemotherapy, and informed consent was duly obtained from all participants. This study was ethically approved by the Qilu Hospital of Shandong University Research Ethics Committee, and complied with the ethical guidelines of the World Medical Association Declaration of Helsinki.

### Whole-genome RNA sequencing and functional enrichment analyses

The gastric cancer cell line MKN45 was partitioned into an intervention group, which was transfected with POU5F1-specific siRNA, and a control group, which was transfected with a negative control. Total RNA was extracted using the RNeasy mini kit (74104, Qiagen, Dusseldorf, German). Paired-end libraries were synthesized using the TruSeq™ RNA Sample Preparation Kit (Illumina, California, USA). The library construction and sequencing procedures were carried out by Applied Protein Technology (Shanghai, China).

The study utilized whole-genome RNA sequencing data to conduct Gene Ontology (GO) enrichment analysis and Kyoto Encyclopedia of Genes and Genomes (KEGG) pathway enrichment analysis. These analyses encompassed biological processes (BP), molecular functions (MF), cellular components (CC), and pathways. A *p*-value < 0.05 was deemed statistically significant.

### Small interfering RNA (si-RNA transfection)

To suppress the expression of TRIM59, TRAF6, and TLR4 in GC cells, the cells were cultured in 6-well plates and subsequently transfected with si-RNA using Lipofectamine^TM^3000 (L3000015, California, Invitrogen) upon reaching 70% confluence.

### Plasmids

For the purpose of POU5F1 knockdown and overexpression, the shRNA sequence targeting POU5F1 was incorporated into the pLVX-shRNA Lenti-vector, while the POU5F1 fragment was integrated into the overexpression vector pLV6ltr-ZsGreen-Puro-CMV. Consequently, POU5F1 lentivirus knockdown and overexpression vectors were generated. The transfection of the vector into GC cells was facilitated using Lipofectamine^TM^3000, and stable cell lines were established through puromycin-based selection. Subsequently, the levels of knockdown and overexpression were assessed at the protein and RNA levels using western blot and real-time quantitative polymerase chain reaction (RT-qPCR) techniques.

### Western blot analysis

Proteins were extracted from GC cells, GC tissues, and mouse tissues using RIPA lysis buffer (P0013B, Beyotime Biotechnology, Shanghai, China) supplemented with 1% protease inhibitors and 1% phosphatase inhibitors. The separation of proteins was achieved through 10% SDS-PAGE and subsequently transferred onto a PVDF membrane (ISEQ00010, Millipore, Massachusetts, USA). The PVDF membranes were then blocked with 5% skim milk for 1.5 h at room temperature, followed by overnight incubation with primary antibodies at 4°C. The membranes were then incubated with secondary antibodies of goat anti-rabbit antibody (1:10000, ZB-2301, ZSGB-BIO, Beijing, China) and goat anti-mouse antibody (1:10000, ZB-2305, ZSGB-BIO) for 2 h at room temperature. ECL luminescent solution (WBKLS0500, Millipore) was used for visualization. The primary antibodies include GAPDH (1:1000, # 5174 S, CST, Massachusetts, USA), POU5F1 (1:1000, #2750, CST), Vimentin (1:1000, #5741, CST), N-Cadherin (1:1000, A0433, ABclonal, Wuhan, China), E-Cadherin (1:1000, A11492, ABclonal), TRIM59 (1:1000, NBP1-59777, NOVUS, Colorado, USA), TRAF6 (1:1000, #8028, CST), TLR4 (1:1000, 66350-1-Ig, Proteintech, Illinois, USA), TIRAP (1:1000,A9663, ABclonal), NF-κB (1:1000, #8242S, CST), P-NF-κB (1:1000, #3033S, CST), PCNA (1:1000, 10205-2-AP, Proteintech).

### Real-time quantitative PCR (RT-qPCR)

Total RNA was extracted from GC tissues and cells using the RNA extraction kit (220011, Fastagen, Shanghai, China). Following measurement of the concentration, 1 μg of total RNA was reverse transcribed using the HiScript III RT SuperMix for qPCR (R323-01, Vazyme, Nanjing, China) and subsequently amplified by qPCR using the ChamQ Universal SYBR qPCR Master Mix (Q711, Vazyme). Primer sequences were synthesized by Tsingke Biotechnology Co., Ltd (Beijing, China). The reference gene β-actin was chosen, and the relative expression was determined using the 2^-ΔΔCt^ method.

### Immunohistochemistry (IHC)

Gastric cancer tissues and animal tissues were fixed using a 4% paraformaldehyde solution and subsequently embedded in paraffin. The resulting tissue was then sliced into sections with a thickness of 4μm. Following deparaffinization using xylene and gradient ethanol solutions, antigen repair was conducted using a citrate repair solution. Subsequently, the sections were incubated in a 3% hydrogen peroxide solution for a duration of 30 min and blocked. POU5F1 (1:200, #2750, CST), Vimentin (1:200, #5741, CST), N-Cadherin (1:200, A0433, ABclonal), E-Cadherin (1:200, A11492, ABclonal), Ki67 (1:200, A20018, ABclonal) and PCNA (1:200, 10205-2-AP, Proteintech) were added to the sections and incubated overnight at 4 °C. The cells were subsequently incubated with secondary antibodies conjugated with HRP for a duration of 1 h at ambient temperature. To assess the degree of antibody binding, DAB staining was employed, while the nuclei were counterstained with hematoxylin.

### Immunoprecipitation (IP)

TRIM59-specific siRNA, TRIM59 overexpression plasmid, and UB-HA plasmid were transfected into GC cells. After 24 h of transfection, cell lysates were prepared by lysing the cells using IP lysis buffer (150 mM NaCl, 50 mM Tris-HCl, 0.5% NP-40, and 2 mM EDTA) on ice for 30 min, followed by centrifugation at 14,000 × *g* for 15 min at 4 °C. The IP group was formed by incubating 300 μL of protein and 3 μL of specific antibody (100:1) overnight at 4 °C. Then, the IP group proteins were subjected to incubation with 40 μL of Protein A/G Plus‑Agarose (sc-2003, Santa Cruz, California, USA) at a temperature of 4 °C for a duration of 10 h. Following this, the supernatant was obtained through centrifugation at 1000 × *g* for 5 min at 4 °C, a process that was repeated three times. Finally, a protein loading buffer was introduced.

### CUT & Tag, library construction and DNA sequencing

A total of 1 × 10^5^ GC cells per sample were obtained and subjected to analysis using the Hyperactive Universal CUT&Tag Assay Kit for Illumina (TD904, Vazyme), following the recommended protocol. The cells were immobilized using activated ConA Beads Pro for a duration of 10 min at room temperature. Subsequently, the supernatant was discarded and primary anti-POU5F1 antibody was introduced, followed by overnight incubation at 4 °C. On the following day, secondary antibodies were diluted with Dig-wash Buffer at a ratio of 1:100, and subjected to rotation for 60 min at ambient temperature. Subsequently, the cells were washed and incubated with pA/G-Tnp Pro for 60 min at room temperature with rotation. After washing, diluted TTBL was introduced and incubated at 37 °C for 60 min. Subsequently, 10% SDS and the suitable quantity of DNA Spike-in were added, mixed thoroughly, and incubated at 55°C for 10 min before collecting the supernatant. The collected supernatant was supplemented with activated DNA Extra Beads Pro and incubated at room temperature for a duration of 20 min. Following the incubation period, the supernatant was discarded after being treated with 1× B&W Buffer for 30 s at room temperature, a process that was repeated once. The DNA Extra Beads Pro was then resuspended by adding an additional 15ul of ddH_2_O, and the resulting samples were subjected to PCR amplification. Subsequently, the PCR products underwent purification and were sequenced on the Illumina platform.

### Wound healing assay

Cells were cultured overnight at 8 × 10^5^/well in 6-well plates, and after the cells adhered, a 200 μL gun tip was used to make a scratch in the well. They were washed using PBS and 1640 medium without FBS was added. The wounds were observed and measured under an optical microscope every 6 h, and the wound healing was evaluated using ImageJ.

### EdU assay

GC cells were cultured in 12-well plates and subjected to EdU detection utilizing the Cell-Light EdU Apollo567 In Vitro Kit (C10310-1, RIBOBIO, Guangdong, China). Subsequently, the cells were observed and imaged using a fluorescence microscope (Nikon Ti2-U), and the rate of EdU incorporation was determined by calculating the ratio of EdU-positive cells to total DAPI-positive cells using ImageJ.

### Transwell assay

GC cells suspended in serum-free 1640 medium were diluted to 5×10^5^/mL, and 100 μL was incubated into the upper chamber of Transwell for migration. 600 μL 1640 medium containing 10% FBS or other treatments was added to the well plate. After 36 h, non-migrating cells were removed, the cells in the lower chamber were fixed and stained with crystal violet, and three random fields were selected for filming and counting.

100 μL Matrigel solution was evenly applied to the upper chamber wall, and the rest of the procedure was the same as that of cell migration assay to detect the invasion of GC cells.

### Clonogenic assay

GC cells were inoculated at a density of 1000 cells per well in 6-well plates and maintained in 1640 medium supplemented with 10% fetal bovine serum for a duration of 14 days, with regular medium replacement every 3 days. Following the incubation period, the cells were immobilized using a 4% paraformaldehyde solution and subsequently subjected to staining using a 0.1% crystal violet dye.

### In vivo assay

BALB/c nude mice at 4 weeks of age were obtained from Gempharmatech Co., Ltd, Jiangsu, China and housed at the Model Animal Research Center, Shandong University. Subsequently, the nude mice were subcutaneously injected with 1 × 10^7^ GC cells, and the measurement of tumor size was conducted every three days using a vernier caliper, commencing on the sixth-day post-injection. The ATRA treatment group (*n* = 4, random allocation) received intraperitoneal injections of the appropriate dosage of ATRA once daily for a period of 12 consecutive days, starting from the 9th day after the initial injection. Conversely, the control group (*n* = 4, random allocation) received daily injections of the same dosage of normal saline. Tumor size was assessed every three days using a vernier caliper. Nude mice were injected with 8×10^6^ GC cells suspended in PBS via the tail vein (*n* = 5, random allocation). After a period of 30 days, the lungs of the nude mice were extracted, and the presence of metastatic lesions was observed and stained with HE. All animal experiments were conducted in adherence to ethical guidelines.

### Statistical analysis

All data were analyzed using Student’s *t* test or one way ANOVA. Data are presented as mean ± SEM, and a *p*-value < 0.05 was considered statistically significant. Statistical analysis was performed using GraphPad Prism 9. In addition to validation of clinical samples and in vivo experiments, three independent replicates were performed.

## Results

### POU5F1 is highly expressed in GC tissues and is associated with poor prognosis

**Fig. 1:**
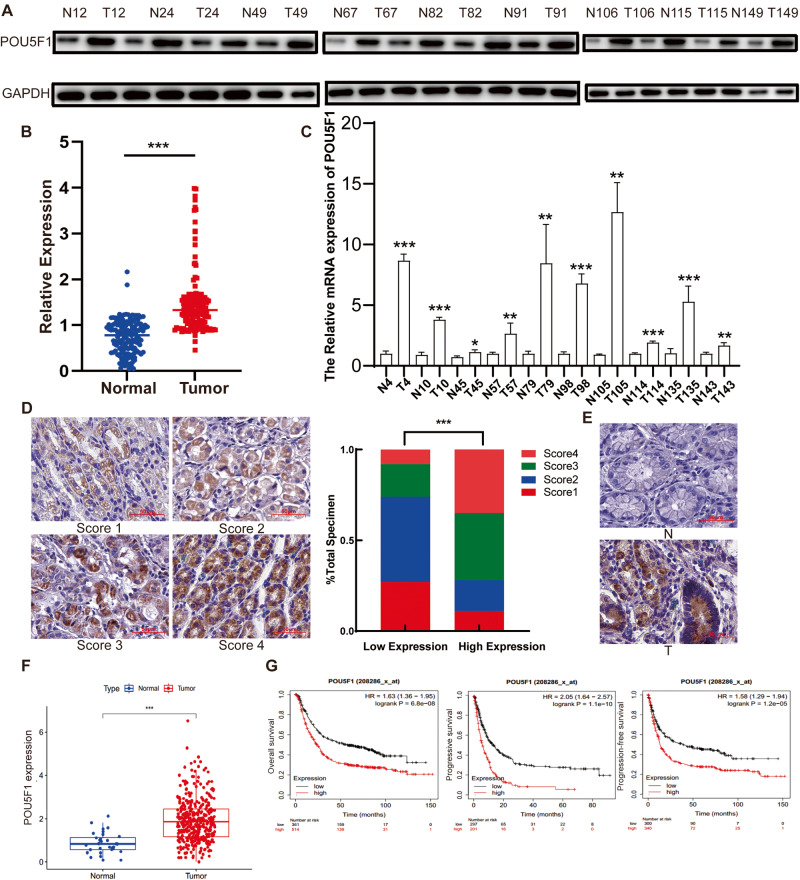
POU5F1 is highly expressed in GC tissues and leads to poor prognosis. **A** Compared with normal tissues, POU5F1 is highly expressed in GC tissues. The statistical quantitative results are shown in (**B**). **C** RT-qPCR results showed that POU5F1 mRNA expression in GC tissues was significantly higher than that in normal tissues. **D** The expression of POU5F1 in GC tissues and adjacent tissues was detected by immunohistochemical staining. **E** We present representative IHC images of normal and gastric cancer tissues. **F** TCGA database analysis showed that POU5F1 was highly expressed in patients with GC. **G** Kaplan–Meier Plotter analysis showed that POU5F1 high expression led to poor prognosis of patients with GC. Values in the figures are presented as mean ± SD. Unpaired *t* test was used to compare the data between two groups, and one-way ANOVA and Tukey’s post hoc test were used to compare the data between multiple groups. *P < 0.05, **P < 0.01, ***P < 0.001. D scale bars = 50 μm. The experiment was repeated three times independently.

Prior research has demonstrated the involvement of POU5F1 in the differentiation and regulation of embryonic stem cells, leading to its predominant expression in embryonic and germ cell tumors [14, 18, 19]. However, recent studies have found that POU5F1 can also be detected in some tissue tumors that are not closely related to primitive cell proliferation, such as bladder cancer [20], lung cancer [21], breast cancer [22], cervical cancer [23], etc. Through analysis of the Cancer Genome Atlas (TCGA), it was determined that POU5F1 exhibits high expression levels in various tumor types, including hepatocellular carcinoma, renal clear cell carcinoma, pancreatic ductal adenocarcinoma, and gastric cancer, thereby contributing to unfavorable prognosis (Supplementary Fig. S1). A total of 150 patients diagnosed with GC were included in this study, from whom GC specimens and corresponding adjacent normal tissue samples were collected. Western blot analysis was performed on selected tissues, with random display and quantification of the results. The findings revealed a significant increase in POU5F1 protein levels in GC tissues (Fig. 1A, B). Furthermore, we conducted an analysis of the mRNA expression level of POU5F1 in tissue samples, revealing a noteworthy elevation in POU5F1 mRNA expression within GC tissues (Fig. 1C). Following this, we employed immunohistochemistry (IHC) to identify the expression of POU5F1 in GC tissues and their corresponding adjacent normal tissues. The IHC score of POU5F1 in GC tissues exhibited a significantly higher value in comparison to that observed in adjacent normal tissues (Fig. 1D). Additionally, we provide representative IHC images depicting the expression of POU5F1 in both normal and GC tissue (Fig. 1E). Furthermore, analysis of the TCGA database revealed a significant up-regulation of POU5F1 expression in GC tissues when compared to normal gastric tissues (Fig. 1F). Kaplan–Meier Plotter analysis showed that POU5F1 expression was significantly negatively correlated with OS, progression-free survival (PFS), and progressive survival (PS) in patients with GC (Fig. 1G).

### POU5F1 promotes the proliferation, migration, and invasion of GC cells, and promotes the EMT process

**Fig. 2:**
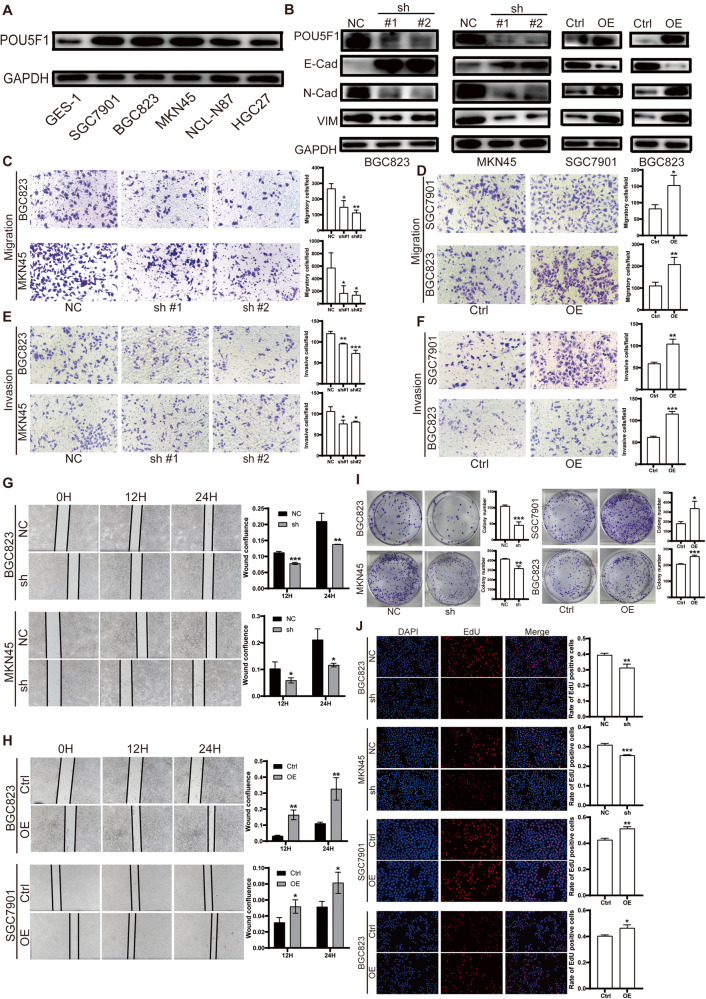
POU5F1 promotes the proliferation, migration and invasion of GC cells. **A** Compared with gastric mucosal epithelial cell GES-1, POU5F1 was highly expressed in GC cells SGC7901, BGC823, MKN45 and Ncl-N87. **B** Representative results of western blot. POU5F1 can promote the expression of VIM and N-Cadherin, and inhibit the expression of E-Cadherin. **C** Silencing POU5F1 attenuated the migration ability of GC cells. **D** Overexpression of POU5F1 enhanced the migration ability of GC cells. **E** Knockdown of POU5F1 attenuated the invasion ability of GC cells. **F** Overexpression of POU5F1 enhanced the invasion ability of GC cells. **G**, **H** Wound healing assay showed that POU5F1 could promote the migration of GC cells. **I** Clonogenic assay showed that POU5F1 could promote the proliferation of GC cells. **J** EdU assay showed that POU5F1 promote the proliferation of GC cells. Values in the figures are presented as mean ± SD. Unpaired *t* test was used to compare the data between two groups. *P < 0.05, **P < 0.01, ***P < 0.001. The experiment was repeated three times independently.

The expression difference of POU5F1 between gastric mucosal epithelial cell GES-1 and GC cell lines BGC823, MKN45, SGC7901, NCL-N87, and HGC27 was compared, revealing a significant increase in POU5F1 expression in GC cell lines (Fig. 2A). We demonstrated the role of POU5F1 in the occurrence and progression of GC by silencing or overexpressing POU5F1 in GC cells. Western blot showed that POU5F1 knockdown inhibited the EMT process of MKN45 and BGC823, while POU5F1 overexpression promoted the expression of mesenchymal markers in SGC7901 and BGC823 (Fig. 2B). The Transwell assay and wound healing assay demonstrated that the knockdown of POU5F1 resulted in a decrease in the migration and invasion capabilities of MKN45 and BGC823 cells, whereas the overexpression of POU5F1 had the opposite effect (Fig. 2C–H). To investigate the impact of POU5F1 on the proliferation of GC cells, clonogenic and EdU assays were conducted. The findings revealed a positive correlation between the expression level of POU5F1 and the proliferation of GC cells (Fig. 2I, J). These findings suggest that POU5F1 can enhance the proliferation, migration, and invasion of GC cells in an in vitro.

### POU5F1 promotes the proliferation and invasion of GC cells in vivo

**Fig. 3:**
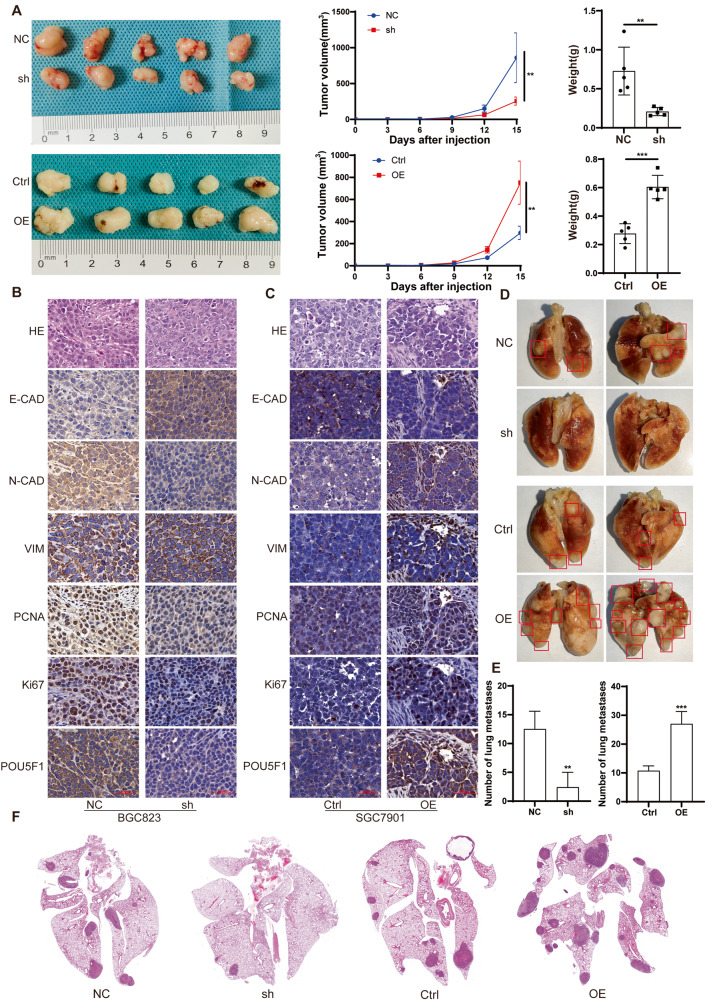
POU5F1 can promote the growth of xenograft tumors and the formation of lung metastases. **A** Nude mice were subcutaneously injected with POU5F1 knockdown GC cells, POU5F1 overexpression GC cells and corresponding control cells. The tumors were removed after 15 days, and the growth rate and weight of the tumors in each group were compared. **B** IHC results showed that POU5F1 knockdown could inhibit the expression of Ki67, PCNA, VIM, N-CAD, and promote the expression of E-CAD in tumors. **C** IHC results showed that POU5F1 overexpression could promote the expression of Ki67, PCNA, VIM, N-CAD, and inhibit the expression of E-CAD in tumors. **D** POU5F1 can promote the formation of lung metastasis of GC cells. The statistical quantitative results are shown in (**E**). **F** Representative HE stained images of lung metastases. Unpaired *t* test was used to compare the data between two groups. *P < 0.05, **P < 0.01, ***P < 0.001. **B**, **C** scale bars = 50 μm. The experiment was repeated three times independently.

To investigate the impact of POU5F1 on the proliferation and invasion of GC cells in mice, we established a xenograft nude mouse model and a nude mouse lung metastasis model. POU5F1 knockdown (POU5F1^KD^) cells, POU5F1 overexpression (POU5F1^OE^) cells, and their respective control cells were prepared and subcutaneously injected into nude mice. After 15 days, the subcutaneous tumors were excised, revealing that POU5F1 knockdown resulted in a decelerated tumor proliferation rate, whereas POU5F1 overexpression promoted tumor proliferation (Fig. 3A). IHC staining revealed a significant decrease in the indicators associated with cell proliferation and EMT in POU5F1^KD^ cells (Fig. 3B). Conversely, these parameters exhibited a significant increase in tumors formed by POU5F1^OE^ cells (Fig. 3C). Protein extraction from tumors in nude mice allowed for the detection of PCNA, VIM, N-Cadherin (N-Cad), and E-Cadherin (E-Cad) protein expressions through western blot analysis. The results demonstrated a positive correlation between the expressions of VIM, N-Cad, and PCNA with the expression of POU5F1, while the expression of E-Cad exhibited a negative correlation with the expression of POU5F1 (Supplementary Fig. S2). In the experimental model of lung metastasis in nude mice, it was observed that there was a significant correlation between the expression of POU5F1 and the number of lung metastases (Fig. 3D–F). These findings indicate that POU5F1 has the potential to impede the proliferation and metastasis of GC tumors in mice.

### POU5F1 can down-regulate the expression level of TRIM59 and promote the expression of TLR4 and TIRAP

**Fig. 4:**
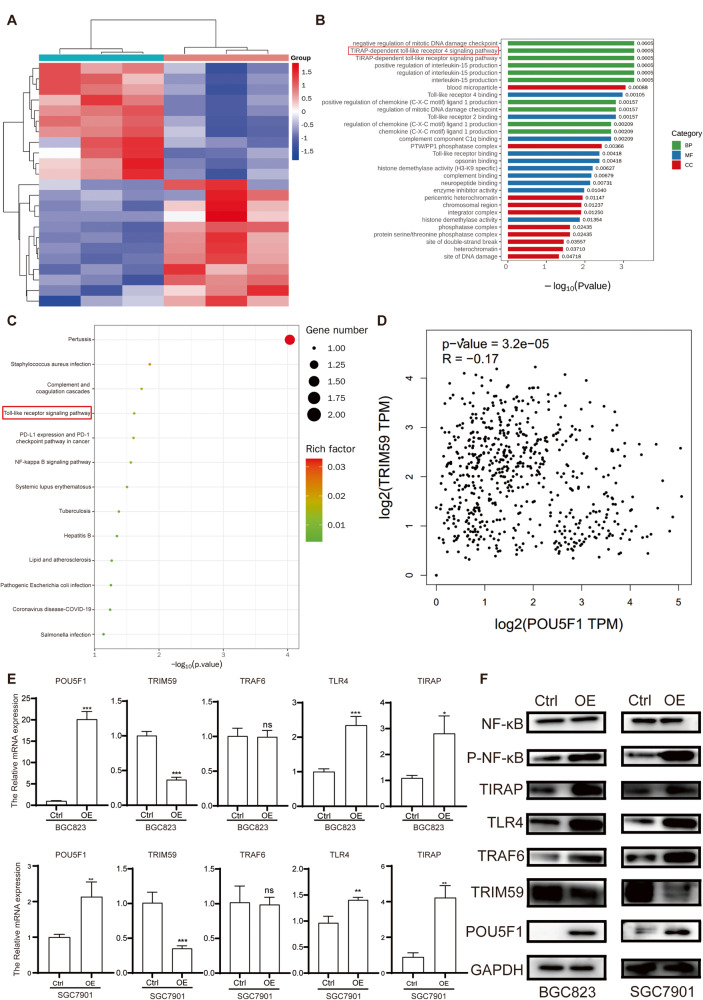
Whole genome RNA sequencing and functional enrichment analysis. **A** Whole genome RNA sequencing suggested that POU5F1 downstream genes TIRAP and TRIM59. **B** KEGG enrichment analysis suggested that POU5F1 downstream genes were related to TIRAP-dependent Toll-like receptor 4 signaling pathway. **C** GO enrichment analysis suggested that POU5F1 downstream genes were related to Toll-like receptor signaling pathway. **D** GEPIA 2 analysis suggested that POU5F1 negatively regulated TRIM59. **E** RT-qPCR was used to verify the sequencing results. **F** Western blot was used to verify the sequencing results. Unpaired *t* test was used to compare the data between two groups. *P < 0.05, **P < 0.01, ***P < 0.001.

To elucidate the mechanism by which POU5F1 modulates the proliferation, migration, and invasion of GC cells, we conducted whole genome RNA sequencing and subsequent functional enrichment analysis. Our sequencing data revealed differential expression of TIRAP and TRIM59 (Fig. 4A). TIRAP, also known as Mal, is a molecule harboring a TIR domain. It specifically interacts with TLR4 and plays a crucial role in the TLR4-mediated MyD88-independent signaling pathway, as well as the activation of the NF-κB signaling pathway [24, 25]. GO enrichment analysis suggested that POU5F1 was related to TIRAP-dependent Toll-like receptor 4 signaling pathway (Fig. 4B). The KEGG pathway enrichment analysis indicated a potential association between POU5F1 and the Toll-like receptor signaling pathway (Fig. 4C). To further investigate this relationship, GEPIA 2 (http://gepia2.cancer-pku.cn/#index) was employed to assess the correlation between POU5F1 and TRIM59 expression, the results showed that the expression of POU5F1 was negatively correlated with the expression of TRIM59 (Fig. 4D). Subsequently, RT-qPCR was conducted to validate the sequencing results, confirming that POU5F1 down-regulated the mRNA level of TRIM59 and enhanced the mRNA expression of TLR4 and TIRAP. However, it was observed that POU5F1 did not regulate TRAF6, a gene involved in the Toll-like receptor 4 signaling pathway (Fig. 4E). It is noteworthy that the western blot analysis revealed that the overexpression of POU5F1 significantly suppressed the expression of TRIM59, while simultaneously increasing the expression of TRAF6 (Fig. 4F). Prior research has demonstrated that TRIM59 functions as an E3 ubiquitin ligase, facilitating the degradation of downstream proteins through ubiquitination [26]. Consequently, we propose the hypothesis that POU5F1 may impede TRIM59-mediated TRAF6 ubiquitination, thereby promoting NF-κB activation and ultimately inducing the malignant phenotype in GC cells.

### TRIM59 regulates TRAF6 expression through the ubiquitin-proteasome pathway

**Fig. 5:**
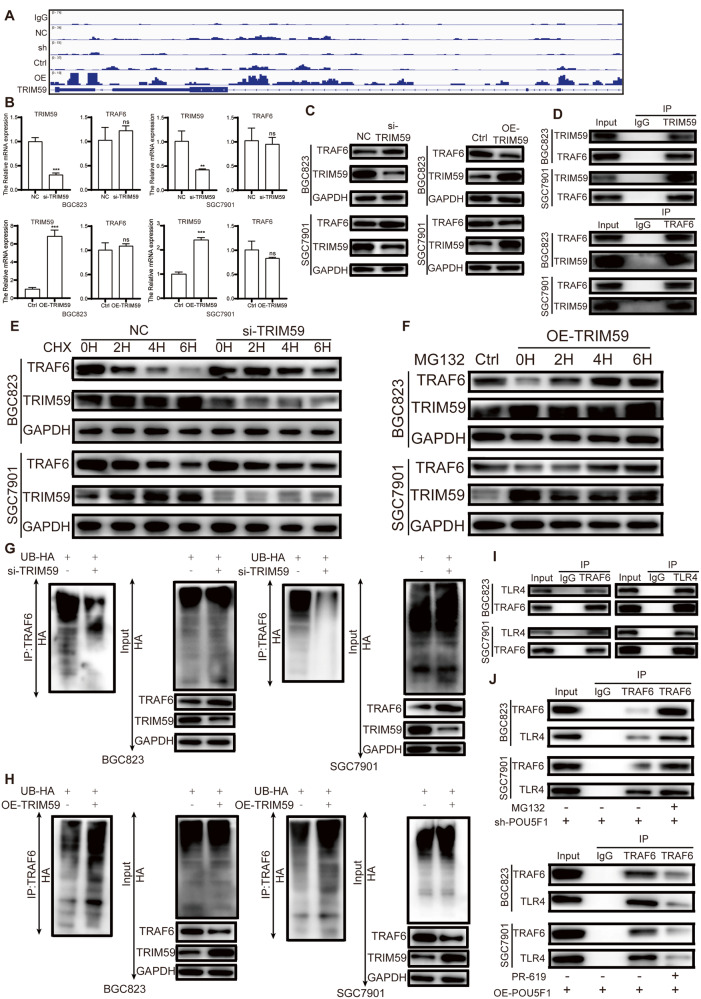
TRIM59 regulates TRAF6 expression through the ubiquitin-proteasome pathway. **A** CUT&TAG results showed that POU5F1 interacted with the promoter of TRIM59. **B** RT-qPCR results showed that knockdown or overexpression of TRIM59 had no significant effect on the mRNA expression of TRAF6. **C** Western blot results showed that TRIM59 inhibited the protein expression of TRAF6. **D** IP assay showed that there was a protein interaction between TRIM59 and TRAF6. **E** TRIM59 knockdown upregulated TRAF6 expression. After adding CHX, the degradation rate of TRAF6 in TRIM59 knockdown group was slower than that in control group. **F** TRIM59 overexpression reduced TRAF6 expression, and MG132 stimulation increased TRAF6 expression. **G** TRIM59 knockdown reduced the ubiquitination level of TRAF6. **H** Overexpression of TRIM59 increased the ubiquitination level of TRAF6. **I** IP assay showed that TRAF6 interacted with TLR4. **J** MG132 could reverse the decrease in the expression of TRAF6 and TLR4 caused by POU5F1 knockdown. PR-619 could reverse the increased expression of TRAF6 and TLR4 caused by POU5F1 overexpression. Unpaired *t* test was used to compare the data between two groups. *P < 0.05, **P < 0.01, ***P < 0.001. The experiment was repeated three times independently.

First, we verified the interaction between POU5F1 and TRIM59. Based on the CUT&TAG experiment, further observation using the Integrative Genomics Viewer (IGV) revealed an interaction between POU5F1 and the promoter of TRIM59 (Fig. 5A). In order to examine the association between TRIM59 and TRAF6, we employed techniques of gene silencing and overexpression of TRIM59 in BGC823 and SGC7901 cell lines, followed by assessment of TRAF6 mRNA and protein expression levels. Our findings from RT-qPCR analysis indicated that altering the expression of TRIM59 did not result in significant changes in TRAF6 mRNA expression in GC cells (Fig. 5B). However, a negative correlation was observed between the expression levels of TRIM59 and TRAF6 protein (Fig. 5C). Following the transfection of flag-TRIM59 plasmid into BGC823 and SGC7901, co-immunoprecipitation of cell lysates using TRAF6 antibody or normal rabbit IgG demonstrated the binding of TRIM59 to TRAF6 (Fig. 5D). In order to investigate whether TRIM59 negatively regulates TRAF6 by promoting its degradation, we introduced 10 mM CHX to si-TRIM59 transfected GC cells. The results showed that TRAF6 interpretation was slowed down in TRIM59 knockdown group compared with NC transfection group after CHX stimulation (Fig. 5E). In order to further validate the degradation of TRAF6 by TRIM59 via the ubiquitin-proteasome pathway, BGC823 and SGC7901 cell lines were transfected with a plasmid overexpressing TRIM59 and subsequently stimulated with the addition of 10 mM MG132, followed by incubation at 37 °C. Western blot results showed that overexpression of TRIM59 reduced the expression level of TRAF6, and MG132 stimulation could increase the expression level of TRAF6 compared with the control group, indicating that TRIM59 caused the degradation of TRAF6 through the ubiquitin-protease pathway (Fig. 5F). Next, we examined the effect of TRIM59 expression levels on TRAF6 ubiquitination levels. Knockdown of TRIM59 in GC cells decreased the ubiquitination level of TRAF6 (Fig. 5G), while overexpression of TRIM59 increased the ubiquitination level of TRAF6 (Fig. 5H). Our results suggest that TRIM59 mediates TRAF6 degradation through ubiquitination. Co-immunoprecipitation of cell lysates with TRAF6 antibody or TLR4 antibody, using IgG as a control, showed that TRAF6 was protein-bound to TLR4 (Fig. 5I). GC cells with POU5F1 knockdown were subjected to treatment with the proteasome inhibitor MG132. Following a 6-h stimulation, co-immunoprecipitation using a TRAF6 antibody revealed that the proteasome inhibitor was capable of reversing the ubiquitination of TRAF6, which had been induced by the upregulation of TRIM59 expression resulting from the downregulation of POU5F1 expression. Additionally, the deubiquitinating enzyme inhibitor PR-619 was able to reverse the decrease in TRAF6 ubiquitination caused by the increased expression of POU5F1 and decreased expression of TRIM59 (Fig. 5J).

### The impact of TRIM59, TRAF6 and TLR4 on the proliferation, migration, and invasion of GC cells

**Fig. 6:**
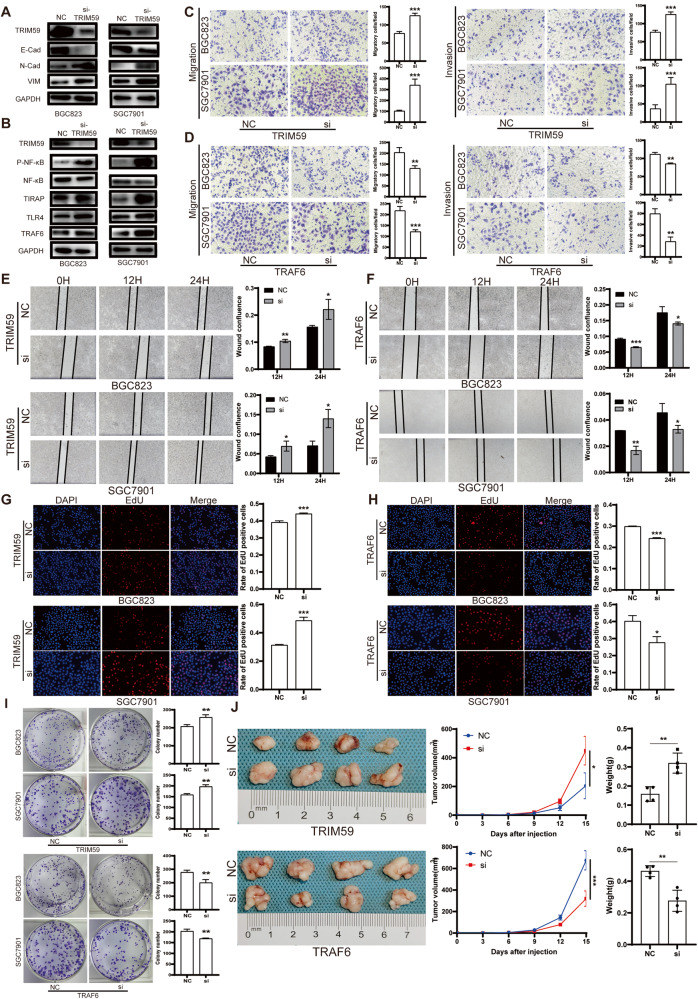
Effects of TRIM59 and TRAF6 on the proliferation, migration and invasion of GC cells. **A** TRIM59 knockdown promoted the EMT process of GC cells. **B** TRIM59 knockdown promotes the activation of NF-κB pathway in GC cells. **C** TRIM59 knockdown promoted the migration and invasion of GC cells. **D** Knockdown of TRAF6 inhibited the migration and invasion of GC cells. **E** Wound healing assay showed that TRIM59 knockdown promoted GC cell invasion. **F** Wound healing assay showed that knockdown of TRAF6 inhibited the invasion of GC cells. **G** EdU assay showed that TRIM59 knockdown promoted the proliferation of GC cells. **H** EdU assay showed that knockdown of TRAF6 inhibited the proliferation of GC cells. **I** Clonogenic assay showed that knockdown of TRIM59 promoted the proliferation of GC cells, while knockdown of TRAF6 inhibited the proliferation of GC cells. **J** TRIM59 knockdown promoted the proliferation of GC cells in nude mice, while TRAF6 knockdown inhibited the proliferation of GC cells in nude mice. Unpaired *t* test was used to compare the data between two groups. *P < 0.05, **P < 0.01, ***P < 0.001. The experiment was repeated three times independently.

To investigate the impact of TRIM59 on the epithelial-mesenchymal transition (EMT) process in GC cells, BGC823 and SGC7901 cells were transfected with si-TRIM59, and the expression levels of EMT-related markers were assessed using western blot analysis. The findings demonstrated that the downregulation of TRIM59 significantly enhanced the EMT process (Fig. 6A). Additionally, the examination of TLR4/TRAF6/NF-κB signaling molecules revealed that TRIM59 knockdown facilitated the activation of the NF-κB pathway (Fig. 6B). The migration and invasion of BGC823 and SGC7901 were assessed using the Transwell assay and wound healing assay to investigate the impact of TRIM59 and TRAF6. Knockdown of TRIM59 was found to enhance the migration and invasion ability of GC cells (Fig. 6C, E), whereas knockdown of TRAF6 was observed to inhibit the migration and invasion ability of GC cells (Fig. 6D, F). The influence of TRIM59 and TRAF6 on the proliferation of BGC823 and SGC7901 was evaluated through the EdU assay and clonogenic assay. The findings indicated that TRIM59 suppressed the proliferation of GC cells, while TRAF6 promoted their proliferation (Fig. 6G–I). These results demonstrated that TRIM59 inhibited the proliferation, migration and invasion of GC cells in vitro, while TRAF6 promoted these features of GC cells. Furthermore, these results were validated in vivo. The proliferation of BGC823 transfected with si-TRIM59 was significantly accelerated in nude mice, and the proliferation of BGC823 transfected with si-TRAF6 was inhibited (Fig. 6J). Protein extraction was performed on the tumor samples obtained from nude mice, followed by western blot analysis to determine the protein expression levels of PCNA and the EMT process. The obtained results indicated a negative correlation between the protein expression of VIM, N-Cad, and PCNA with the expression of TRIM59, while a positive correlation was observed between the expression of E-Cad and TRIM59. Additionally, the overexpression of TRAF6 was found to promote tumor EMT and enhance the expression of PCNA (Supplementary Fig. S3). In addition, we knocked down TLR4 expression in GC cells. Results showed that knockdown of TLR4 significantly inhibited the proliferation, migration and invasion of GC cells in vitro. In the in vivo experiment, knockdown of TLR4 significantly inhibited tumor growth in nude mice. The expression of PCNA, N-Cad and VIM decreased, while the expression of E-Cad increased in tumors (Supplementary Fig. S4).

### TRIM59 is down-regulated in GC tissues and has a positive effect on the prognosis of GC patients

TRIM59 exhibits down-regulation in GC tissues and exerts a favorable impact on the prognosis of GC patients. We conducted an assessment of TRIM59 expression at both the protein and mRNA levels in GC tissues and their corresponding adjacent normal tissues. Our findings revealed a significant reduction in TRIM59 expression within GC tissues compared to normal tissues (Supplementary Fig. S5A–C). To further investigate the association between TRIM59 expression and GC patient prognosis, we employed the Kaplan-Meier Plotter. The outcomes demonstrated that elevated TRIM59 expression correlated positively with improved prognosis among GC patients (Supplementary Fig. S5D).

### All-trans retinoic acid (ATRA) can inhibit the proliferation, migration and invasion of GC cells

**Fig. 7:**
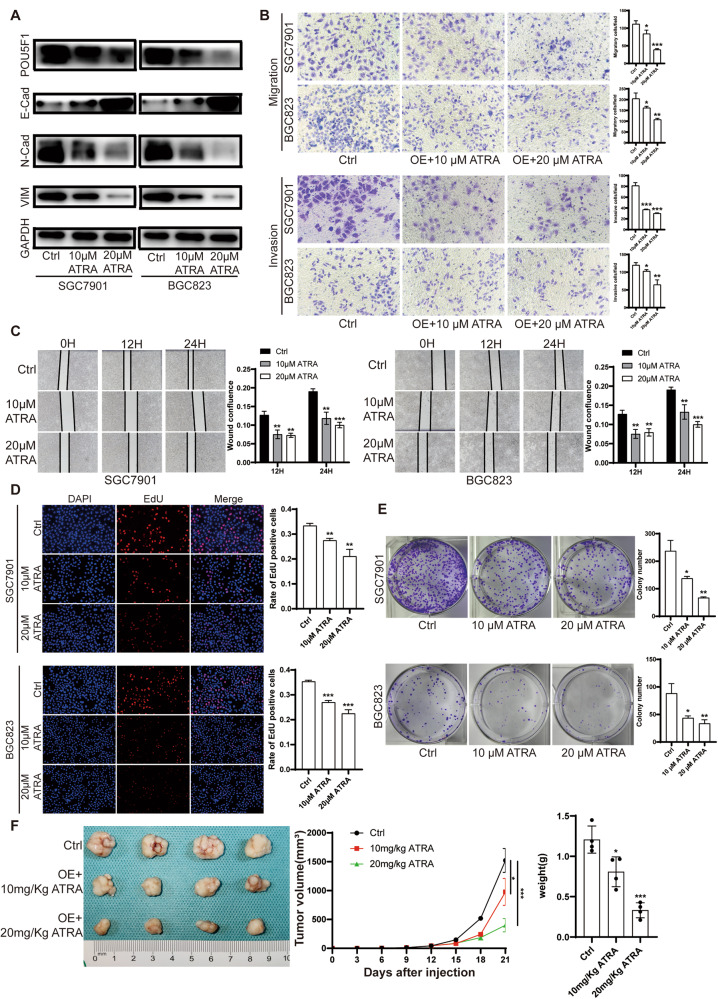
ATRA inhibits the expression of POU5F1 in GC cells. **A** POU5F1 expression was significantly inhibited by 10 μM and 20 μM ATRA in GC cells for 48 h, and the EMT process was also significantly inhibited. **B** Transwell assay showed that ATRA significantly inhibited the migration and invasion of GC cells. **C** Wound healing assay showed that ATRA significantly inhibited the migration ability of GC cells. **D**, **E** EdU assay and clonogenic assay showed that ATRA significantly inhibited the proliferation of GC cells. **F** Xenografts in nude mice showed that ATRA inhibited the growth of GC cells in nude mice, which was consistent with the results in vitro. Unpaired *t* test was used to compare the data between two groups. *P < 0.05, **P < 0.01, ***P < 0.001. The experiment was repeated three times independently.

All-trans retinoic acid (ATRA) is a metabolic intermediary of vitamin A in animals and assumes a significant function in cellular proliferation, differentiation, and apoptosis in vertebrates [27]. In clinical practice, ATRA is the primary therapeutic option for managing acute promyelocytic leukemia (APL) [28]. Previous research has demonstrated the potential of ATRA to exhibit anti-tumor effects in various cancers, such as Kaposi’s sarcoma, head and neck squamous cell carcinoma, ovarian cancer, and other solid tumors, when tested in vitro [29]. However, the extent of its anti-tumor activity in GC remains relatively unexplored. It has been reported that ATRA is able to inhibit POU5F1 expression through retinoic acid response elements located in the promoter-enhancer region [30]. Additionally, other investigations have revealed that ATRA can impede the expression of POU5F1 in bladder cancer cells, thereby rendering these cells less susceptible to destruction induced by oncolytic adenovirus carrying POU5F1 response element (ORE) [31]. In this study, we conducted an investigation to examine the impact of all-trans retinoic acid (ATRA) on the proliferation, migration, and invasion of BGC823 and SGC7901 cells. Various concentrations of ATRA were utilized to stimulate GC cells. Our findings from western blot analysis demonstrated a significant inhibition of the EMT process in both BGC823 and SGC7901 cells upon treatment with ATRA (Fig. 7A). Furthermore, the Transwell assay and wound healing assay revealed a significant reduction in the migration and invasion capabilities of GC cells following ATRA treatment (Fig. 7B, C). Additionally, the EdU assay and colony formation assay provided evidence of a significant decrease in the proliferation of BGC823 and SGC7901 cells upon exposure to ATRA (Fig. 7D, E). These results confirmed that ATRA could inhibit the proliferation, migration and invasion of GC cells in vitro. The results of xenograft in nude mice demonstrated that ATRA could inhibit tumor proliferation in nude mice (Fig. 7F).

**Fig. 8:**
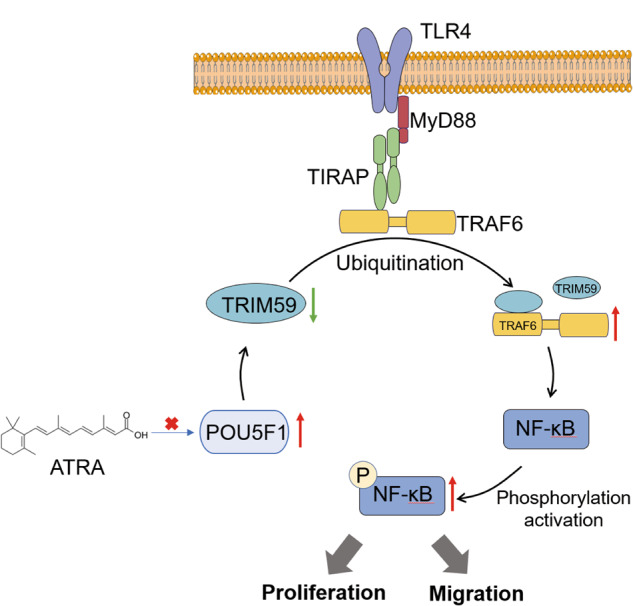
The role of POU5F1, TRIM59, and TLR4/TRAF6/NF-κB pathway in the progression of GC. POU5F1 can down-regulate the expression of TRIM59, reduce the ubiquitination level of TRAF6, inhibit the ubiquitination degradation of TRAF6, promote the formation of TLR4/TIRAP/TRAF6 complex, promote the activation of NF-κB pathway, and finally promote the proliferation, migration, and invasion of GC cells. ATRA can inhibit the expression of POU5F1, which is expected to become an adjuvant drug for the treatment of GC.

Based on our study, we propose a model for the role of ATRA, POU5F1, TRIM59, and TRAF6 in GC (Fig. 8). POU5F1 can down-regulate the expression of TRIM59, reduce the ubiquitination level of TRAF6, inhibit the ubiquitination degradation of TRAF6, promote the formation of TLR4/TIRAP/TRAF6 complex, promote the activation of NF-κB pathway, and finally promote the proliferation, migration and invasion of GC cells. ATRA can inhibit the expression of POU5F1, which is expected to become an adjuvant drug for the treatment of GC.

## Discussion

Gastric cancer, a disease with multifactorial etiology and high mortality rates, exhibits genetic susceptibility [32]. Despite continuous advancements in clinical surgical techniques, a significant proportion of patients experience postoperative recurrence of GC, resulting in unfavorable prognoses [33]. Consequently, there is an urgent need to investigate the molecular physiological mechanisms underlying the onset and progression of GC, identify novel diagnostic molecular markers, and discover new therapeutic targets and targeted drugs.

POU homeobox gene family transcription factor POU5F1 is an important regulator to maintain cell totipotency [34], It has been reported to be expressed in a variety of adult stem cells, including pancreatic stem cells, mesenchymal stem cells, and gastric stem cells [35]. It has been reported that POU5F1 maintains the CSC-like properties of lung cancer cells and is associated with poor prognosis in lung adenocarcinoma [8, 36]. Therefore, we investigated the effect of POU5F1 on the occurrence and progression of GC, and established the POU5F1/TRIM59/NF-κB/EMT regulatory axis in GC cells. POU5F1 enhances the EMT mechanism in GC cells by facilitating the activation of the NF-κB pathway. Consequently, POU5F1 holds potential as a prospective diagnostic indicator and therapeutic target for GC. Moreover, POU5F1 exerts influence on the expression of downstream genes and proteins via diverse epigenetic modifications, including phosphorylation, acetylation, and methylation [37–39]. The TRIM (Tripartitemotif) protein family is a protein family containing RBCC structure, exhibits high conservation and functions as an E3 ubiquitin ligase, facilitating protein ligation and mediating ubiquitination [40]. This investigation reveals that POU5F1 can effectively decrease the ubiquitination extent of TRAF6 through the down-regulation of TRIM59, thereby facilitating the activation of the NF-κB signaling pathway.

In the present study, it was observed that POU5F1 exhibited significantly elevated expression levels in GC tissues as compared to normal tissues. This finding was further corroborated by data obtained from public databases. Moreover, our investigation revealed that POU5F1 exerted a promotional effect on the proliferation, migration, and invasion capacities of GC cells. Additionally, in vivo experiments demonstrated that POU5F1 also facilitated the proliferation and invasion of GC cells.

Whole genome RNA sequencing and functional enrichment analysis successfully identified TRIM59, a gene downstream of POU5F1, as well as the TLR4/TRAF6/NF-κB signaling pathway. TRIM59, a constituent of the TRIM protein family, has been implicated in the initiation and advancement of diverse tumor types. Notably, TRIM59 has been observed to activate the Ras and Rb signaling pathways, thereby facilitating the progression of prostate cancer [41]. Furthermore, investigations have demonstrated that TRIM59 can foster the onset and progression of GC through its involvement in the ubiquitination and degradation of p53 [42]. In esophageal cancer, the inhibition of TRIM59 has been found to induce an up-regulation of P53, thereby augmenting the chemosensitivity of esophageal cancer cells towards cisplatin [43]. Nevertheless, our investigation revealed that the down-regulation of TRIM59 expression by POU5F1 resulted in contrasting outcomes. The findings from Kaplan-Meier Plotter analysis indicated that a high expression level of TRIM59 had a favorable impact on the prognosis of patients with GC.

Toll-like receptors (TLRs) are a group of transmembrane proteins that possess the ability to engage downstream proteins of identical domain for the purpose of facilitating signal transduction, such as Myeloid differentiation factor-88 (MyD88). Upon TLR binding to MyD88, a series of Interleukin-receptor-associated kinase (IRAK) family proteins [44, 45] can be recruited. Following stimulation, phosphorylation of IRAK1 and IRAK4 occurs, leading to their dissociation from MyD88 and subsequent activation of the downstream TRAF6. TRAF6 can activate TGFβ-activated kinase (TAK1) through K63 polyubiquitin chain, and TAK1 complex activates IKK complex, leading to the phosphorylation of NF-κB inhibitor IκB, which ultimately leads to the activation of NF-κB [46]. TRAF6 is an important member of the TNF receptor-associated factors (TRAFs). As an important adaptor protein, TRAF6 plays an important role in immunity by participating in the regulation of NF-κB, MAPK/PI3K and interferon regulatory factor (IRF) signal pathways [47, 48]. Multiple studies have demonstrated that TRAF6 exhibits abnormal overexpression in various malignant tumors, including lung cancer [49], colon cancer [50], and melanoma [51], and plays a crucial role in regulating tumor cell proliferation, migration, and invasion [52]. In our investigation, we have discovered that TRIM59 is capable of facilitating the ubiquitination process of TRAF6, while POU5F1 negatively modulates the expression of TRIM59, consequently reducing the ubiquitination level of TRAF6. This intricate mechanism ultimately promotes the activation of the NF-κB pathway, thereby significantly enhancing the proliferation, migration, and invasion of GC cells.

All-trans retinoic acid (ATRA) is converted from vitamin A in the body through A series of REDOX processes such as retinol ester hydrolase, retinol dehydrogenase, and retinaldehyde dehydrogenase [53]. ATRA acts as a differentiation inducer, impeding tumor cell proliferation and promoting their differentiation towards a normal phenotype, thereby restoring normal cellular function and ultimately leading to tumor remission. The utilization of ATRA in the management of acute promyelocytic leukemia (APL) has yielded remarkable therapeutic outcomes [54]. ATRA has been shown to exert a potent role in stem cell differentiation through several retinol response elements, including POU5F1, in the promoter-enhancer regions of its target genes. Consequently, we investigated the potential therapeutic impact of ATRA on GC by modulating the expression of POU5F1 in GC cells. The findings revealed a dose-dependent inhibition of POU5F1 expression in GC cells by ATRA, accompanied by a decrease in proliferation, migration, and invasion of GC cells. Furthermore, ATRA exhibited the ability to impede tumor growth in nude mice. Nevertheless, the clinical application of ATRA is not without its challenges, including adverse effects such as cutaneous and mucosal dryness, skeletal muscle pain, and hepatic impairment. Enhancing the therapeutic efficacy and mitigating the adverse effects of drugs have emerged as crucial concerns in the utilization of ATRA for cancer treatment. In forthcoming research endeavors, two potential strategies can be explored. Firstly, modifying the molecular structure of ATRA and synthesizing derivatives with heightened efficacy and diminished side effects. Secondly, combining ATRA with established chemotherapy drugs to minimize drug dosage and augment therapeutic outcomes.

## Conclusions

In this study, it was observed that POU5F1 exerts a down-regulatory effect on TRIM59, thereby leading to a decrease in the ubiquitination level of TRAF6. Consequently, the activation of the NF-κB signaling pathway is enhanced, ultimately facilitating the proliferation, migration, and invasion of GC cells. Additionally, the administration of ATRA was found to suppress the expression of POU5F1 in GC cells, thereby inhibiting the malignant characteristics of GC cells. These findings suggest that ATRA holds promise as a potential therapeutic agent for the treatment of GC.

### Supplementary information

supplementary materials and legends

Extended Data 1

Extended Data 2

## Data Availability

All data generated or analyzed during this study are included in this published article and its supplementary information files.
