# Supplementary material for: POU5F1 promotes the proliferation, migration, and invasion of gastric cancer cells by reducing the ubiquitination level of TRAF6
Source: Cell Death Dis. 2023 Dec 7;14(12):802. doi: 10.1038/s41419-023-06332-8 (PMC10703809; doi:10.1038/s41419-023-06332-8)
Supplement: Supplementary file 1 — supplementary materials and legends [file 41419_2023_6332_MOESM1_ESM.pdf]

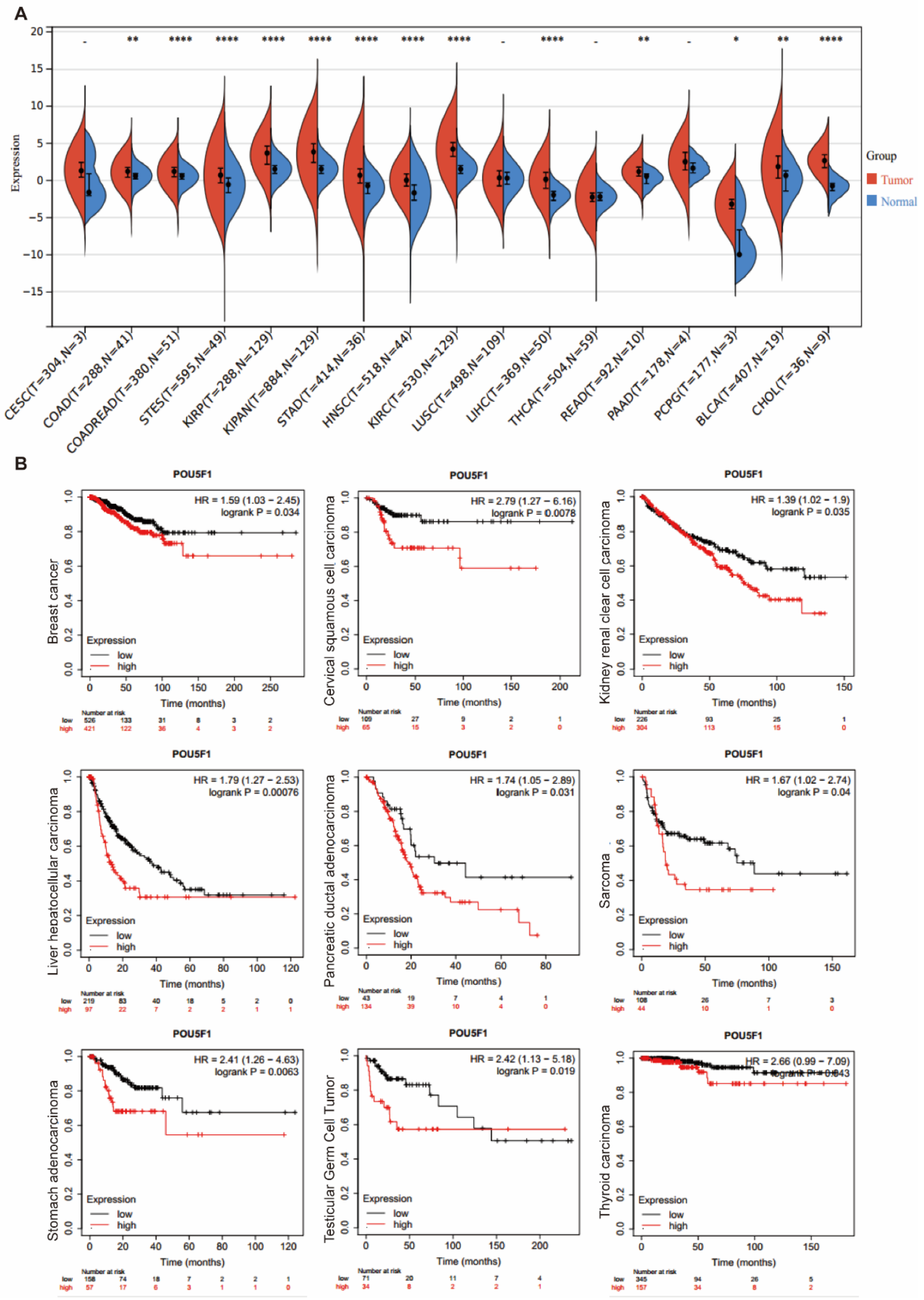

Supplementary Fig. S1 Expression of POU5F1 in pan-cancer and its effect on the prognosis of pan-cancer. **(A)** The expression of POU5F1 in pan-cancer was analyzed using TCGA database, and the results showed that, POU5F1 was detected in Colon adenocarcinoma (COAD), Kidney renal

papillary cell carcinoma (KIRP), Stomach adenocarcinoma (STAD), and Liver The expression was significantly increased in hepatocellular carcinoma (LIHC), Bladder Urothelial Carcinoma (BLCA), Cholangio carcinoma (CHOL) and other cancers. **(B)** Kaplan-Meier Plotter was used to analyze the effect of POU5F1 on the prognosis of pan-cancer, and the analysis showed that POU5F1 had a significant negative effect on the prognosis of KIRP, STAD, LIHC and other tumors.

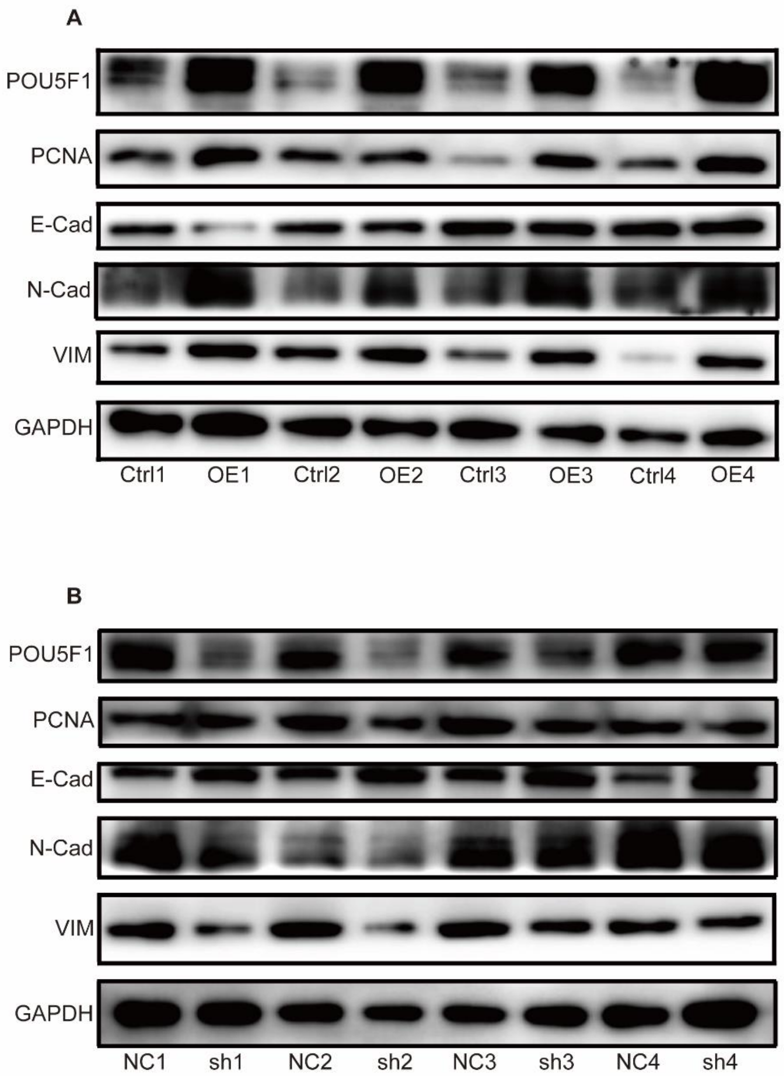

Supplementary Fig. S2 Effects of POU5F1 on EMT and proliferation-related protein expression in nude mice xenograft tumors. **(A)** The expression of VIM, N-Cad and PCNA was increased and the expression of E-Cad was decreased in the tumor formed by GC cells overexpressing POU5F1. **(B)** In POU5F1 knockdown GC cells, the expression of VIM, N-Cad and PCNA decreased, and the expression of E-Cad increased. These results are consistent with those obtained *in vitro*.

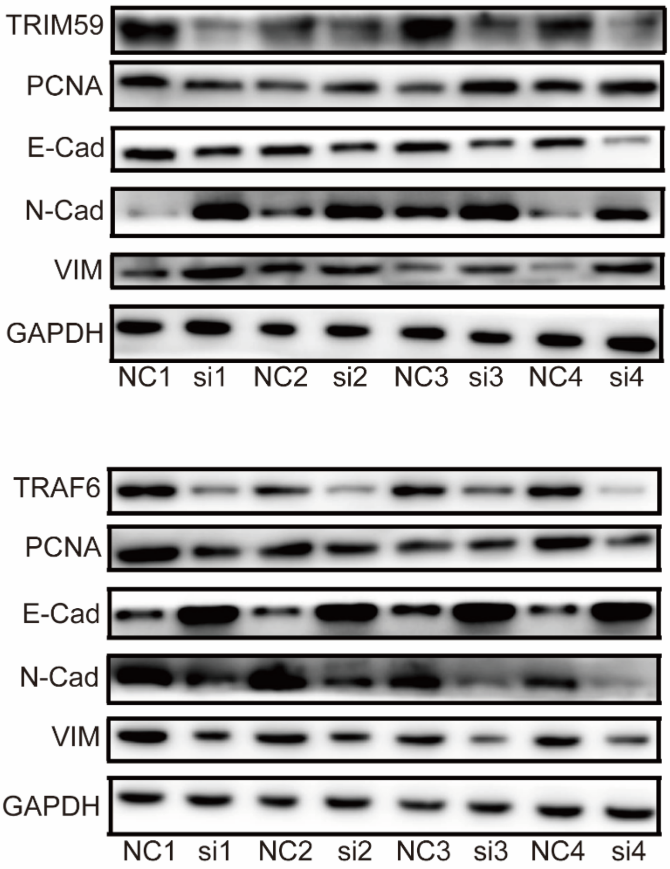

Supplementary Fig. S3 Effect of TRIM59 and TRAF6 knockdown on protein expression in xenograft in nude mice. **(A)** Knockdown TRIM59 increased the expression of VIM, N-Cad and PCNA, and decreased the expression of E-Cad in xenograft tumor in nude mice. **(B)** TRAF6 knockdown decreased the expression of VIM, N-Cad and PCNA in xenograft tumor in nude mice, and promoted the expression of E-Cad. These results are consistent with those of *in vitro* experiments.

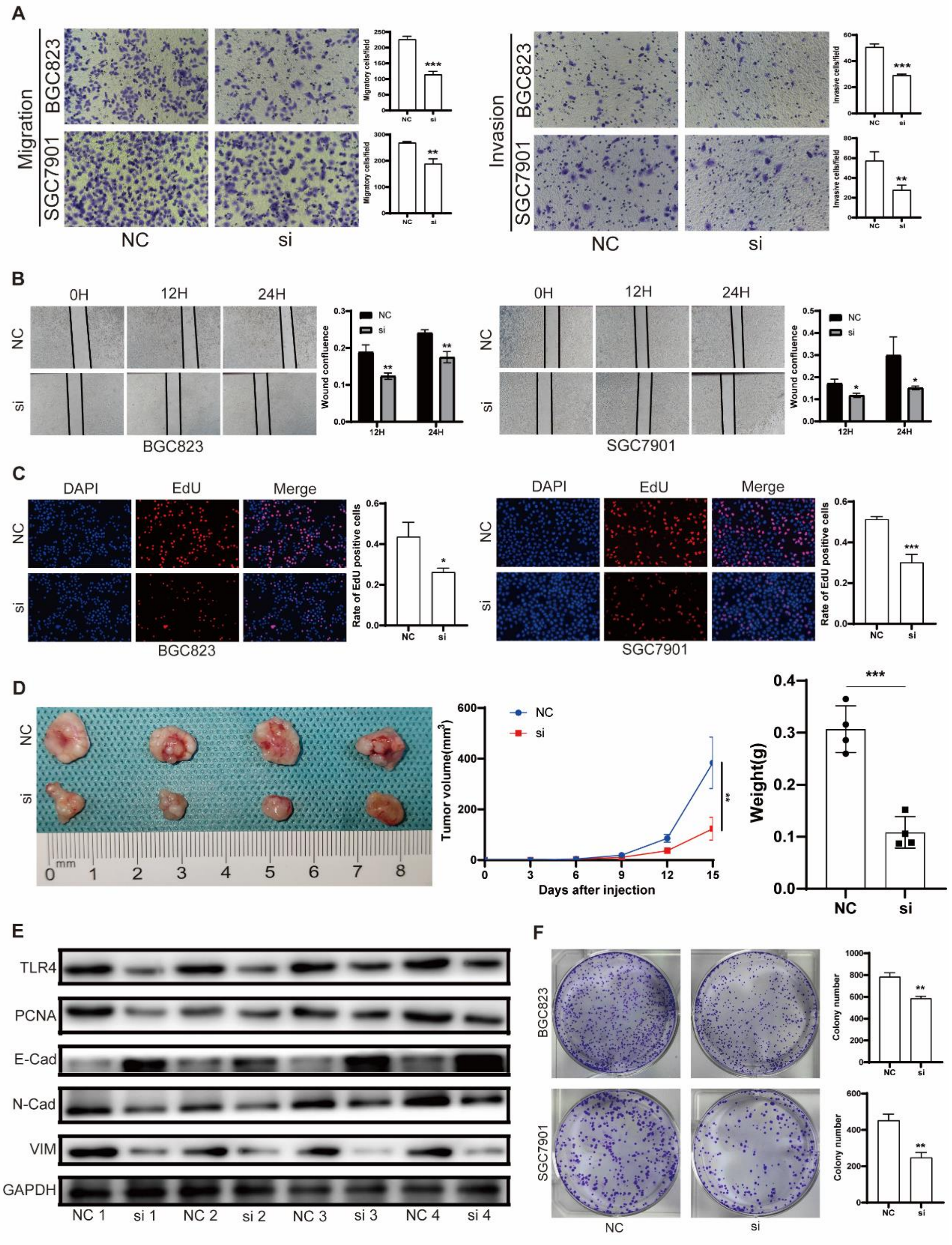

Supplementary Fig. S4 Effect of TLR4 on proliferation, migration and invasion of GC cells. **(A)** Knockdown of TLR4 reduced the migration and invasion ability of GC cells. **(B)** Wound healing assay showed that knockdown of TLR4 attenuated the migration ability of GC cells. **(C)** The results

of EdU assay showed that TLR4 knockdown attenuated the proliferation of GC cells. **(D)** Knockdown of TLR4 slowed the growth of xenografts in nude mice. **(E)** The results of western blot showed that the expression of VIM, N-Cad and PCNA decreased, and the expression of E-Cad increased in the xenografts of nude mice after TLR4 knockdown. **(F)** Clonogenic assay showed that knockdown of TLR4 significantly inhibited the proliferation of GC cells. Unpaired t test was used to compare the data between two groups. $^*P < 0.05$, $^{**}P < 0.01$, $^{***}P < 0.001$. The experiment was repeated three times independently.

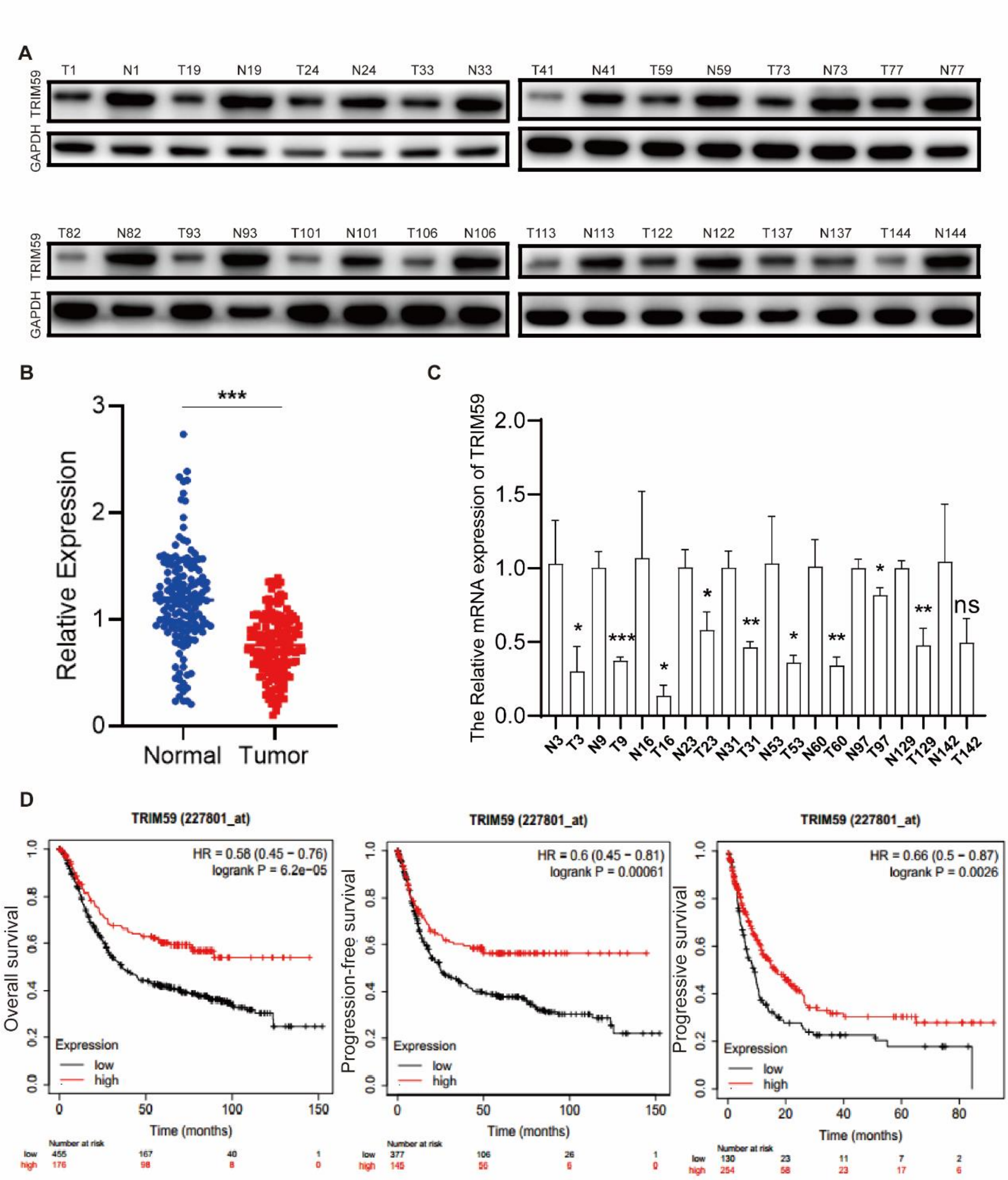

Supplementary Fig. S5 TRIM59 is down-regulated in GC tissues and improves the prognosis of GC patients. **(A)** POU5F1 was down-regulated in GC tissues compared with normal tissues. Statistical quantitative results are shown in **(B)**. **(C)** RT-qPCR results showed that the expression of

TRIM59 mRNA was relatively low in GC tissues. **(D)** Kaplan-Meier Plotter analysis showed that high expression of TRIM59 improved the prognosis of GC patients. Unpaired t test was used to compare the data between two groups. *P < 0.05, **P < 0.01, ***P < 0.001. The experiment was repeated three times independently.

Supplementary Table 1 Primer sequences for RT-qPCR.

Name	Sequence
β-actin forward	5’-GAAGAGCTACGAGCTGCCTGA-3’
β-actin reverse	5’-CAGACAGCACTGTGTTGGCG-3’
POU5F1 forward	5’-TCAGGAGATATGCAAAGCAGAA-3’
POU5F1 reverse	5’-TTGCCTCTCACTCGGTTCTC-3’
TRIM59 forward	5’-AAGATCCTCGTGTACCTGCCAT-3’
TRIM59 reverse	5’-CAATGCCAGTTGGAGCAATTTC-3’
TRAF6 forward	5’-CTATTCACCAGTTAGAGG-3’
TRAF6 reverse	5’-GCTCACTTACATACATACT-3’
TLR4 forward	5’-GATAGCGAGCCACGCATTCA-3’
TLR4 reverse	5’-TTAGGAACCACCTCCACGCA-3’
TIRAP forward	5’-AGTGTCCGCCATCAGGGA-3’
TIRAP reverse	5’-CATTTGTGGGAATCCGAGGC-3’

Supplementary Table 2 Sequences for si-RNA and plasmids.

Name	Sequence
si-TRIM59	5’- GGGAAACAGAATCTAGTAACA-3’
si-TRAF6	5’-TGGATTCTACACTGGCAAA-3’
si-TLR4	5’-GGGCUUAGAACAACUAGAATT-3’
POU5F1-i1F	gatccGGGAAGGTATTCAGCCAAAtcaagagTTTGGCTGAATACCTTCCCtttttt
POU5F1-i1R	aattaaaaaaGGGAAGGTATTCAGCCAAActcttgaTTTGGCTGAATACCTTCCCg
POU5F1-i2F	gatccGGAGGAAGCTGACAACAATtcaagagATTGTTGTCAGCTTCCTCCtttttt
POU5F1-i2R	aattaaaaaaGGAGGAAGCTGACAACAATctcttgaATTGTTGTCAGCTTCCTCCg

Supplementary Table 3 The relationship between the POU5F1 expression in GC tissues and clinicopathologic features.

**Indicators**	**N=150**	**POU5F1 expression**	**p value**
Age			
≥ 60	69	1.464±0.711	0.857
< 60	81	1.484±0.653	
Gender			
Male	77	1.344±0.519	0.179
Female	73	1.395±0.531	
Tumor size (cm)			
≥ 5	61	1.564±0.903	0.415
< 5	89	1.493±0.487	
Pathological stage			
I+II	63	1.354±0.454	0.0056
III+IV	87	1.657±0.789	
Diferentiation degree			
Low	57	2.069±0.869	<0.001
Medium–high	93	1.276±0.259	
