# Supplementary material for: POU5F1 promotes the proliferation, migration, and invasion of gastric cancer cells by reducing the ubiquitination level of TRAF6
Source: Cell Death Dis. 2023 Dec 7;14(12):802. doi: 10.1038/s41419-023-06332-8 (PMC10703809; doi:10.1038/s41419-023-06332-8)
Supplement: Supplementary file 3 — Extended Data 2 [file 41419_2023_6332_MOESM3_ESM.docx]

Figure 1

55kDa

POU5F1
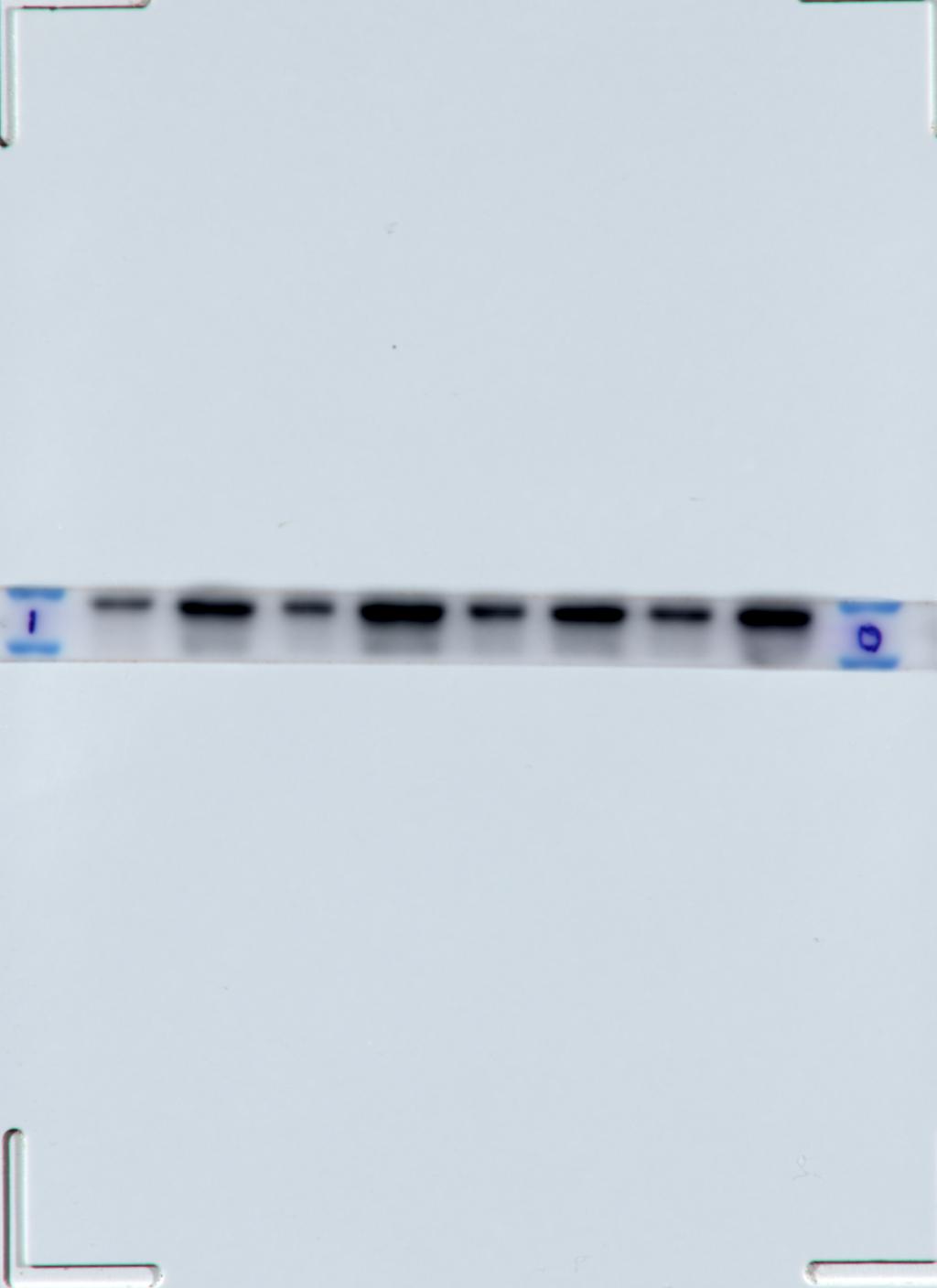
 40kDa

40kDa

GAPDH
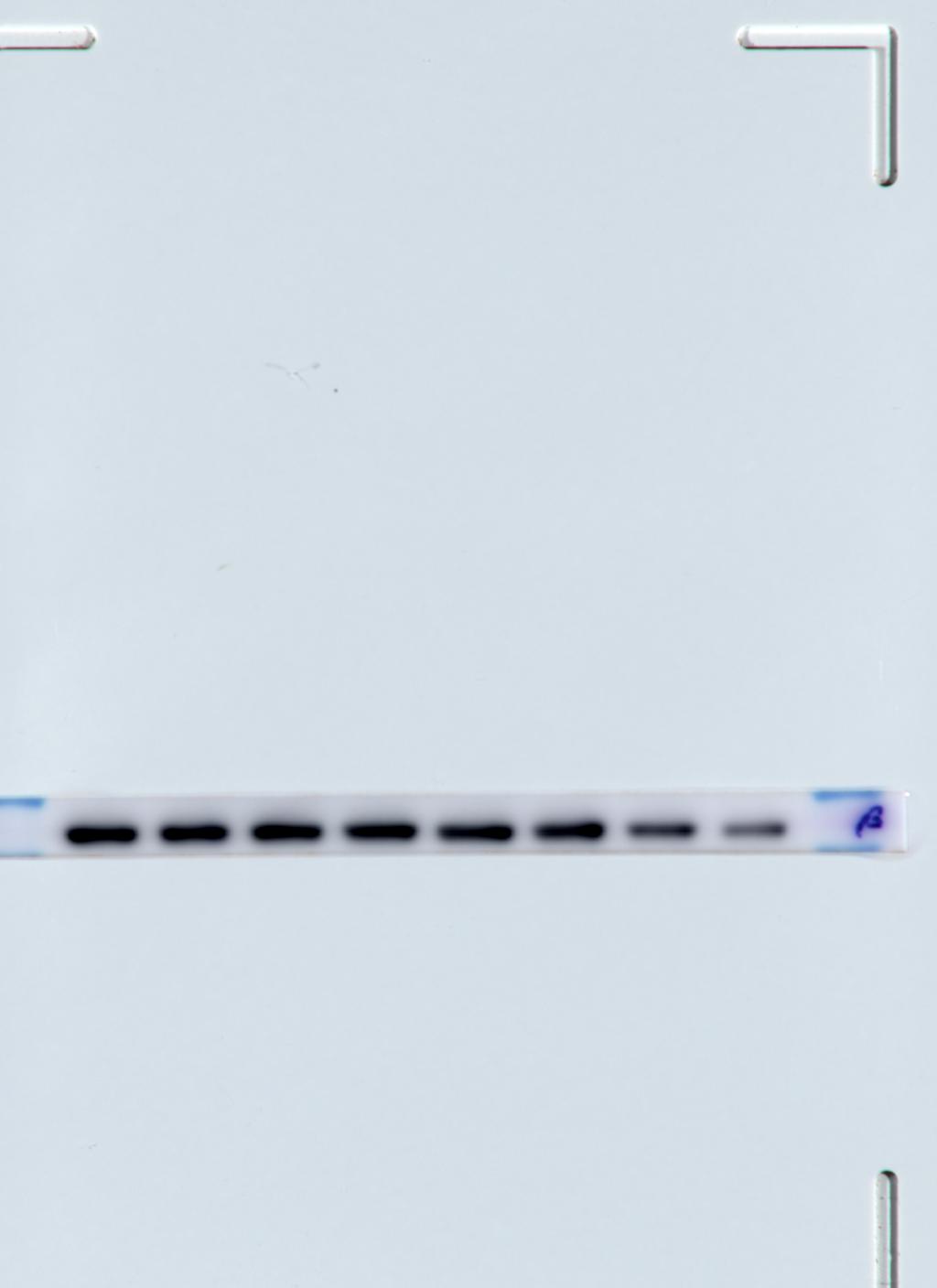
 35kDa

55kDa

POU5F1
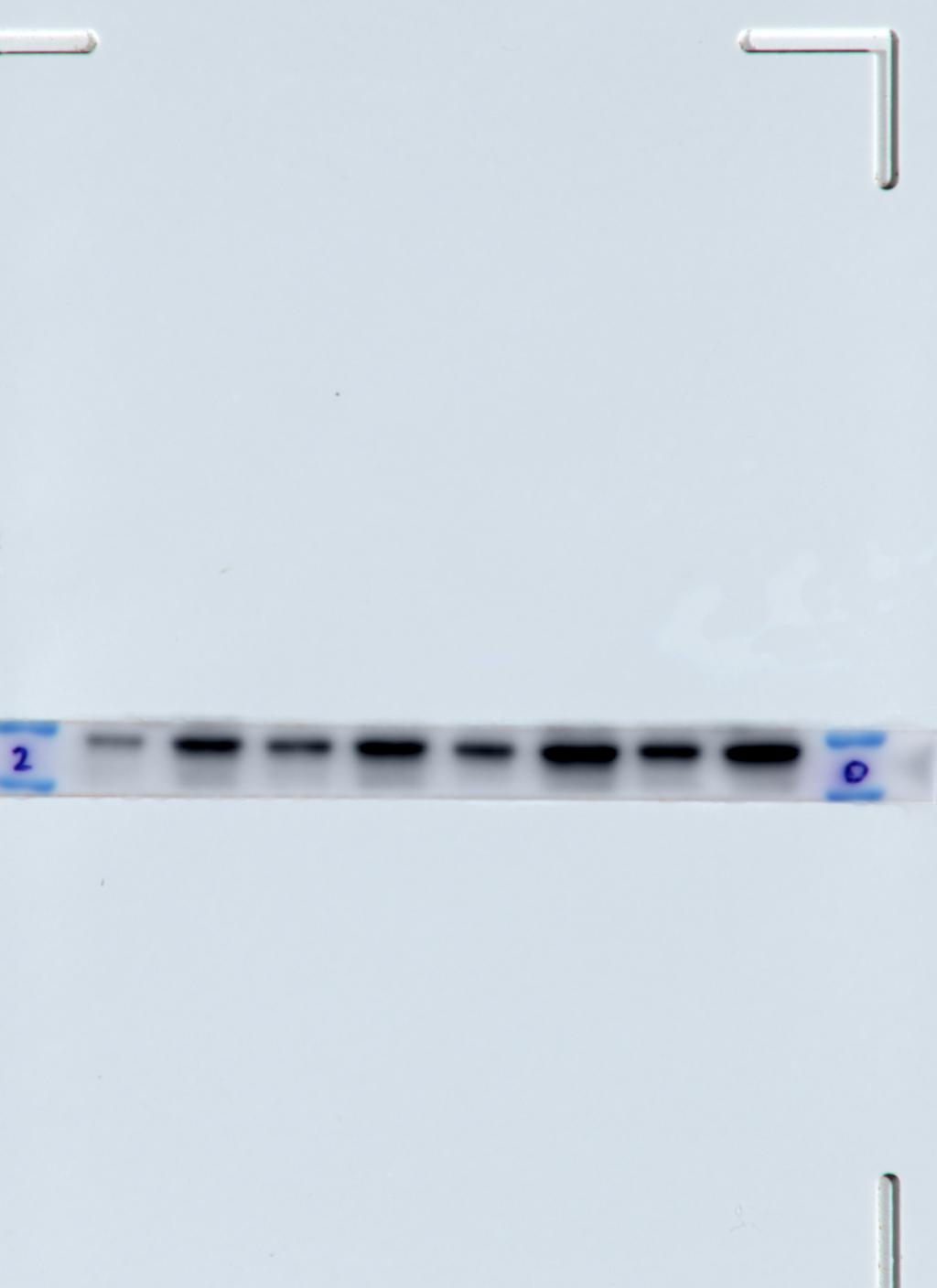
 40kDa

40kDa

GAPDH
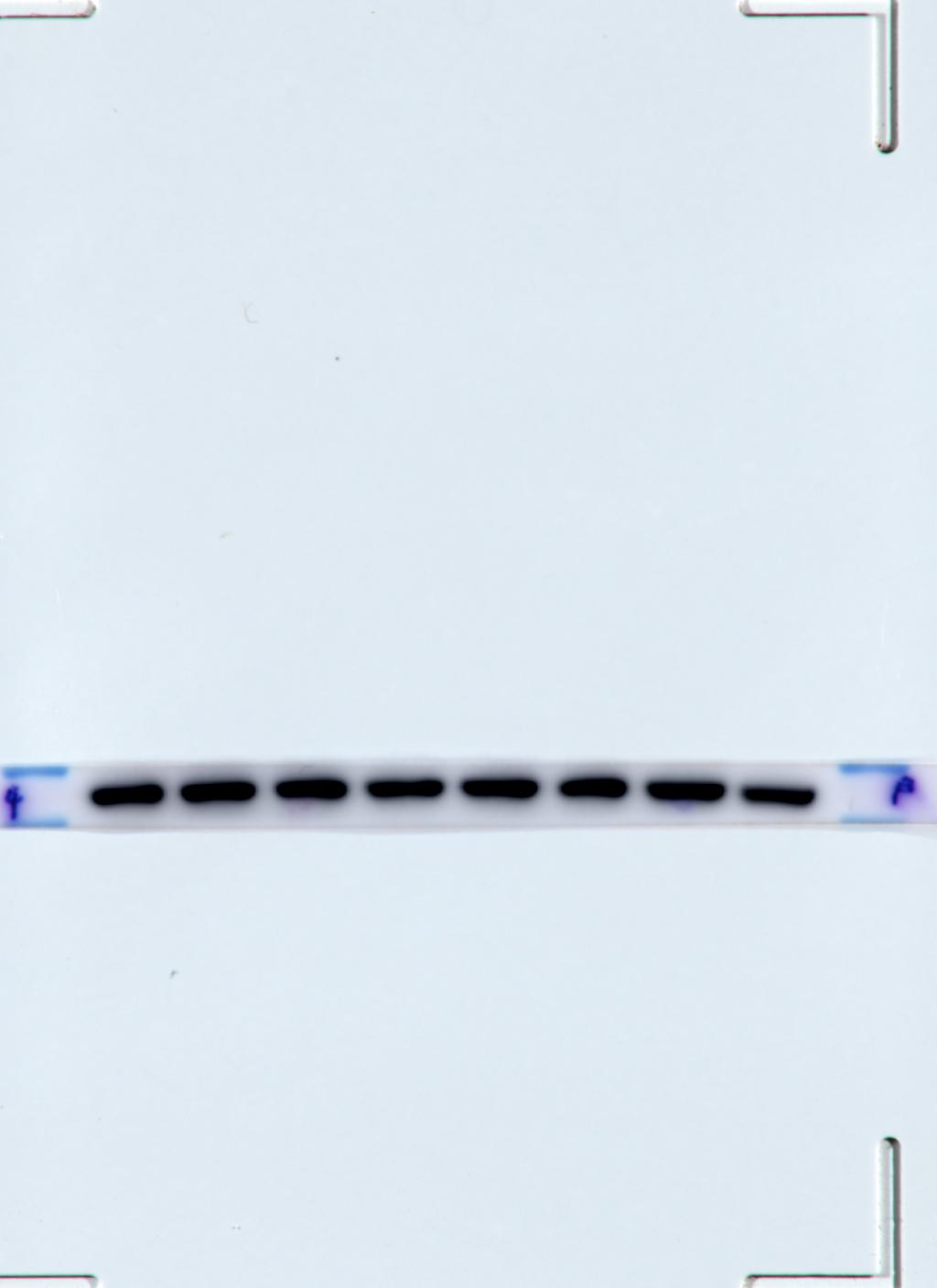
 35kDa

55kDa

POU5F1
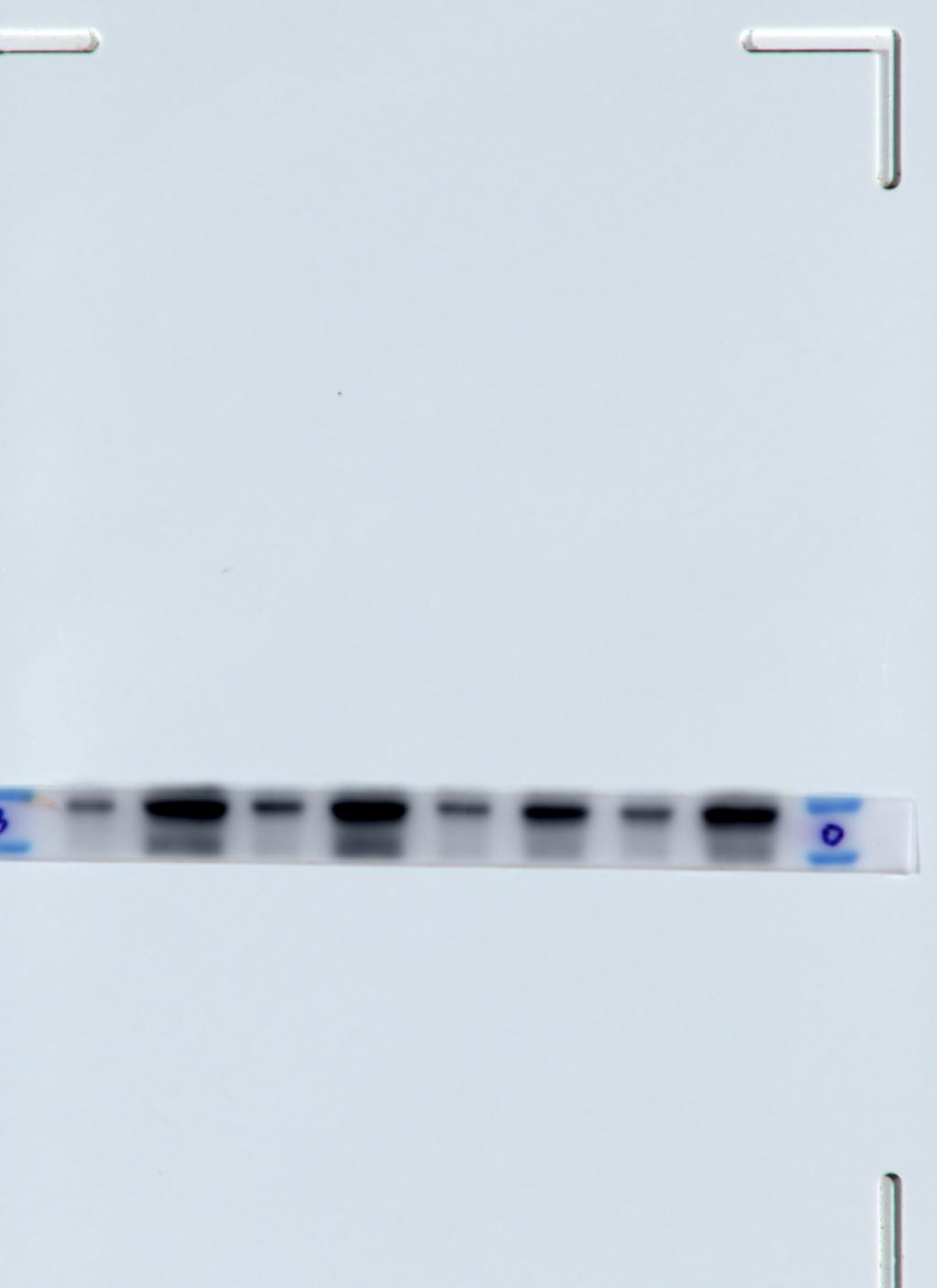
 40kDa

40kDa

GAPDH
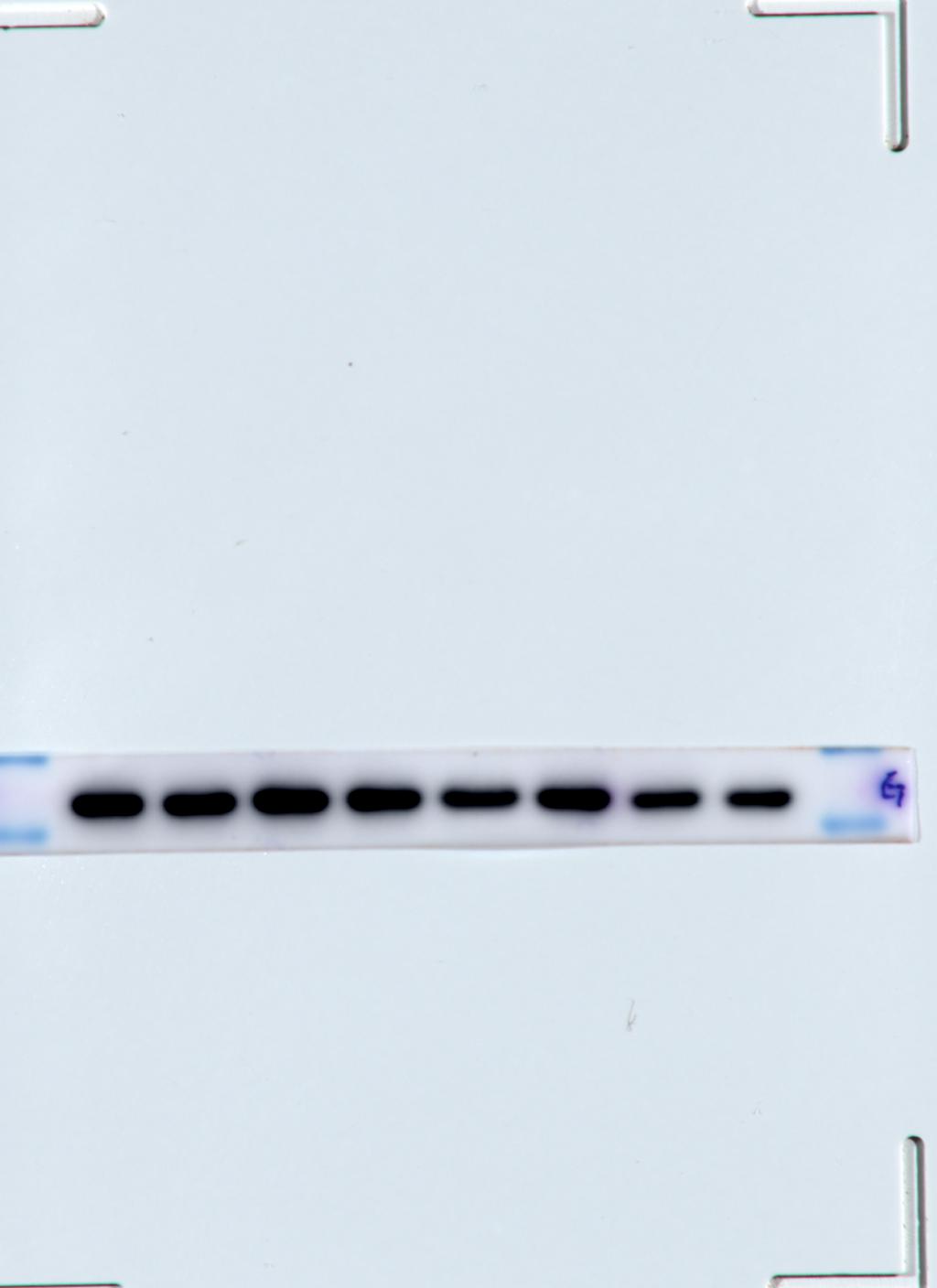
 35kDa

Figure 2

55kDa

POU5F1
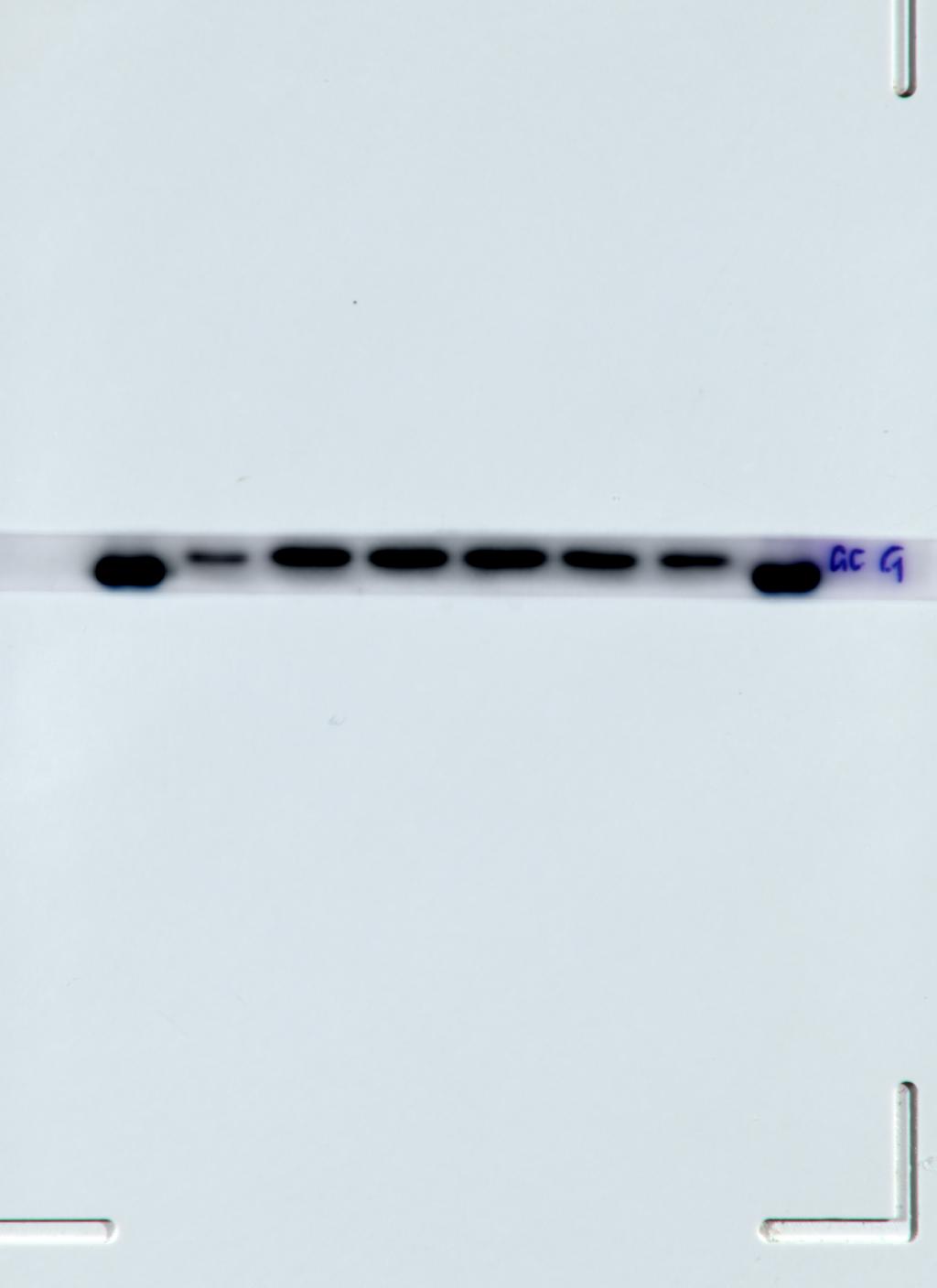
 40kDa

40kDa

GAPDH
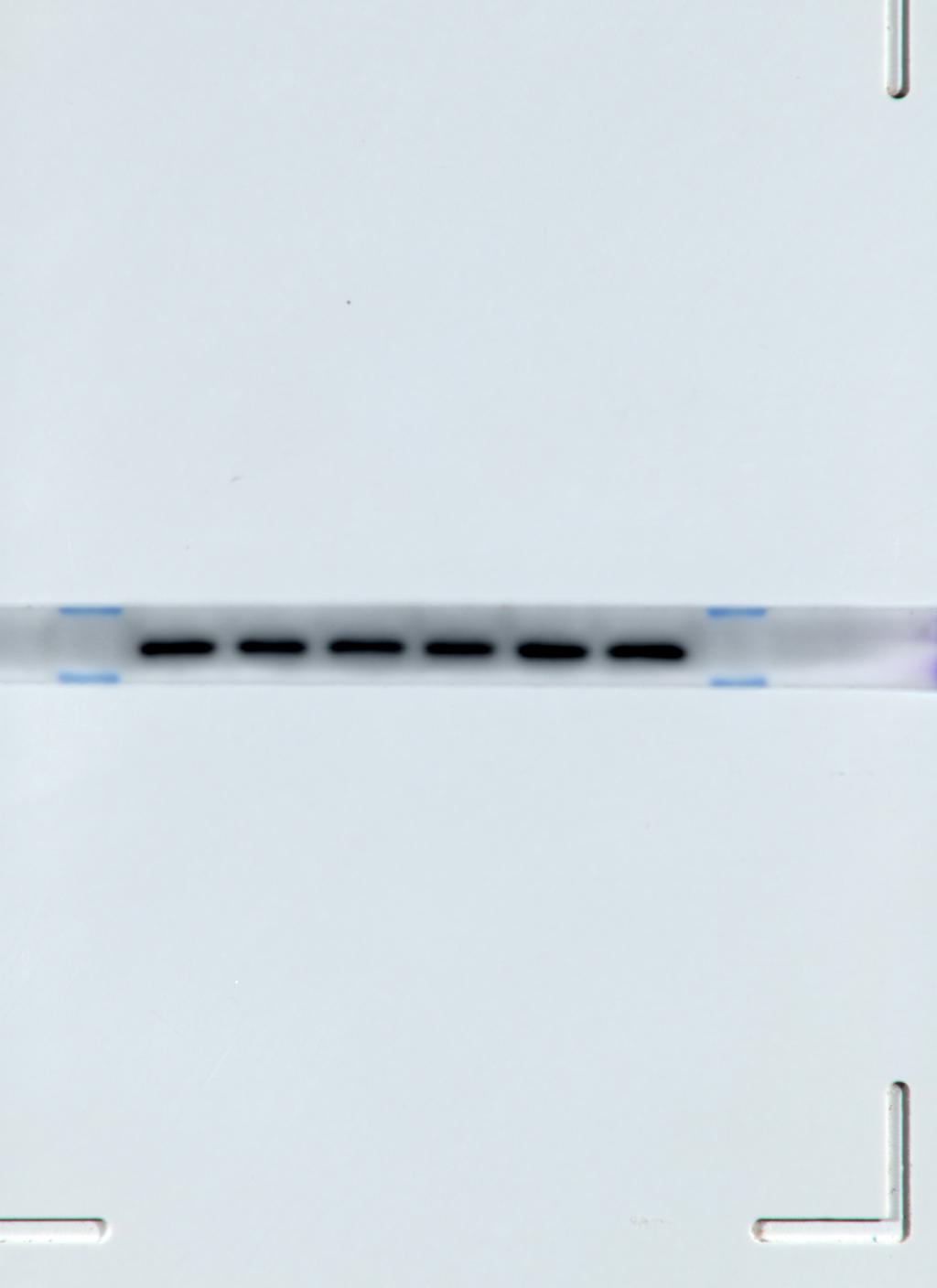
 35kDa

180kDa

N-Cad
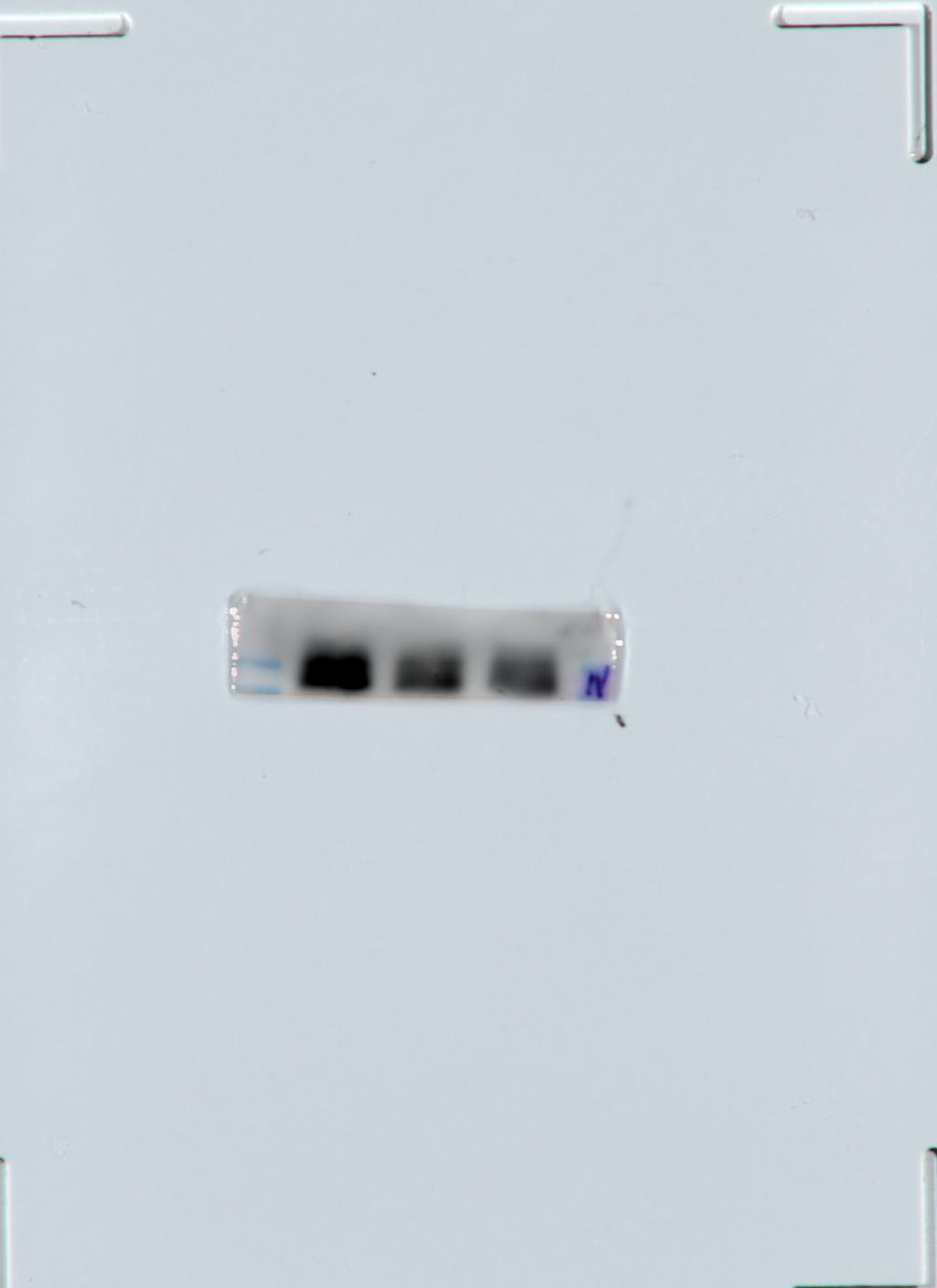
 130kDa

100kDa

E-Cad
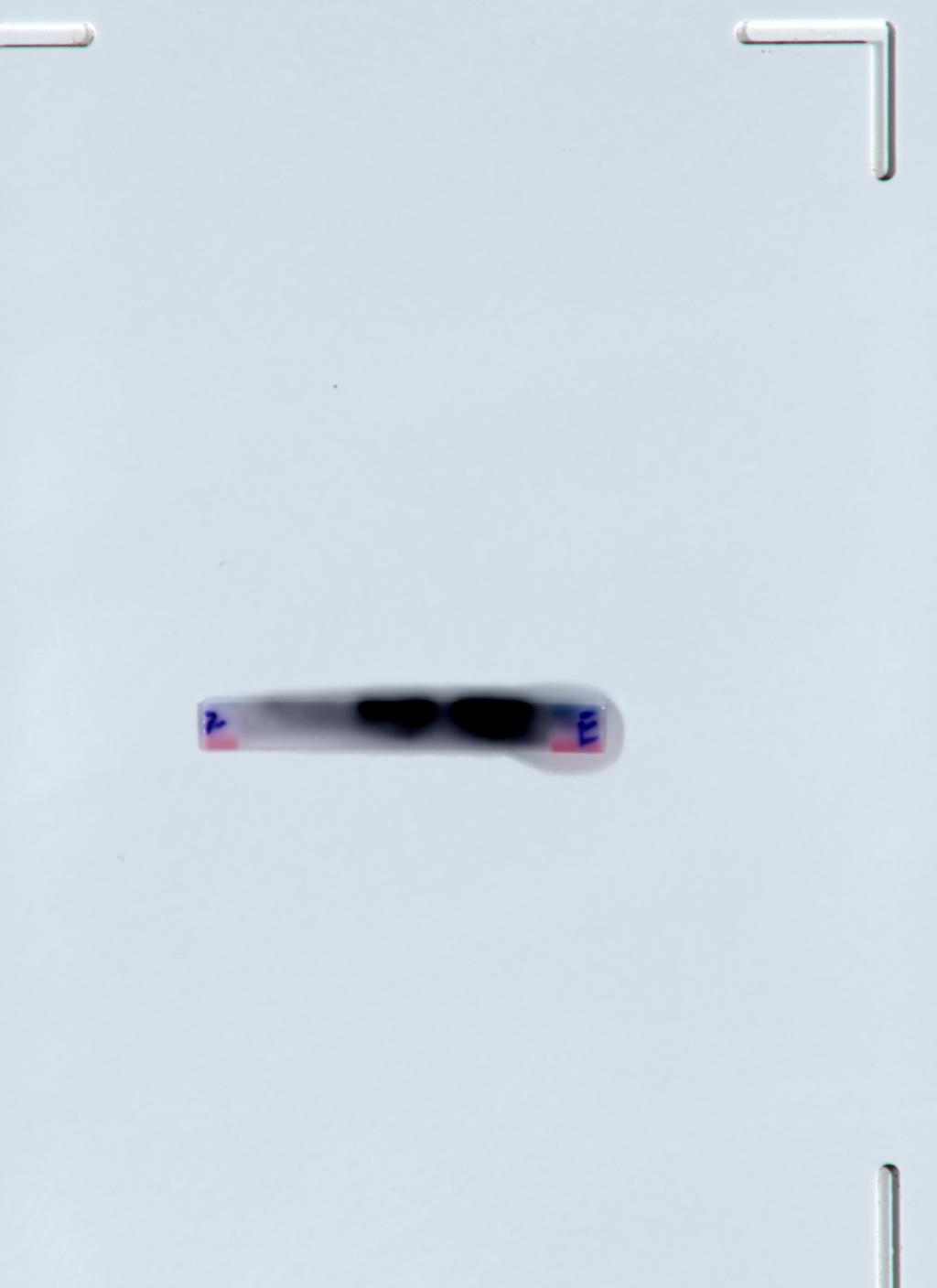
 70kDa

70kDa

VIM
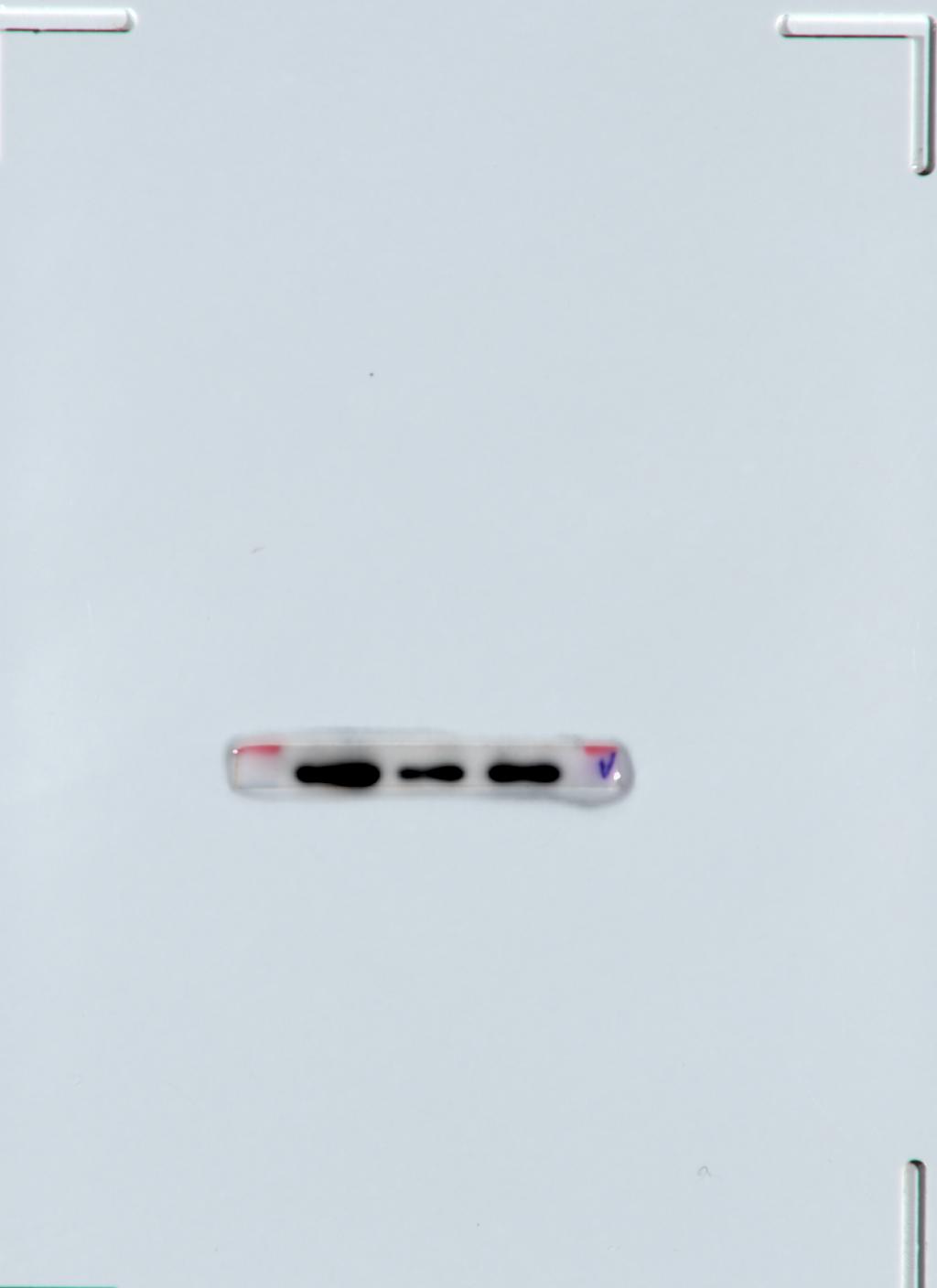
 55kDa

55kDa

POU5F1
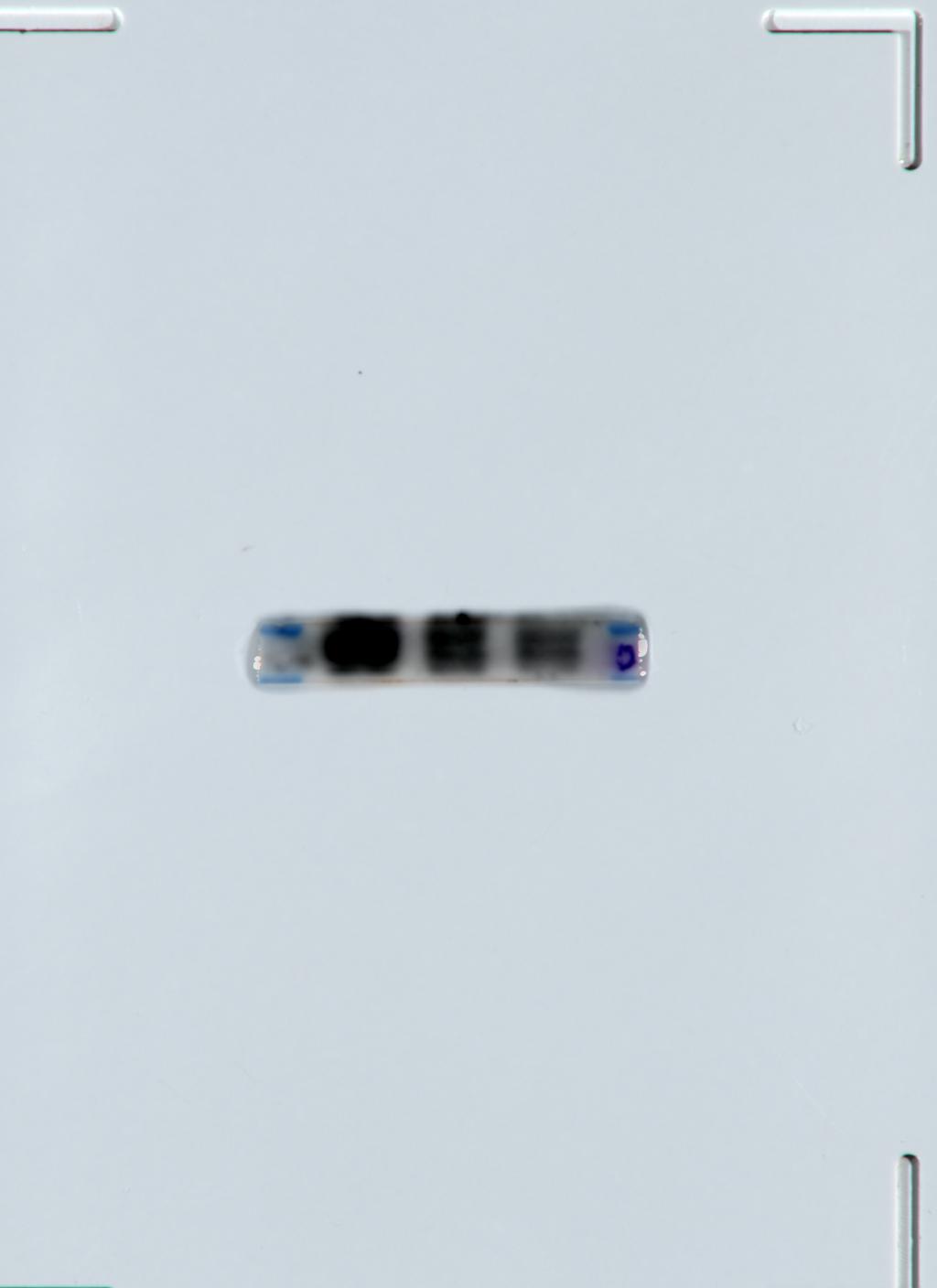
 40kDa

40kDa

GAPDH
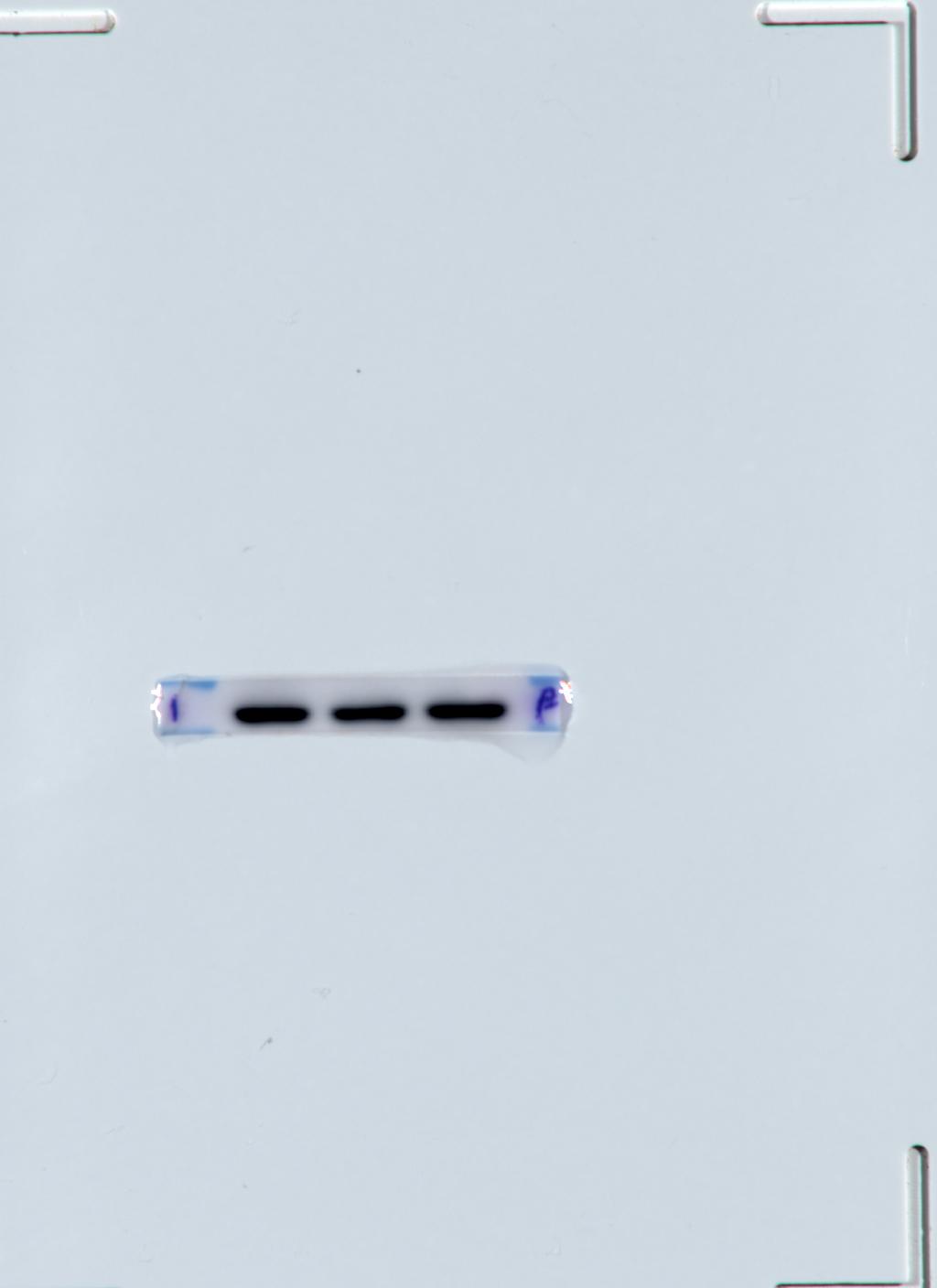
 35kDa

180kDa

N-Cad
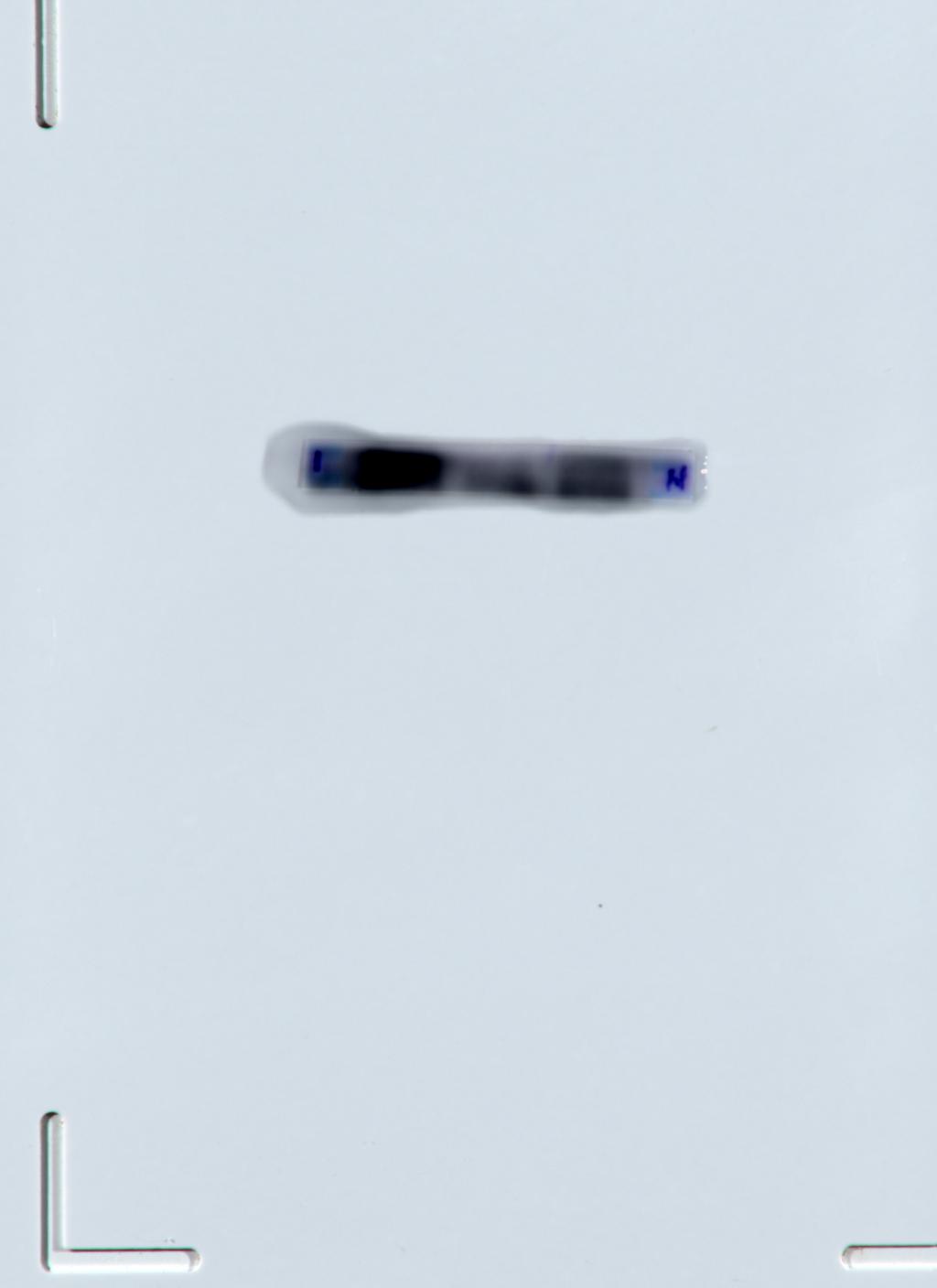
 130kDa

100kDa

E-Cad
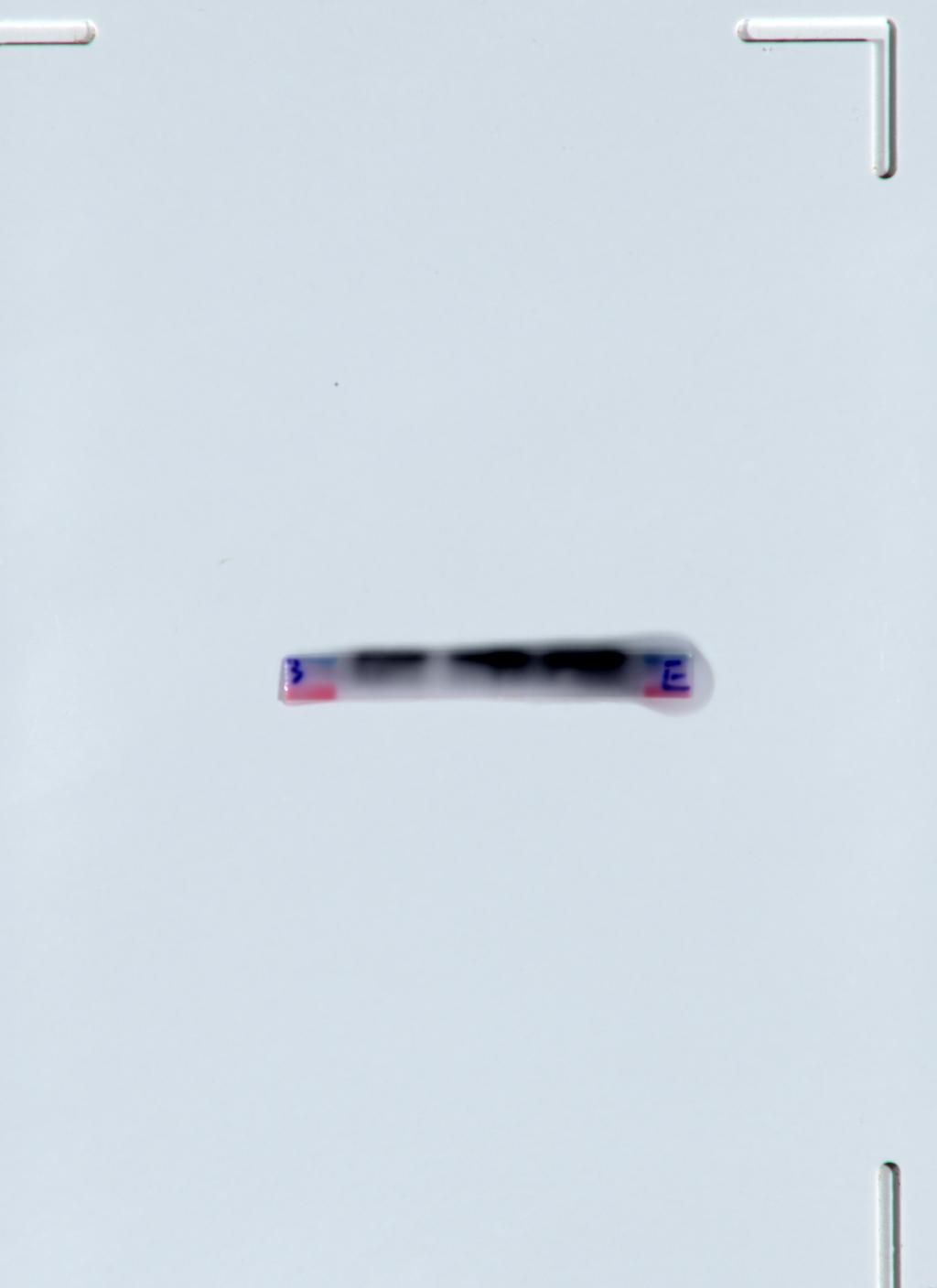
 70kDa

70kDa

VIM
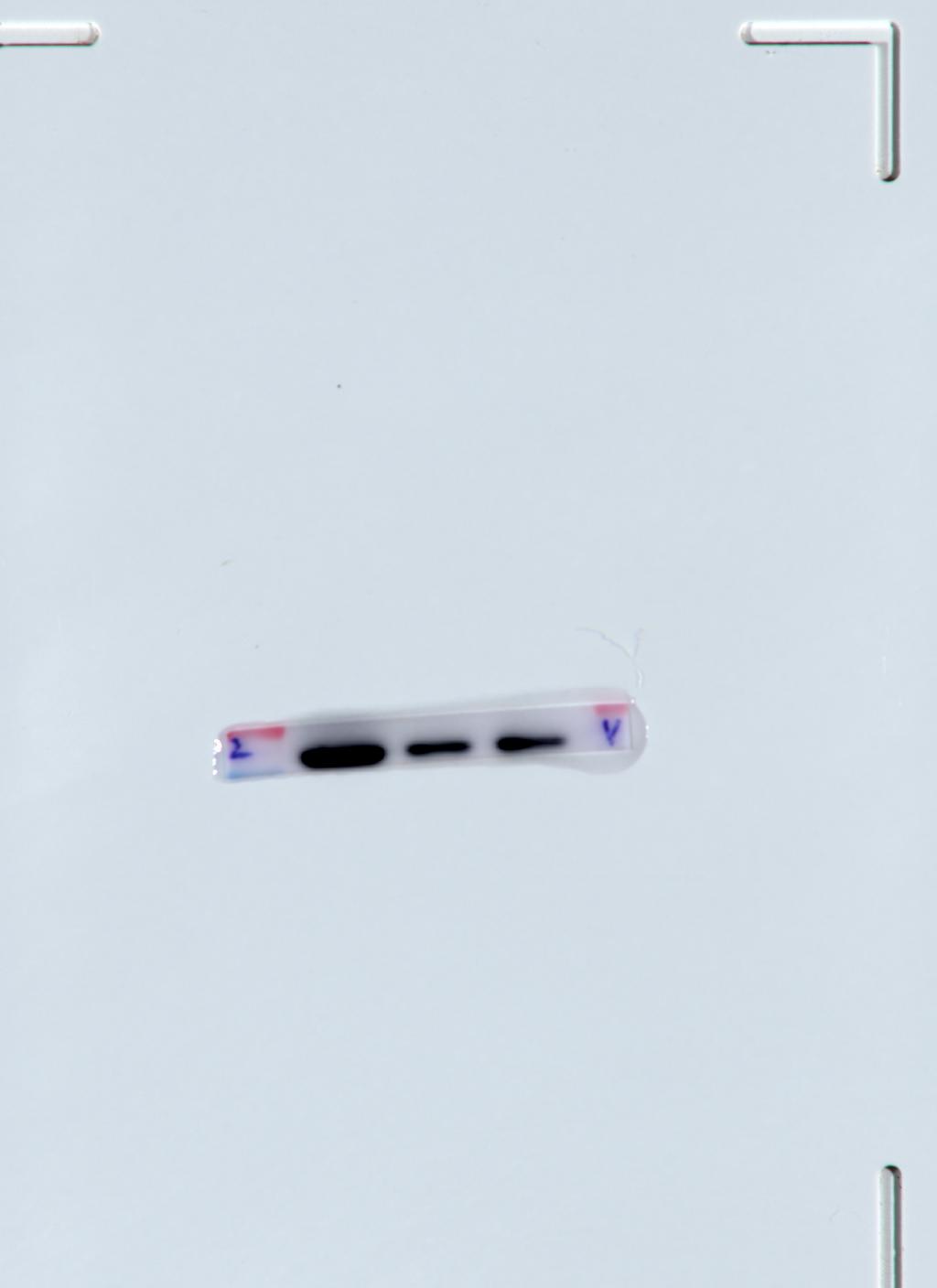
 55kDa

55kDa

POU5F1
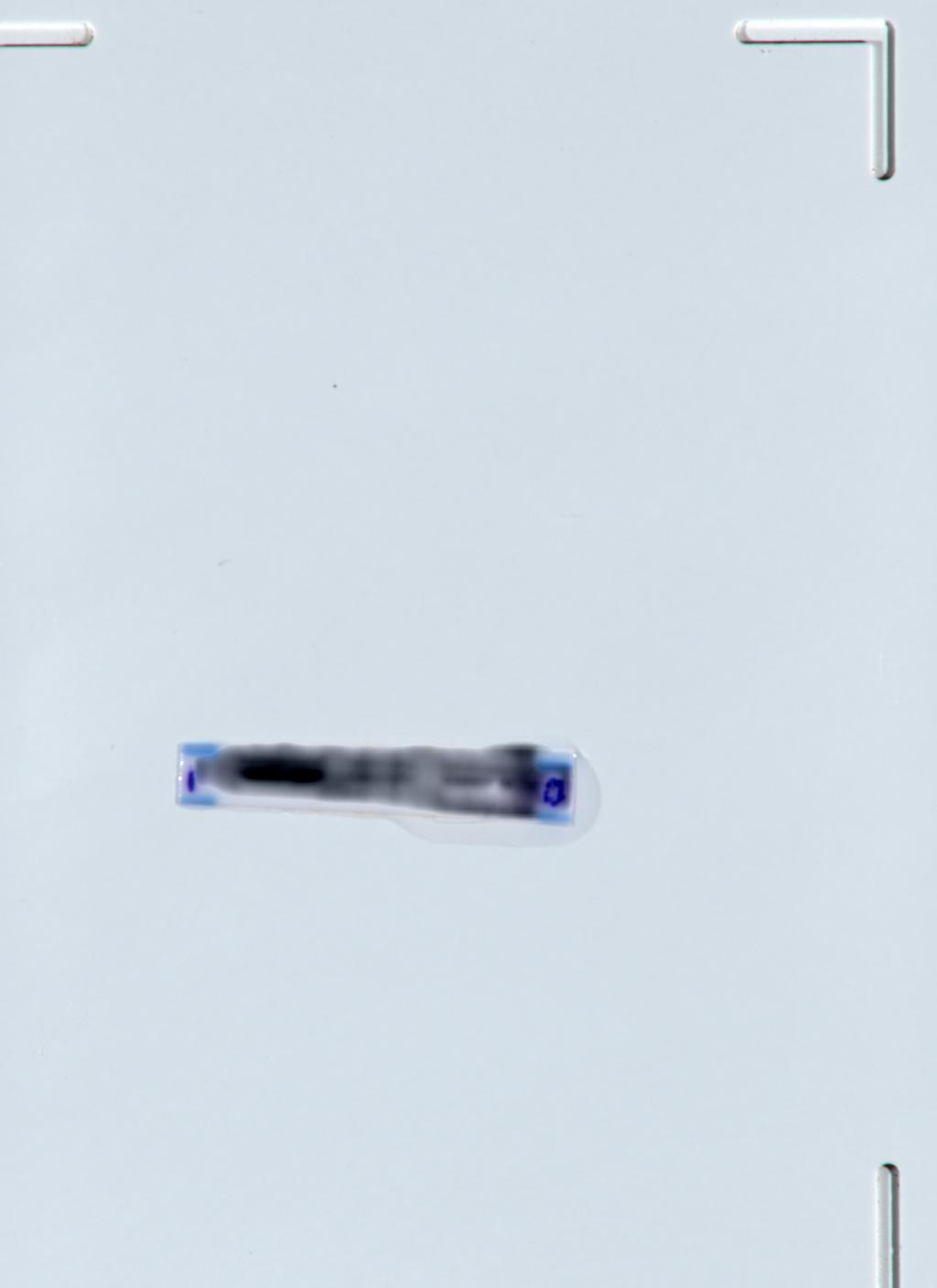
 40kDa

40kDa

GAPDH
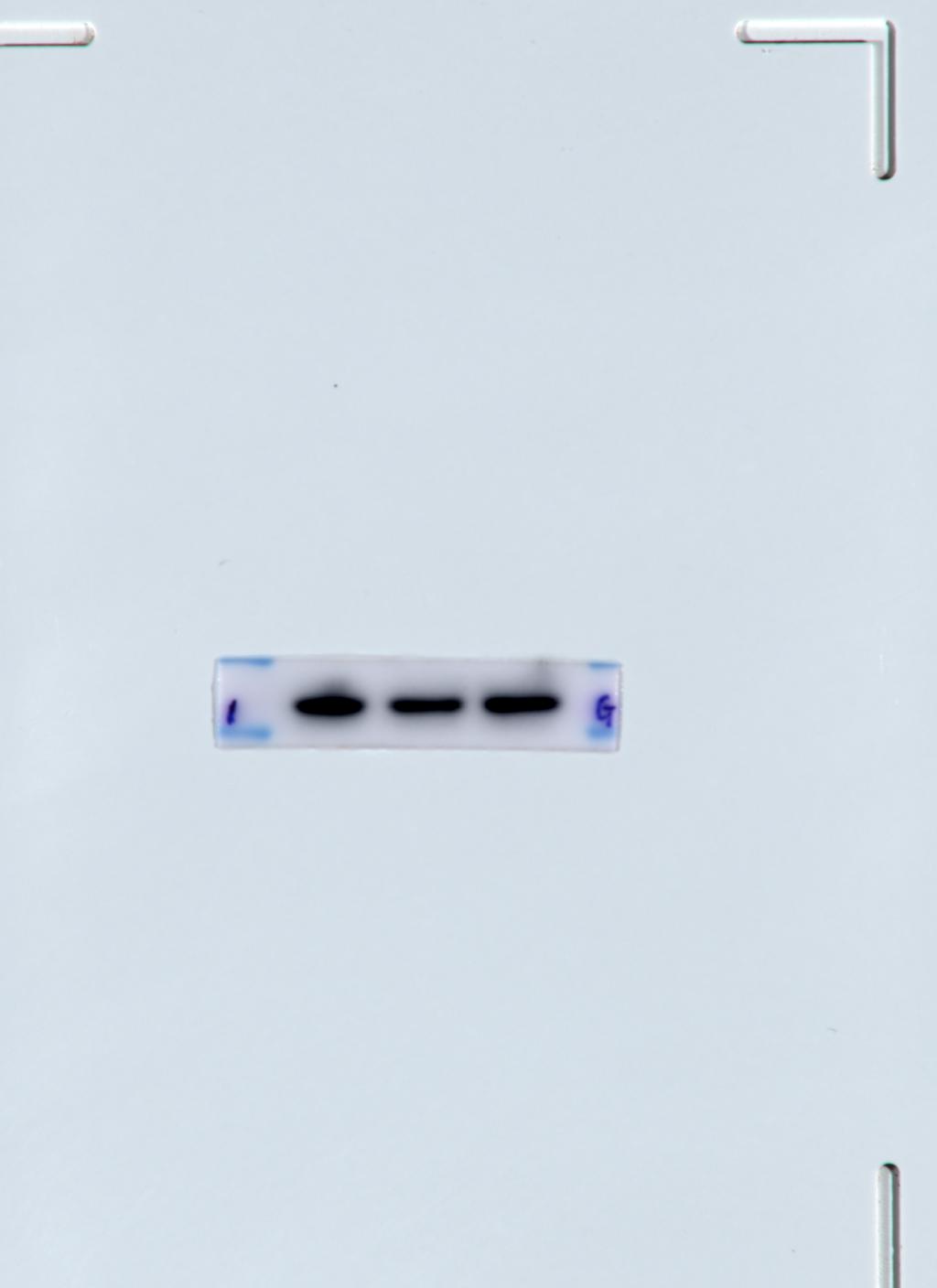
 35kDa

180kDa

N-Cad
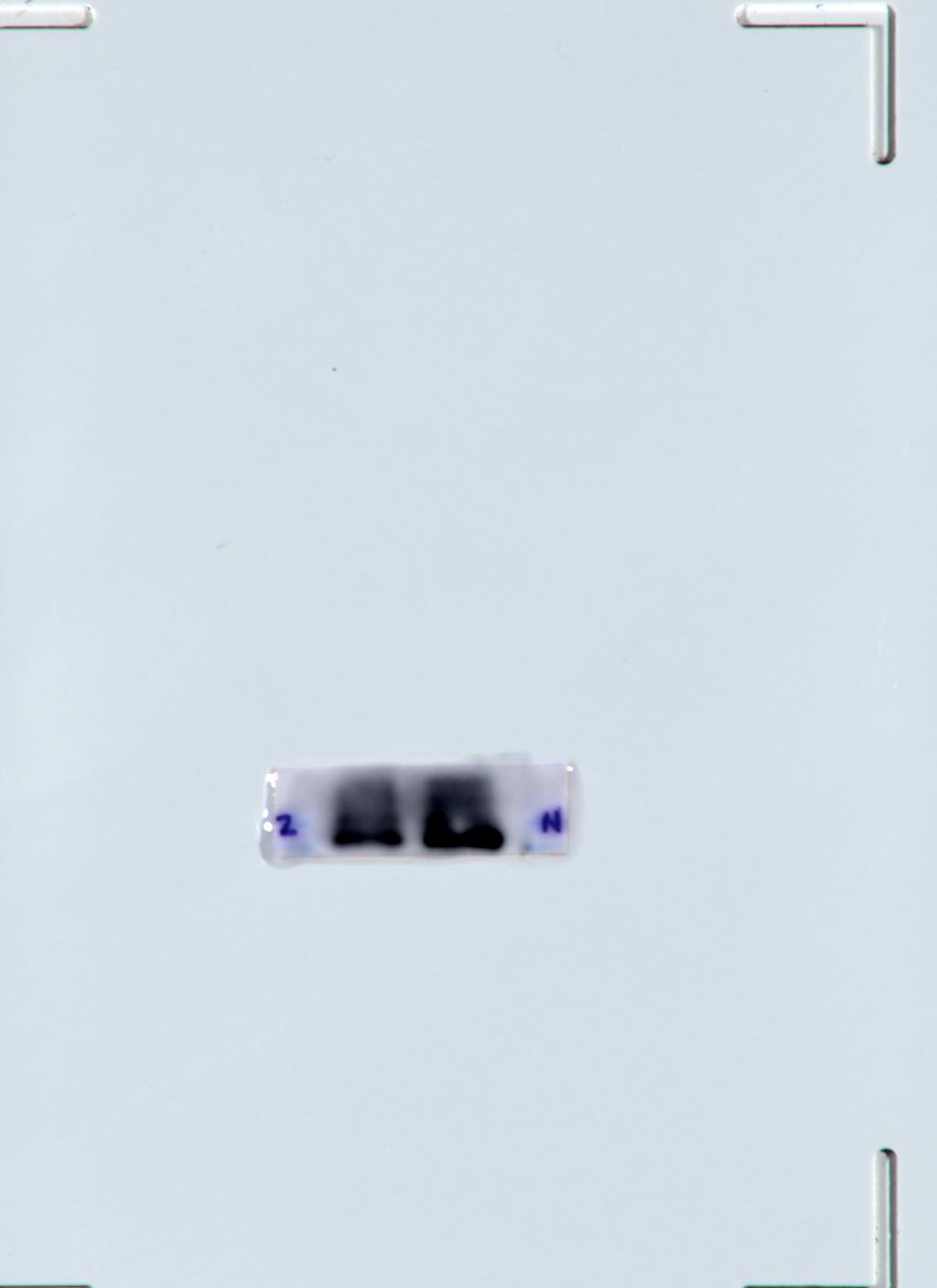

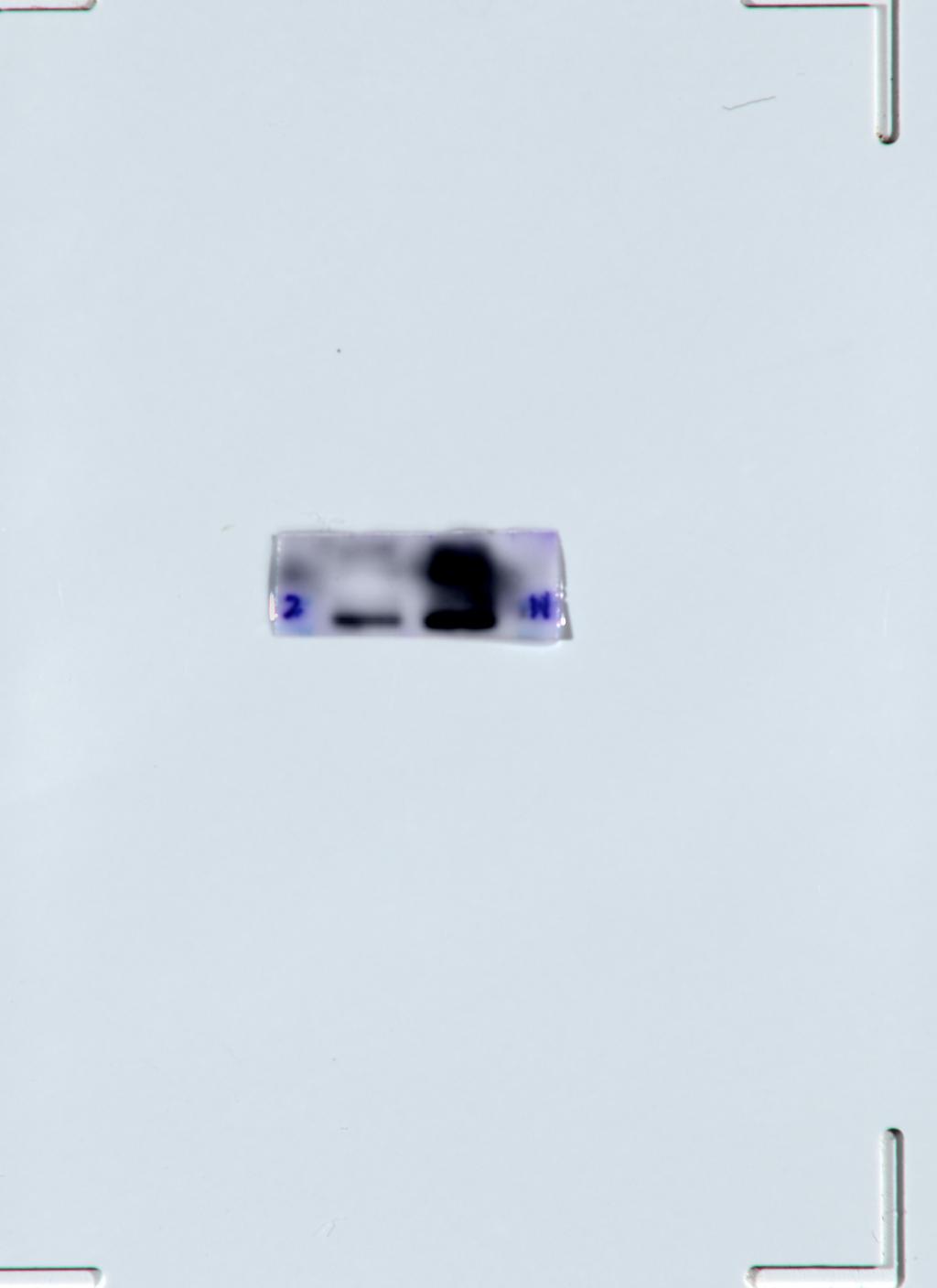
 130kDa

100kDa

E-Cad
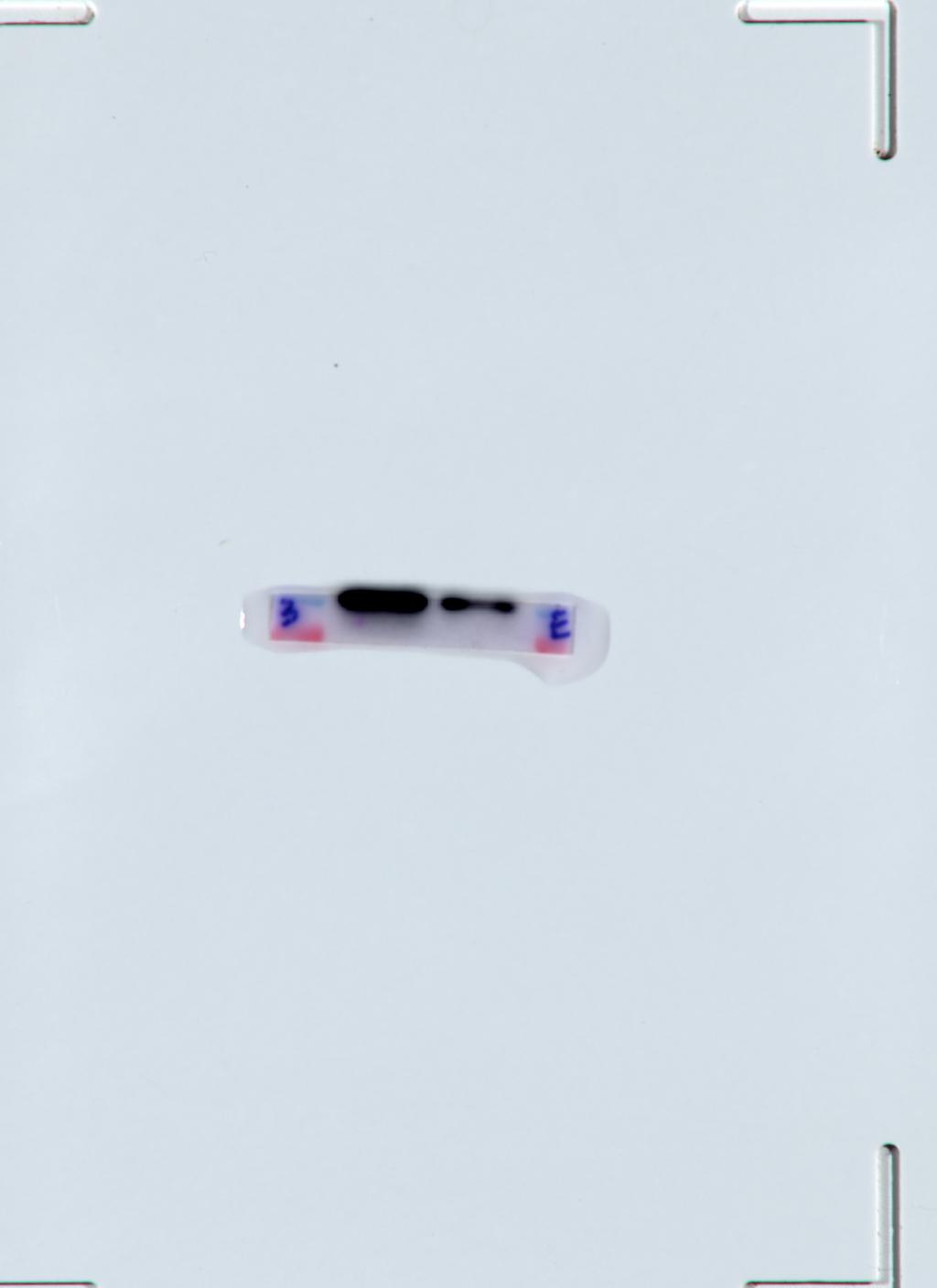

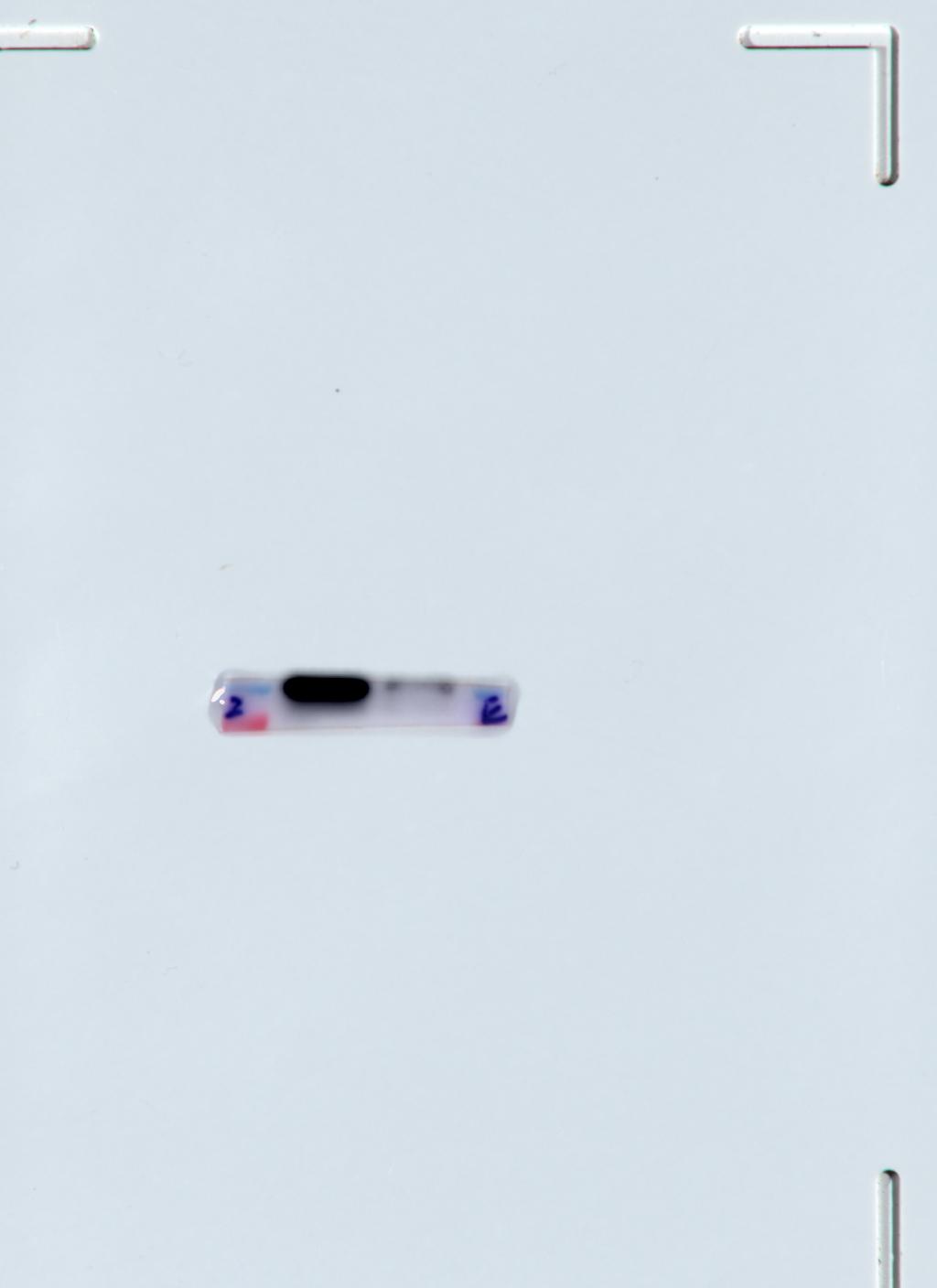
 70kDa

70kDa

VIM
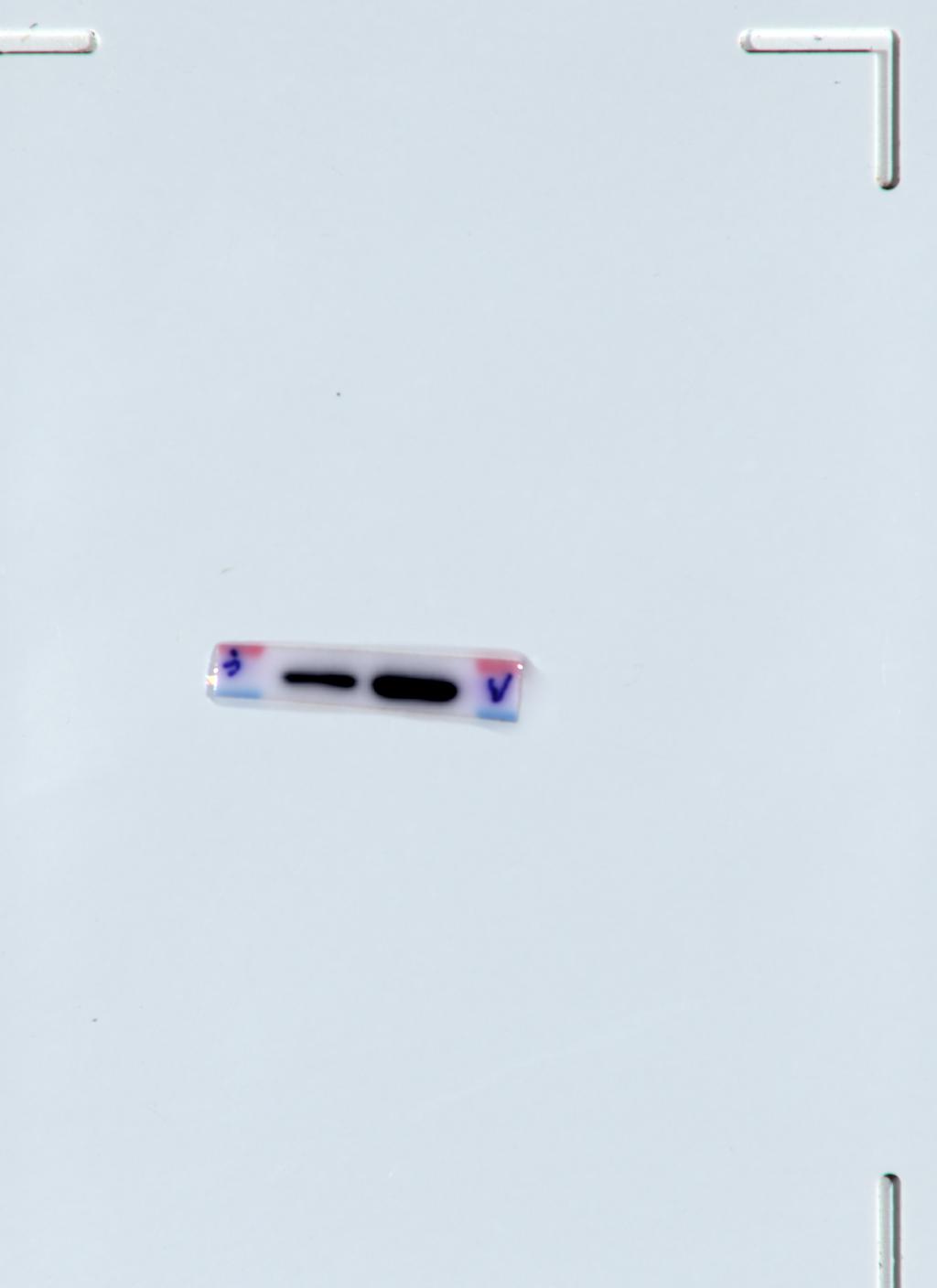

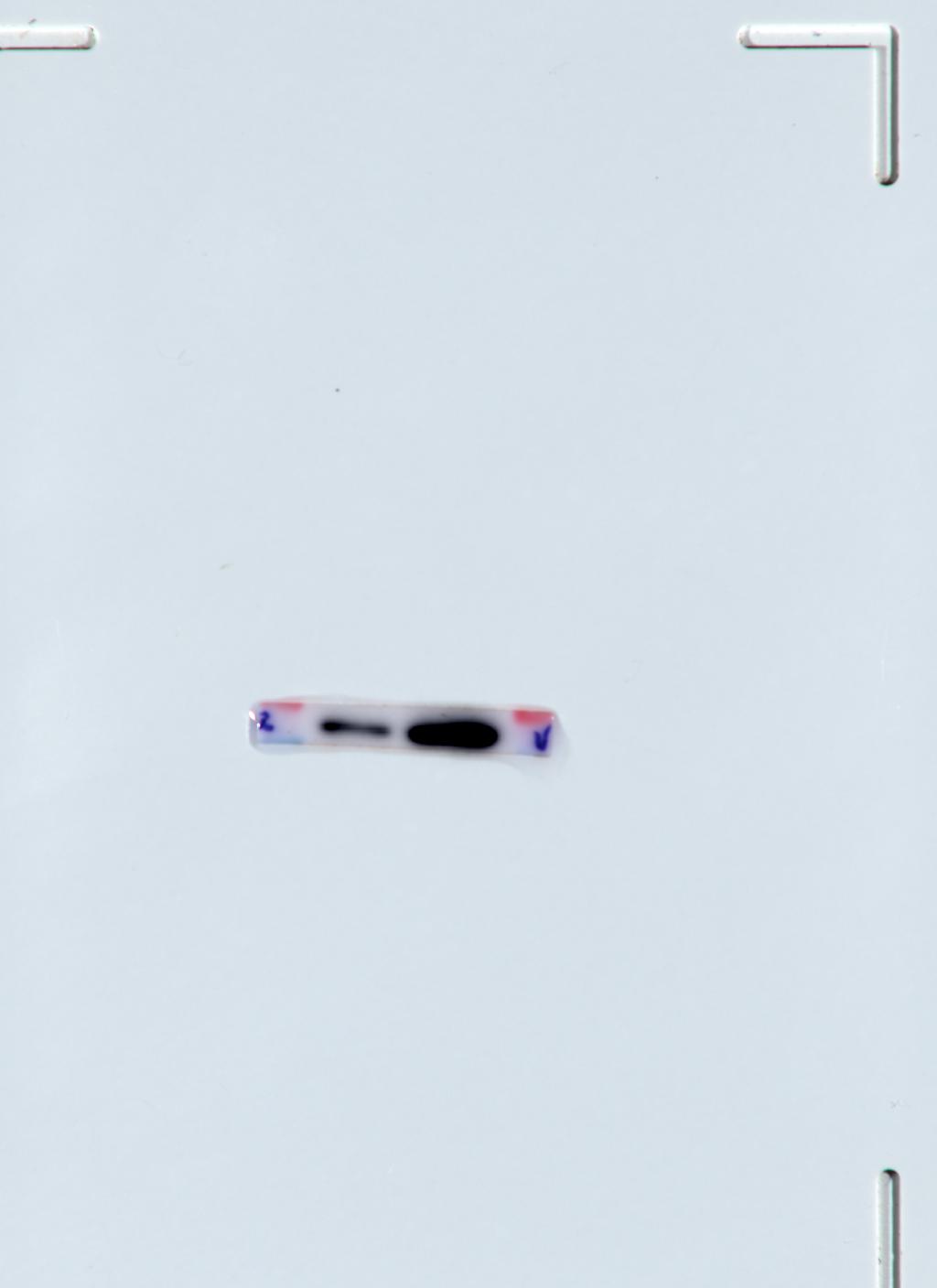
 55kDa

55kDa

POU5F1
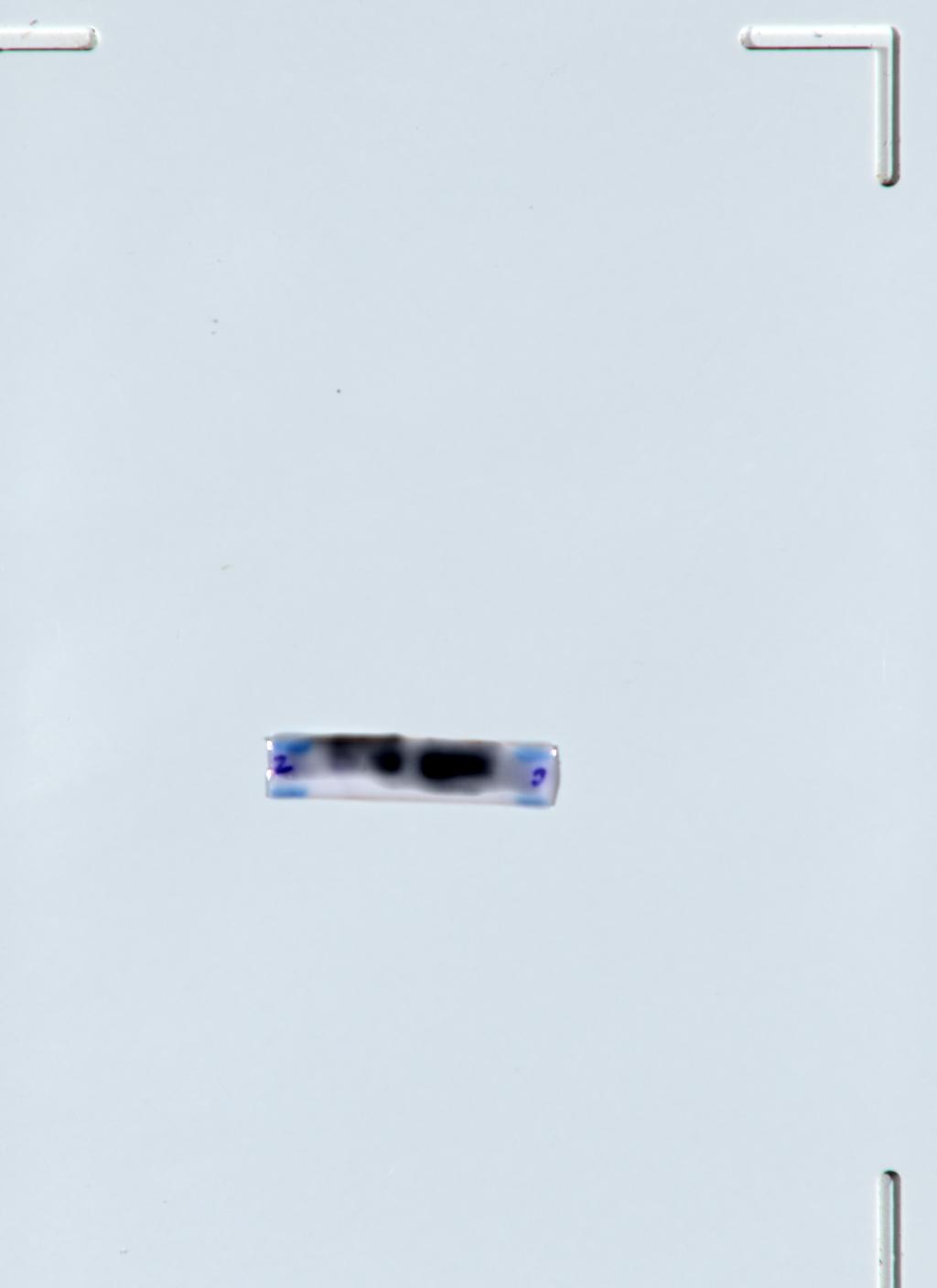

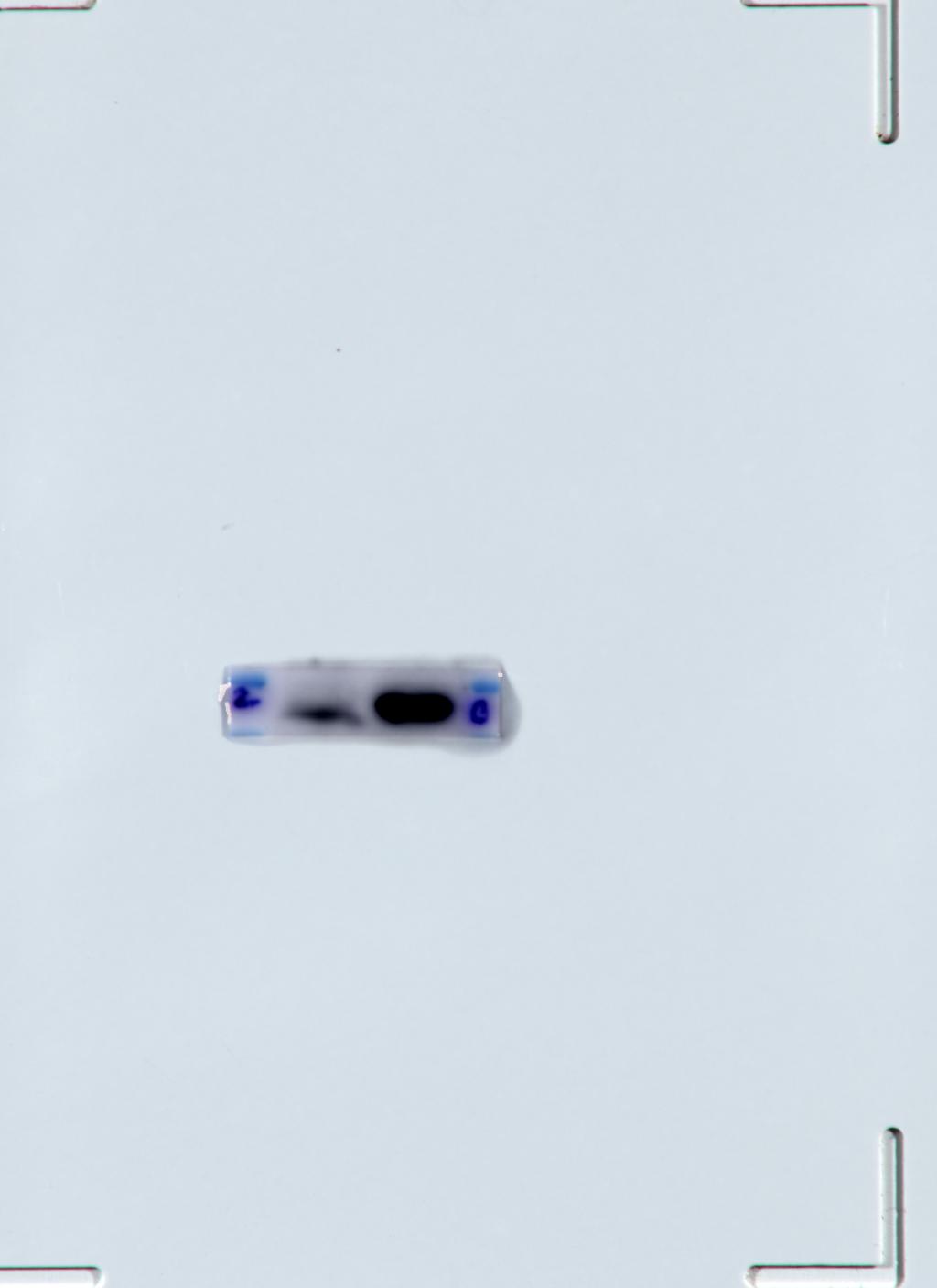
 40kDa

40kDa

GAPDH
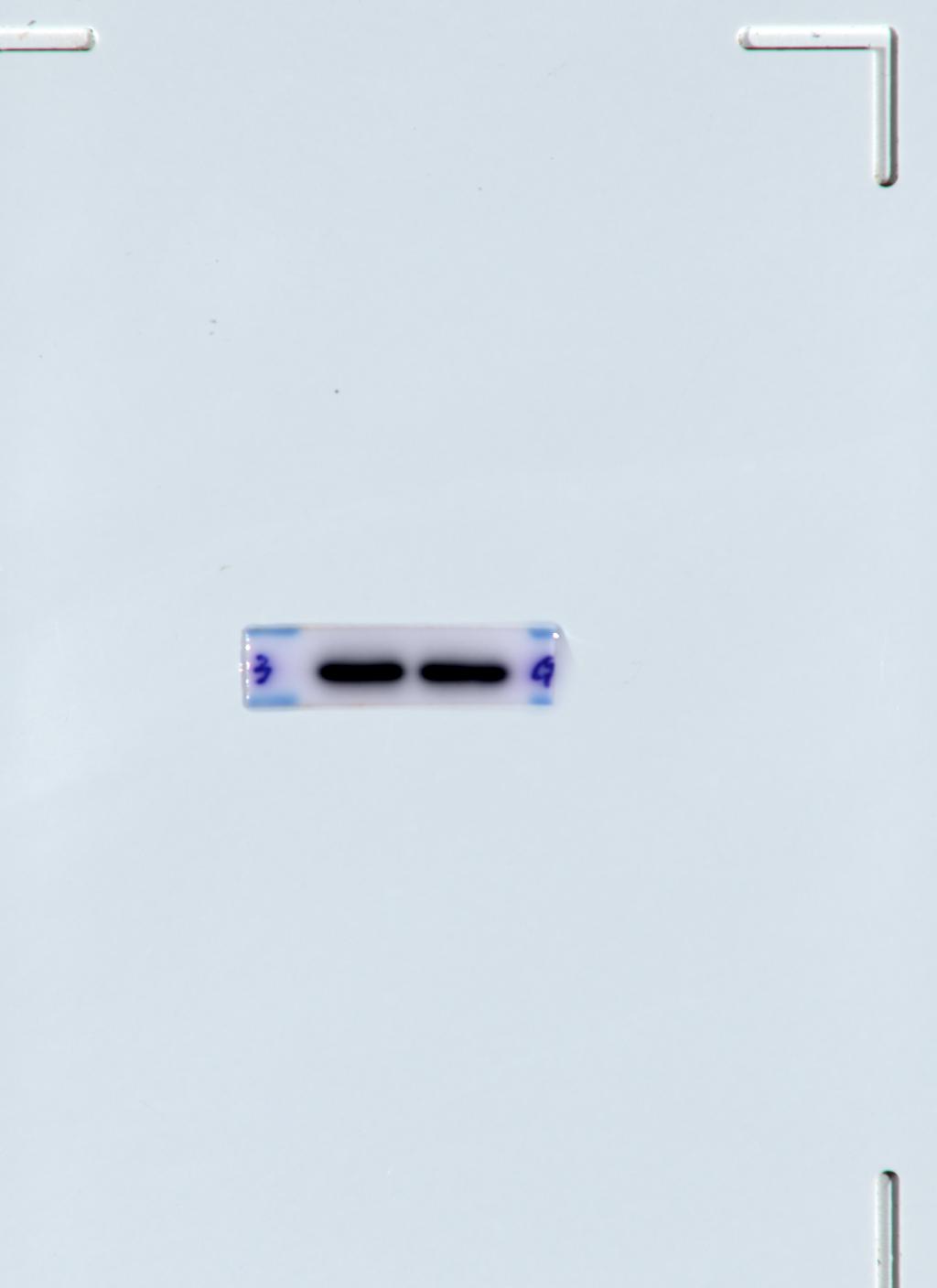

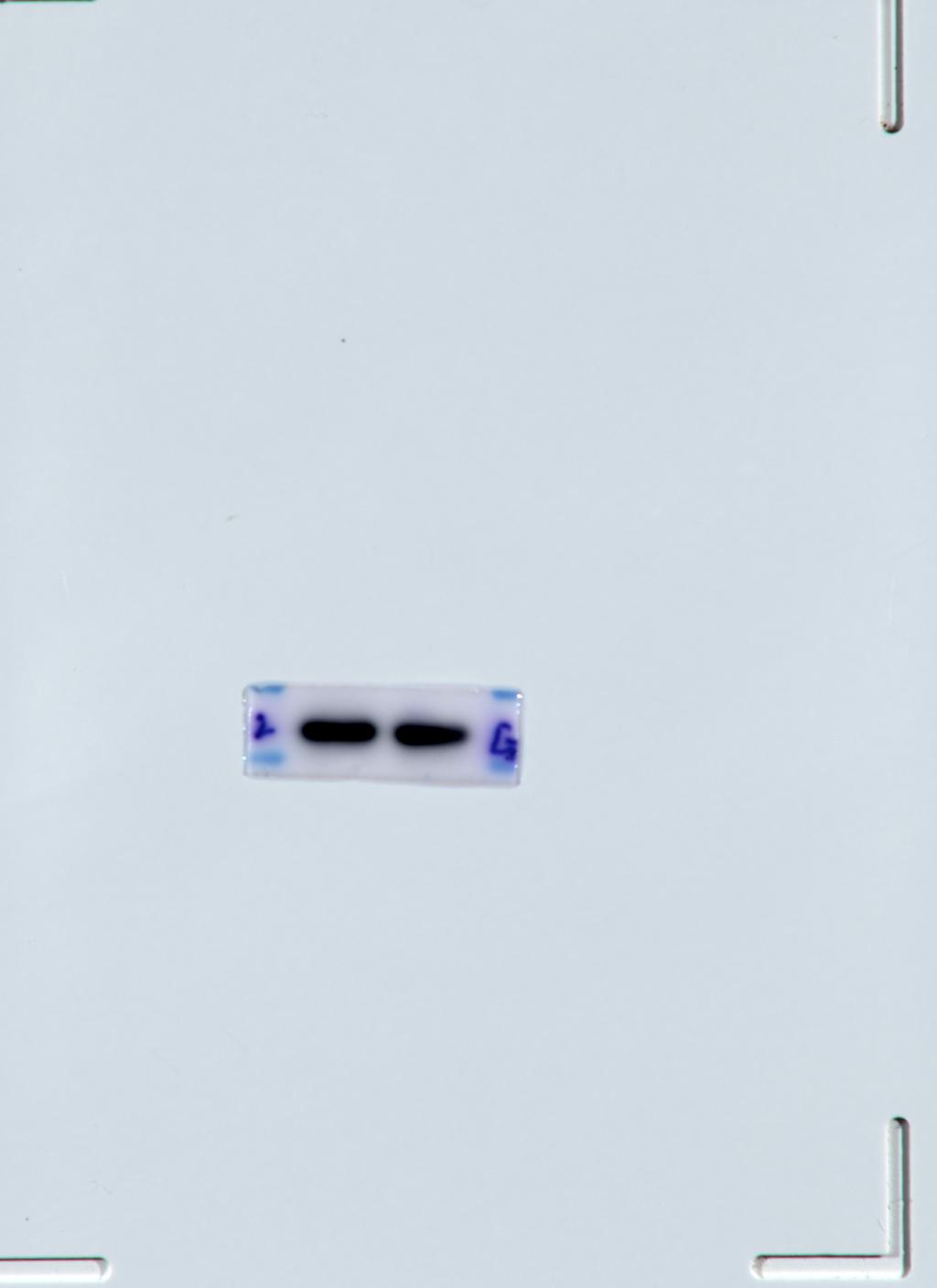
 35kDa

Figure 4

70kDa

NF-κB
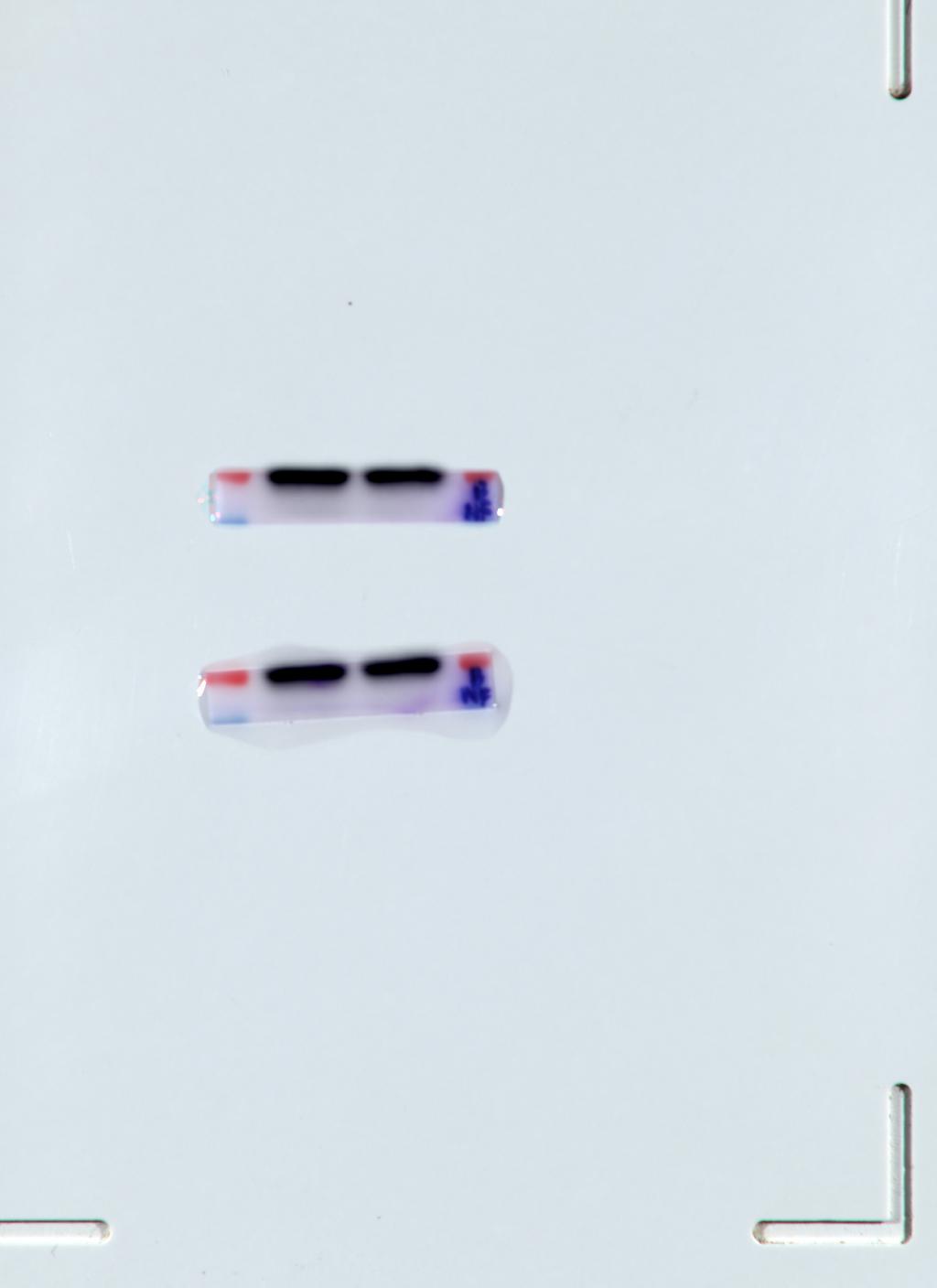

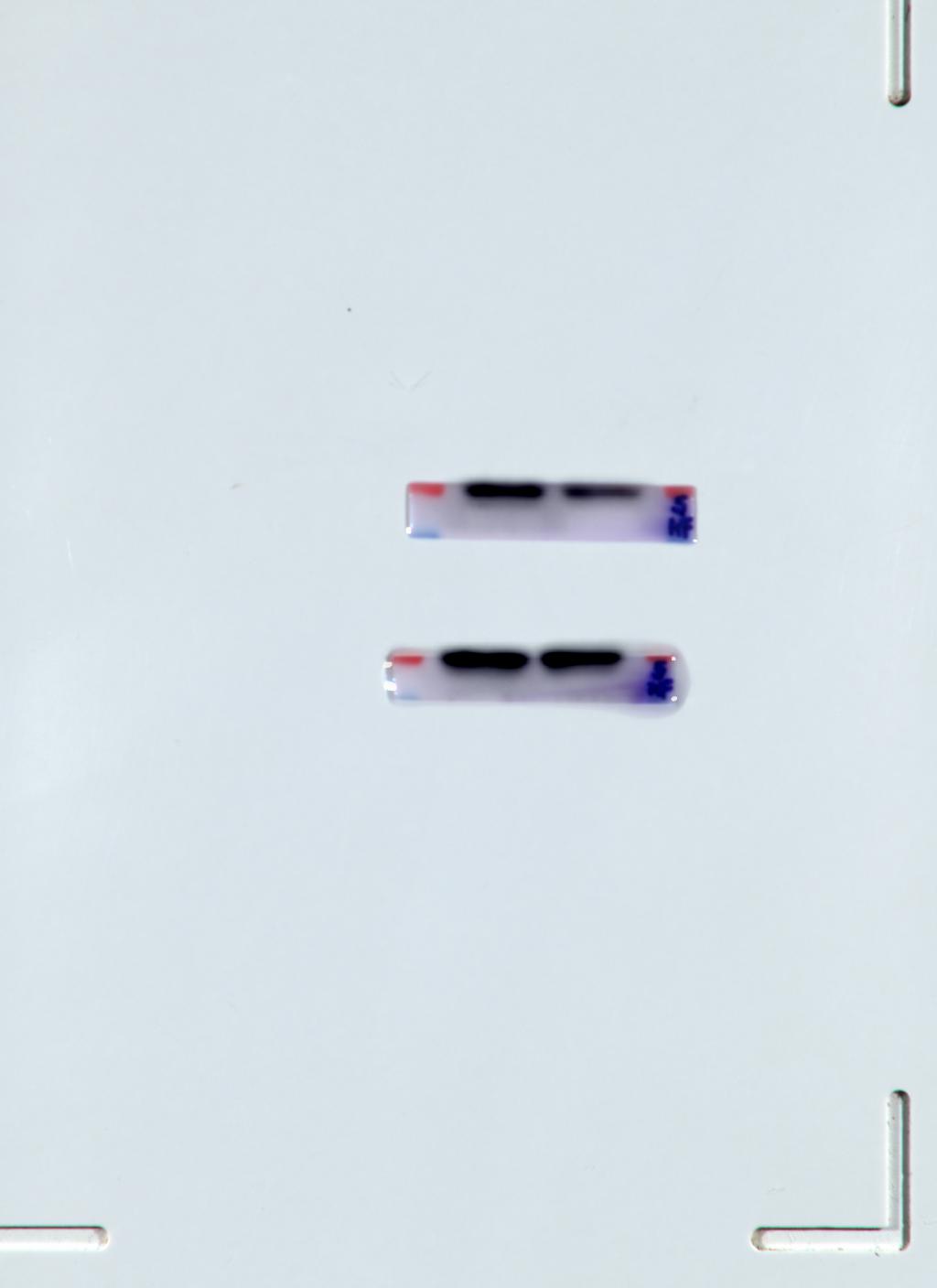
 55kDa

70kDa

P-NF-κB
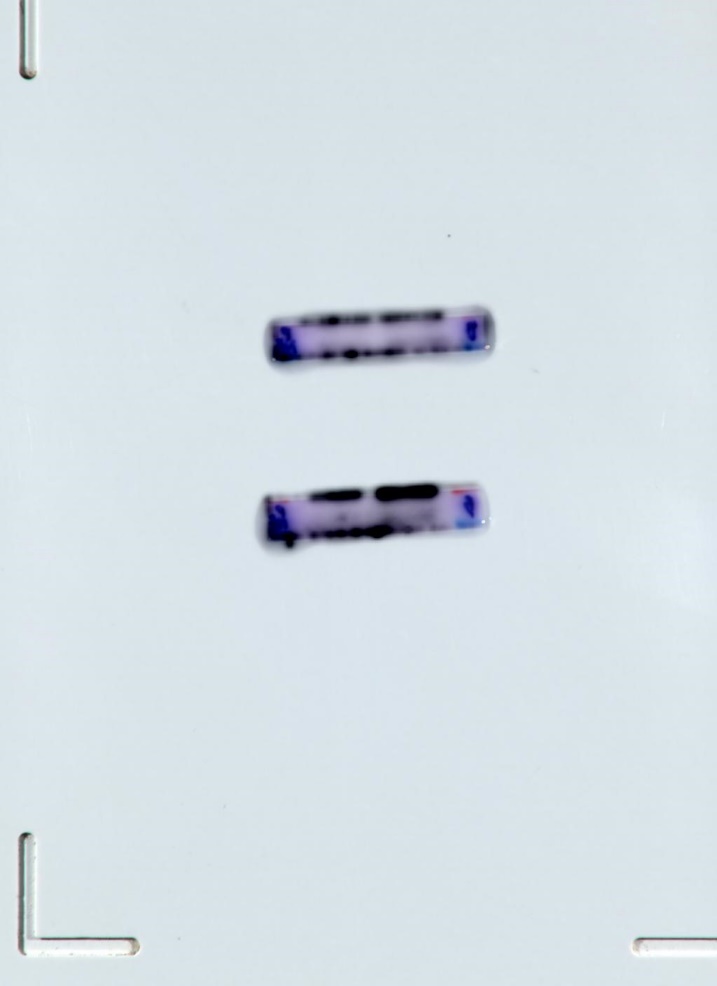

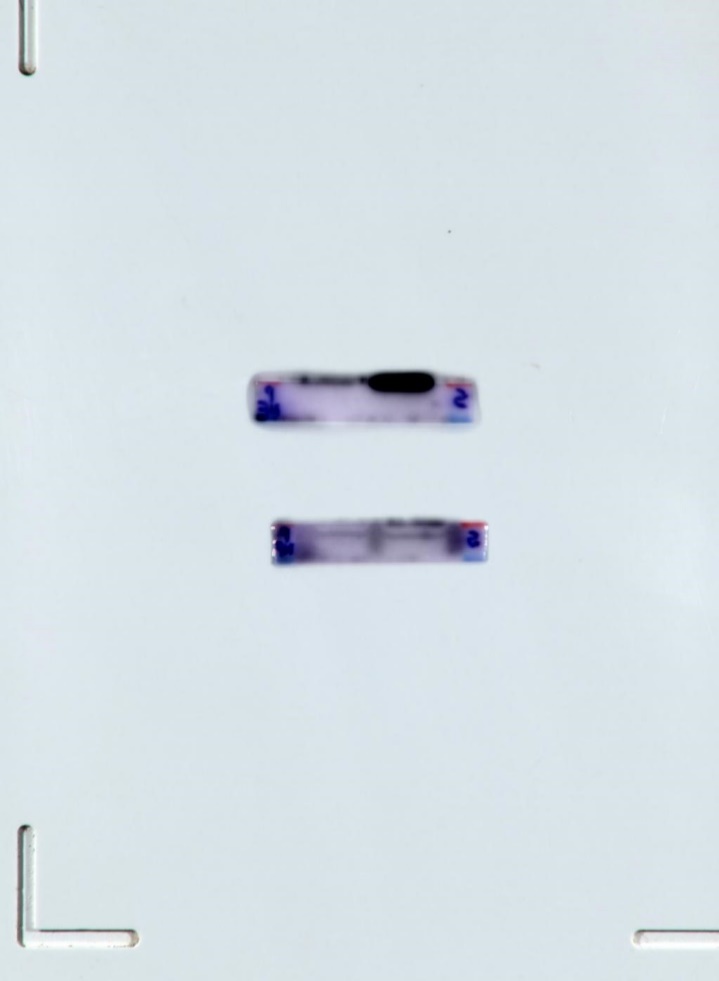
 55kDa

40kDa

TIRAP
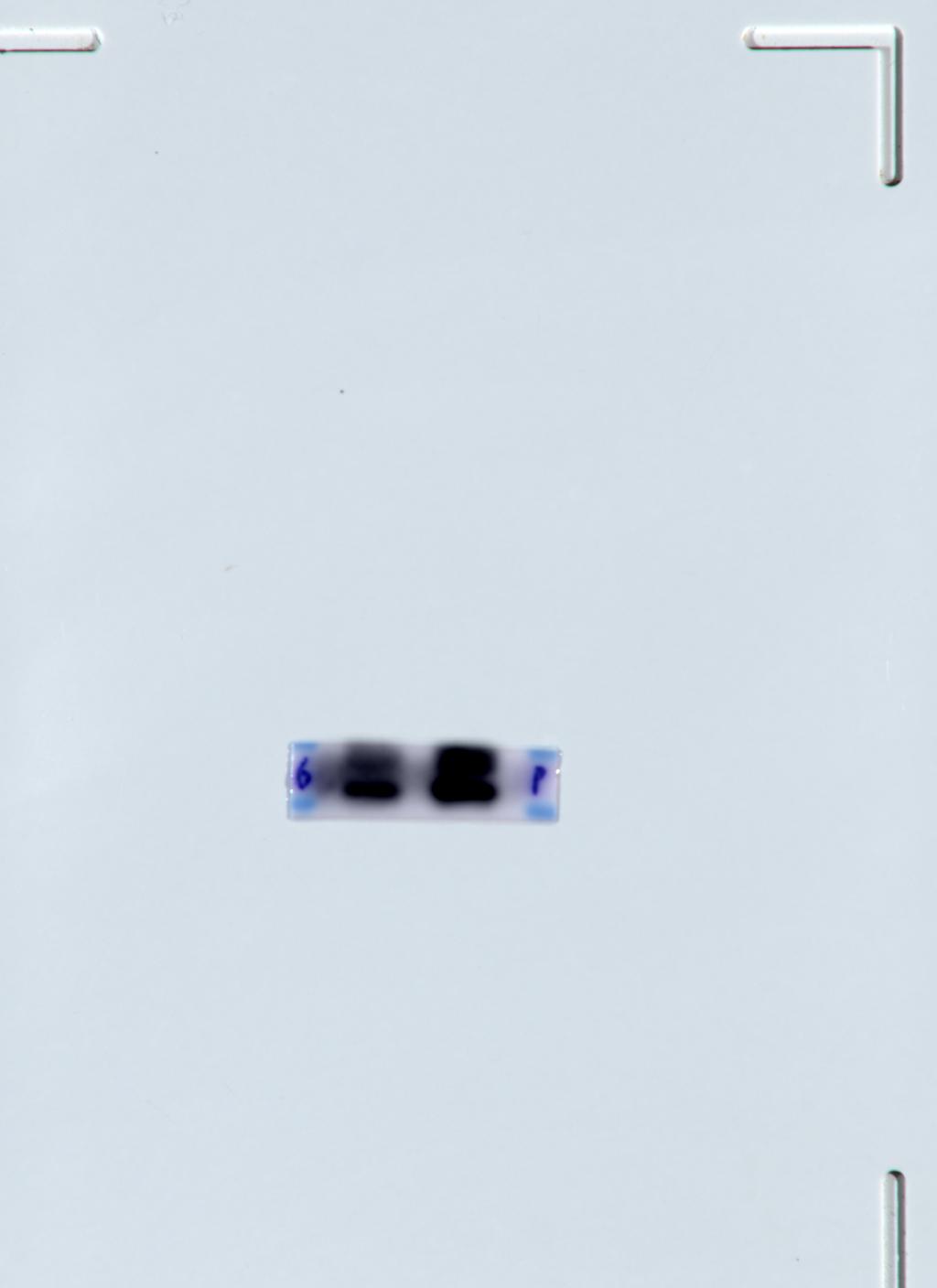

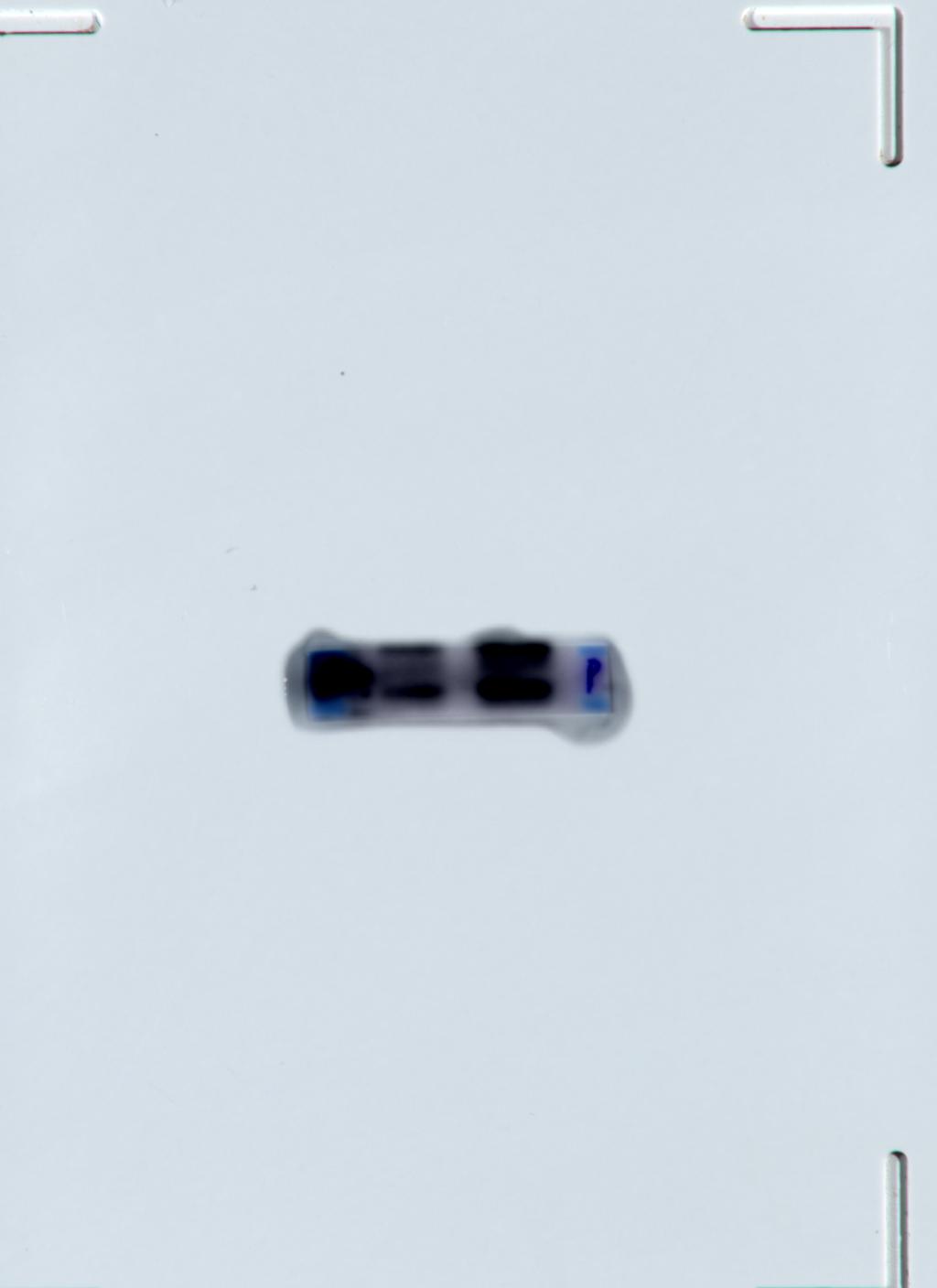
 35kDa

130kDa

TLR4
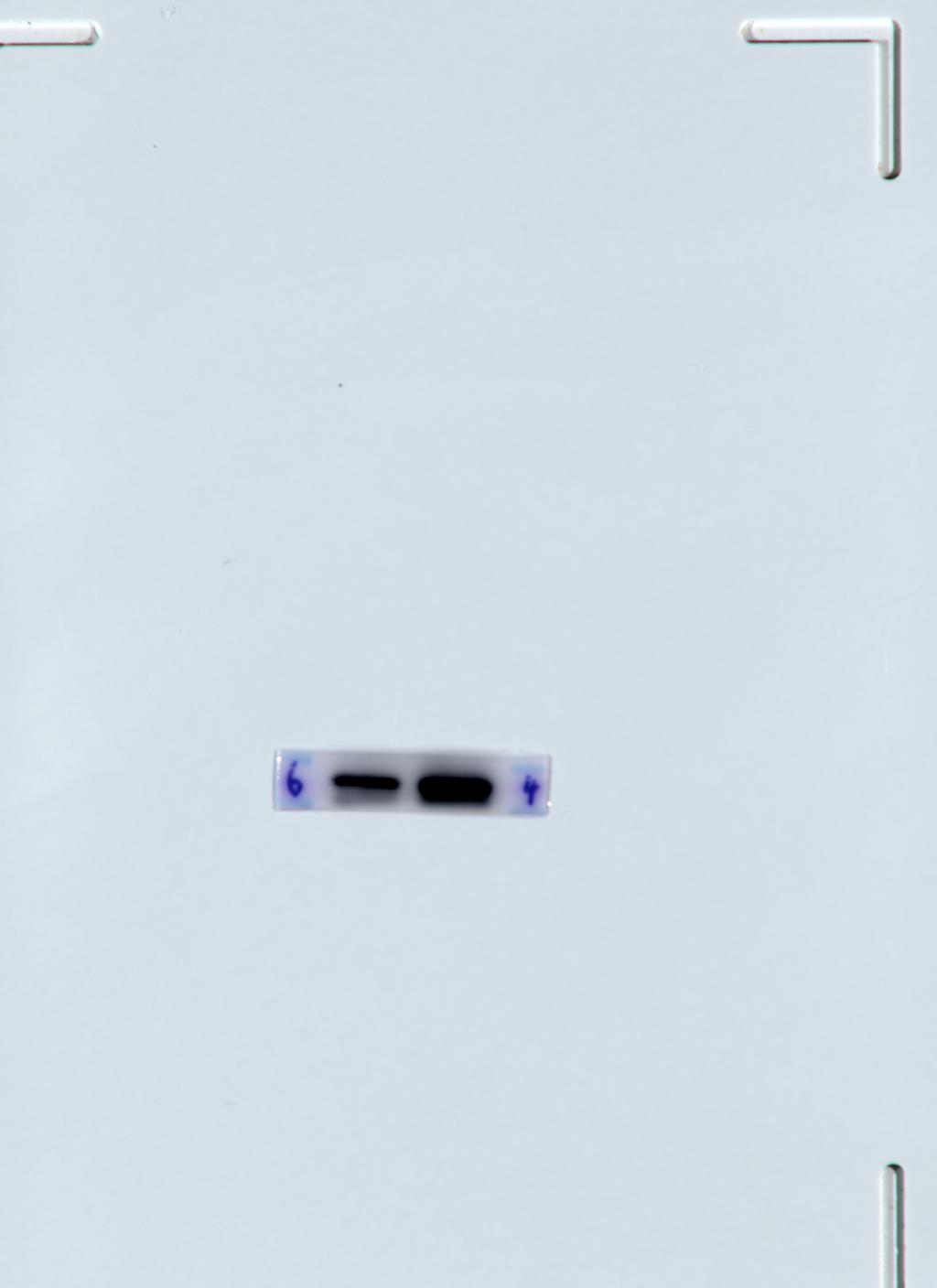

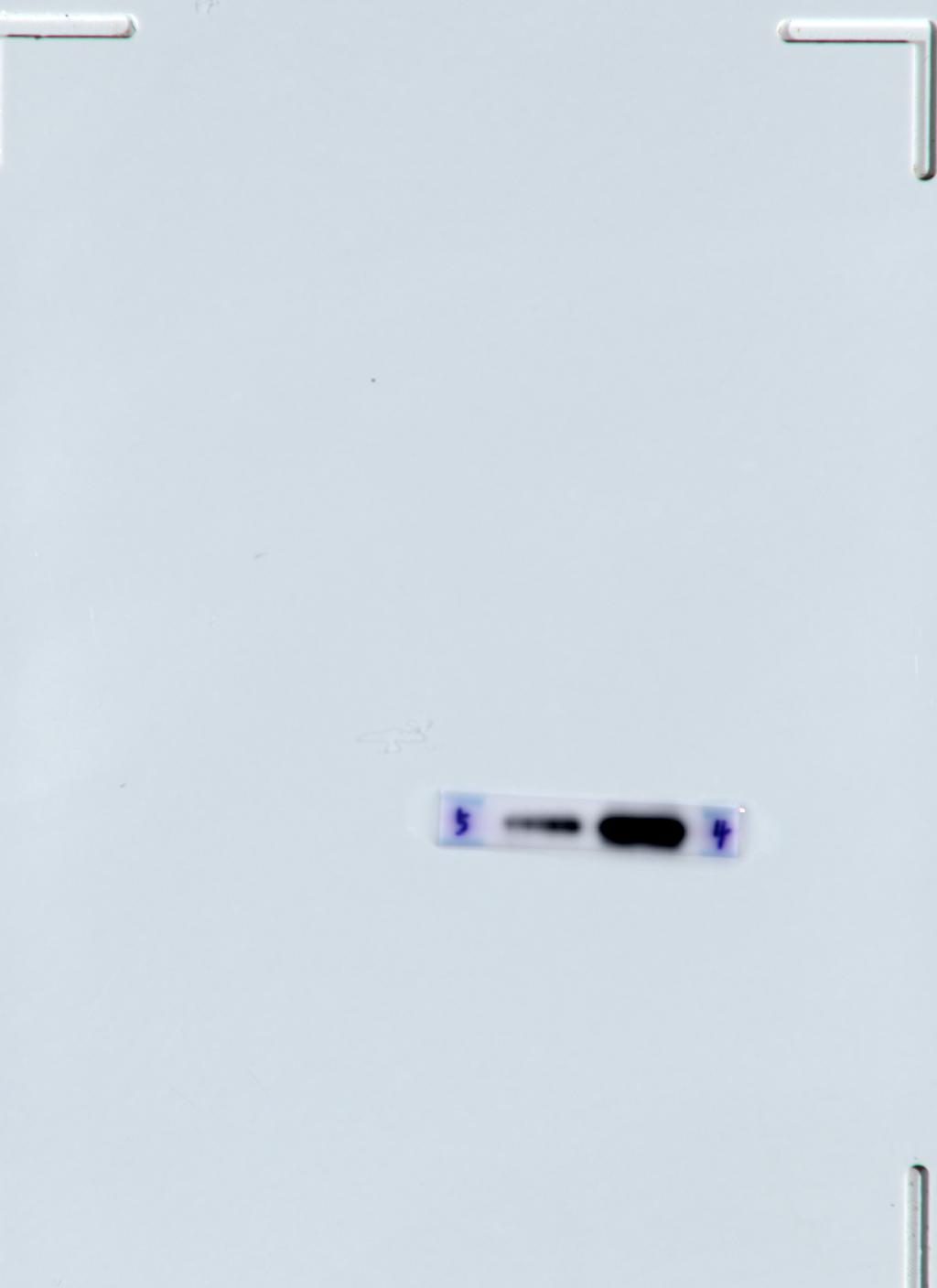
 100kDa

70kDa

TRAF6
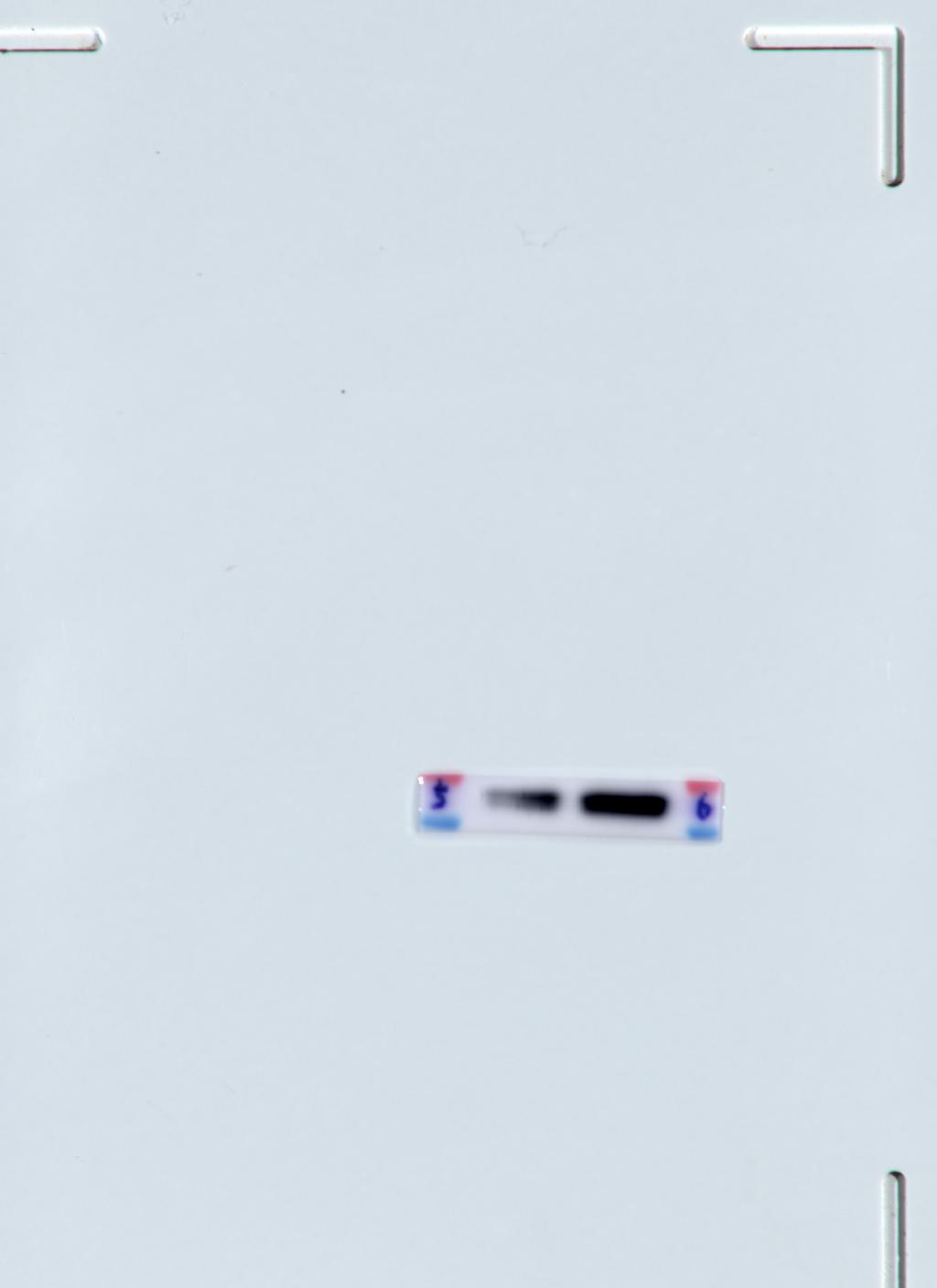

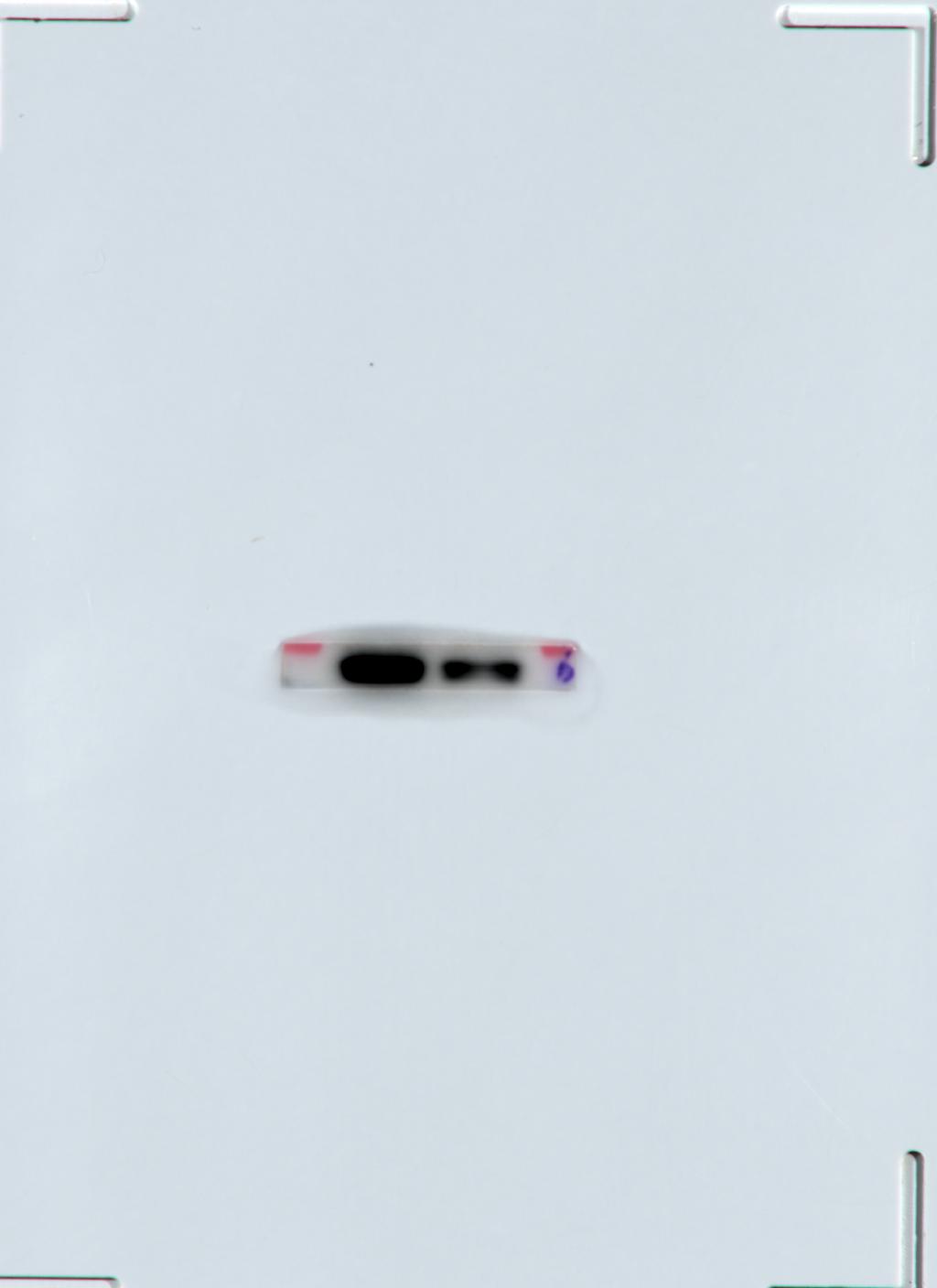
 55kDa

55kDa

TRIM59
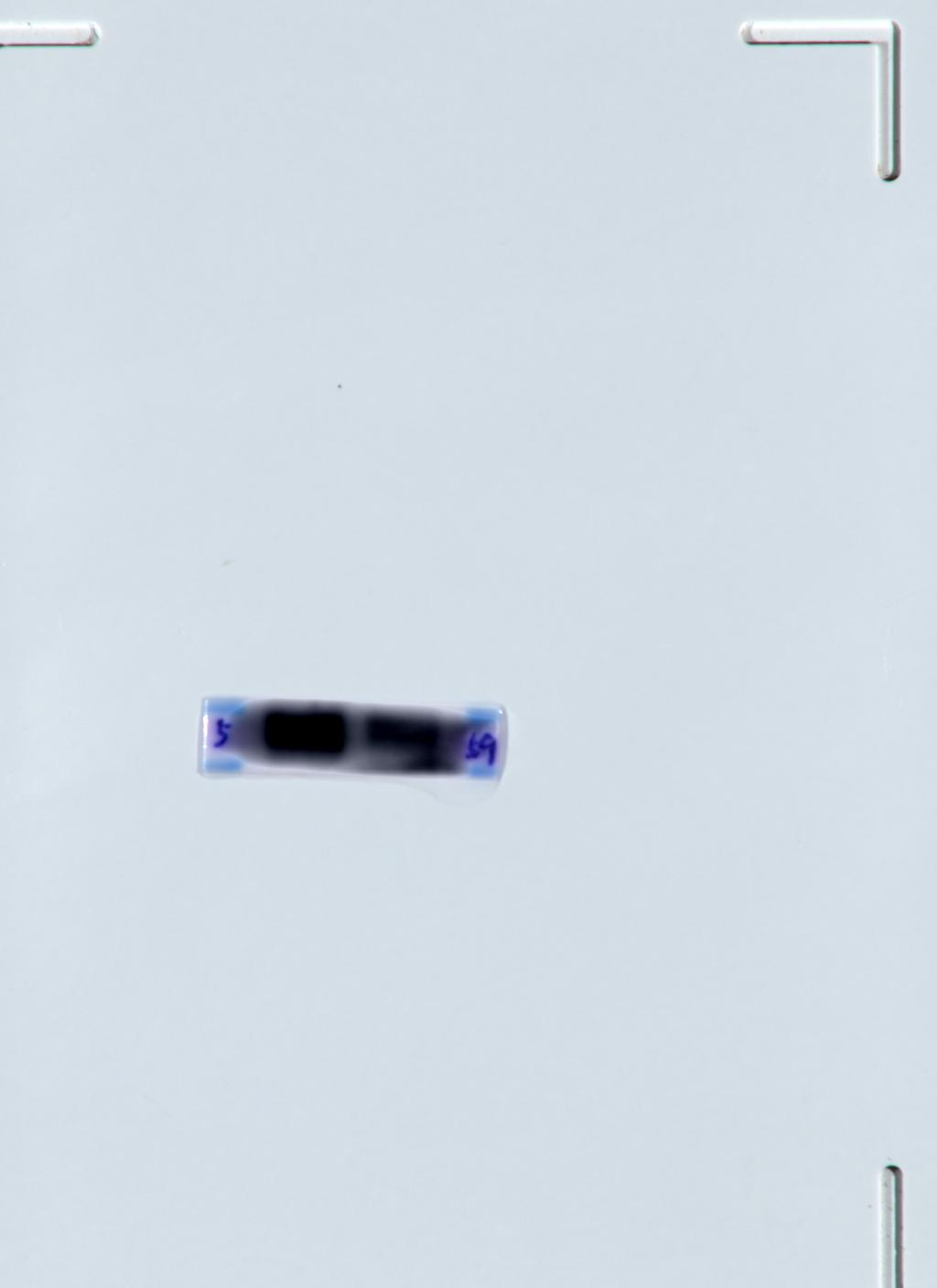

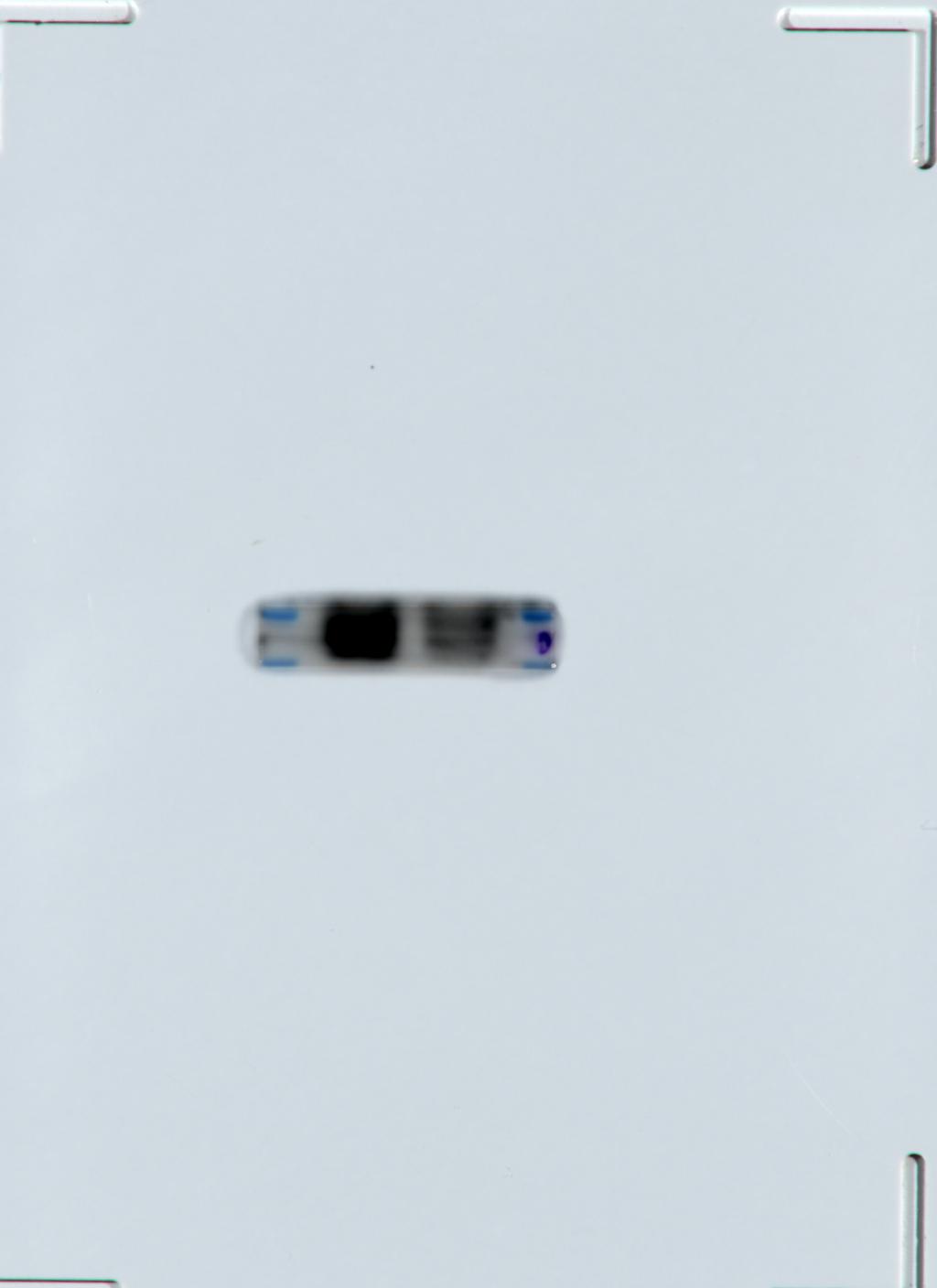
 40kDa

55kDa

POU5F1
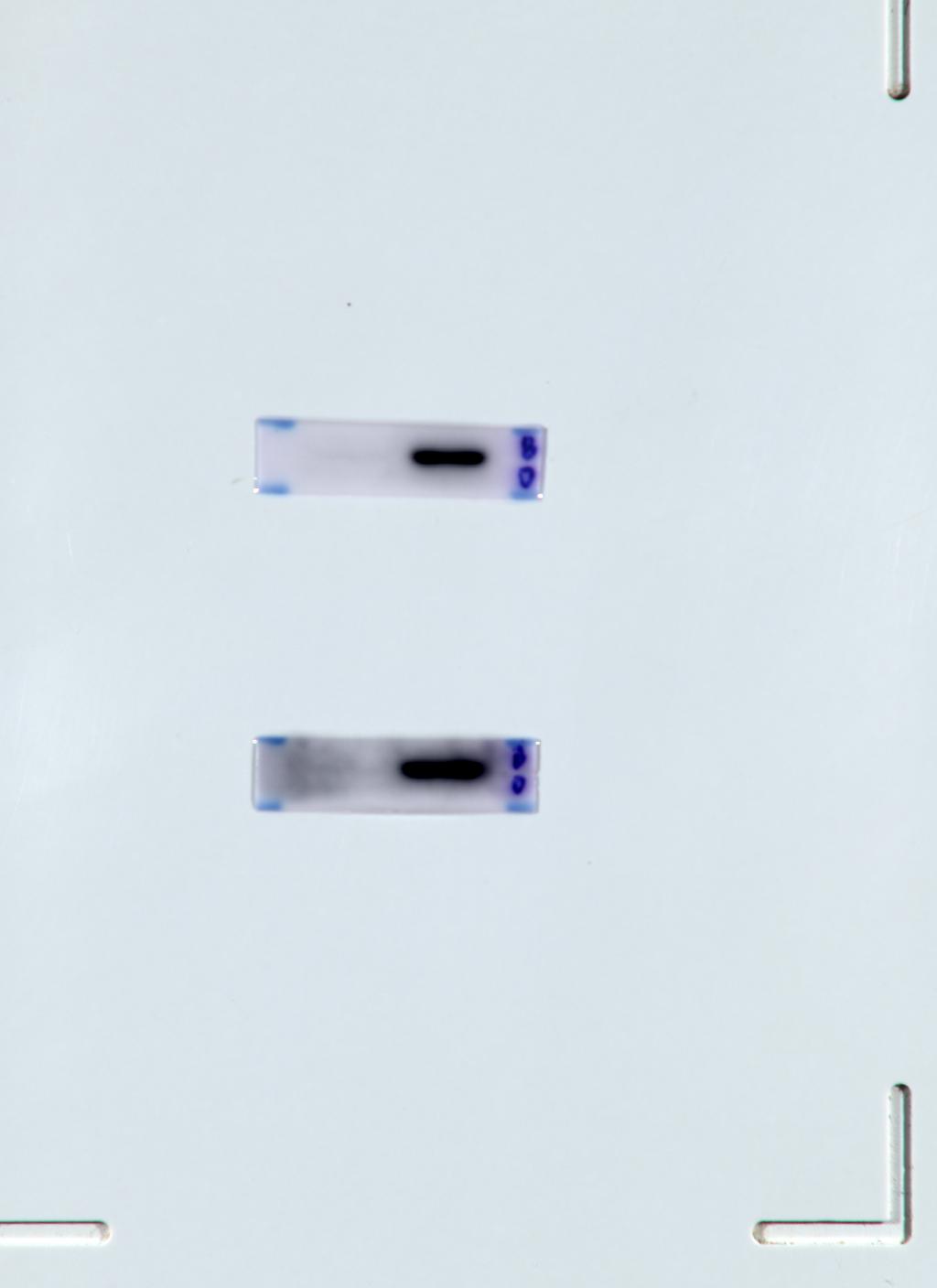

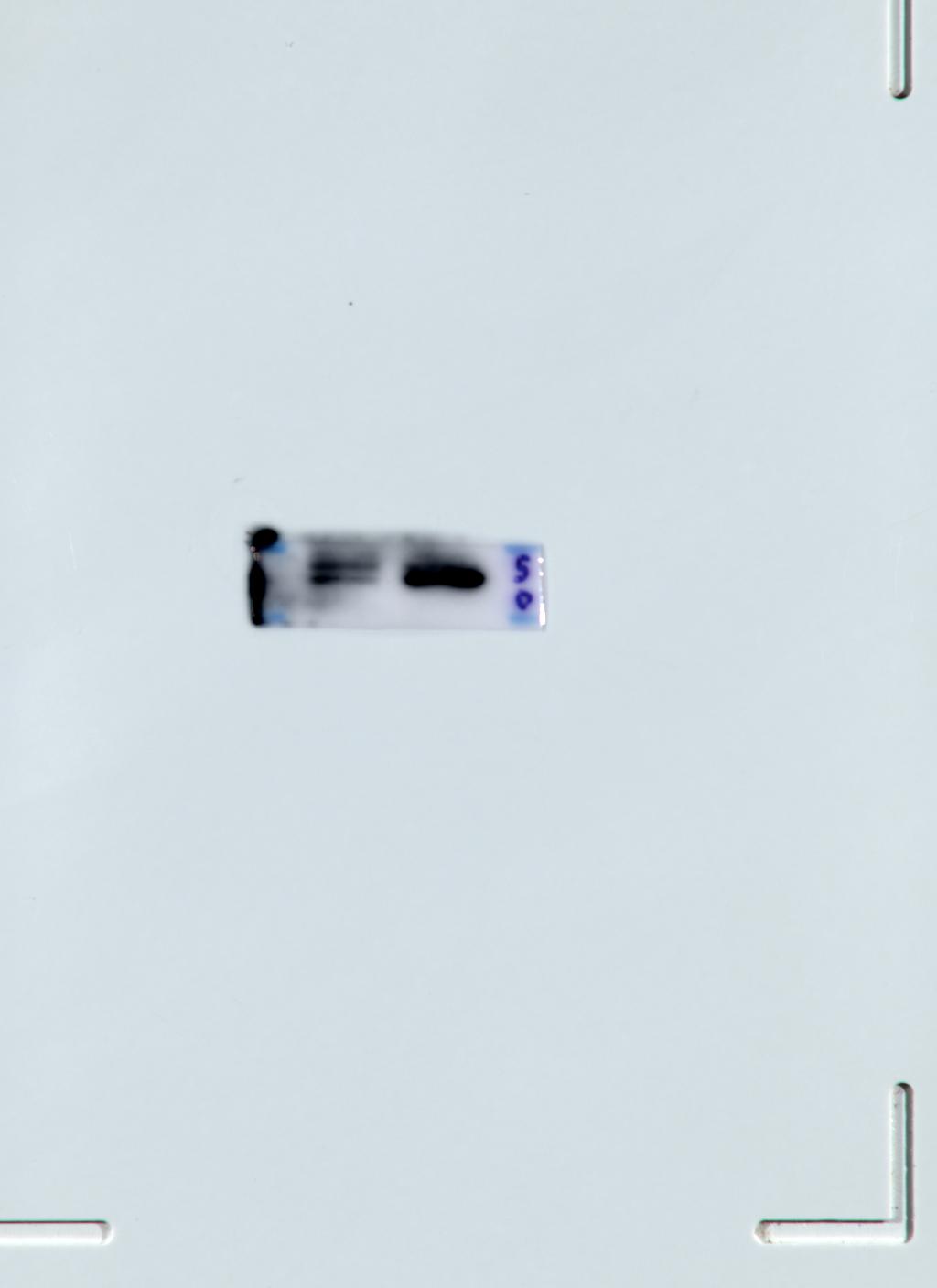
 40kDa

40kDa

GAPDH
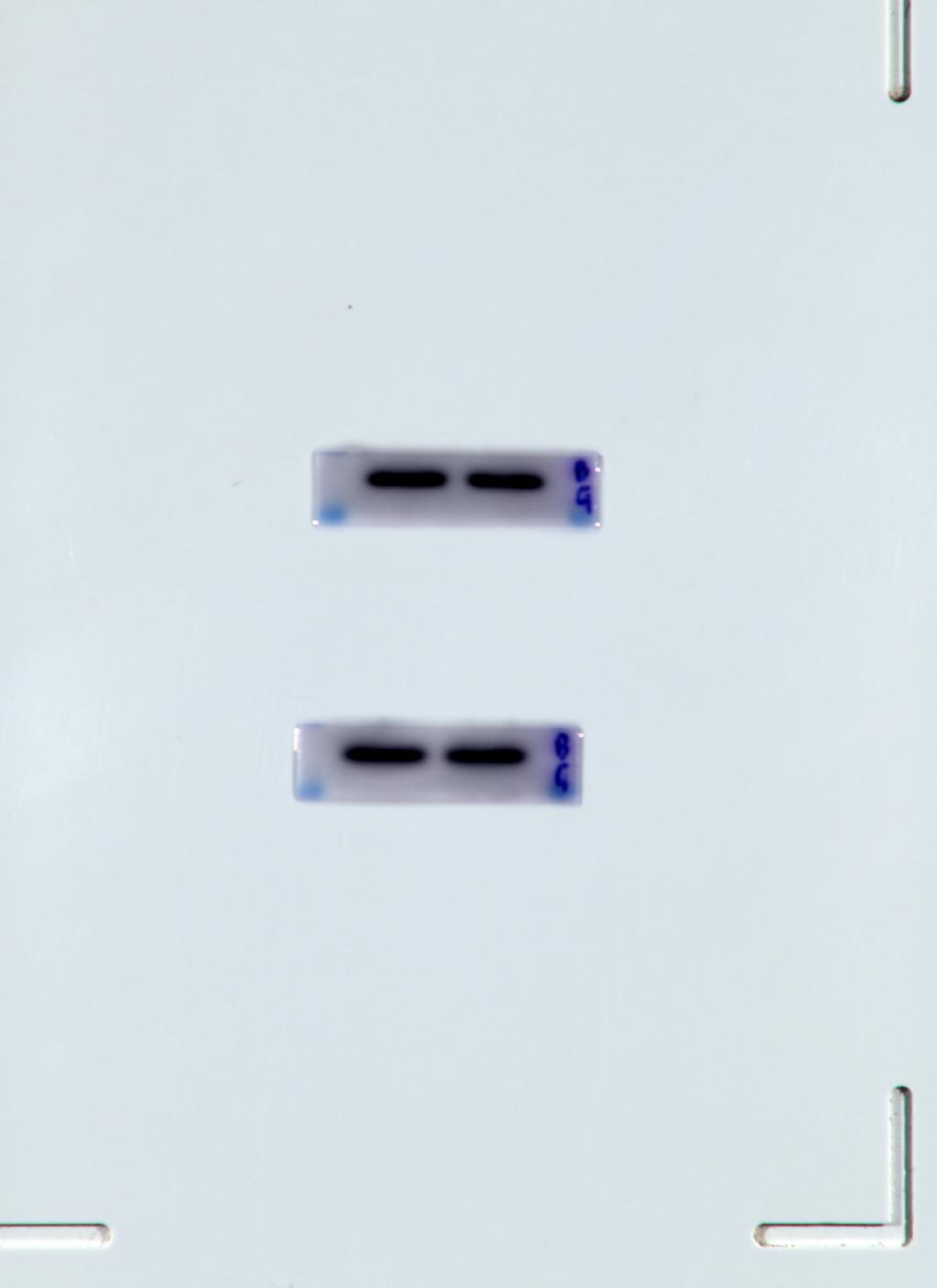

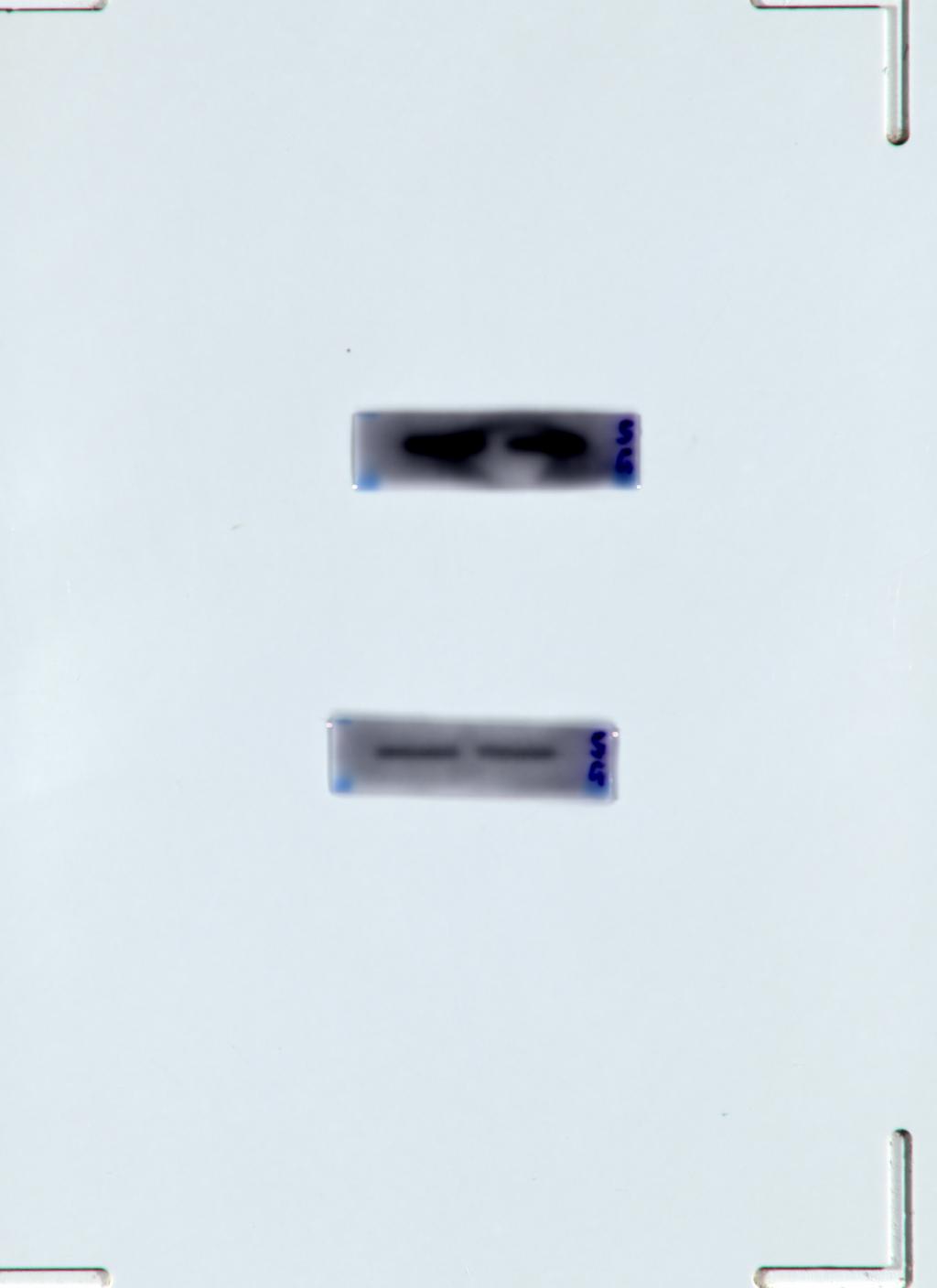
 35kDa

Figure 5

70kDa

TRAF6
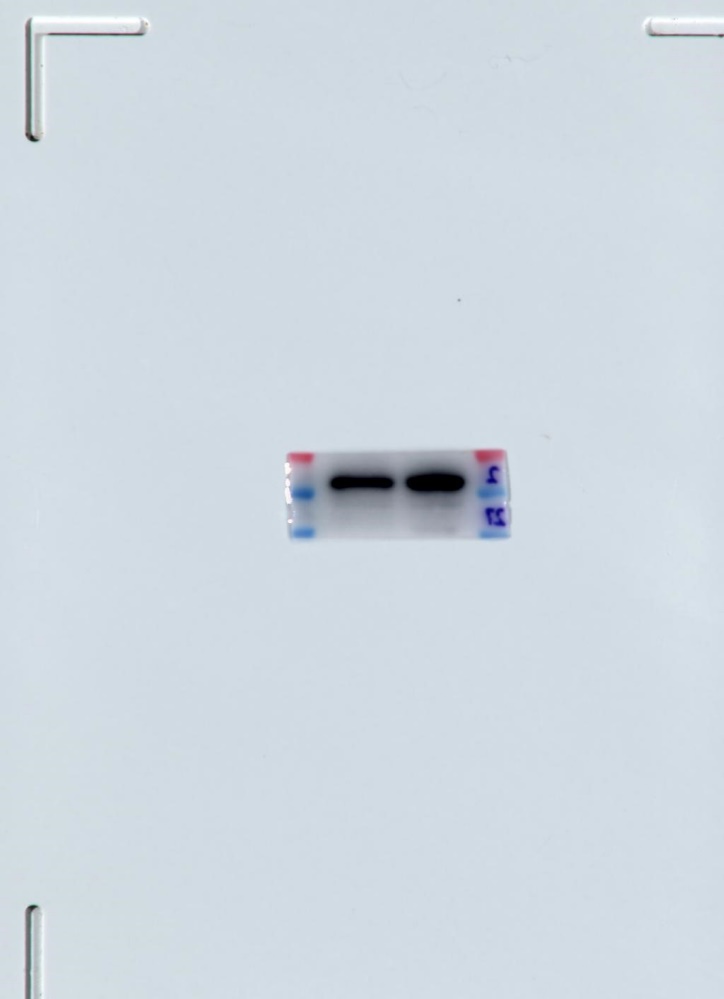

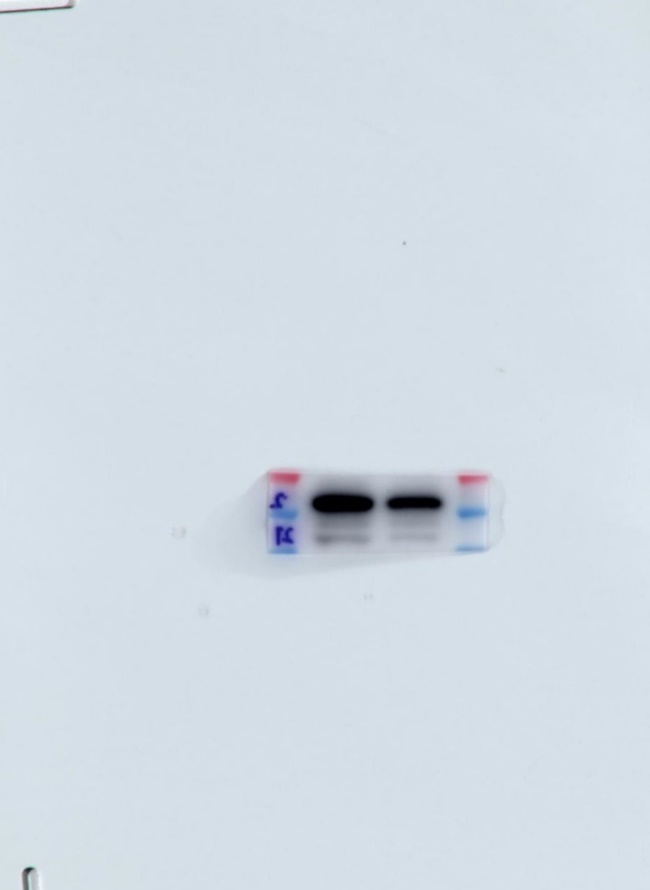
 55kDa

55kDa

TRIM59
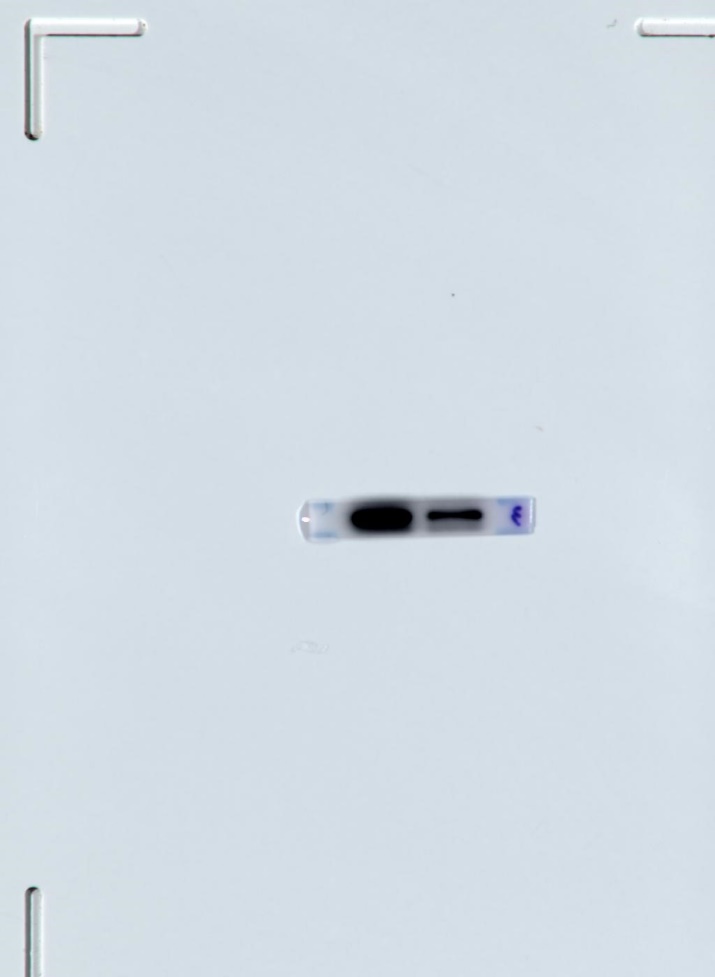

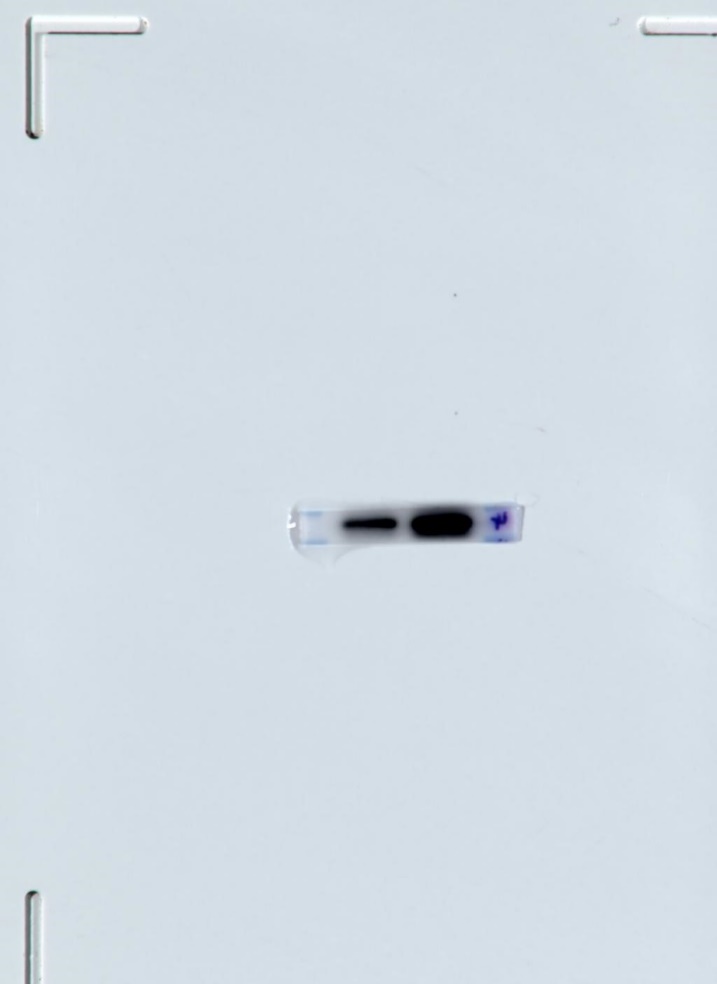
 40kDa

40kDa

GAPDH
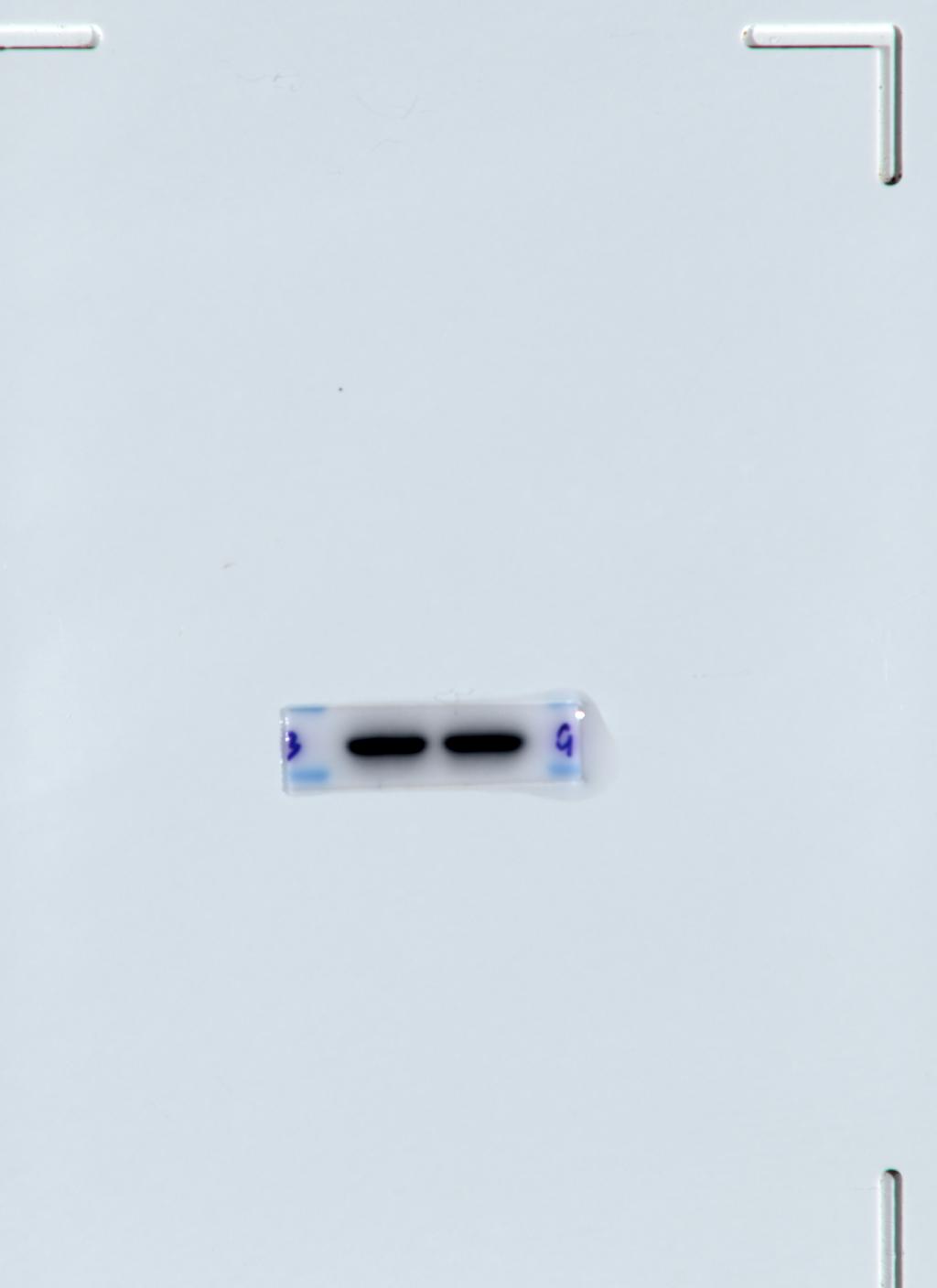

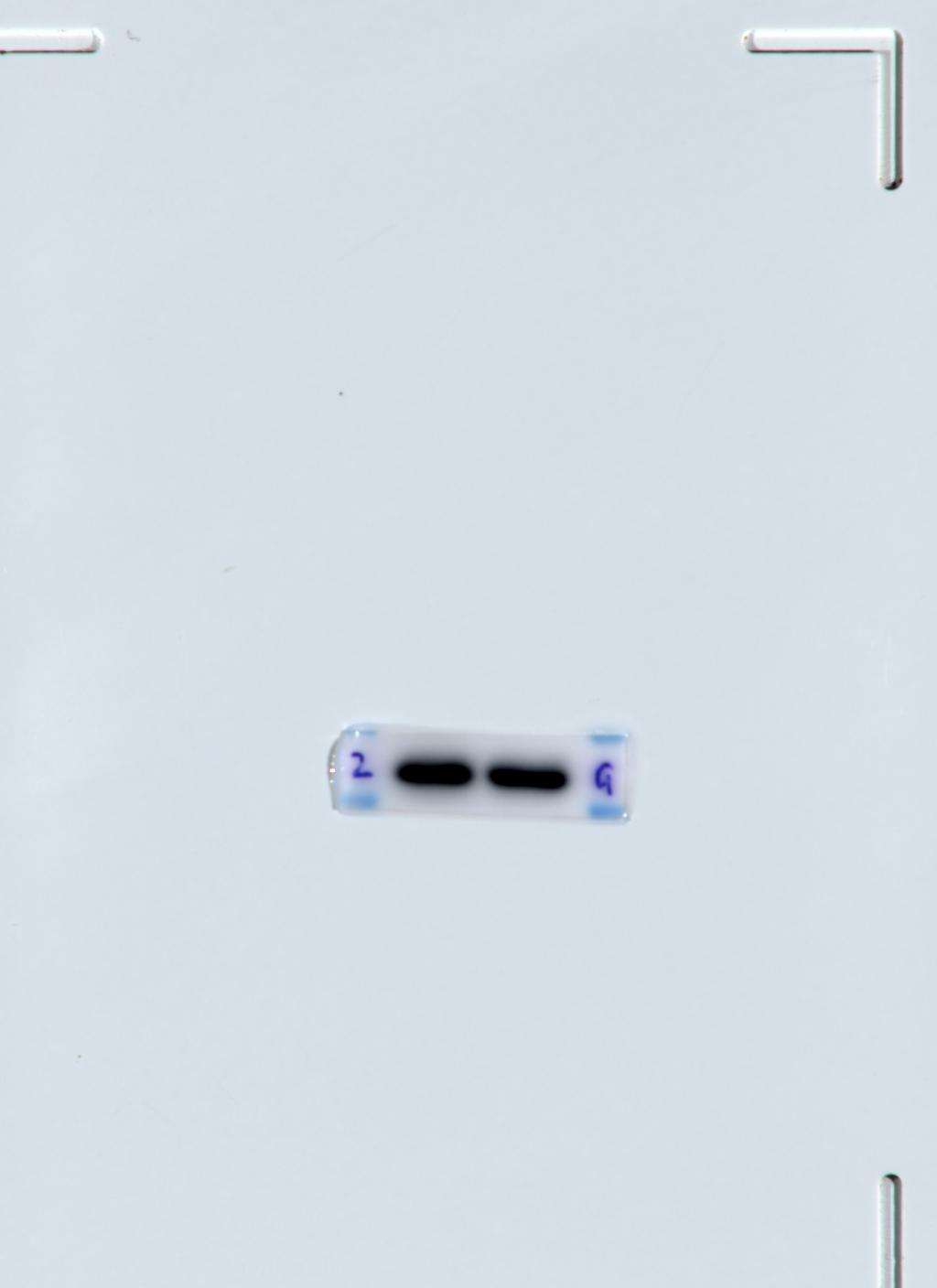
 35kDa

70kDa

TRAF6
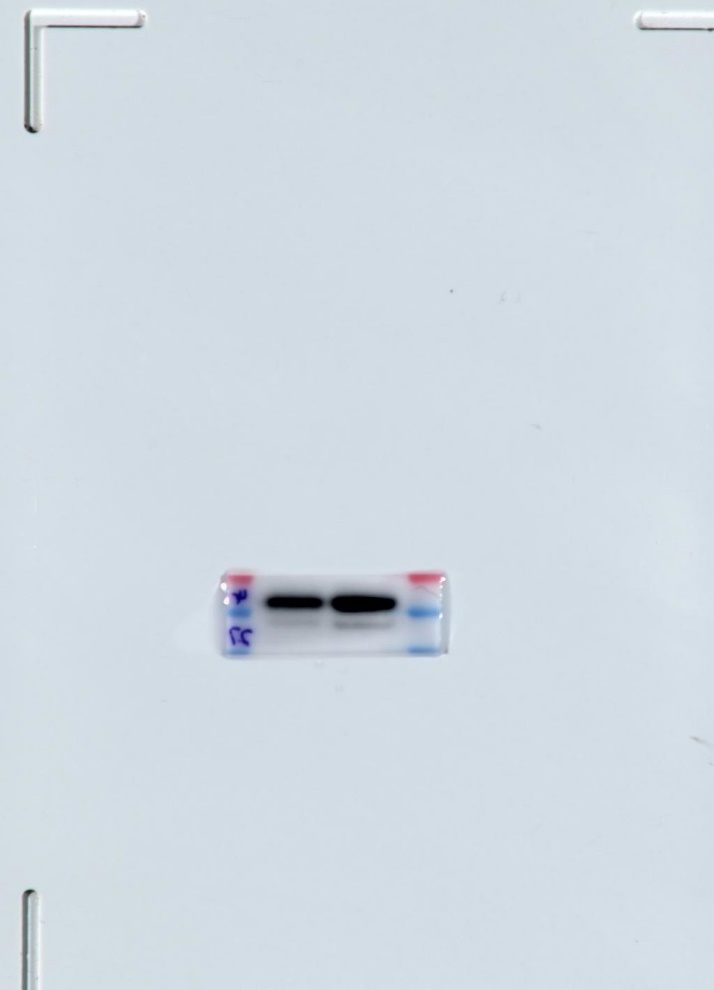

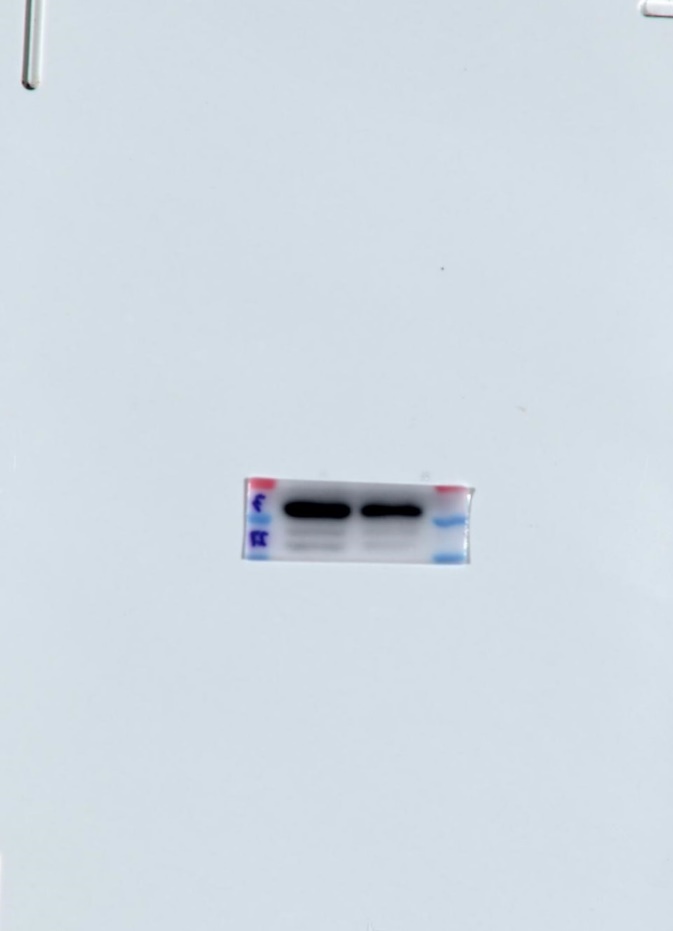
 55kDa

55kDa

TRIM59
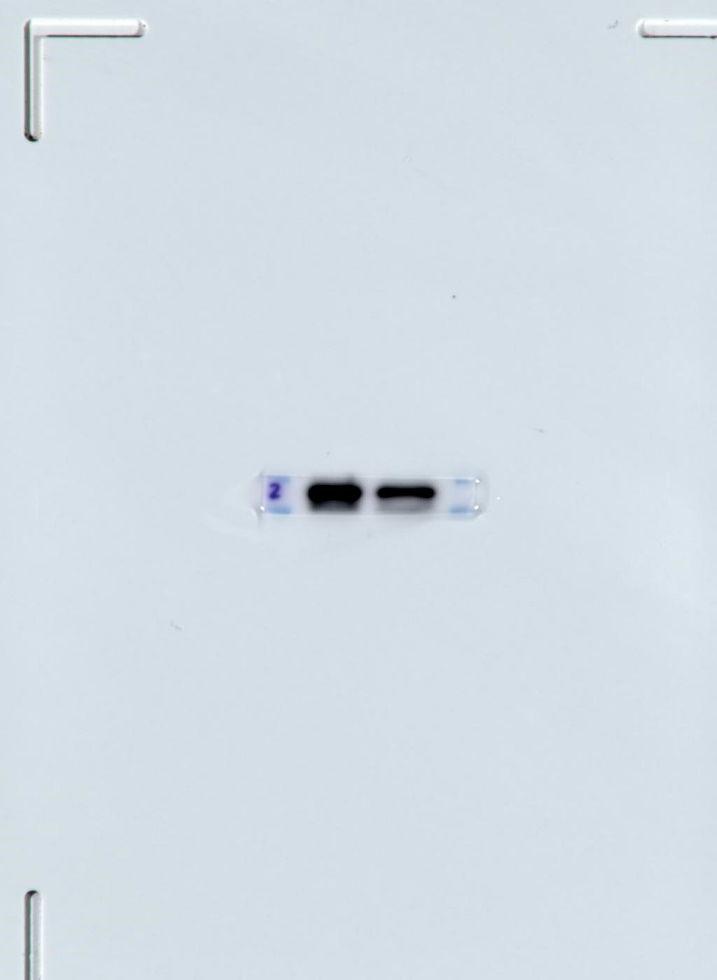

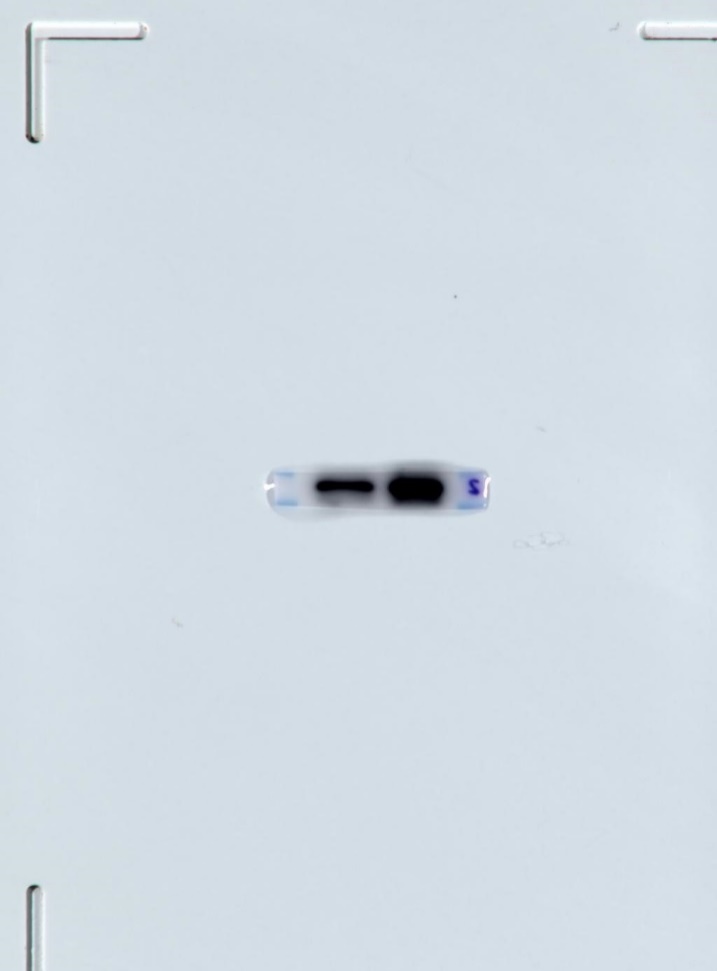
40kDa

40kDa

GAPDH
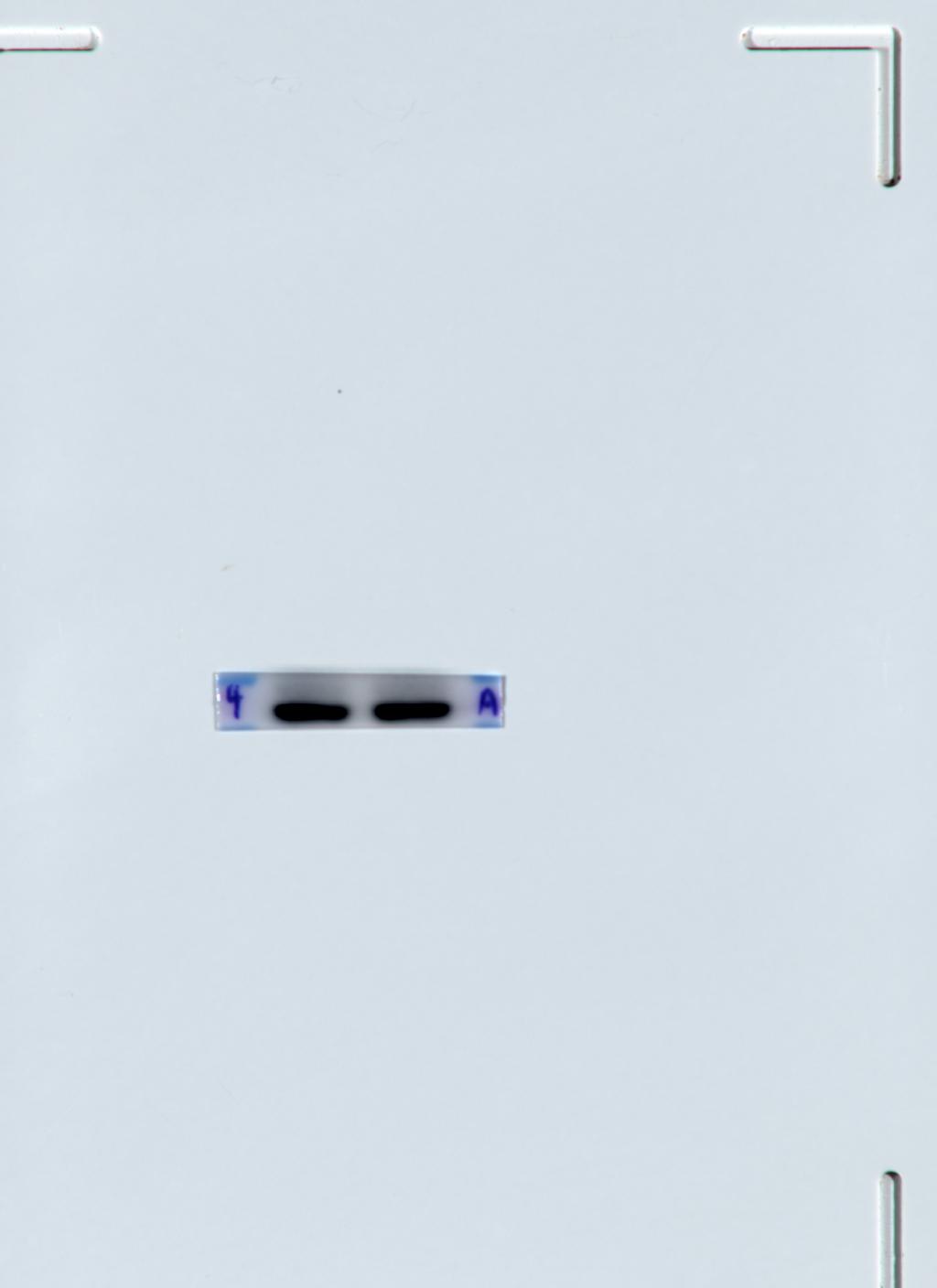

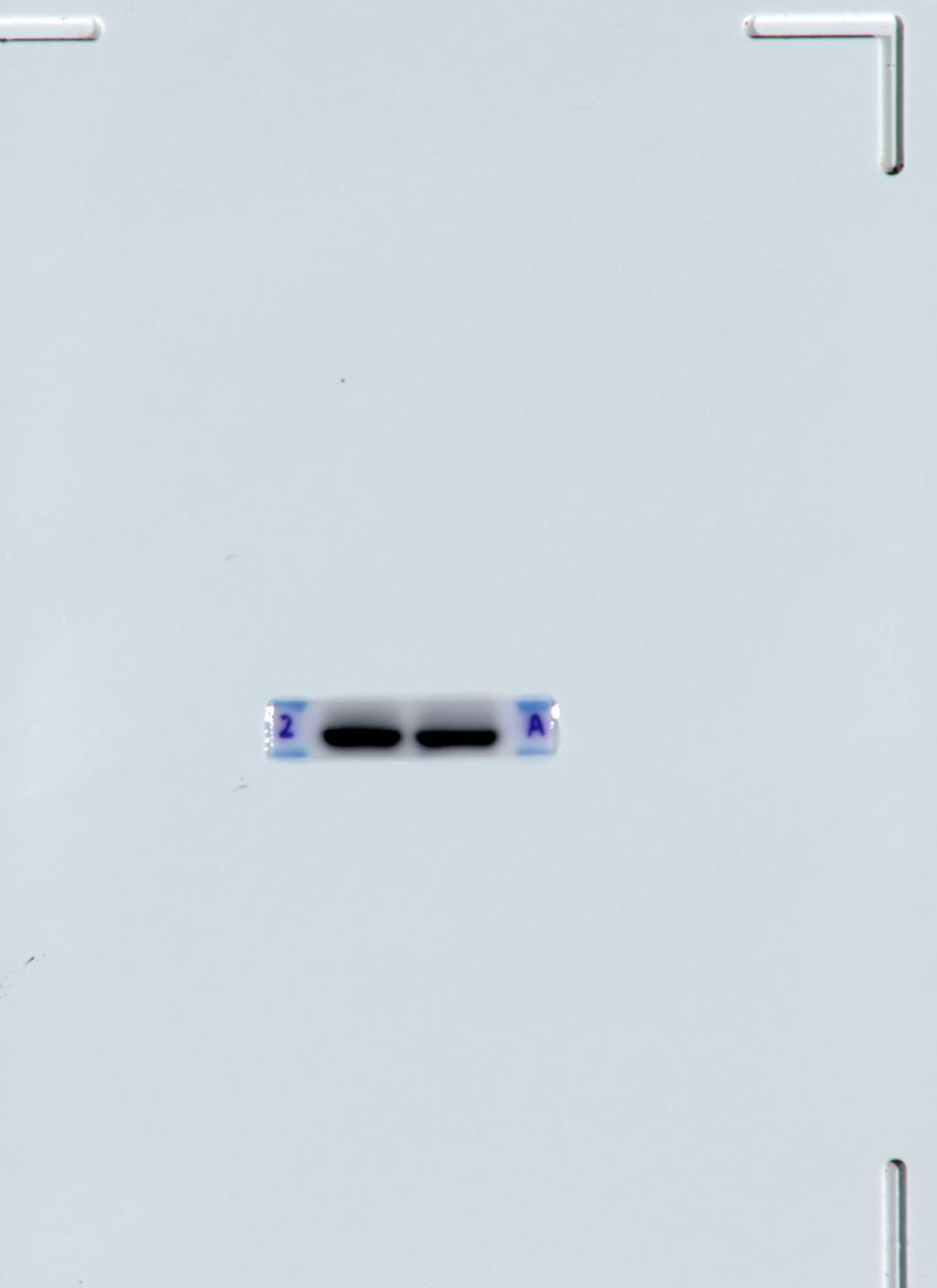
 35kDa

70kDa

TRAF6
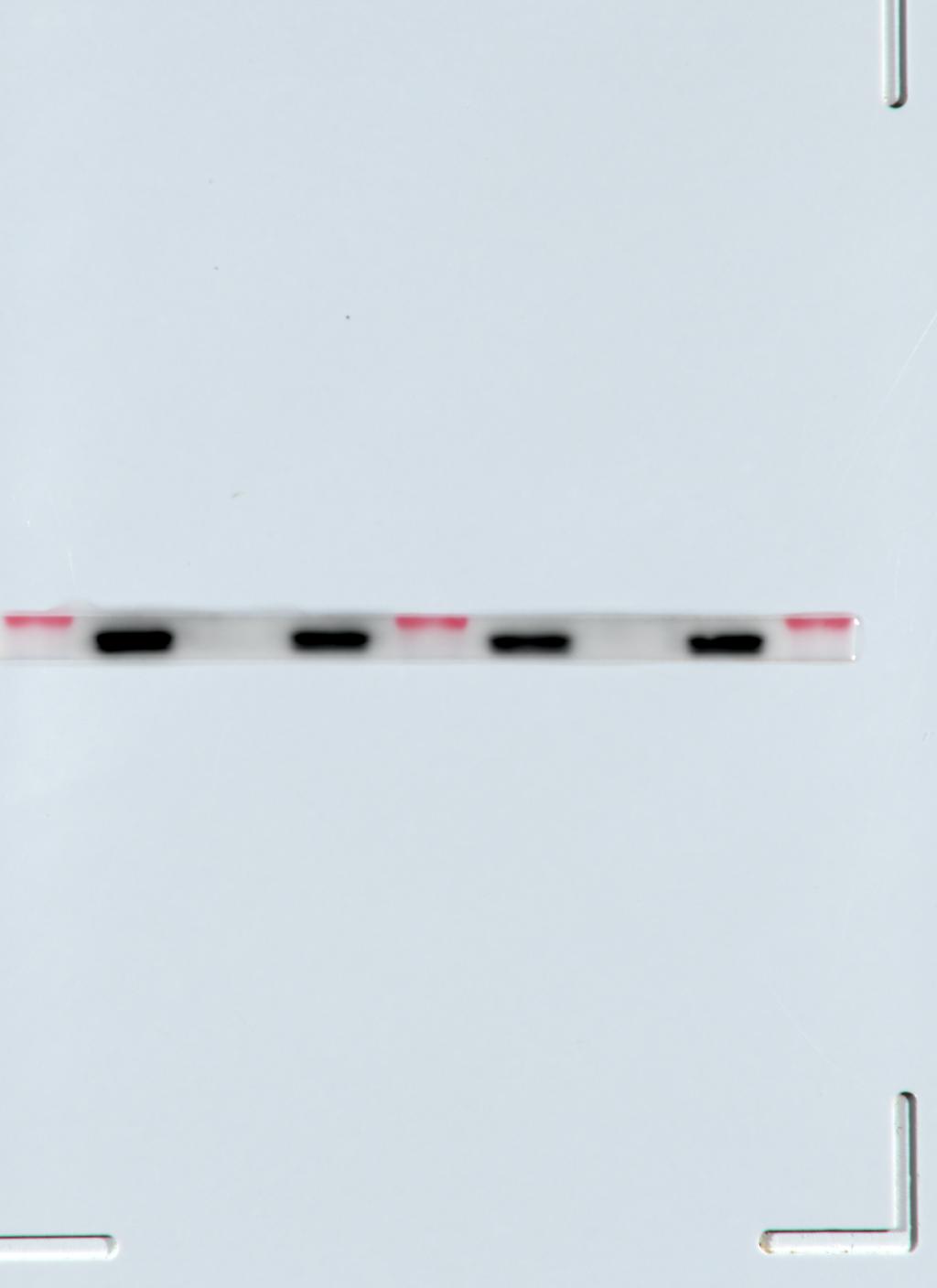
 55kDa

55kDa

TRIM59
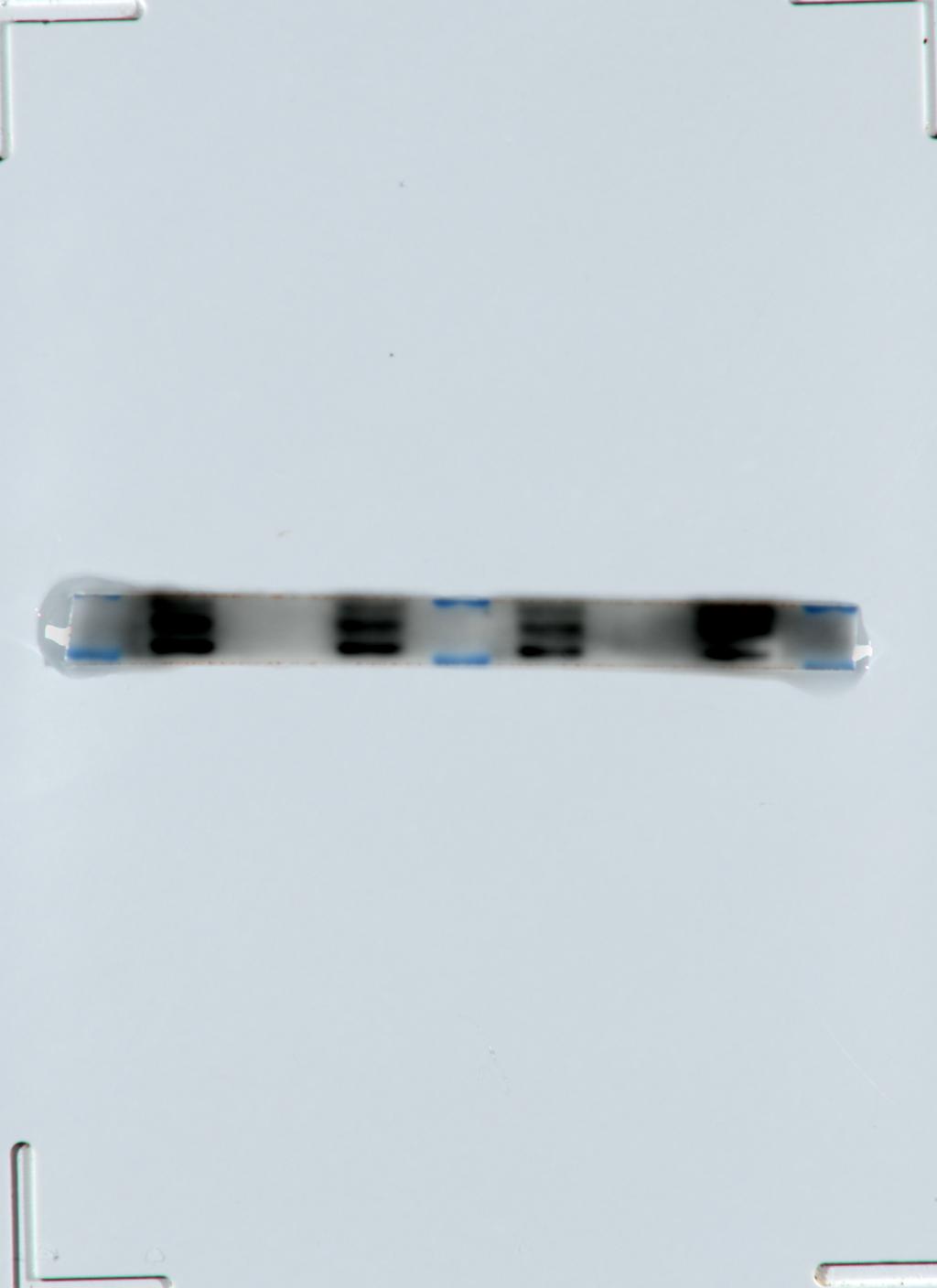
 40kDa

70kDa

TRAF6
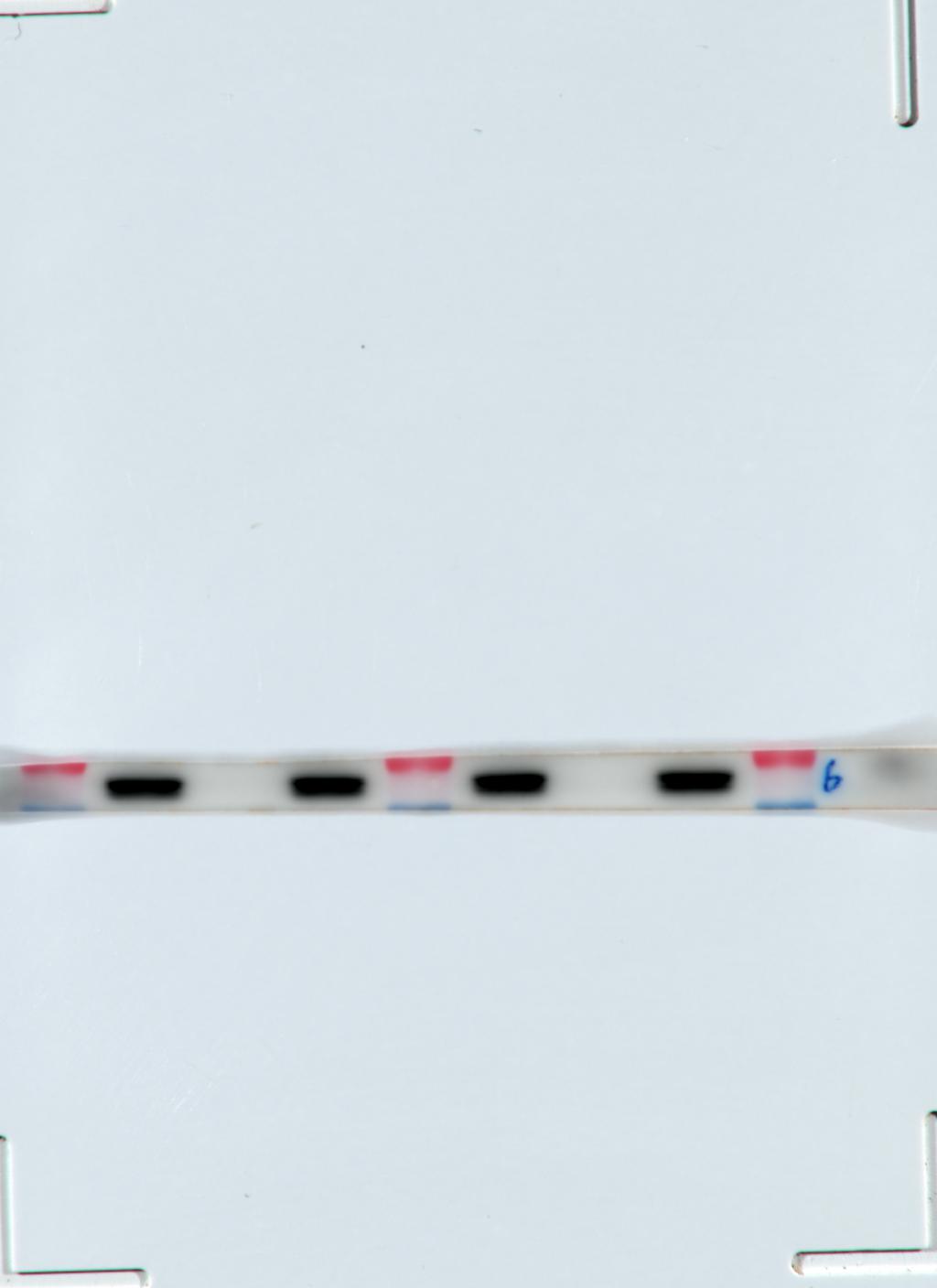
 55kDa

55kDa

TRIM59
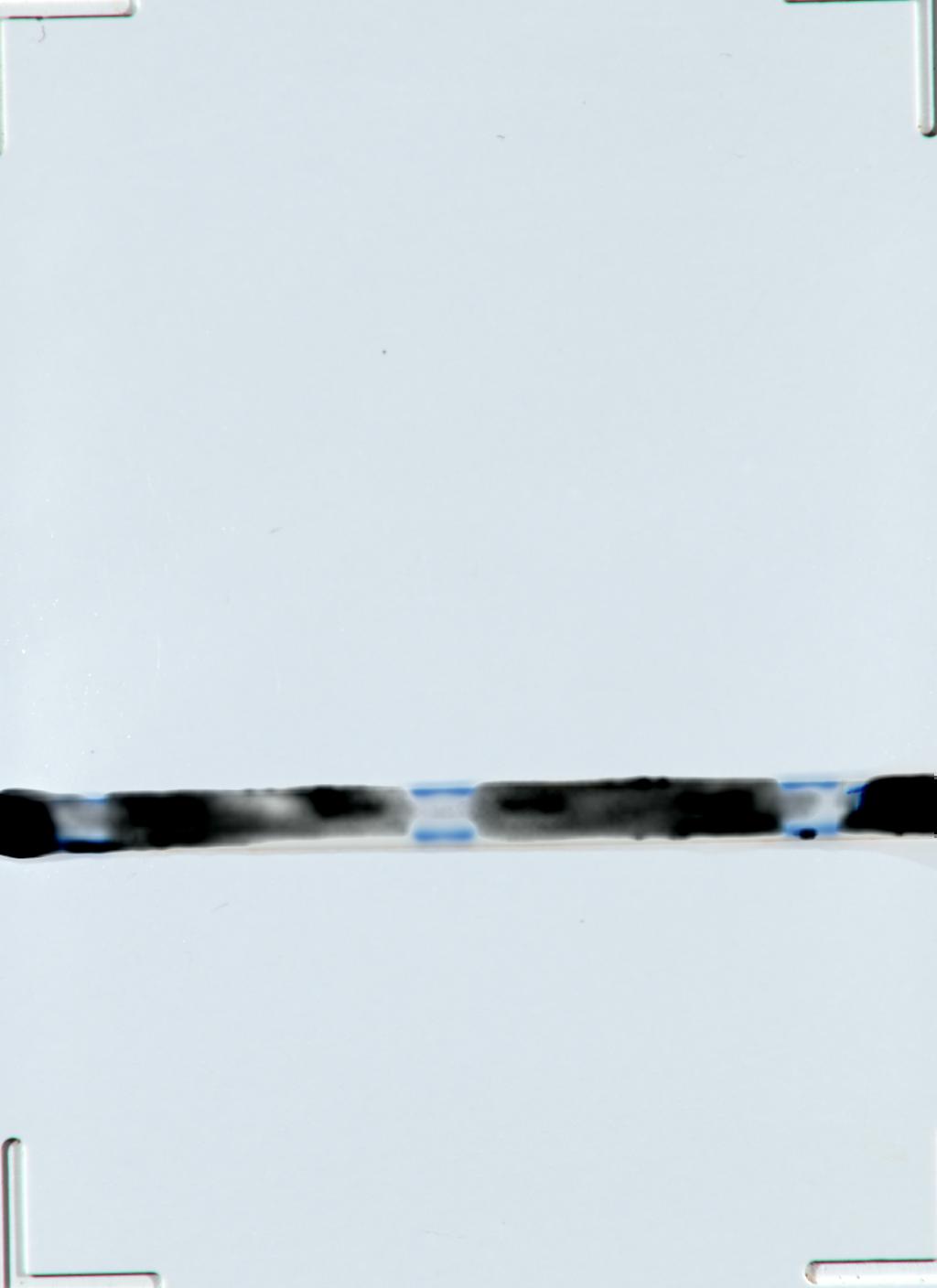
 40kDa

70kDa

TRAF6
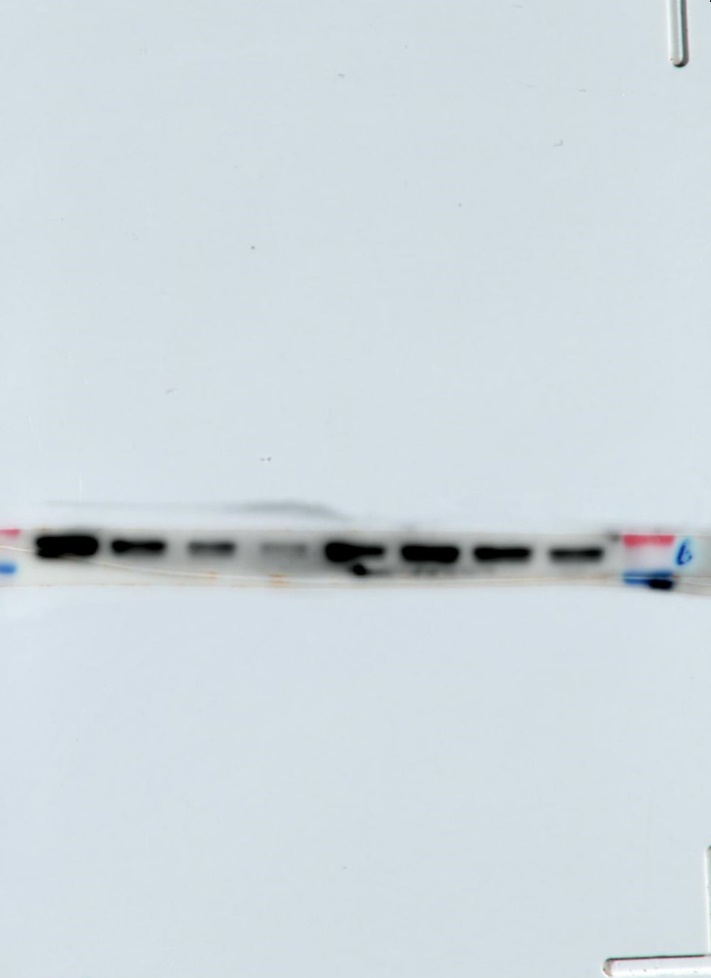
 55kDa

55kDa

TRIM59
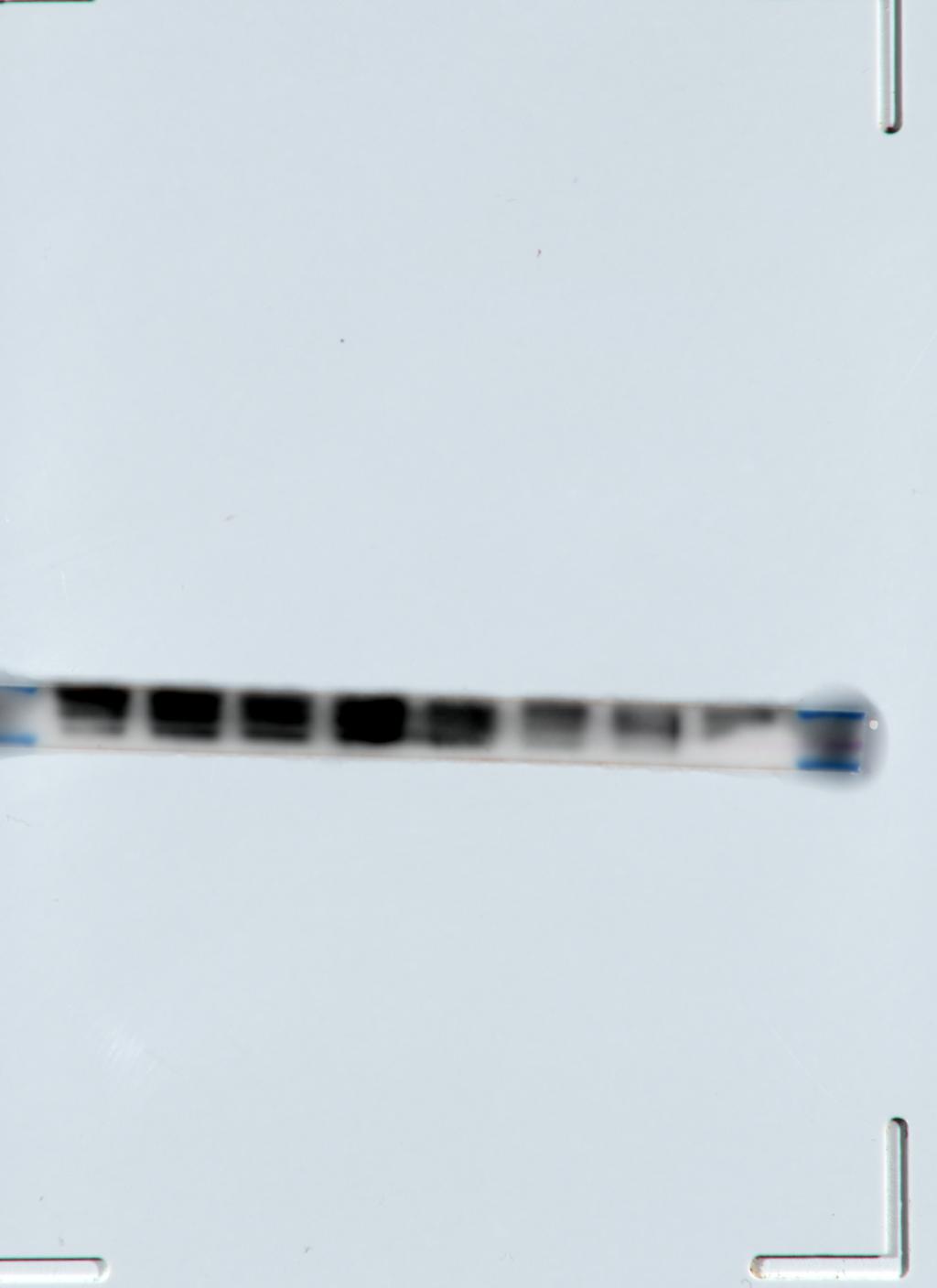
 40kDa

40kDa

GAPDH
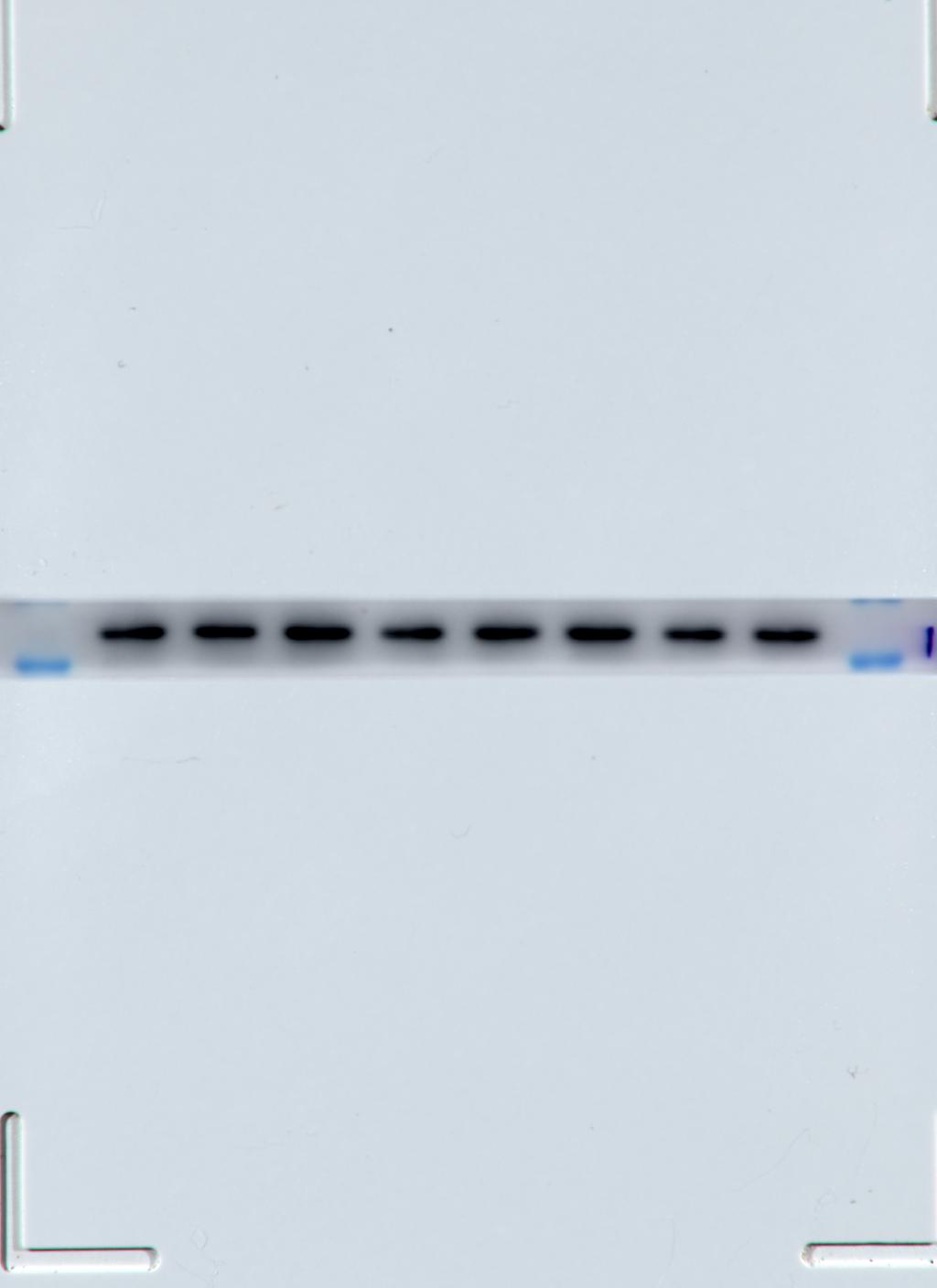
 35kDa

70kDa

TRAF6
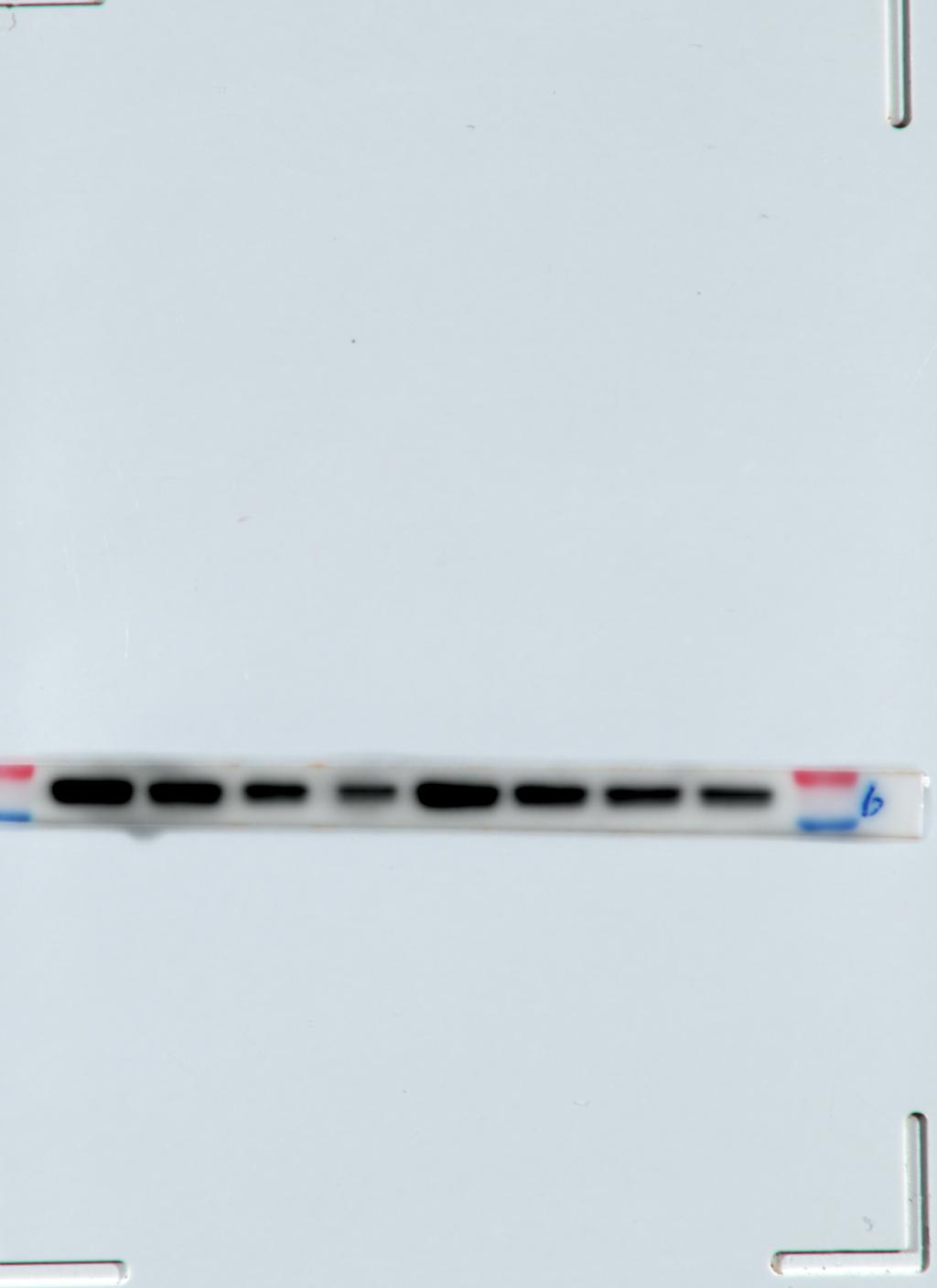
 55kDa

55kDa

TRIM59
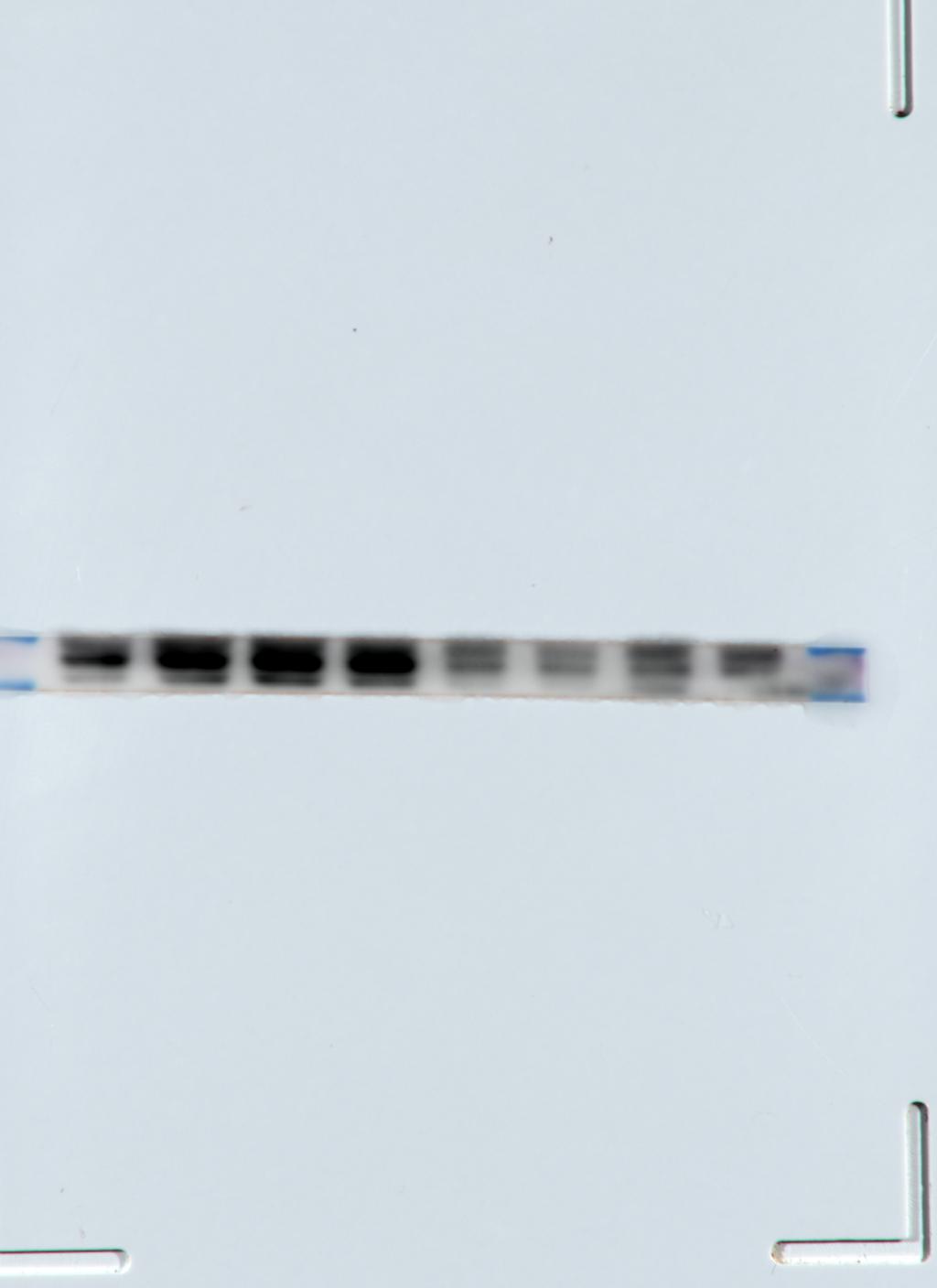
 40kDa

40kDa

GAPDH
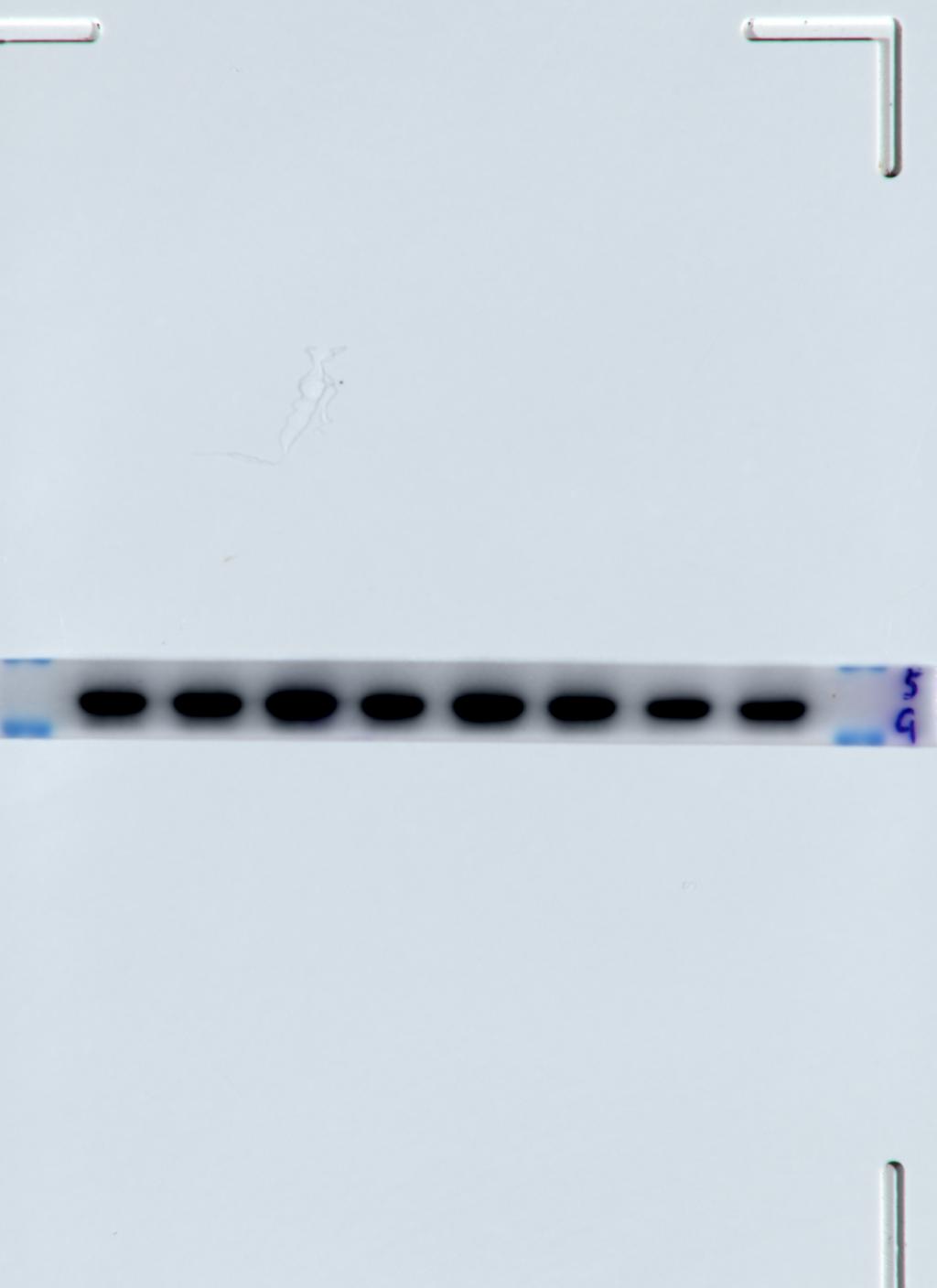
 35kDa

70kDa

TRAF6
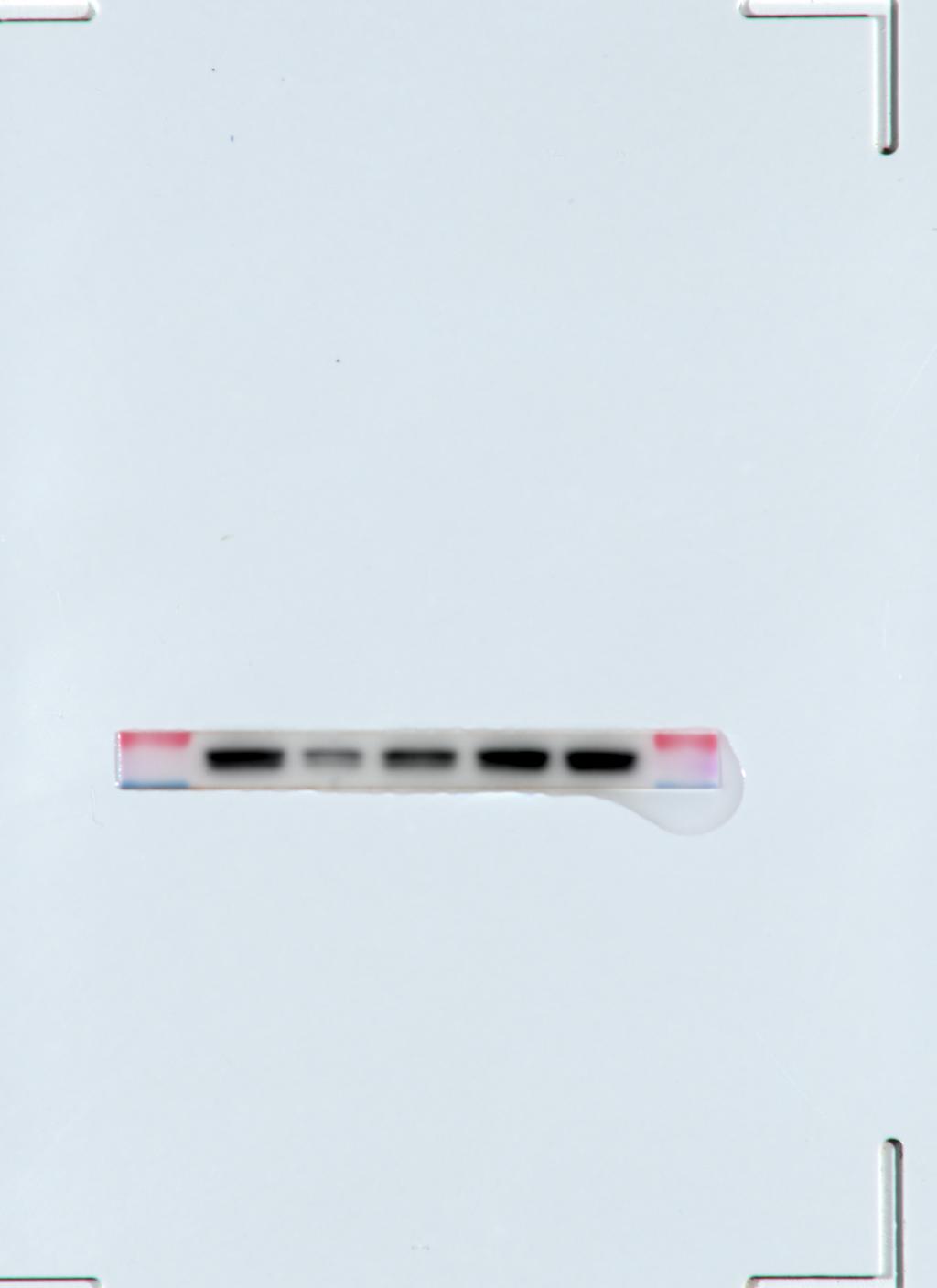
 55kDa

55kDa

TRIM59
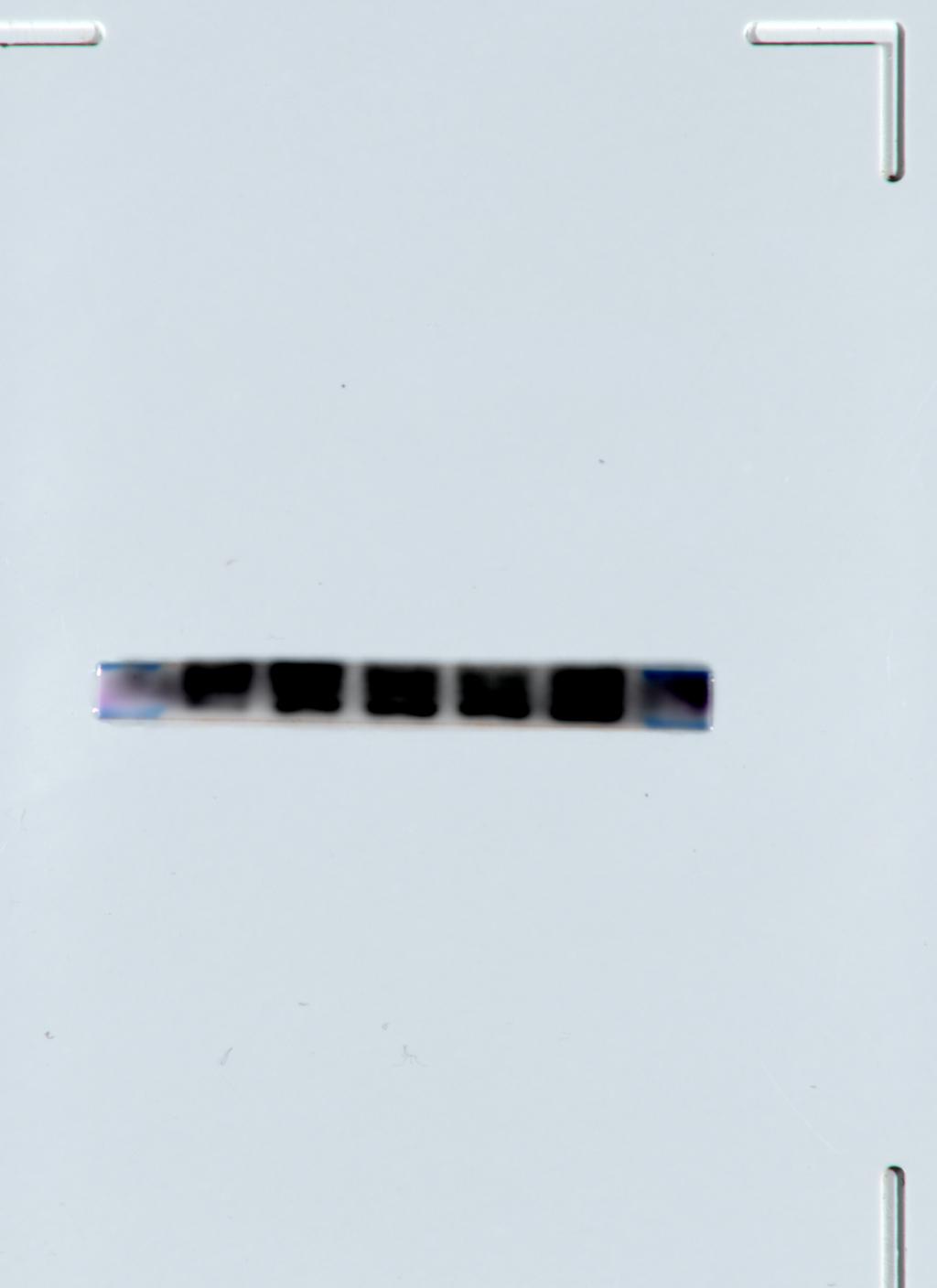
 40kDa

40kDa

GAPDH
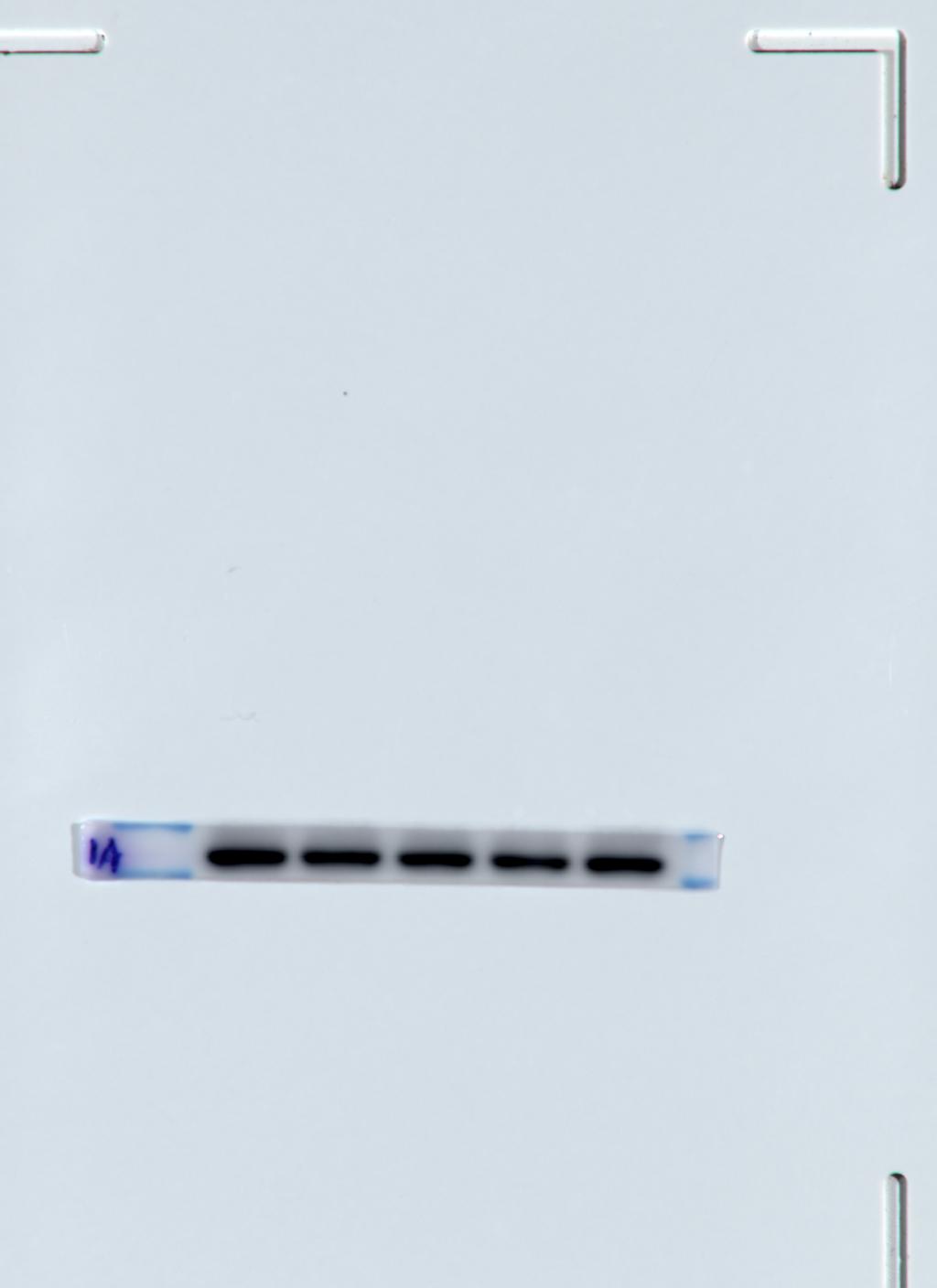
 35kDa

70kDa

TRAF6
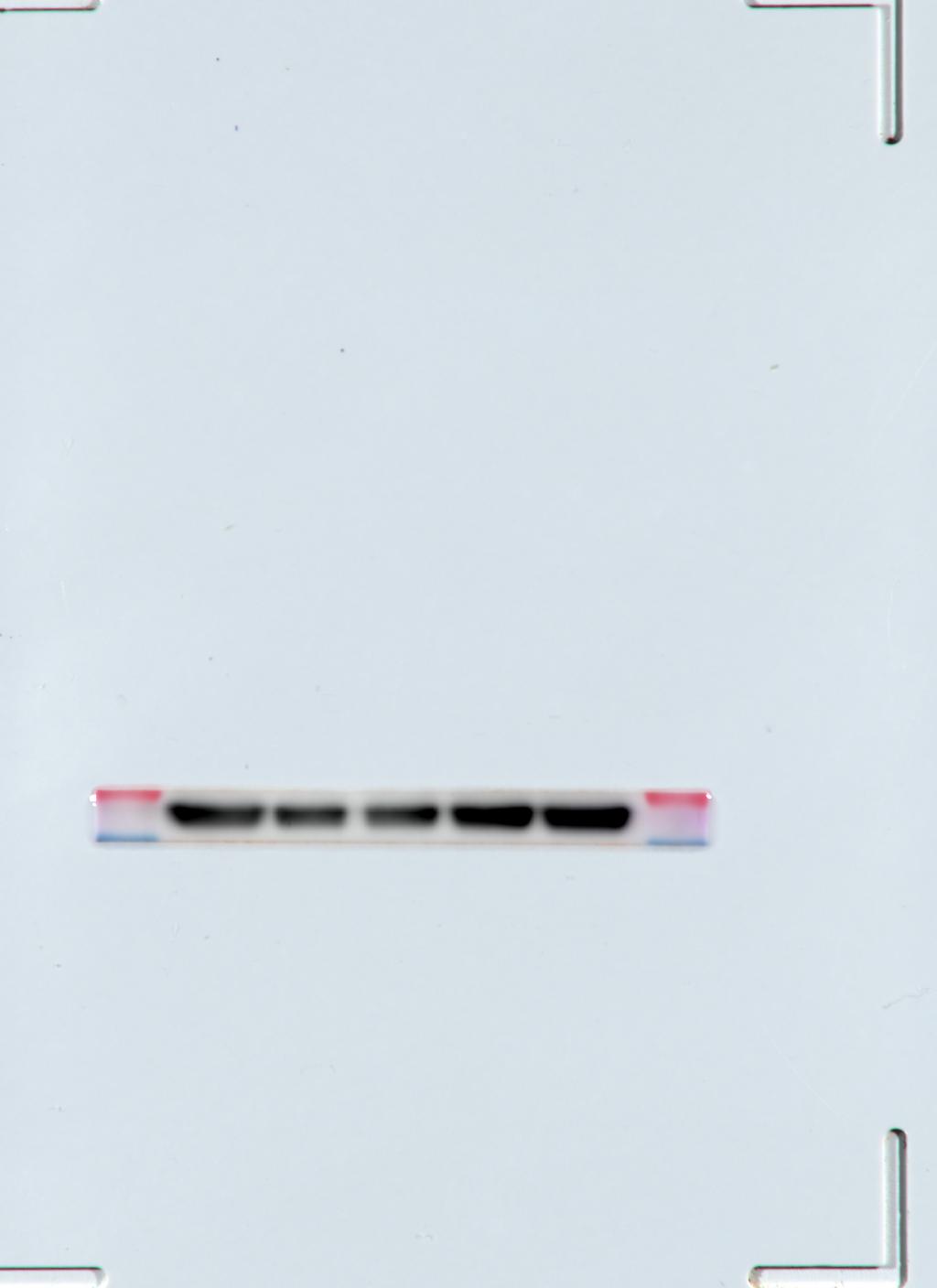
 55kDa

55kDa

TRIM59
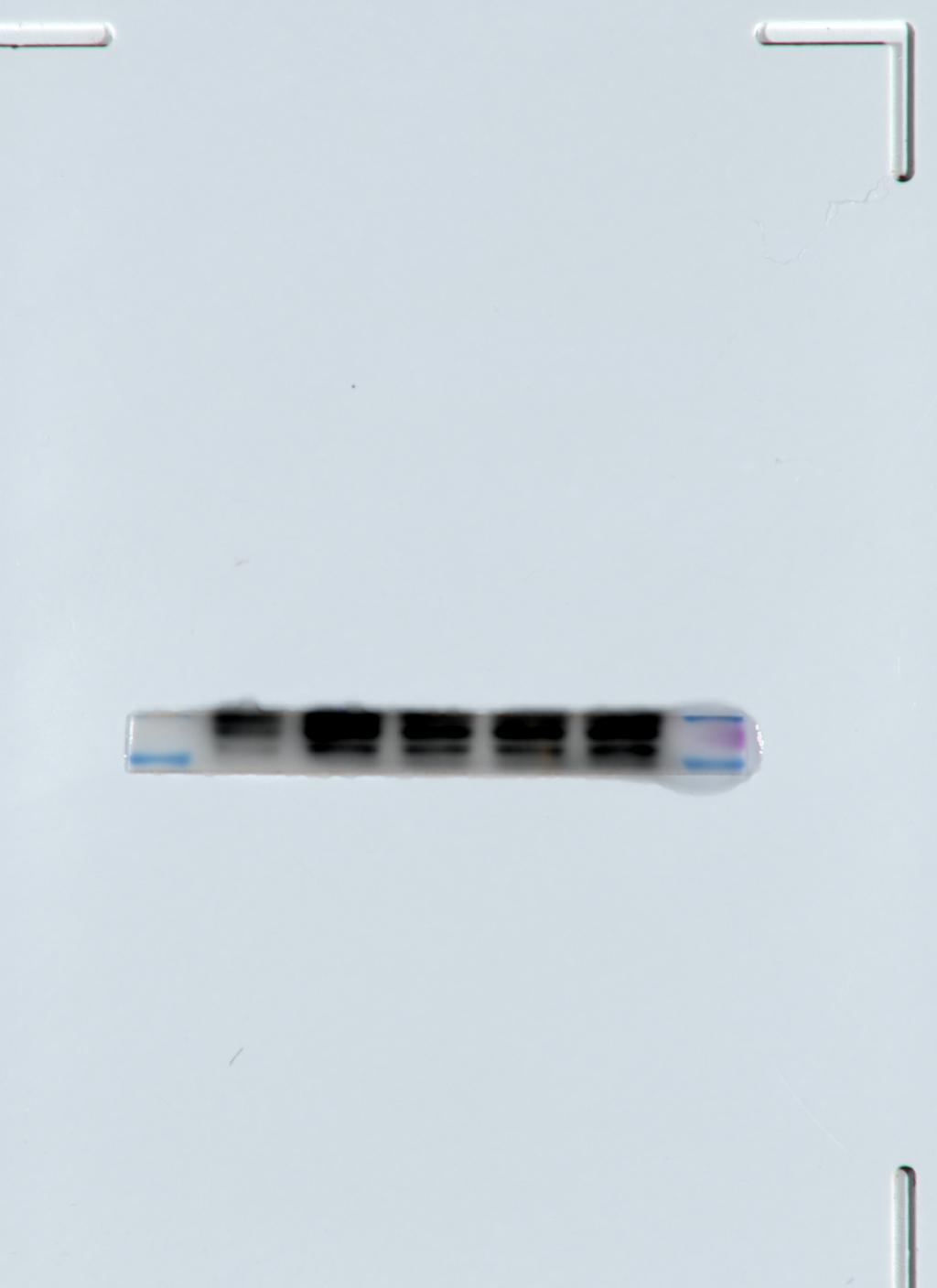
 40kDa

40kDa

GAPDH
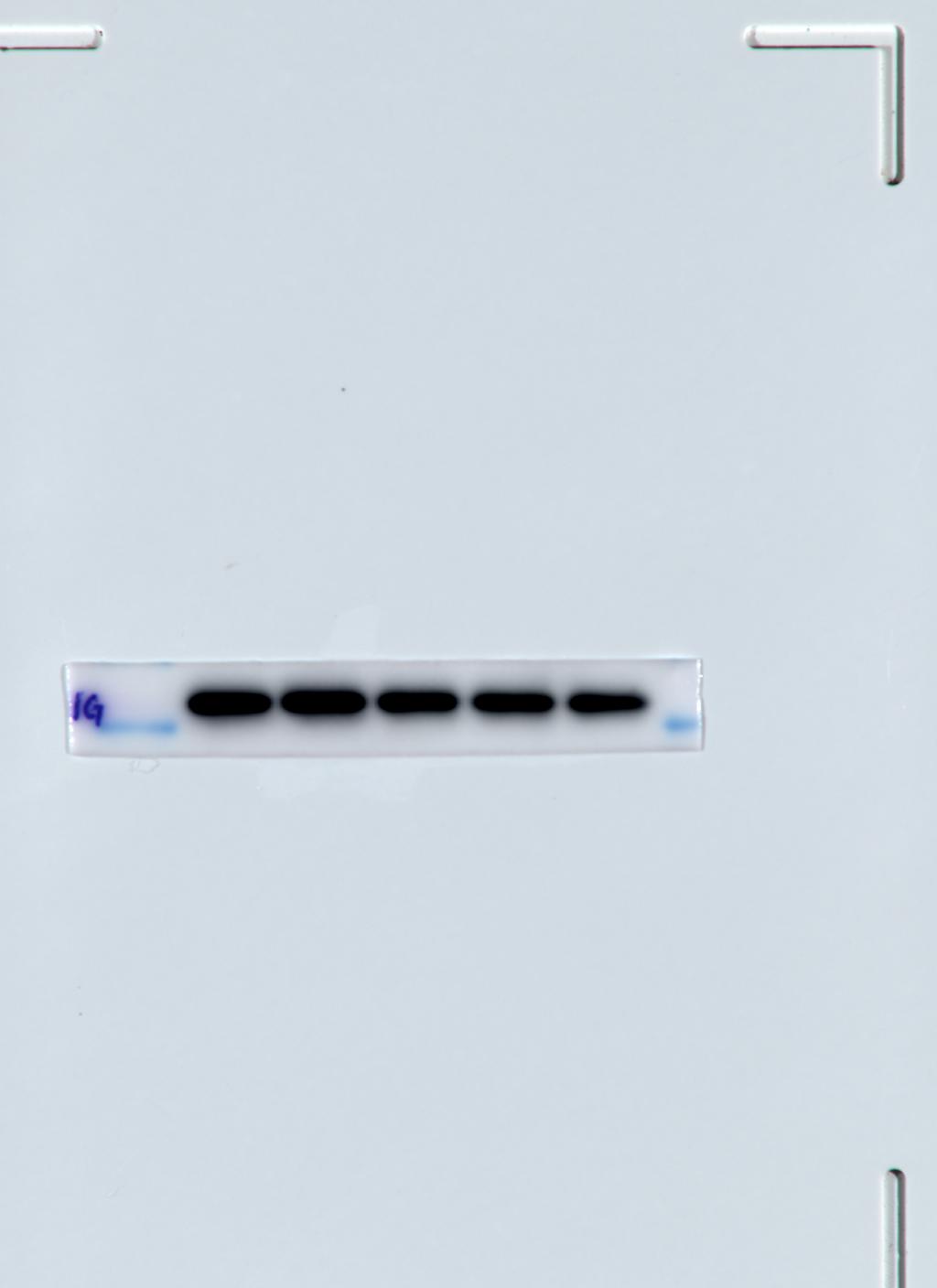
 35kDa


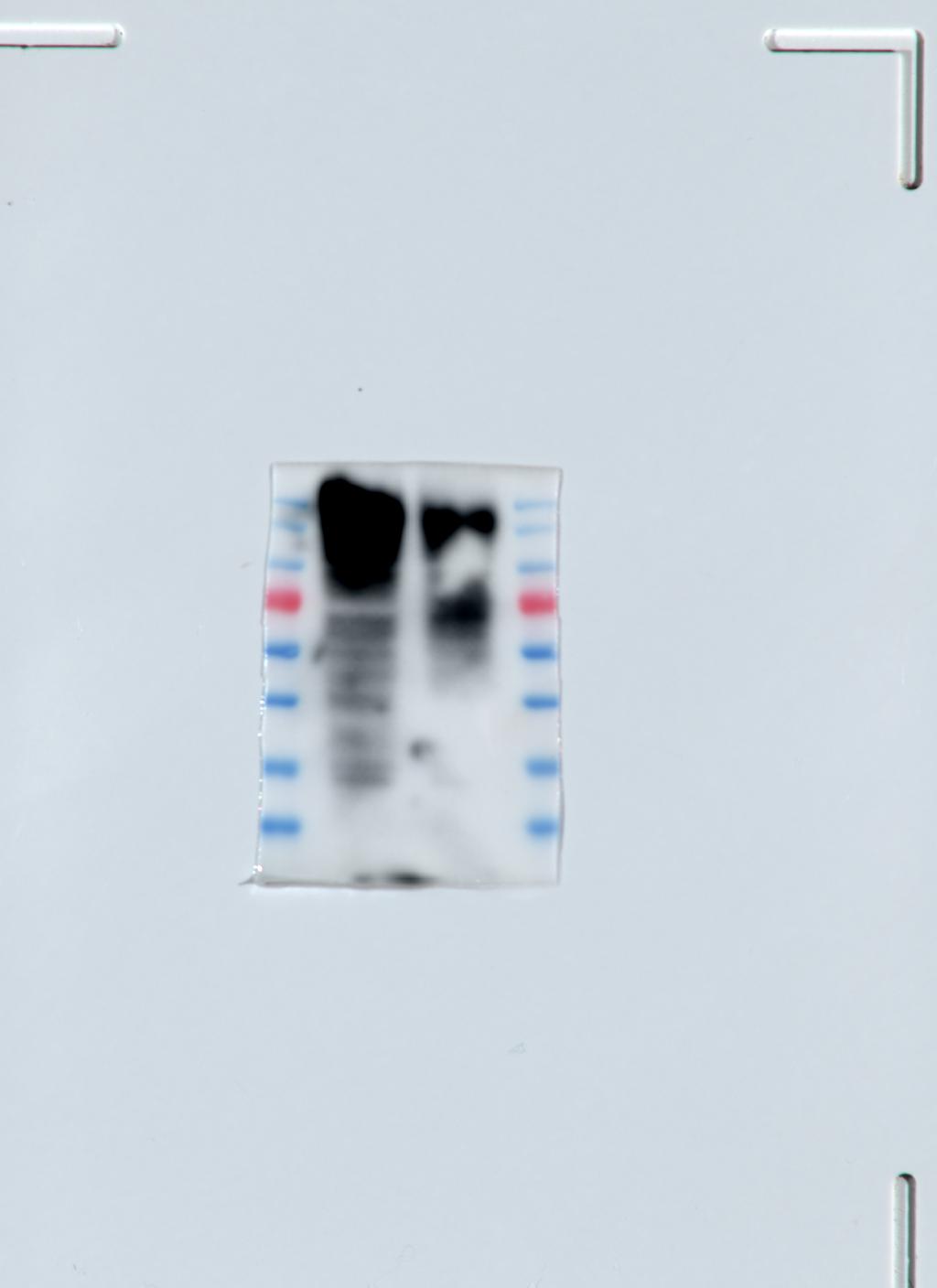

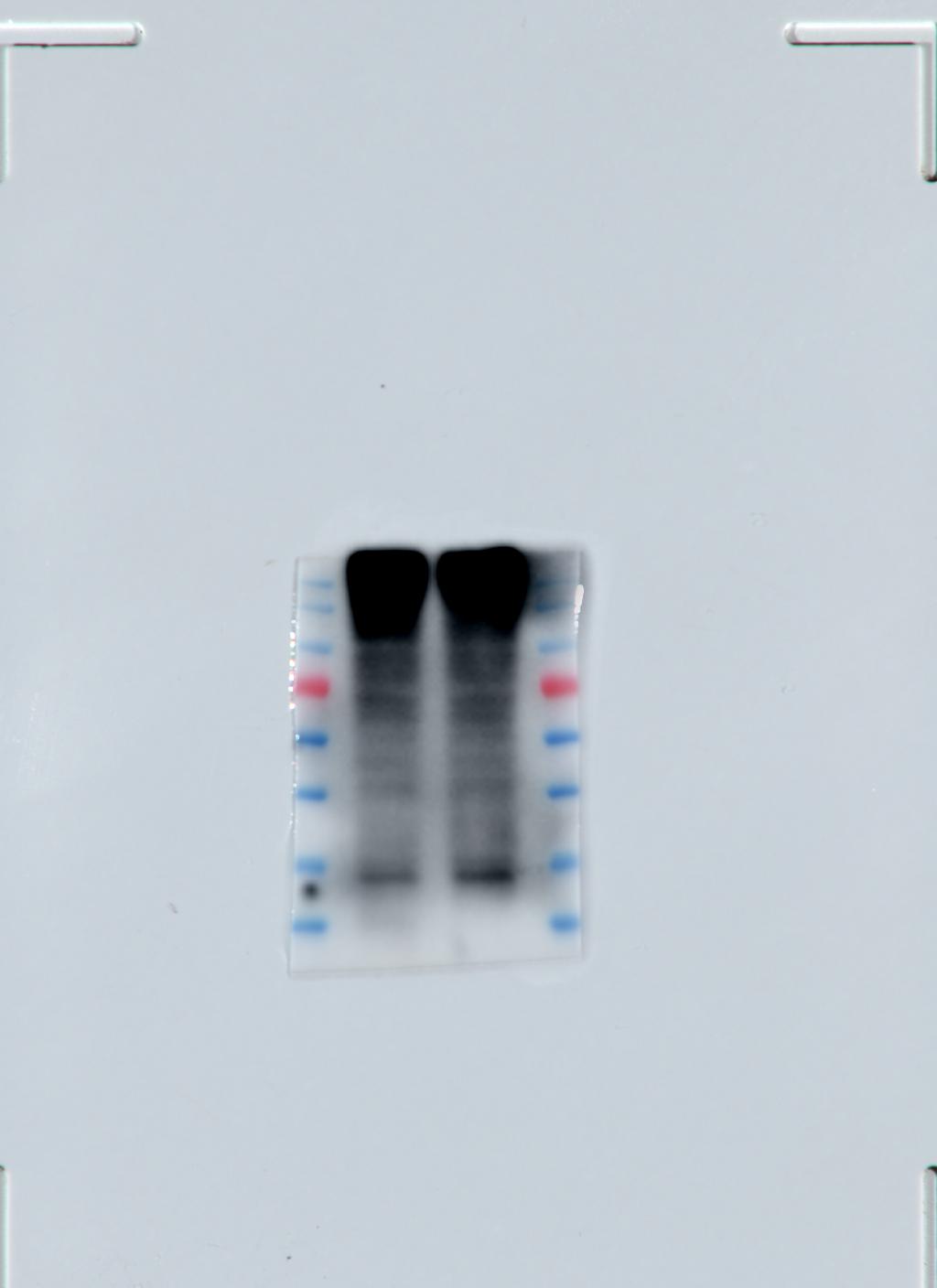

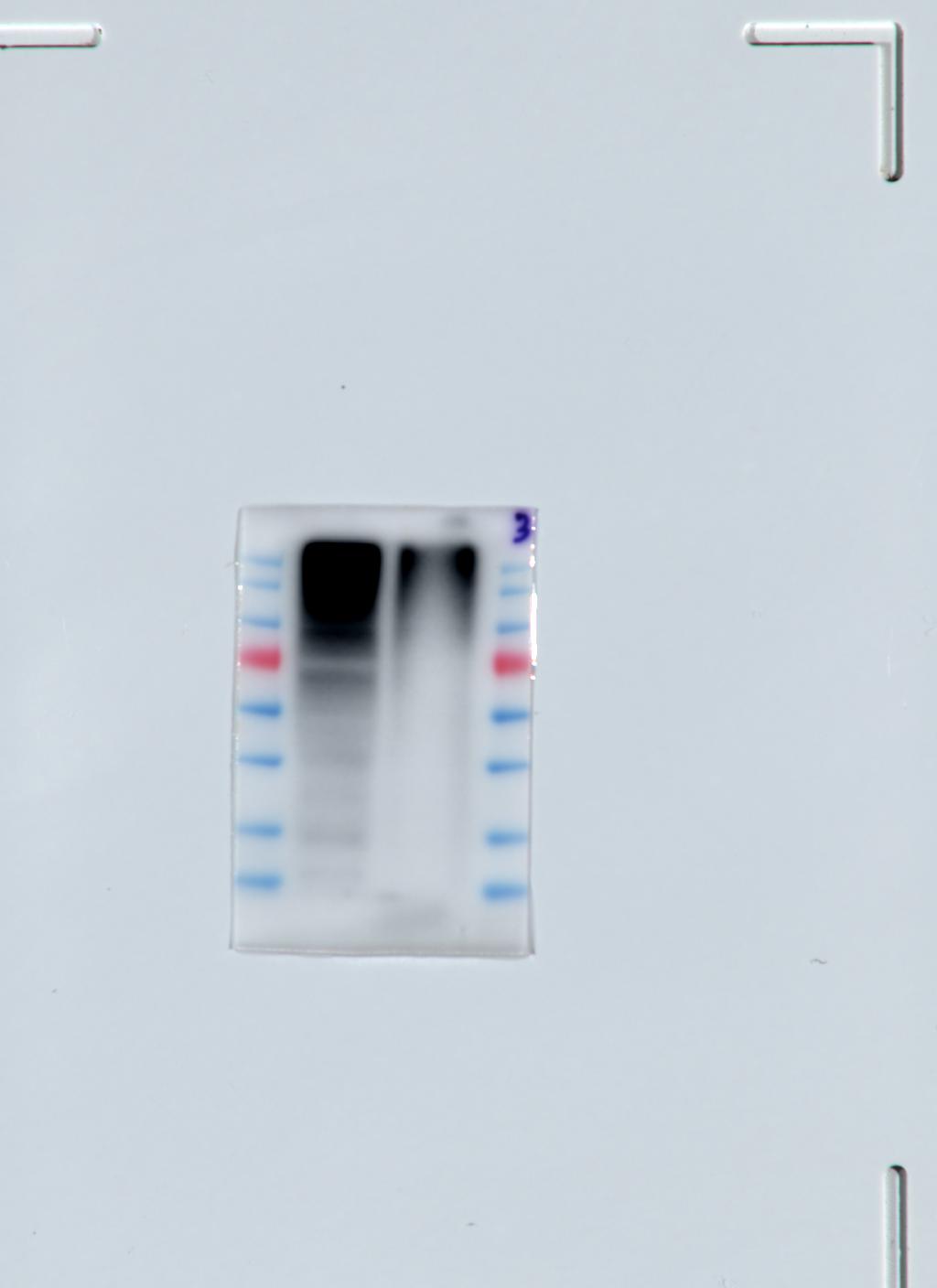

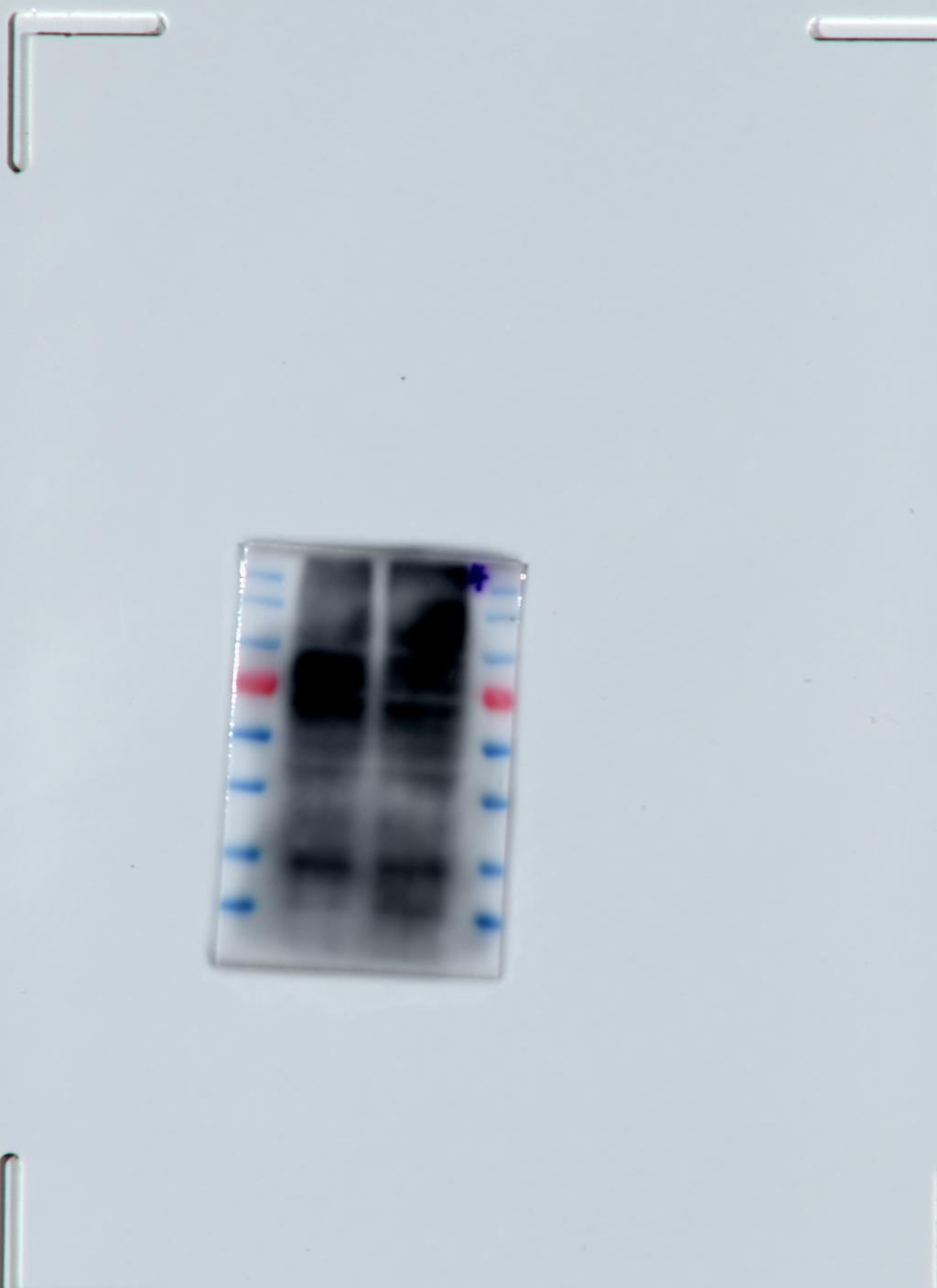


70kDa 70kDa

TRAF6
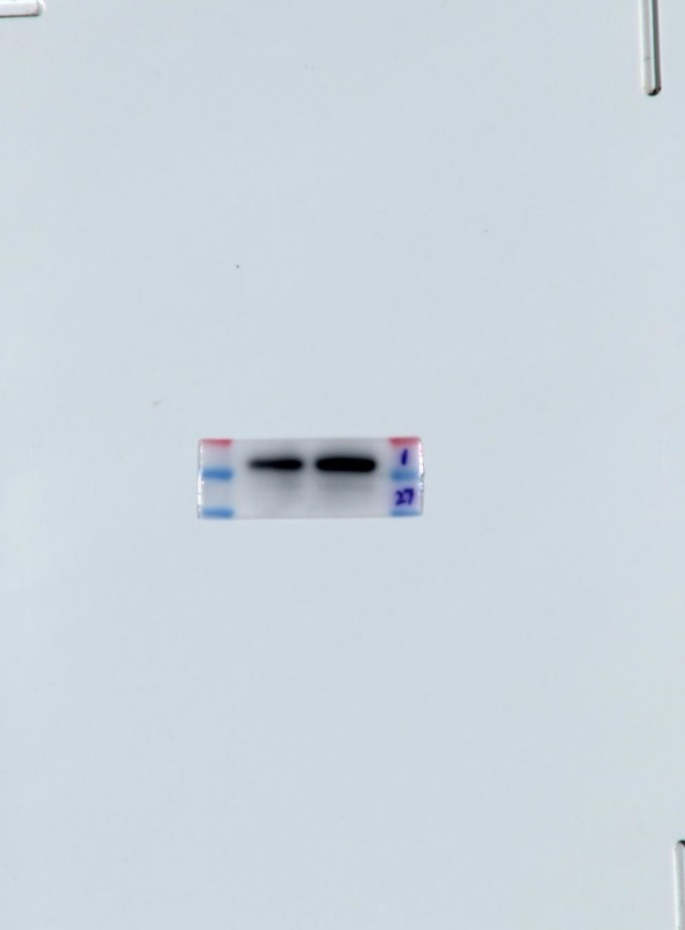
 55kDa TRAF6
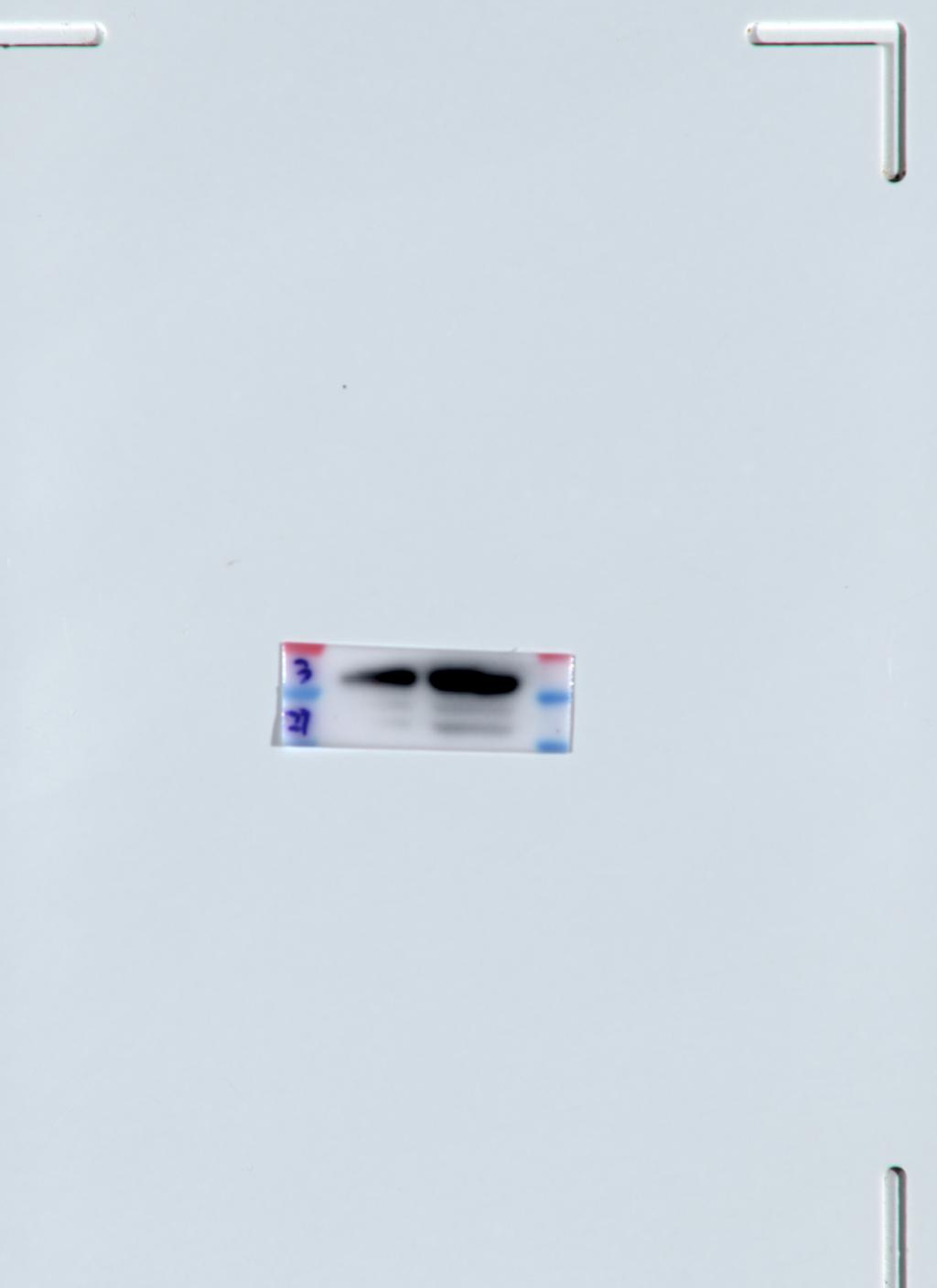
 55kDa

55kDa 55kDa

TRIM59
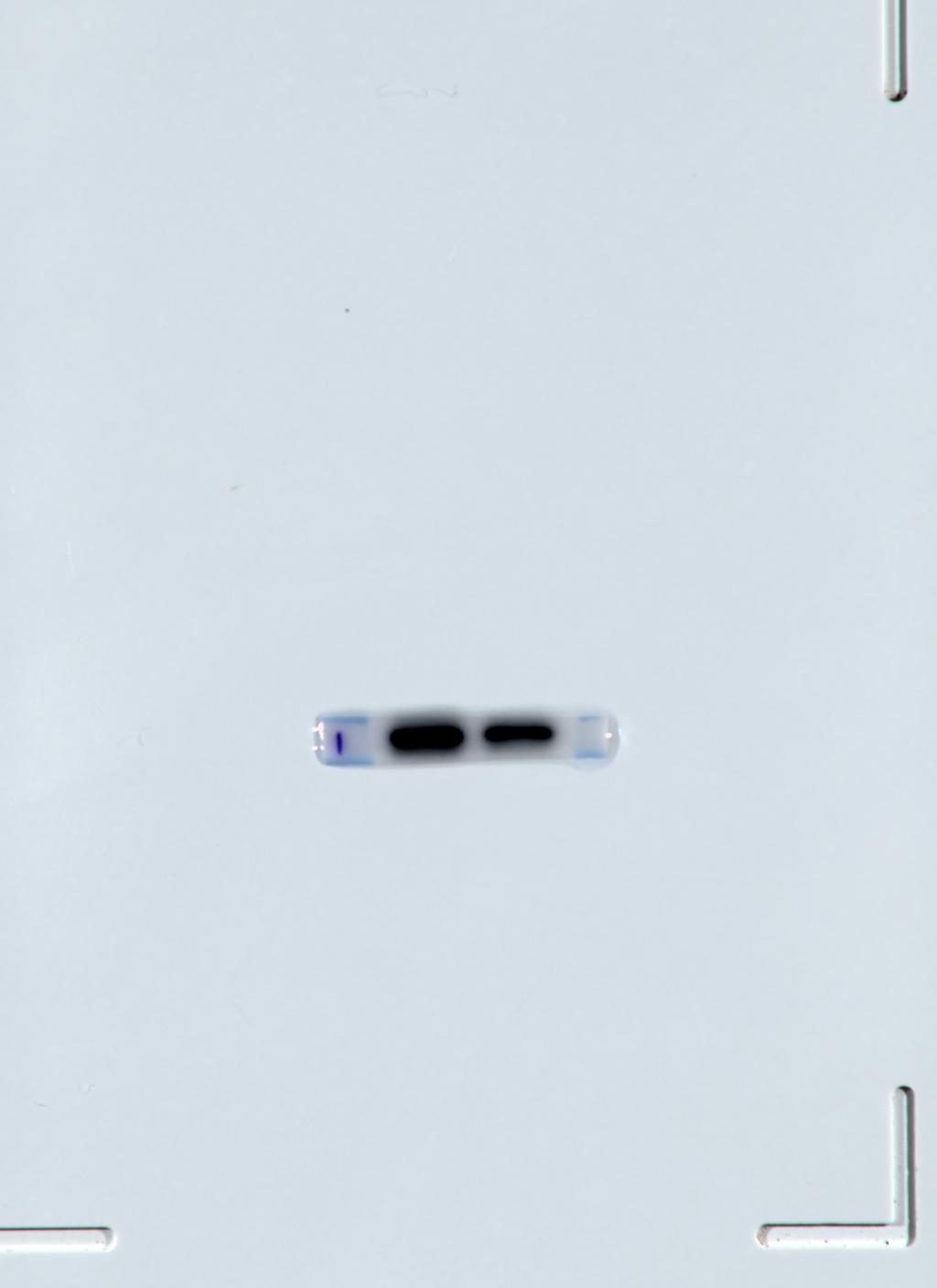
 40kDa TRIM59
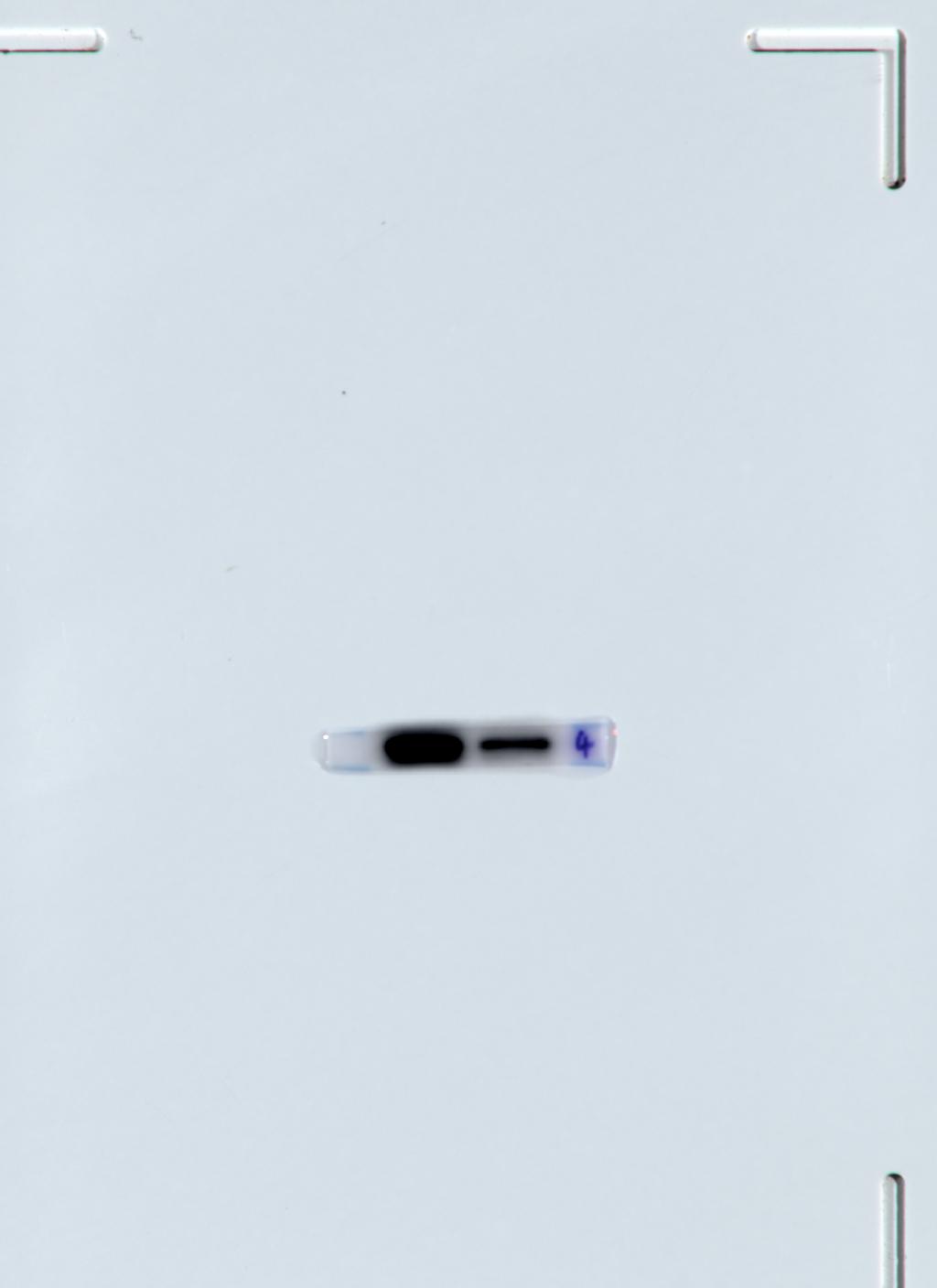
 40kDa

40kDa 40kDa

GAPDH
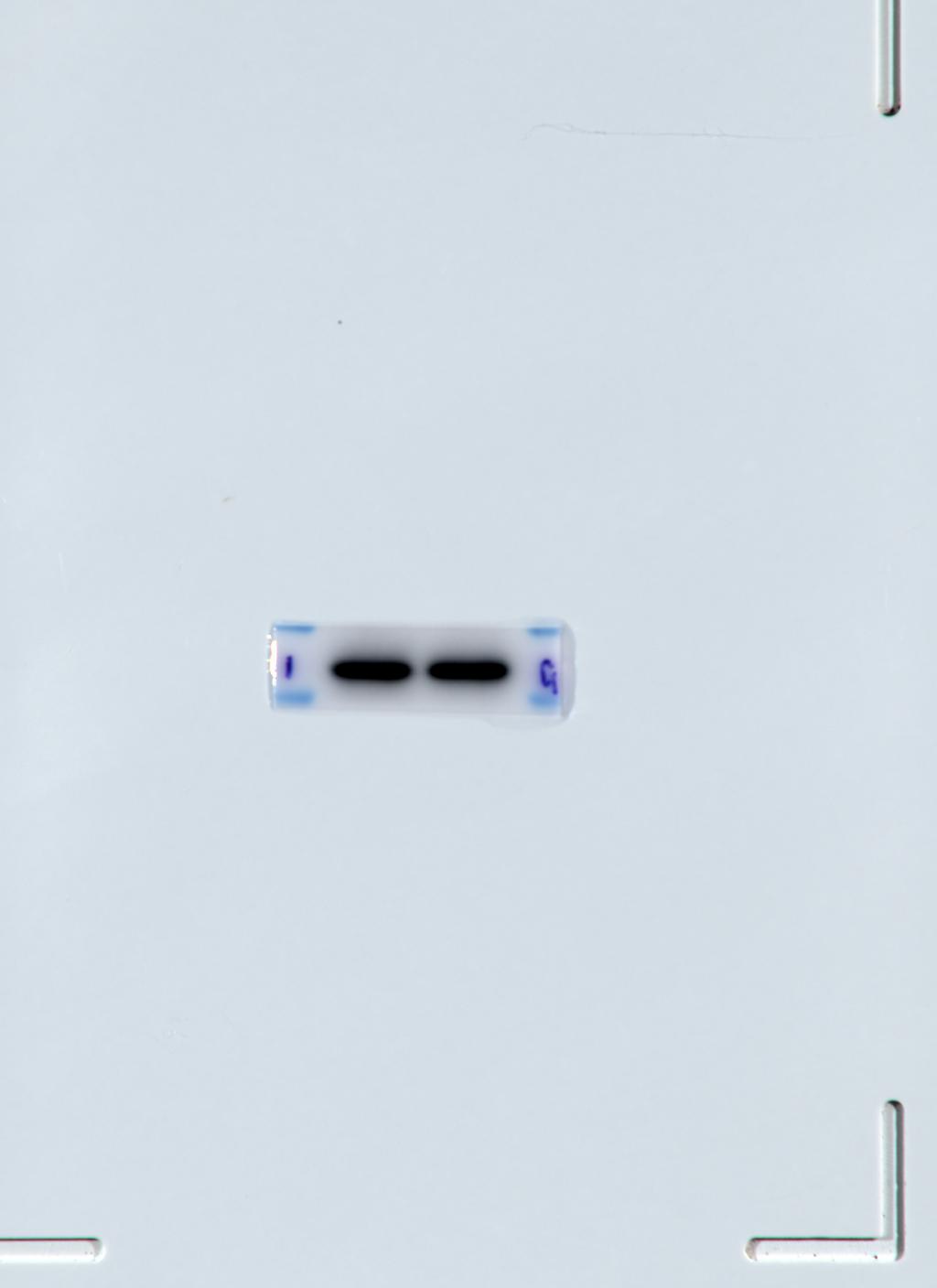
 35kDa GAPDH
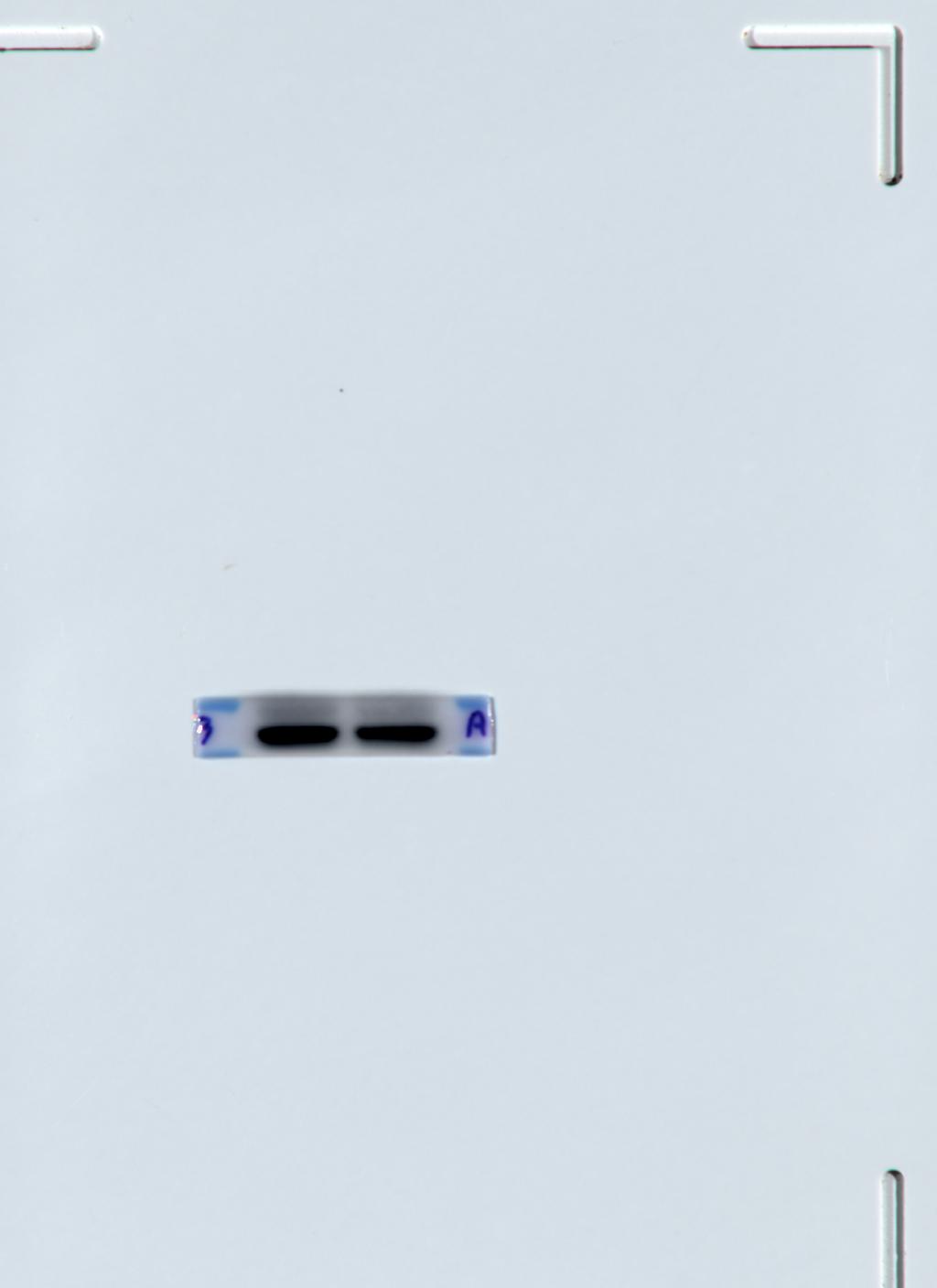
 35kDa


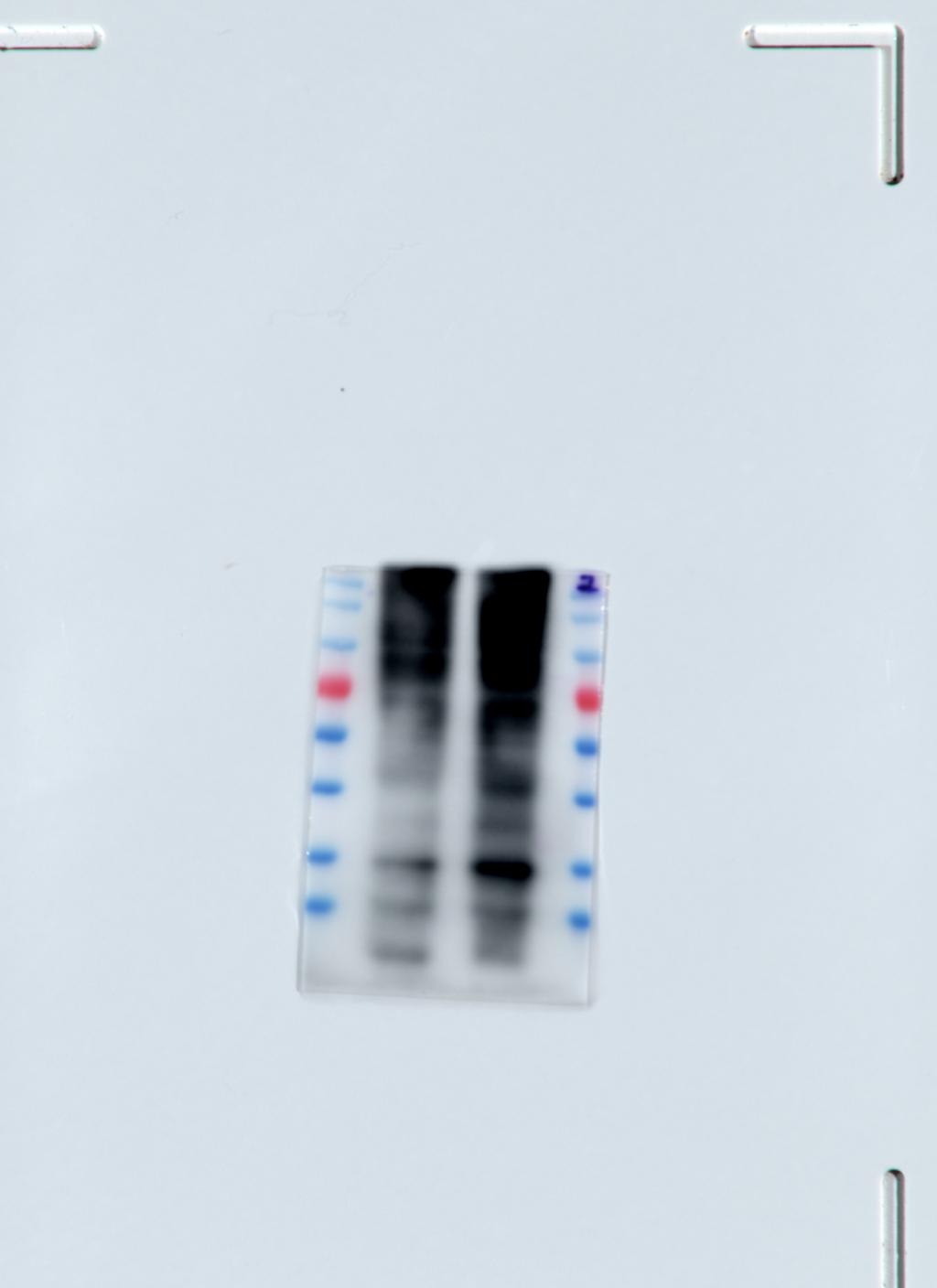

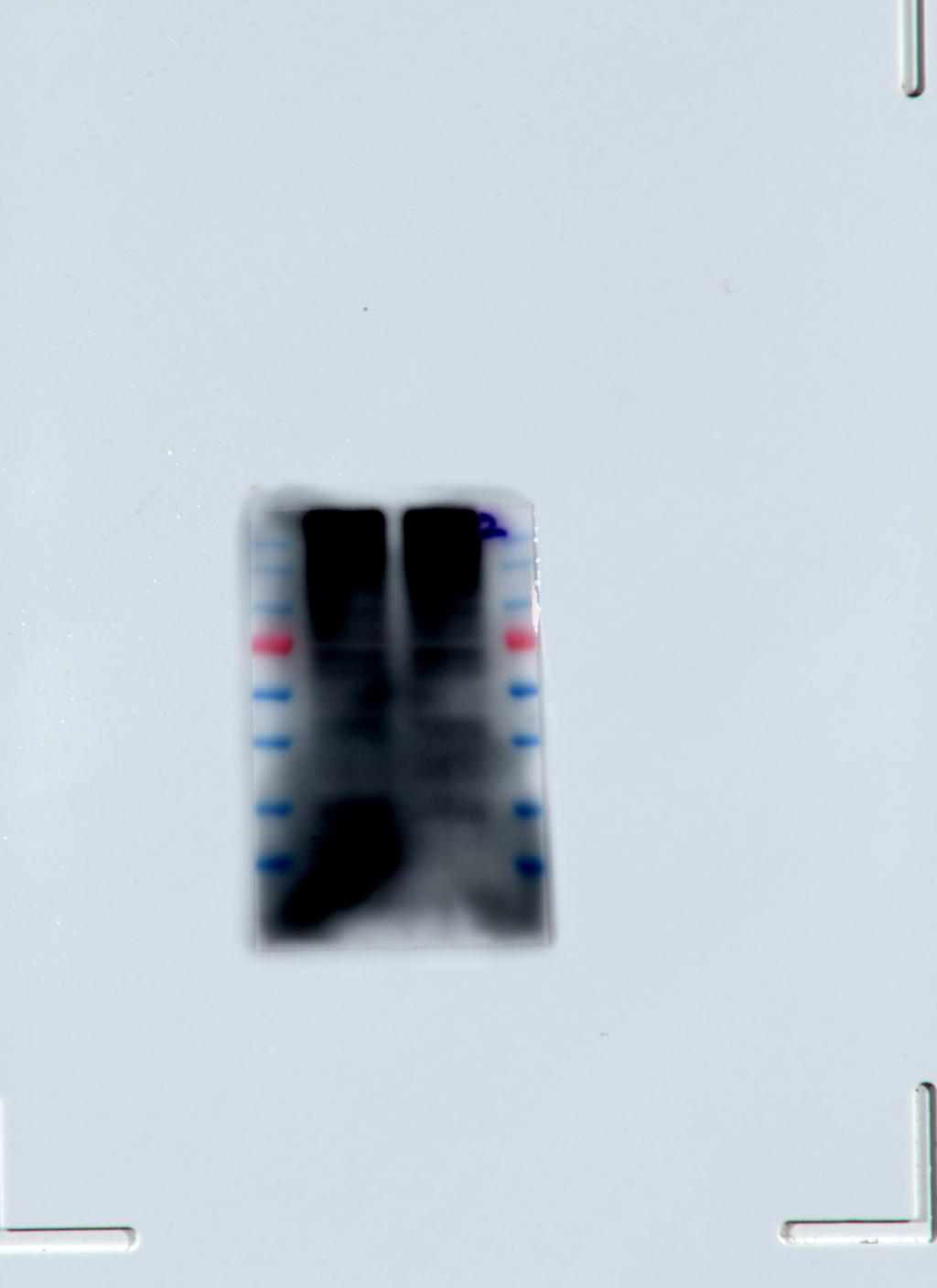

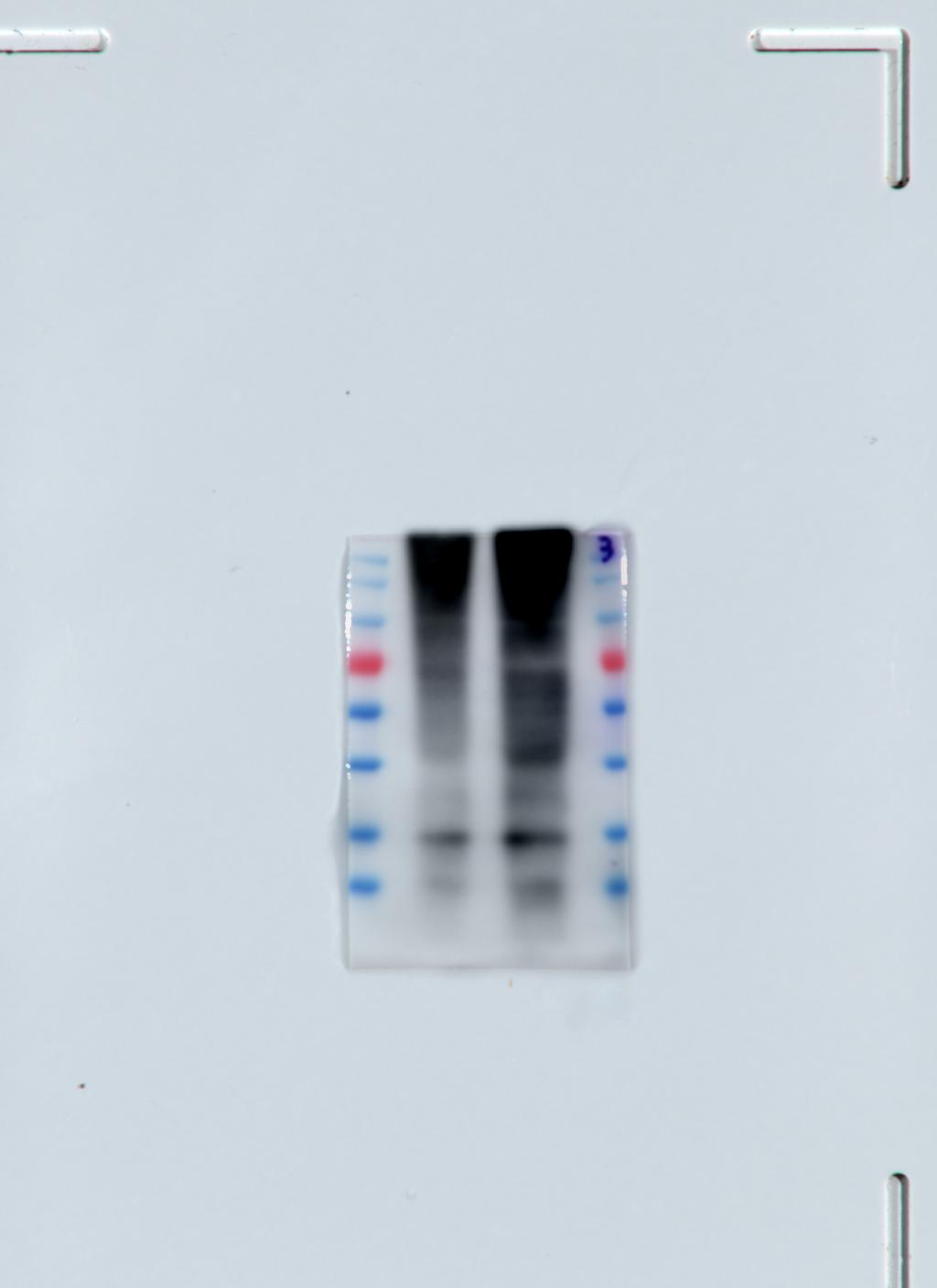

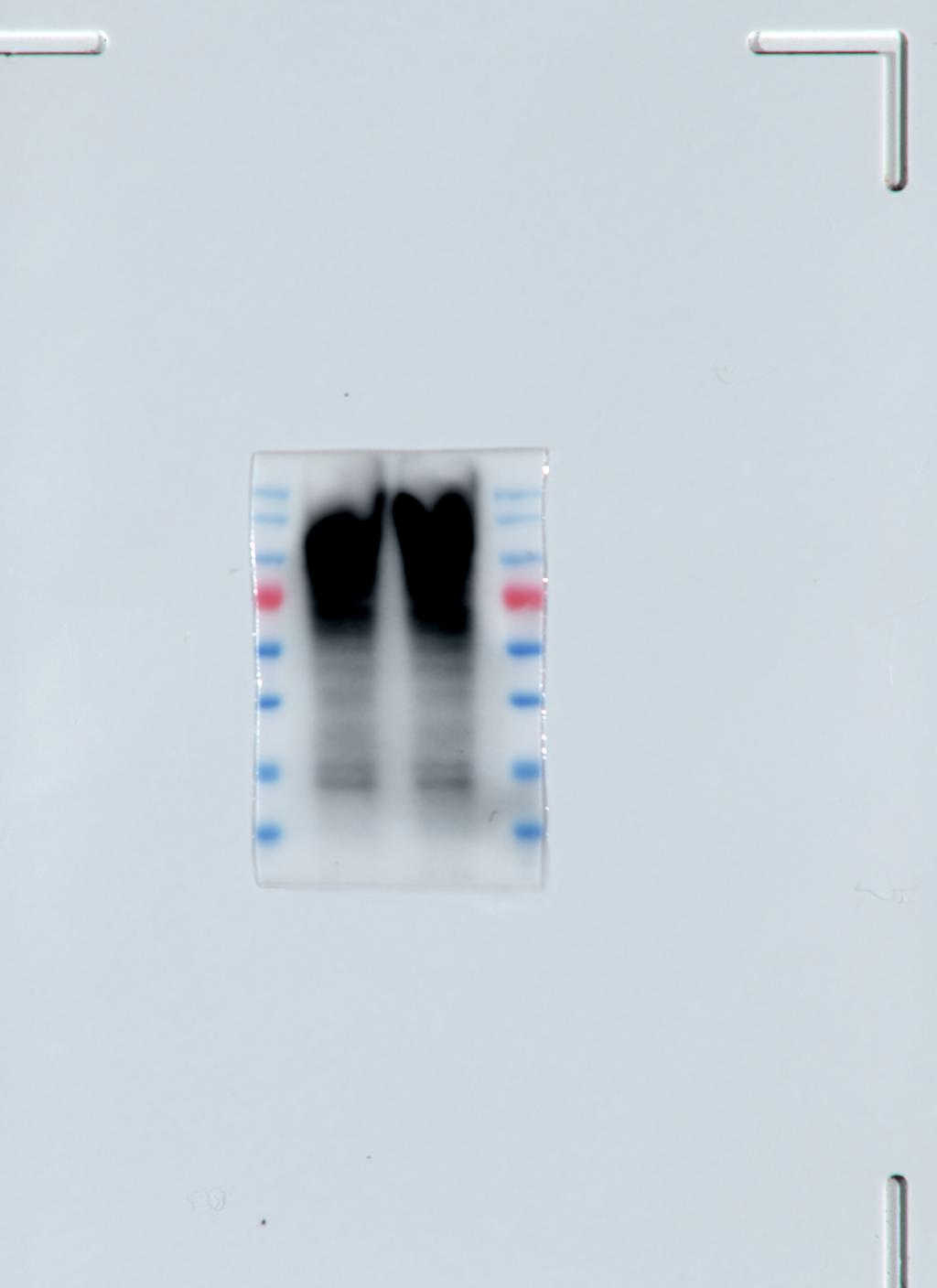


70kDa 70kDa

TRAF6
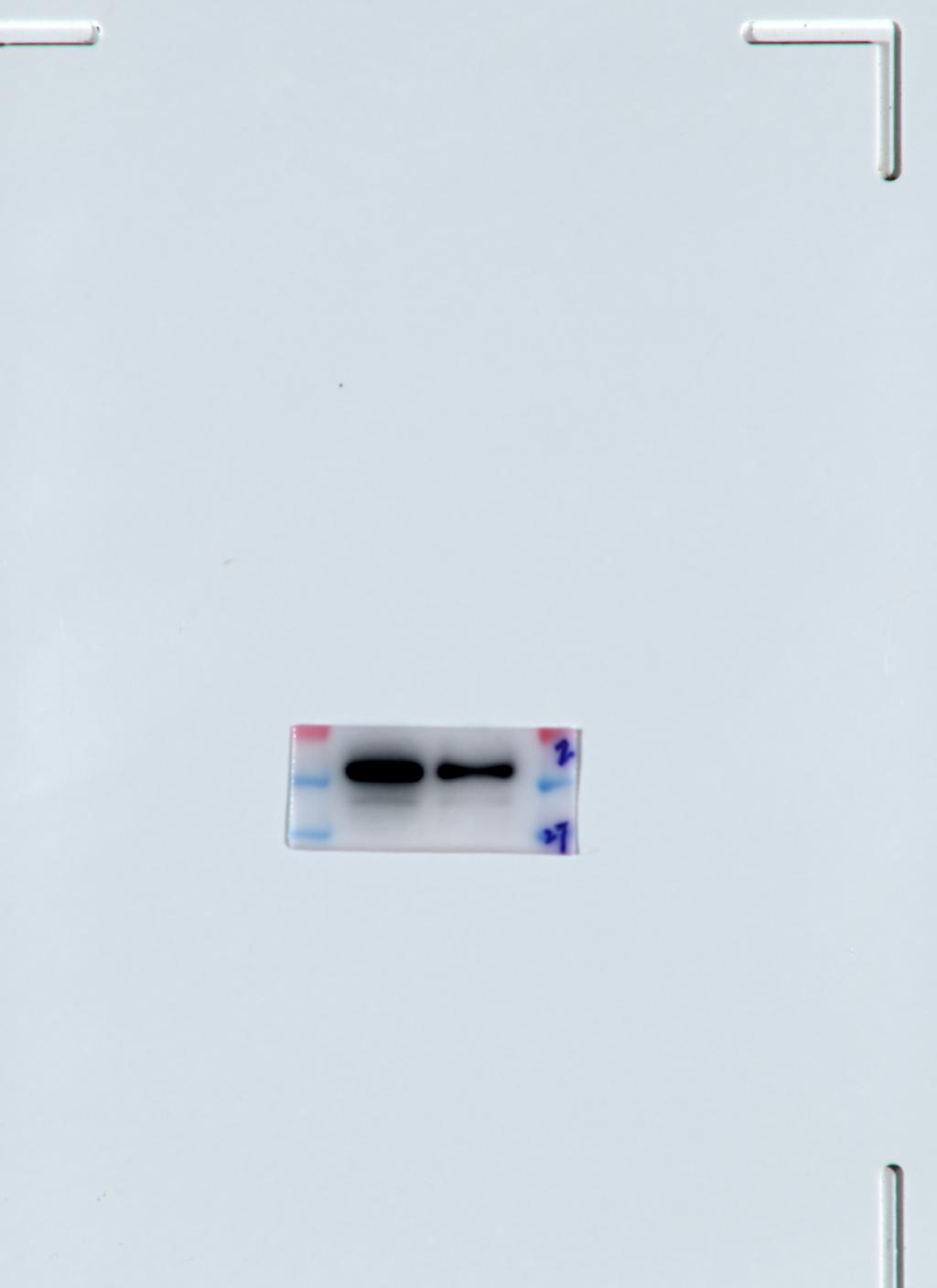
 55kDa TRAF6
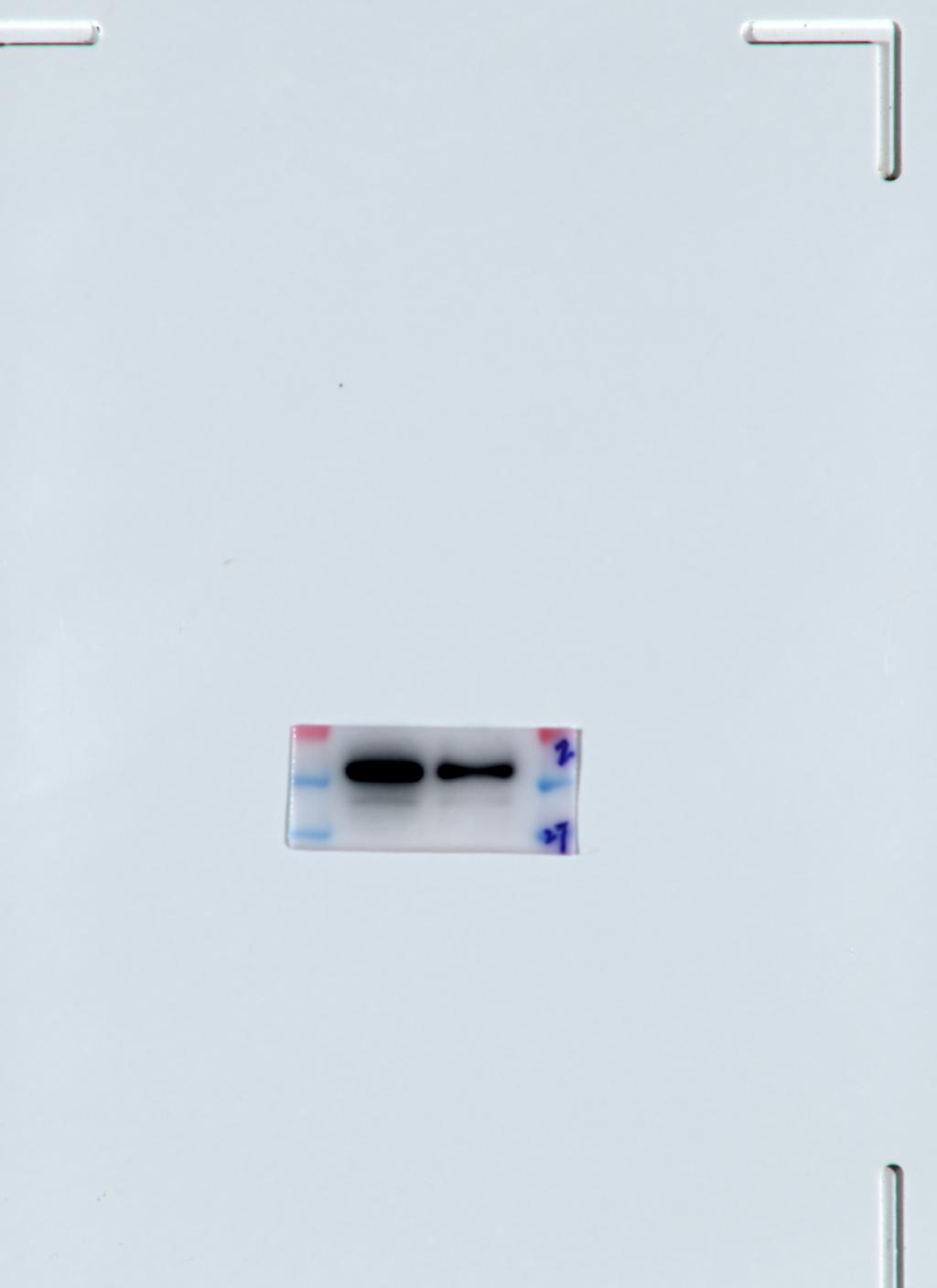
 55kDa

55kDa 55kDa

TRIM59
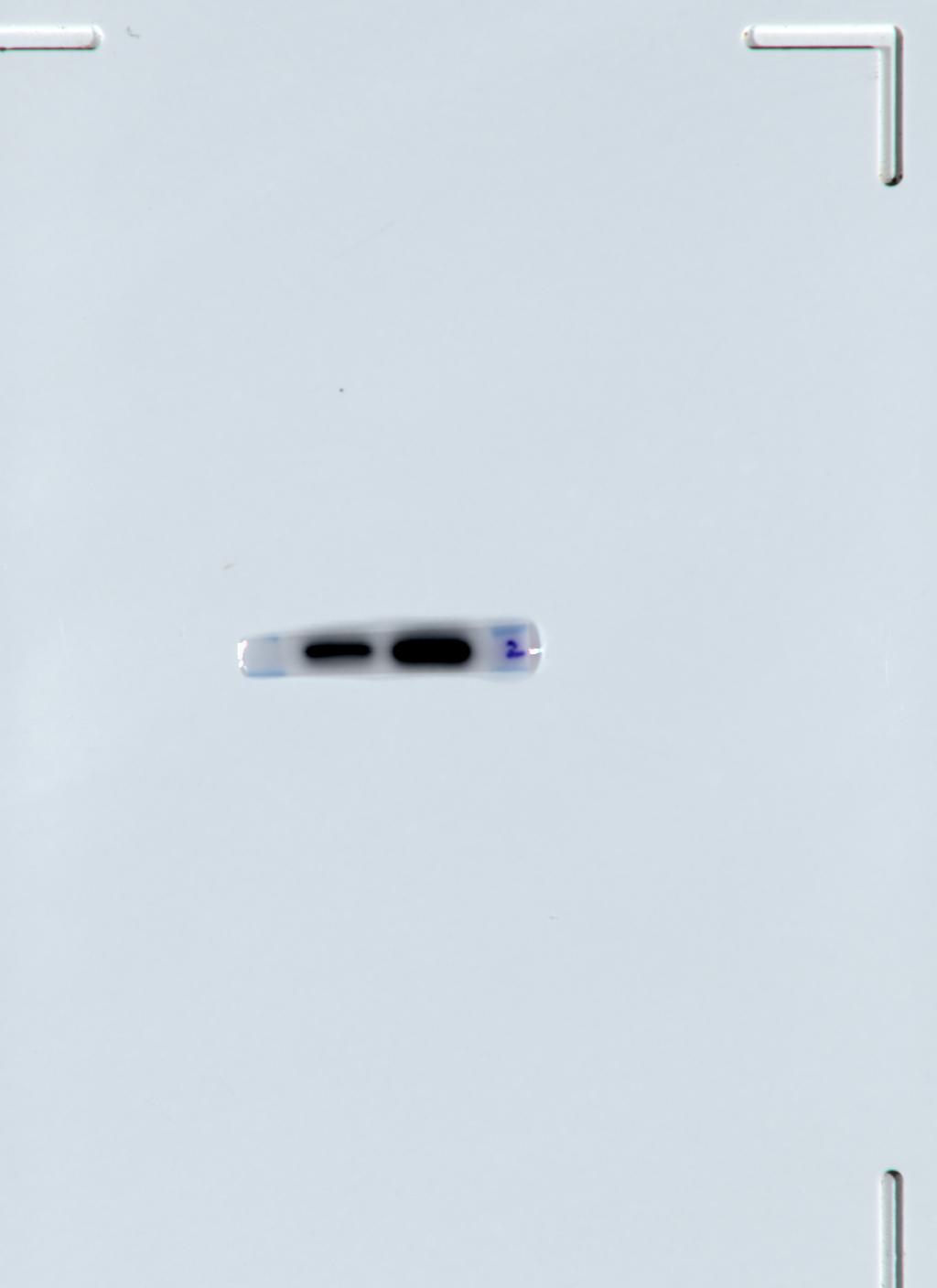
 40kDa TRIM59
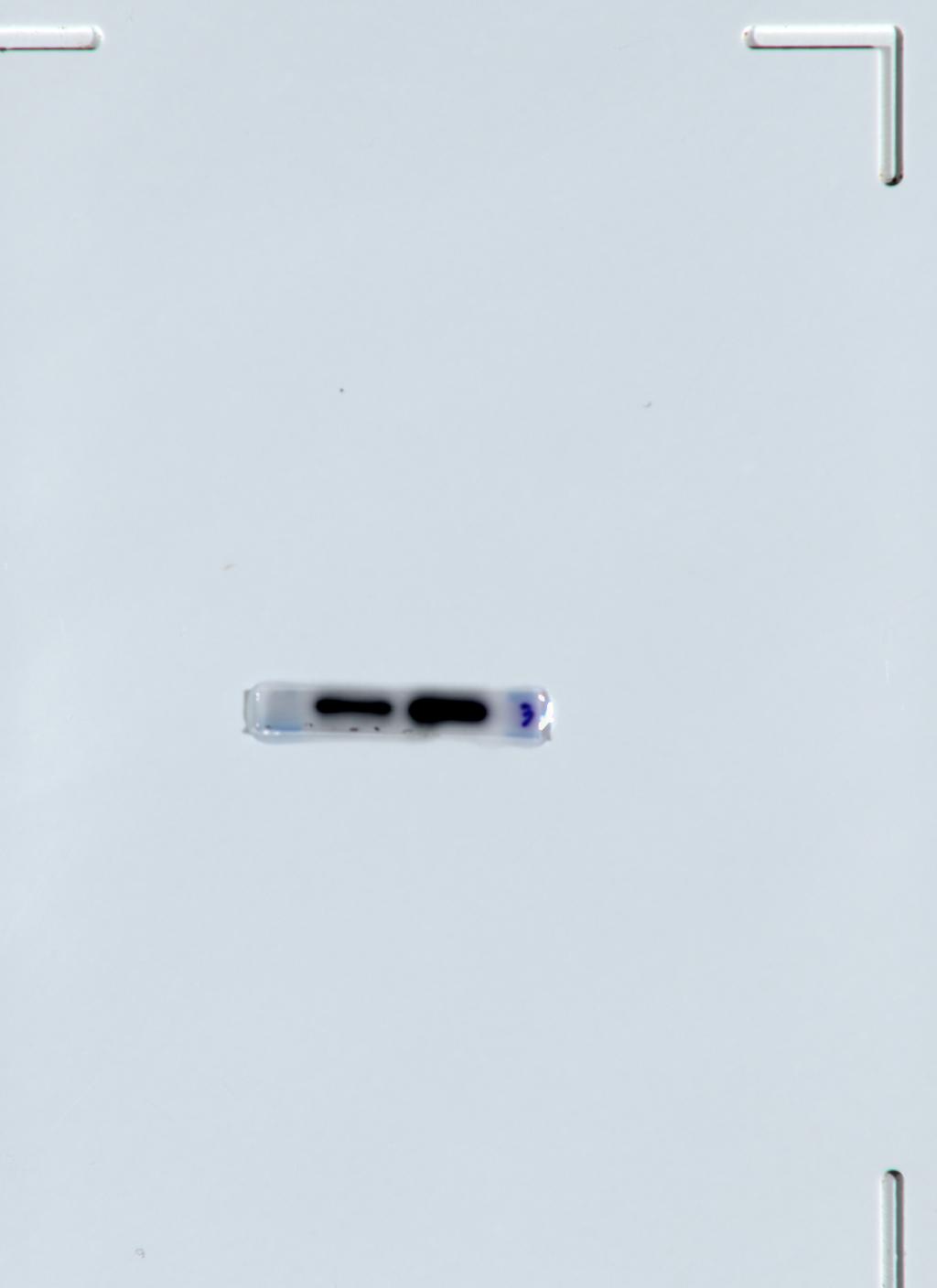
 40kDa

40kDa 40kDa

GAPDH
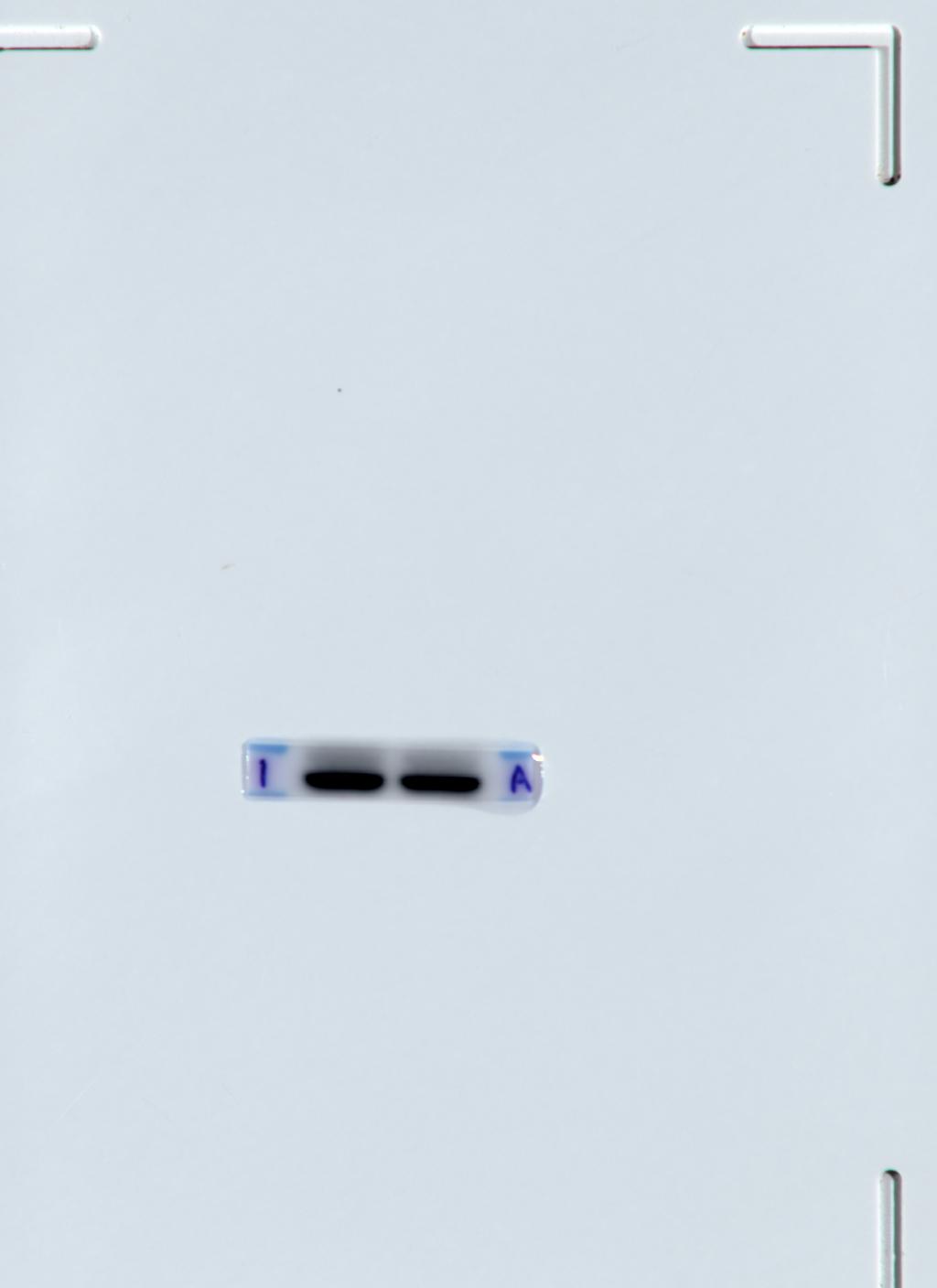
 35kDa GAPDH
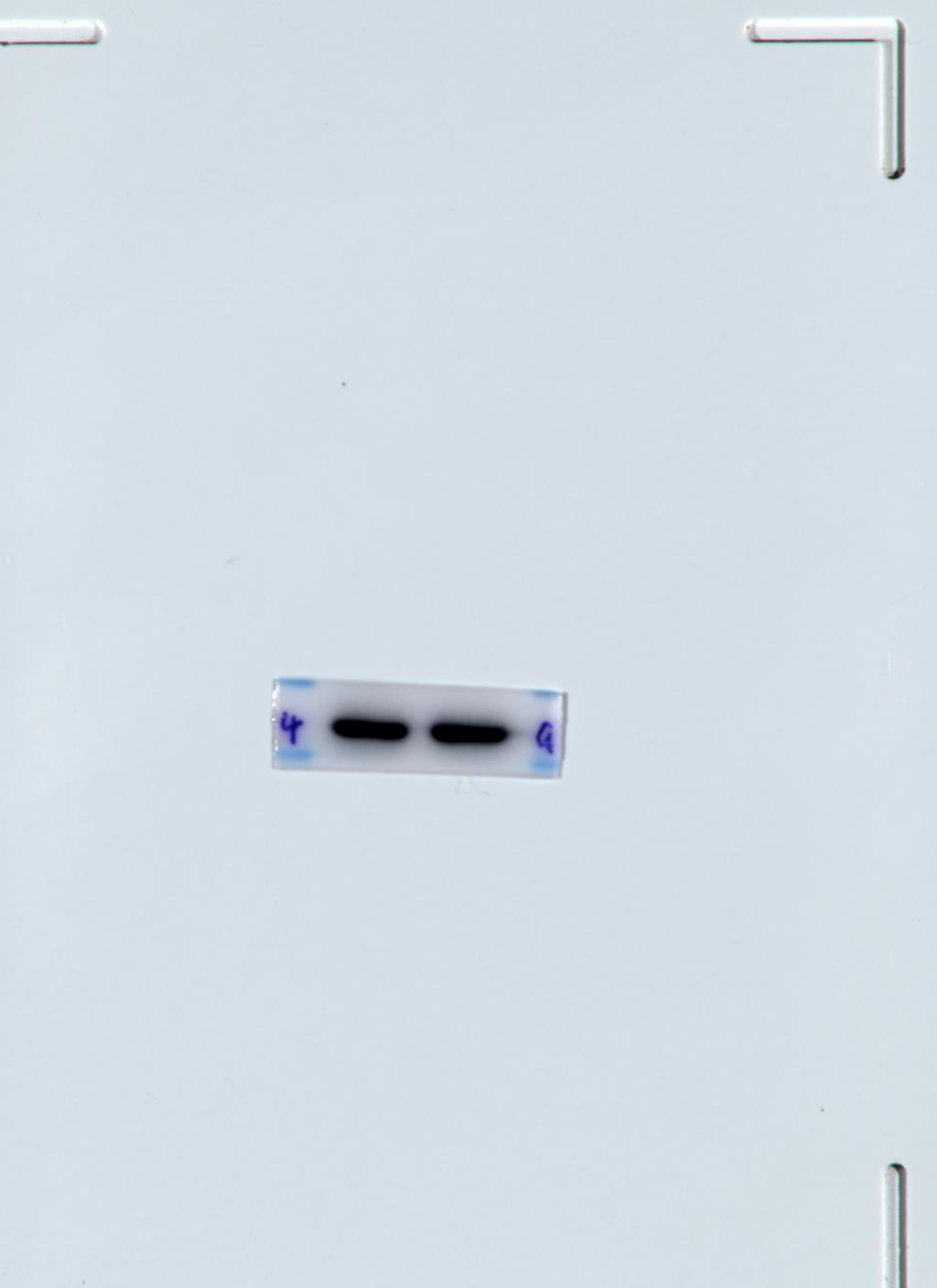
 35kDa

70kDa

TRAF6
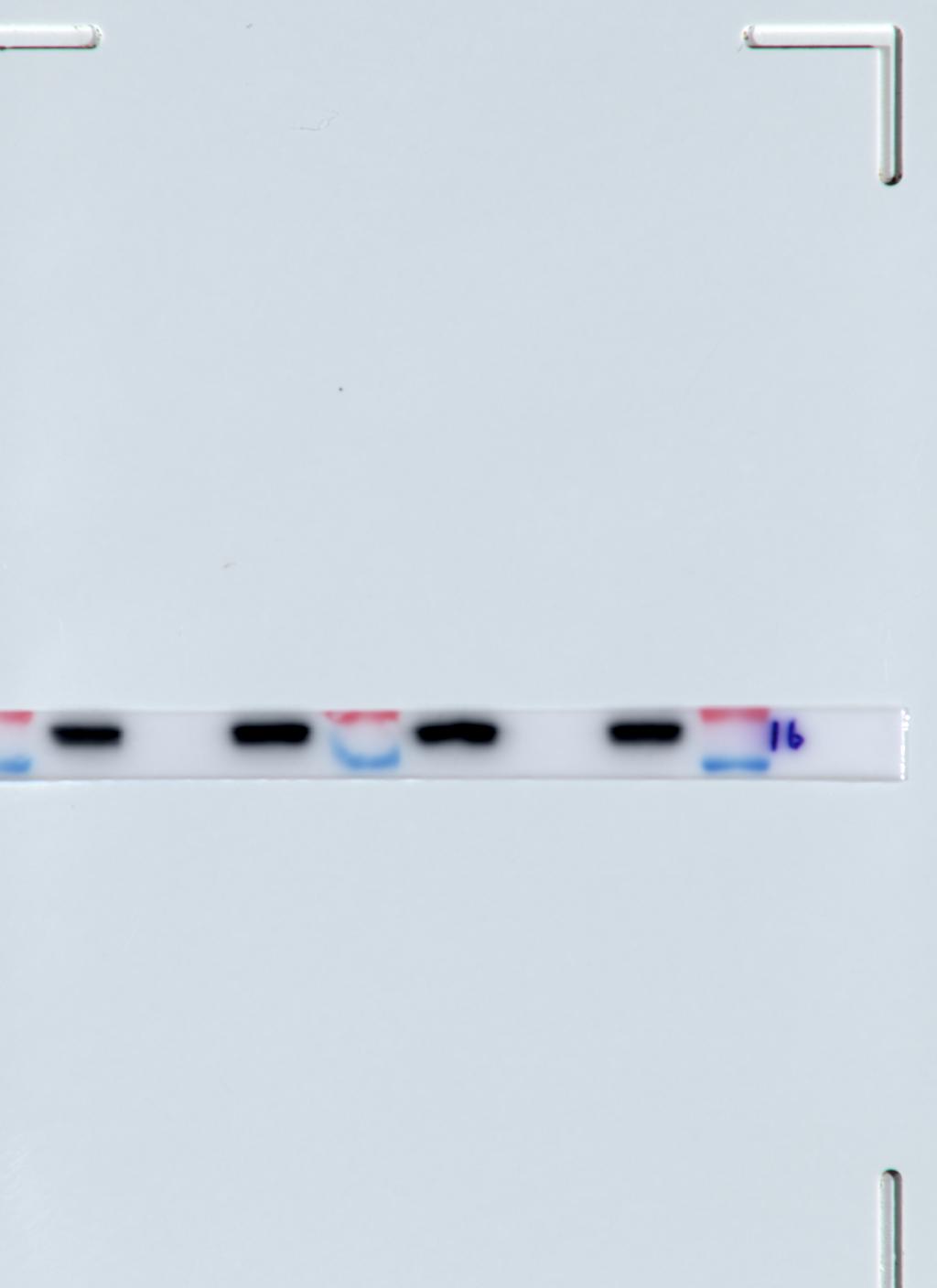
 55kDa

130kDa

TRL4
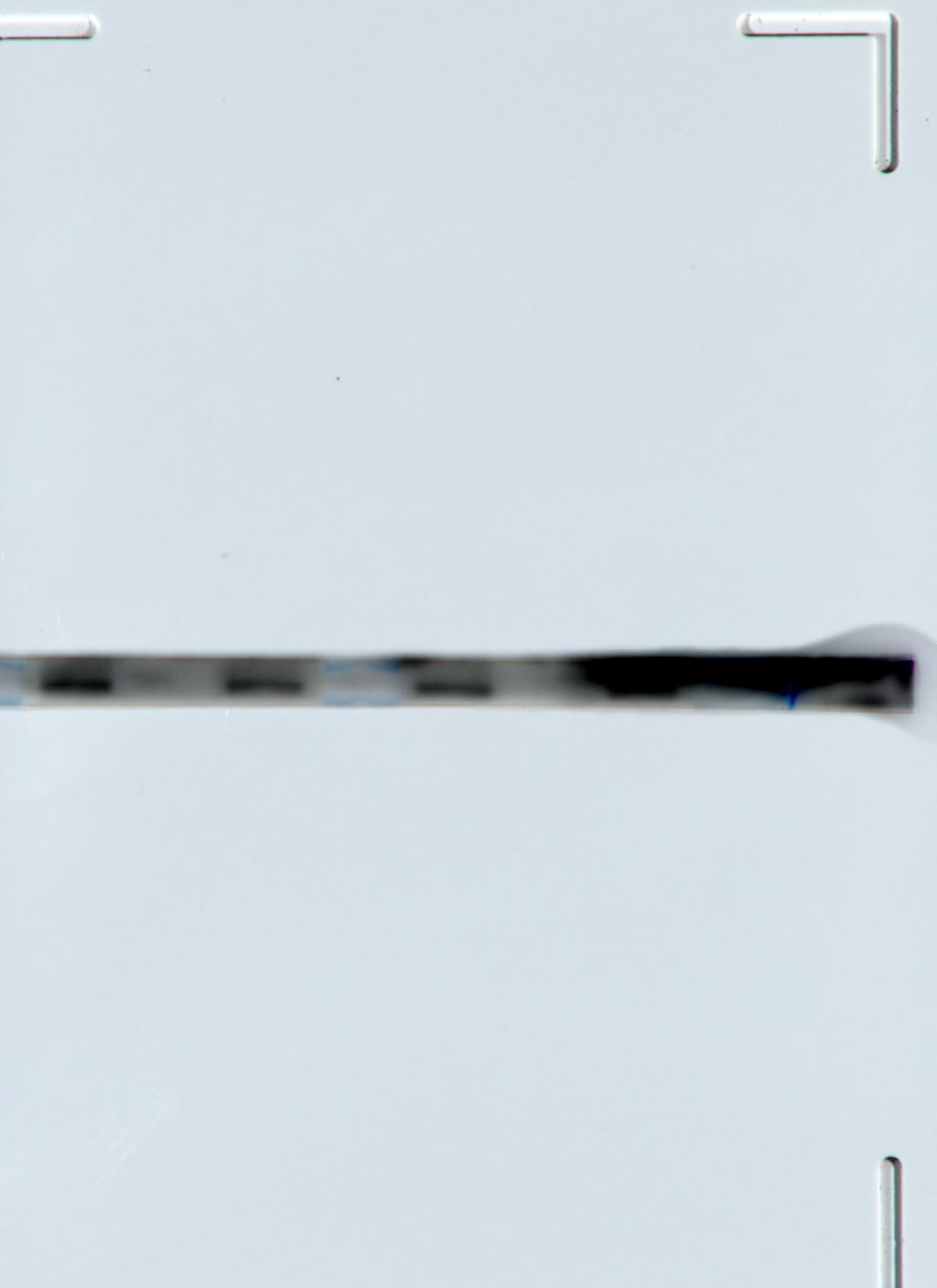
 100kDa

70kDa

TRAF6
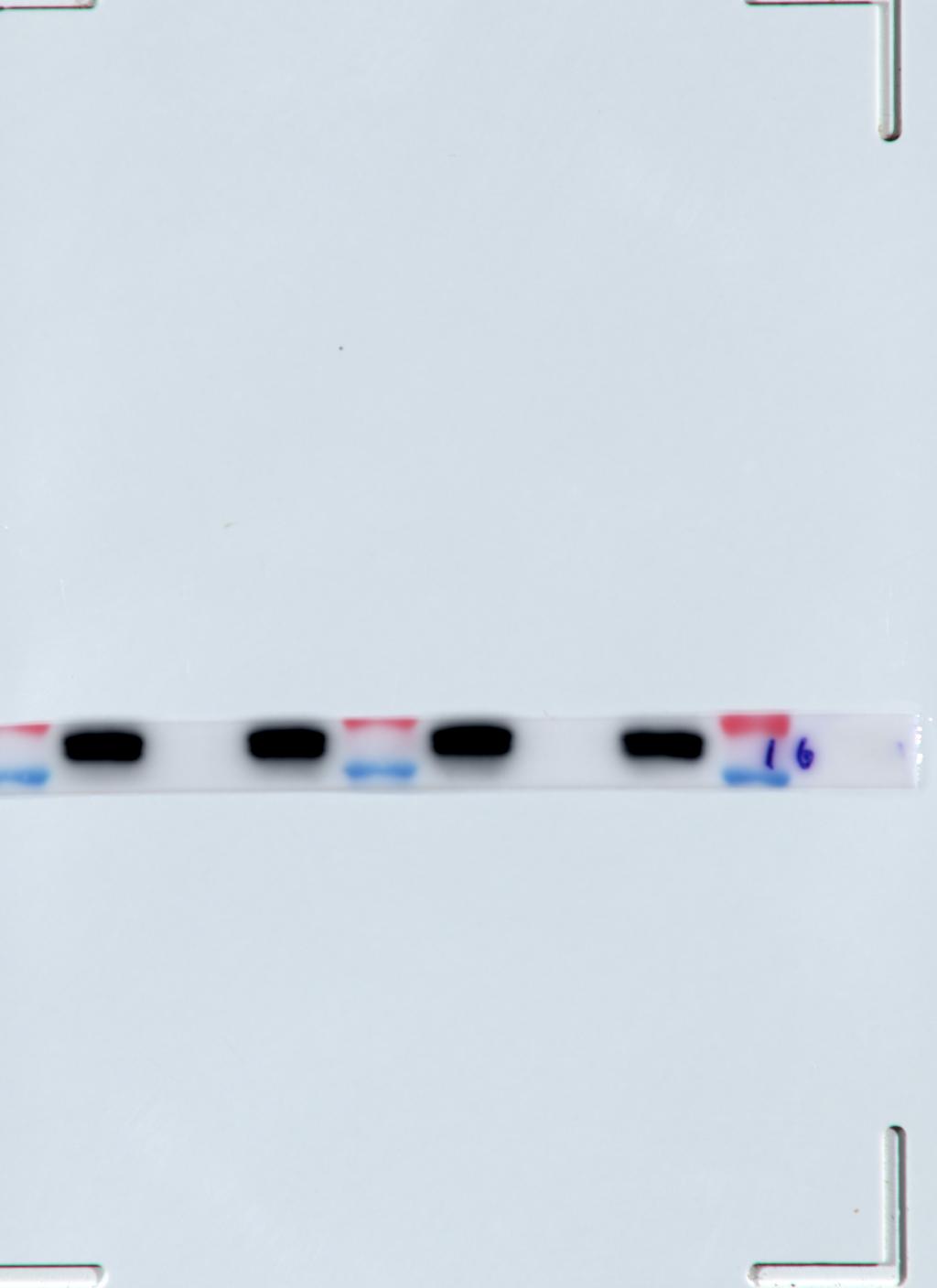
 55kDa

130kDa

TLR4
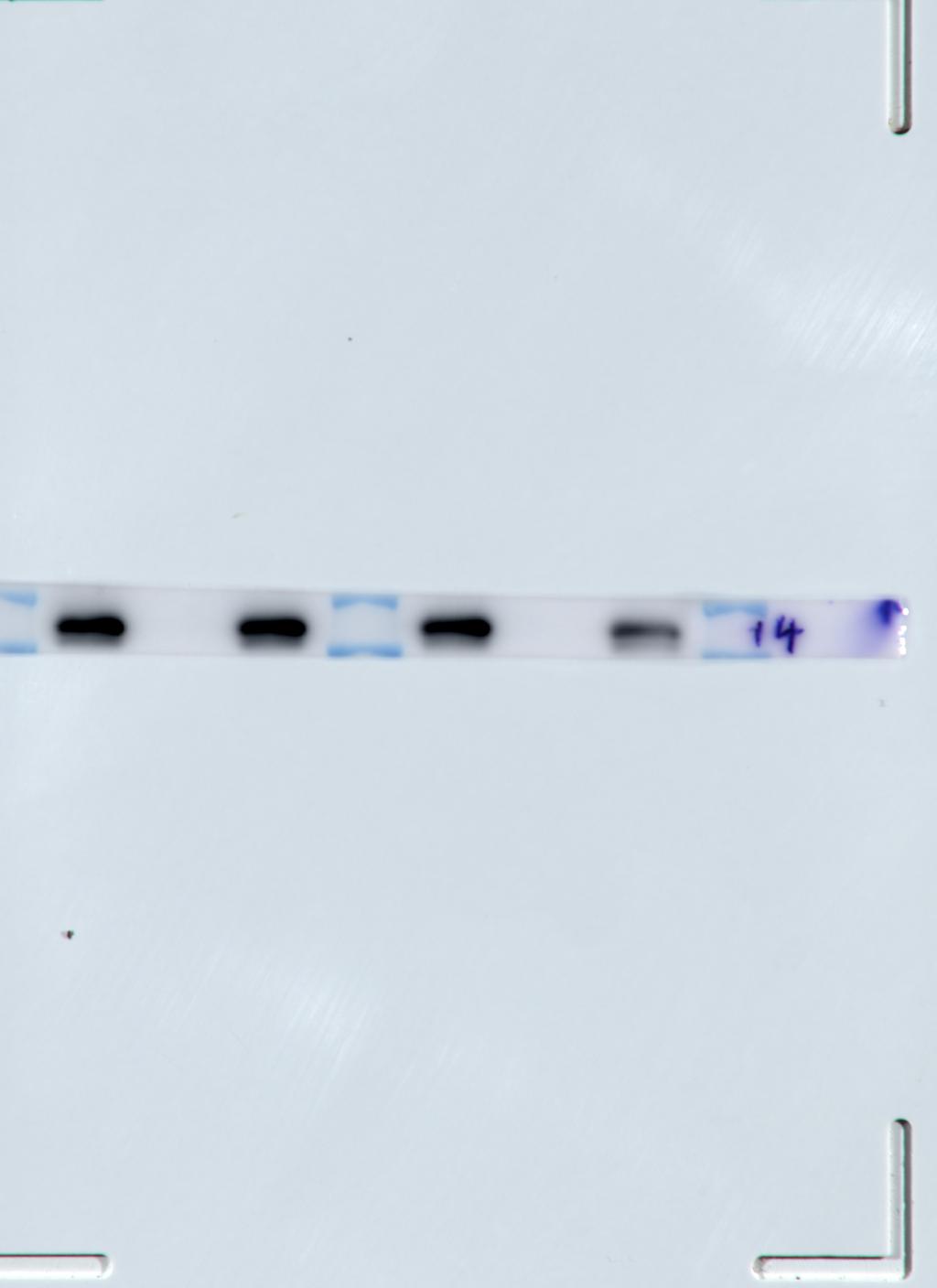
 100kDa

70kDa

TRAF6
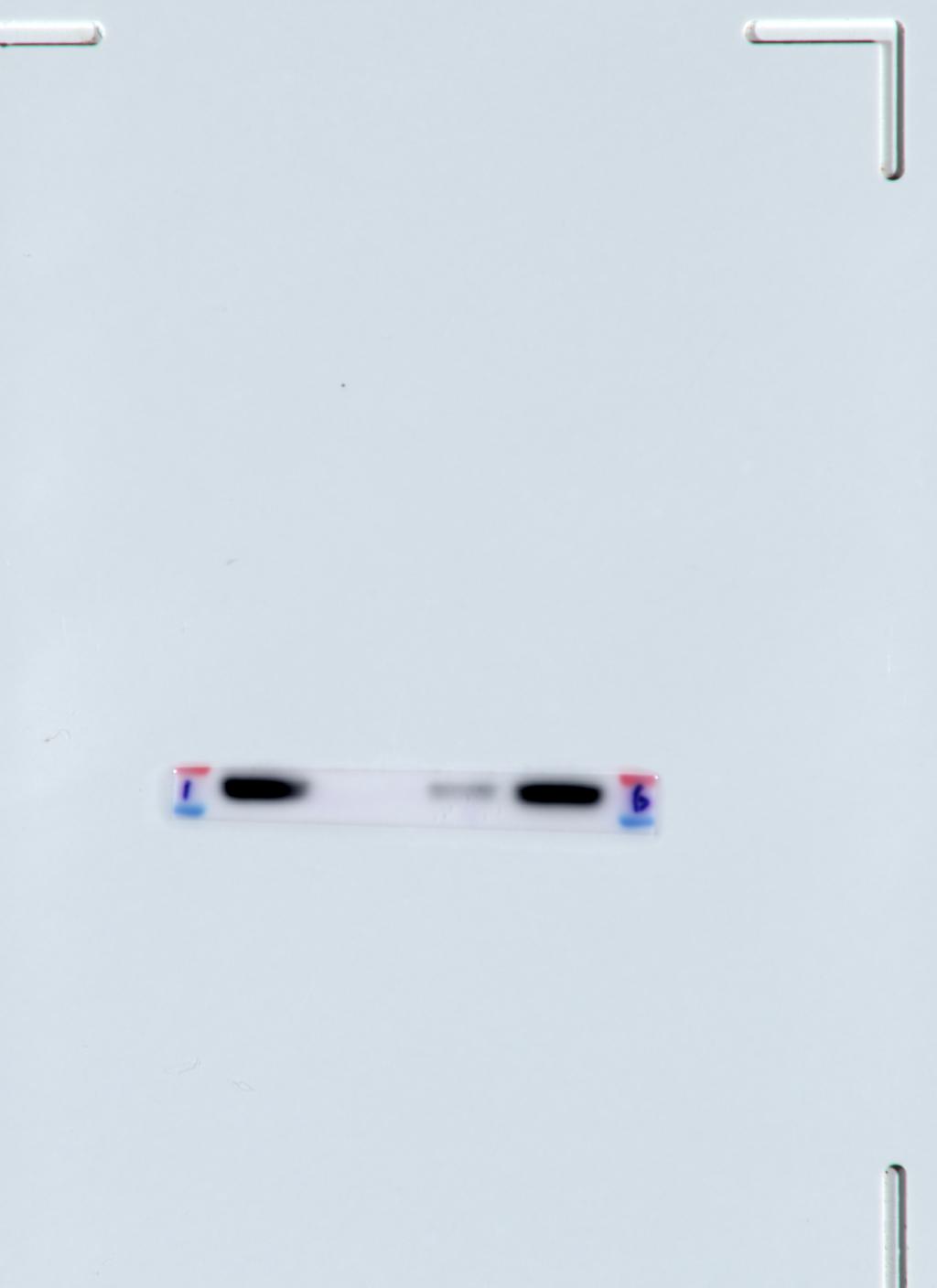
 55kDa

130kDa

TLR4
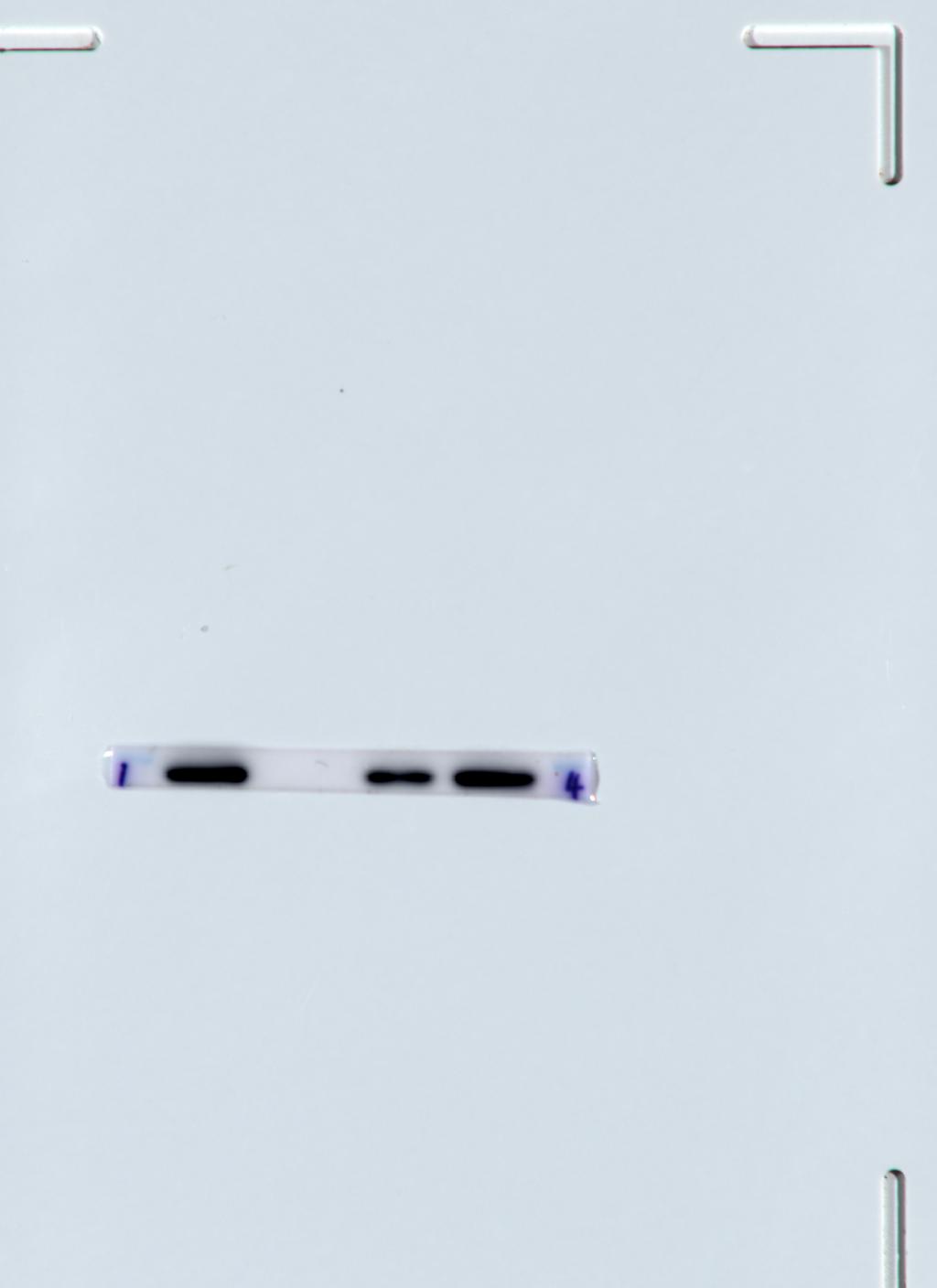
 100kDa

70kDa

TRAF6
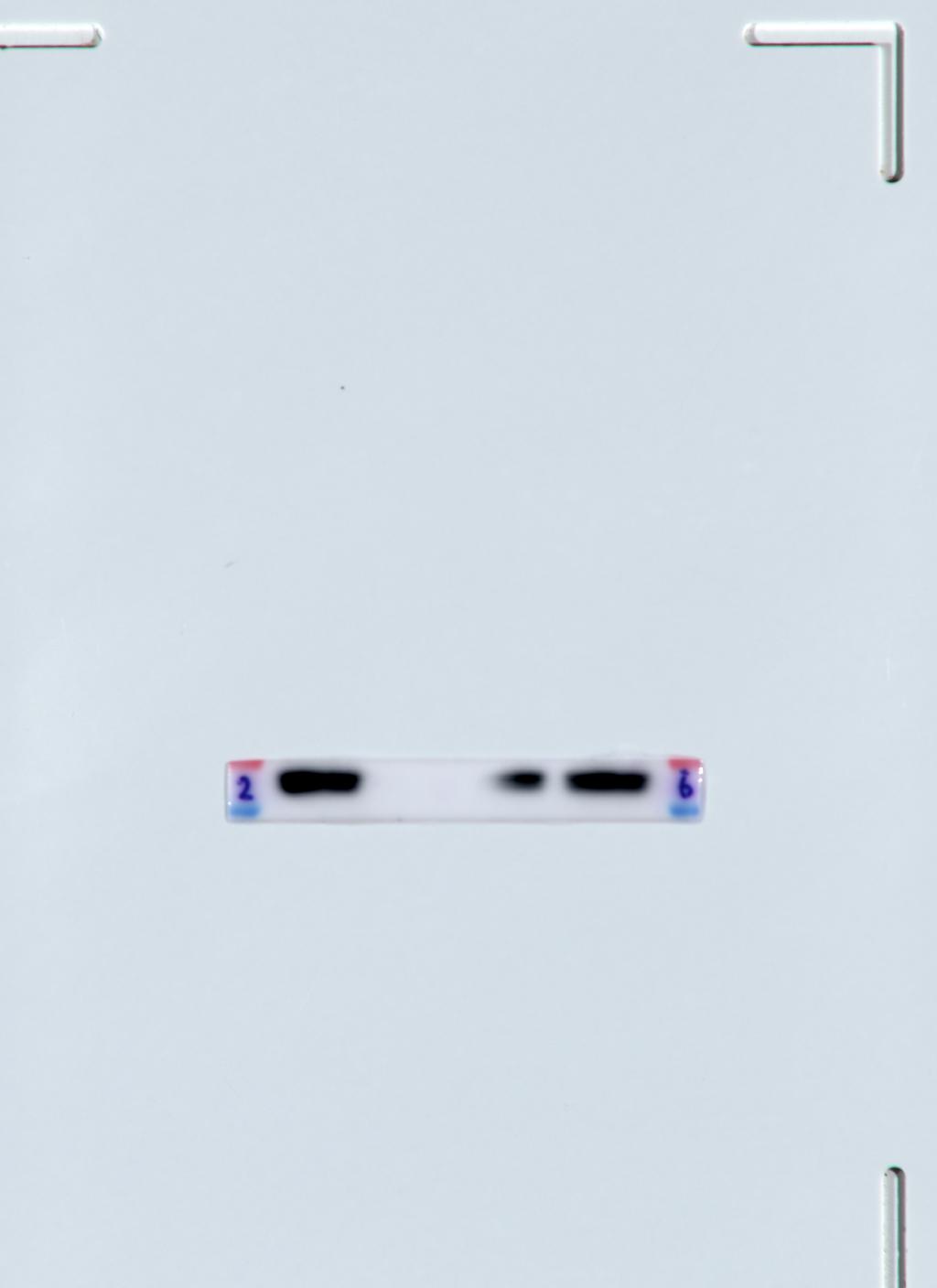
 55kDa

130kDa

TLR4
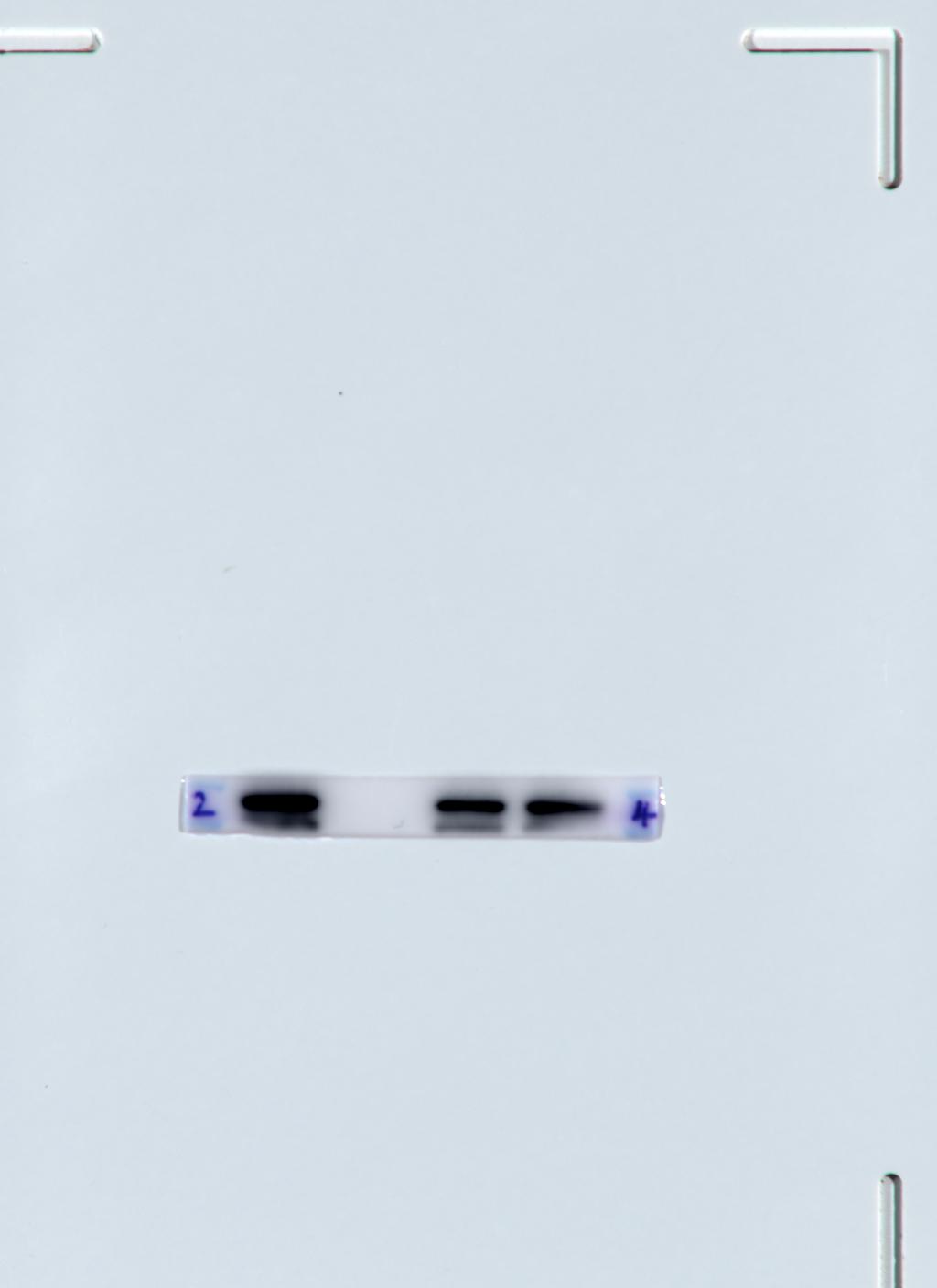
 100kDa

70kDa

TRAF6 55kDa

130kDa

TLR4 100kDa

70kDa

TRAF6 55kDa

130kDa

TLR4 100kDa

Figure 6

55kDa

TRIM59 40kDa

100kDa

E-Cad 70kDa

180kDa

N-Cad 130kDa

70kDa

VIM 55kDa

40kDa

GAPDH 35kDa

55kDa

TRIM59 40kDa

70kDa

P-NF-κB 55kDa

70kDa

NF-κB 55kDa

40kDa

TIRAP 35kDa

130kDa

TLR4 100kDa

70kDa

TRAF6 55kDa

40kDa

GAPDH 35kDa

Figure 7

55kDa

TRIM59 40kDa

40kDa

GAPDH 35kDa

55kDa

TRIM59 40kDa

40kDa

GAPDH 35kDa

55kDa

TRIM59 40kDa

40kDa

GAPDH 35kDa

55kDa

TRIM59 40kDa

40kDa

GAPDH 35kDa

Fig S2

55kDa

POU5F1 40kDa

40kDa

PCNA 35kDa

100kDa

E-Cad 70kDa

180kDa

N-Cad 130kDa

70kDa

VIM 55kDa

40kDa

GAPDH 35kDa

55kDa

POU5F1 40kDa

40kDa

PCNA 35kDa

100kDa

E-Cad 70kDa

180kDa

N-Cad 130kDa

70kDa

VIM 55kDa

40kDa

GAPDH 35kDa

Figure S3

55kDa

POU5F1 40kDa

100kDa

E-Cad 70kDa

180kDa

N-Cad 130kDa

70kDa

VIM 55kDa

40kDa

GAPDH 35kDa

Figure S4

55kDa

TRIM59 40kDa

40kDa

PCNA 35kDa

100kDa

E-CAD 70kDa

180kDa

N-CAD 130kDa

70kDa

VIM 55kDa

40kDa

GAPDH 35kDa

70kDa

TRAF6 55kDa

40kDa

PCNA 35kDa

100kDa

E-CAD 70kDa

180kDa

N-CAD 130kDa

70kDa

VIM 55kDa

40kDa

GAPDH 35kDa

Figure S5

130kDa

TLR4 100kDa

40kDa

PCNA 35kDa

100kDa

E-Cad 70kDa

180kDa

N-Cad 130kDa

70kDa

VIM 55kDa

40kDa

GAPDH 35kDa
